# Evoked pleasure and approach-avoidance in response to pollution

**DOI:** 10.1371/journal.pone.0234210

**Published:** 2020-06-25

**Authors:** Anaïs Beaumont, Thierry Lelard, Harold Mouras, Sylvie Granon

**Affiliations:** 1 UR-UPJV: LNFP 4559, Laboratoire de Neurosciences Fonctionnelles et Pathologies, Centre Universitaire de Recherche en Santé, Amiens, France; 2 Institut des Neurosciences Paris-Saclay, Centre National de la Recherche Scientifique, Équipe Neurobiologie de la Prise de Décision, Gif-sur-Yvette, France; 3 UR-UPJV: APERE 3300, Adaptations Physiologiques à l’Exercice et Réadaptation à l’Effort, UFR des Sciences du Sport, Université de Picardie-Jules-Verne, allée P. Grousset, Amiens, France; University of Bologna, ITALY

## Abstract

From year-to-year, environment is becoming one of the major concerns of human societies. Few studies have investigated the biological processes involved in environmental scene perception. Here, we initiate a line of research by beginning to study emotional processes involved in this perception. Our results demonstrate a clear distinction between “Clean” and “Polluted” environments according to the pleasure and approach desire ratings they induced. Moreover, women expressed higher pleasure in the “Clean” condition, as did older participants. Finally, rural scenes induced higher pleasure in participants than urban ones.

## Introduction

Since several decades, numerous studies have supported the evidence of a close link between perceptual and motor (underlying action) processes. As stated in a recent review [1], many disciplines (e.g., psychology, philosophy, and neuroscience) have underlined how actions—and, therefore, motor processes—participate intrinsically in all aspects of visual experience. Historically, phenomenology [2, 3] has underlined the importance of sensations and the whole body for visual experience. Regarding the biological processes involved, and in addition to phenomenology and enaction [4], when conceiving the embedding of the perceiver in a *lifeworld*, cognitive and motor processes should participate in the construction of the whole visual experience. Therefore, regarding landscapes and the space representation that they involve, it seems reasonable to infer that not only the physical features of a landscape affect its neural and psychological representation, but so do many other mechanisms, such as emotional processes (through, for example, the emotional/agreeable valence attributed to a landscape). Several previous studies have demonstrated the influence of emotional information on motor reactions [such as in posturography; 5, 6, 7, 8].

In this framework, this study questions the interrelation between the motor and affective components of behavior [5]. We have explored this issue in the introductions of several of our studies [5, 6, 7, 8], and it has been studied elsewhere. For example, Darwin proposed that emotion adapts behavior to the context [9]. Numerous studies have supported the postulation that emotions influence motor processes [10, 11, 12, 13]. The connection between emotion and motor control is included by some theorists as foremost in the definition of emotion [14], using the appetitive/defensive ratings they induced for their classification. In addition, several studies have described the importance of incarnation (i.e., the involvement of the perceiver in the perceived scene) to modulate the motor correlates of emotional processing. Successively, we have been able to report (i) a freezing-type response when viewing painful stimuli compared to non-painful ones [5]; (ii) a higher modulation of posturographic responses when perceivers project themselves into the situation (i.e., when they have embodied the situation; Lelard et al., [6]). Moreover, examining the temporality of such responses [15] allowed to identify complex modulatory effects, supporting the idea of a differential modulation of emotion on motor responses throughout the 12 seconds of an emotional picture presentation period. Altogether, these results are consistent with the idea of an emotional re-shaping of the representation of visual scenes.

Environment has been of major concern worldwide. At many levels, this concern is about how policies can be developed to influence pro-environmental behaviors. In consumer societies, pollution appears to be a major problem and contributes significantly to the degradation of the environment. An important question is to assess how humans perceive environmental pollution. It seems natural to think that the perception of polluted environmental scenes is an unpleasant experience that one wishes to shorten through a reaction involving an interaction between emotional and motor processes. No scientific study has addressed the issue of biological circuits involved in the perceptual and cognitive processing of pollution scenes that humans may face daily. Moreover, as underlined above, to fully explore these mechanisms, a consideration of the emotional component that is potentially associated with pollution seems to be crucial. For example, the valence attributed to an environmental scene could potentially determine actions (i.e., approaching/avoiding), which can, in turn, re-shape the psychological and neural representations of environmental scenes.

The present study aims to explore the pleasure and approach/avoidance mechanisms evoked by stimuli on the issue of pollution. To achieve this, we tested potential parameters, such as inter-individual parameters or scene characteristics (i.e., environmental features and the presence of individuals) that could have an impact on the perception of the pollution concept. Our main hypothesis was that emotional content attributed to polluted scenes influences subjective rating, leading to action tendencies toward these landscapes.

## Material and methods

### Participants

Fifty-one volunteers (twenty-three men and twenty-eight women, mean age: 35.87 ± 13.84 years old) with no known visual or motor impairment and no previous or current treatments for psychiatric or neurologic disorders were included in the study. Each participant signed an informed consent form. Experimental procedures were conducted in accordance with the ethical standards of the Declaration of Helsinki and were approved by the local ethics committee (CER Université Paris Saclay, Orsay, France).

### Procedure about the construction of the photograph dataset

**Fig 1:**
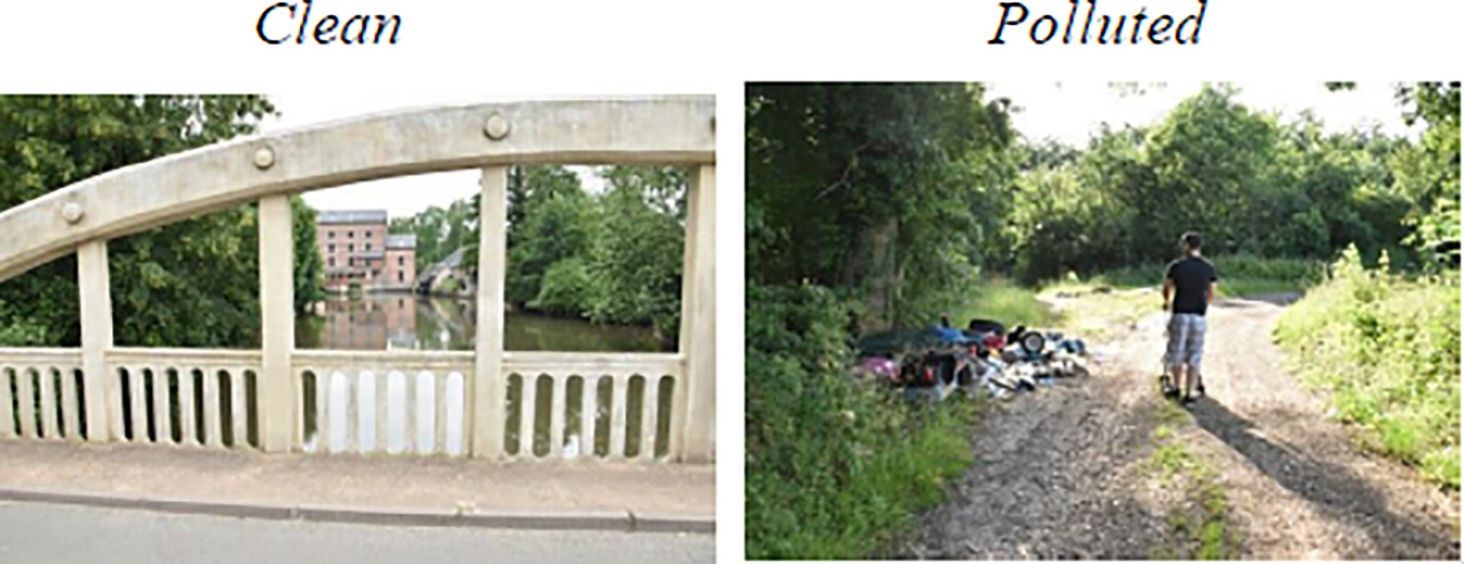
Examples of pictures for each condition. A. Scene of the “Clean” condition. B. Scene of the “Polluted” condition.

**Fig 2:**
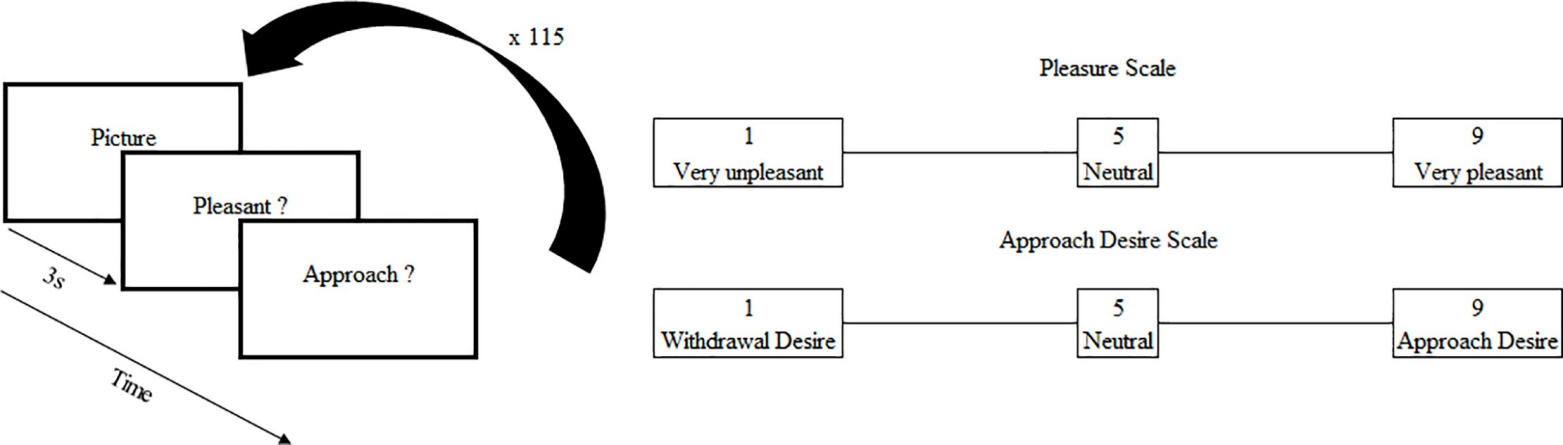
A. Stimuli presentation protocol. Each scene is presented for 3 seconds and is followed, each time, by the same two questions. First, participants had to evaluate, on a scale between 1 and 9, how much pleasant the scene was. Then, on the same scale, they had to evaluate how much this scene encouraged them to be in the situation (approach desire). B. Pleasure and approach desire scales.

One-hundred and sixteen static visual stimuli were ordered to a professional photographer (Adélie Granon) thanks to the grant mentioned in the acknowledgments meaning that all pictures pertained to the Principal Investigator of the study (S. Granon). The photograph search for rural or urban landscapes of our daily living environment. For the “Clean” condition, no waste was included in the pictures whereas for the “Polluted” condition the landscapes incorporated visible waste from the point of view of the photographer (from a path or close to a road). Hereafter, all the pictures were included in the following steps of the experiment. They defined two conditions: “Clean” and “Polluted” (for an example, see Fig 1). Stimuli were randomly presented through the E-Prime 2 Software (Psychology Software Tools, Inc., Pittsburgh, PA, USA; Fig 2). Each stimulus was presented for 3 seconds and was followed by 2 questions, always presented in the same order. Participants had to answer them with a pressure on the keyboard with no time limit. However, we asked them to respond as quickly as possible. For each scene, participants had to quantify on a Likert-type scale between 1 and 9 the pleasure (question 1) and the approach desire (question 2) evoked by the presented scene.

### Data recording and analyses

#### Pictures selection

First, we conducted a global analysis on the pleasure and approach desire that were evoked by each condition. The goal was to check if scenes of the “Clean” condition evoked a higher pleasure but also a higher approach desire than the one of the “Polluted” condition. To do so, we did a repeated-measures one-way ANOVA on the data. When sphericity was not accepted, a Greenhouse-Geisser (GG) correction was applied. Then, we conducted a per-picture analysis in order to extract the most relevant pictures for each condition. For the “Clean” condition, we set a threshold at 6 in order to extract scenes that will have a mean evoked pleasure and approach desire statistically higher than 6 and not only neutral (around 5). For the “Polluted” condition, we set a threshold at 4 in order to extract scenes that will have a mean evoked pleasure and a mean evoked approach desire statistically lower than 4 and not only neutral. For each scene, we conducted a t-test for paired measures. The statistical significance was set for p-value < 0.05.

#### Population and pictures characteristics effects

We performed analyses in search for picture characteristics (urban/rural context, presence of other individuals) and population characteristics (gender, categories of age) that could impact ratings. For each case, we started by a two-factor ANOVA with repeated measures on the data upon the environmental features (urban vs rural), the presence of individuals and the gender. For age's categories, we conducted a three-way ANOVA with repeated measures. When an interaction was found between the condition (“Clean” vs “Polluted”) and another criterion, we checked the impact of this criterion in each condition using a repeated-measure one-way ANOVA. When sphericity was not accepted, a Greenhouse-Geisser (GG) correction was applied. When an impact of categories of age was found, we conducted a post-hoc analysis. The statistical significance was set for p-value < 0.05.

## Results

The F, p and effect size of the main analyzes are reported in S1 Table.

### Analysis on the mean data

**Fig 3:**
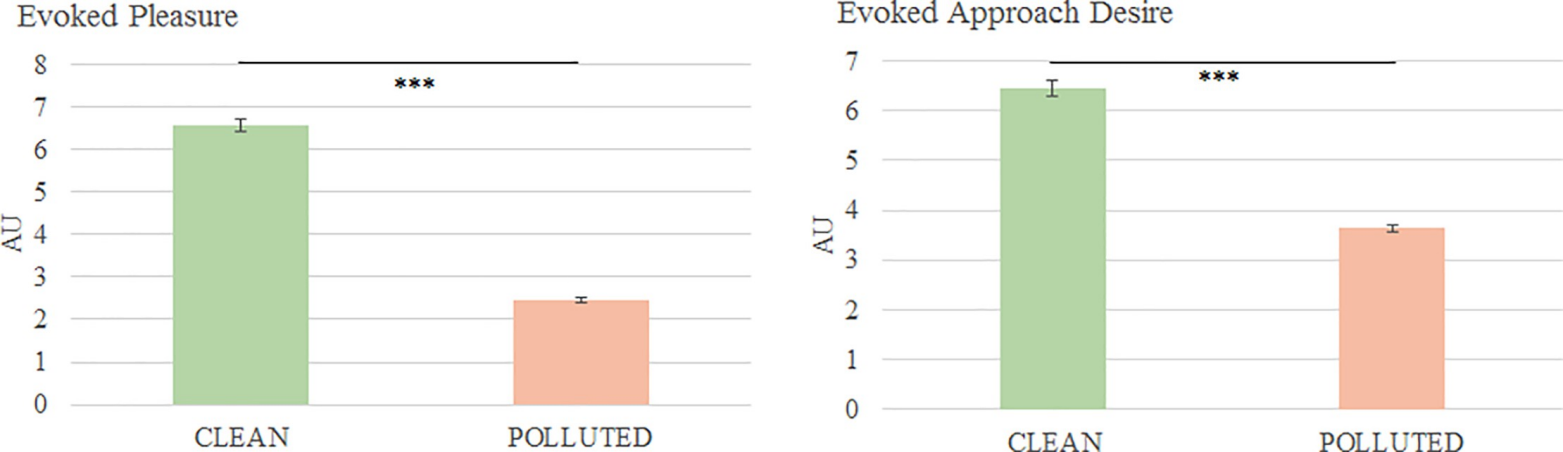
Mean evoked pleasure and approach desire (mean ± SEM). Results obtained on N = 58 pictures by condition. A. Mean evoked pleasure. The mean evoked pleasure in the “Clean” condition is statistically higher than for the “Polluted” condition (p = 1.41 x 10^−30^). B. Mean approach desire. Pictures of the “Clean” condition evoked a mean approach desire that is statistically higher than the one evoked by the pictures of the “Polluted” condition (p = 7.99 x 10^−15^). Significant differences are shown as *** p < 0.001.

The “Clean” condition evoked a higher pleasure and approach desire than the “Polluted” condition (p < 0.0001 in each case; Fig 3).

### Per-picture analysis—picture selection

**Fig 4:**
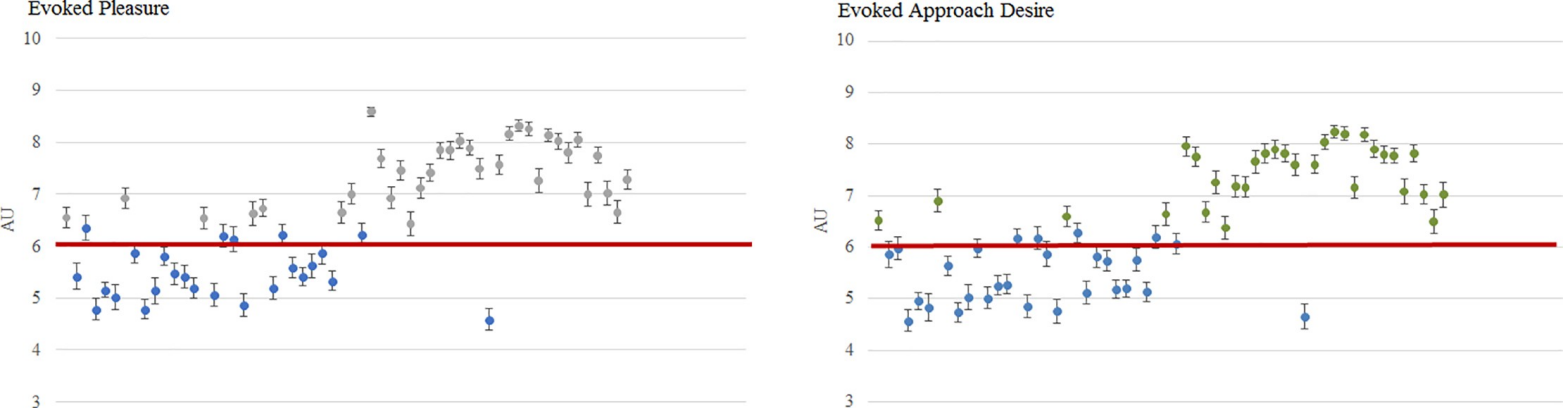
Pleasure and approach desire evoked by pictures of the “Clean” condition (mean ± SEM). Results obtained on N = 51 participants. Each dot represents a picture of the “Clean” condition. The red line represents the threshold that is set at 6. A. Pleasure evoked by pictures of the “Clean” condition. Each grey dot represent a picture of the “Clean” condition that induce an evoked pleasure that was statistically higher than 6. B. Approach desire evoked by pictures of the “Clean” condition. Each green dot represents a picture of the “Clean” condition that induced an evoked approach desire but also an evoked pleasure that was statistically higher than 6. In each case, blue dots represent pictures that were not statistically relevant for the pleasure rating.

Our goal was to select for future studies the most relevant pictures among the ones we collected in each condition, that is to say the pictures for which we haven't any ambiguous ratings. In the “Clean” condition, thirty-three pictures were evaluated with an evoked pleasure that was statistically higher than 6 [Fig 4]. Based on our inclusion criteria, we looked for the evaluation of the pictures according to the approach desire. Among the thirty-three pictures that have a satisfying evoked pleasure, only thirty have an evoked approach desire that were statistically higher than 6 [Fig 4]. We have decided this threshold of 6 in order to avoid scenes that would be evaluated as neutral (with a rating around 5).

**Fig 5:**
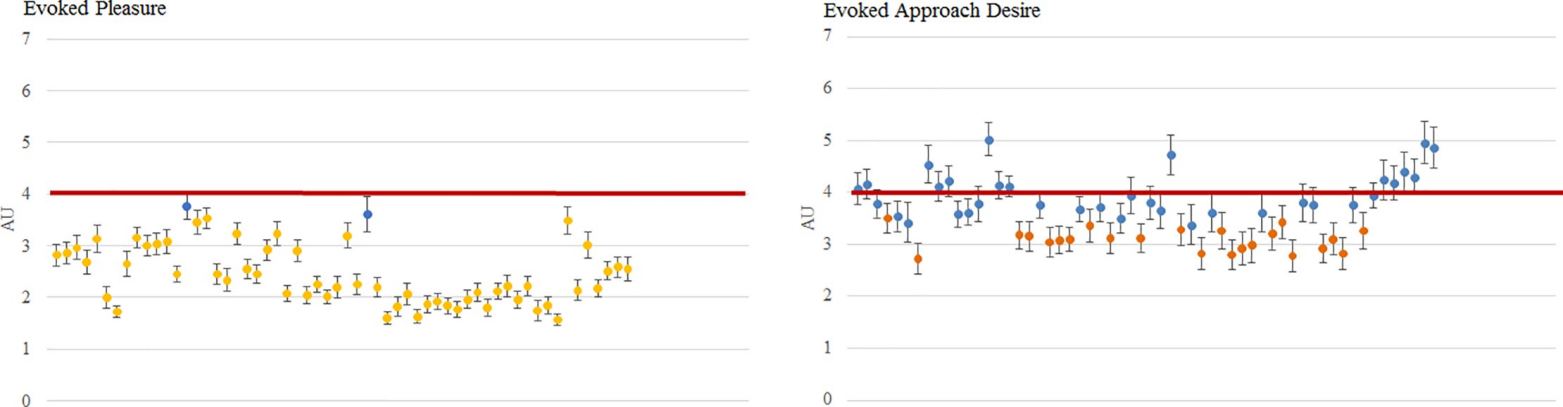
Pleasure and approach desire evoked by pictures of the “Polluted” condition (mean ± SEM). Results obtained on N = 51 participants. Each dot represents a picture of the “Polluted” condition. The red line represents the threshold that is set at 4. A. Pleasure evoked by pictures of the “Polluted” condition. Each yellow dot represent a picture of the “Polluted” condition that induce an evoked pleasure that was statistically lower than 4. B. Approach desire evoked by pictures of the “Polluted” condition. Each orange dot represent a picture of the “Polluted” condition that induced an evoked approach desire but also an evoked pleasure that was statistically lower than 4. In each case, blue dots represent pictures that were not statistically relevant for the pleasure rating.

In the “Polluted” condition, fifty-six scenes were evaluated with an evoked pleasure that is statistically lower than 4 [Fig 5]. Based on our inclusion criteria, we looked for the evaluation of the pictures according to the approach desire ratings. Among the fifty-six scenes evaluated with a satisfying evoked pleasure, only twenty-three had an evoked approach desire that was statistically lower than 4 [Fig 5]. We set this threshold at 4 in order to avoid pictures that would be evaluated as neutral (with a rating around 5).

### Individual and picture characteristics effects

Based on our analysis and on the participant feedback, it seems that the approach desire concept leads to ambiguous results (see the Discussion section below). Therefore, for all subsequent analyses, we focused on data obtained for the evoked pleasure evaluation.

#### Urban VS rural landscapes

**Fig 6:**
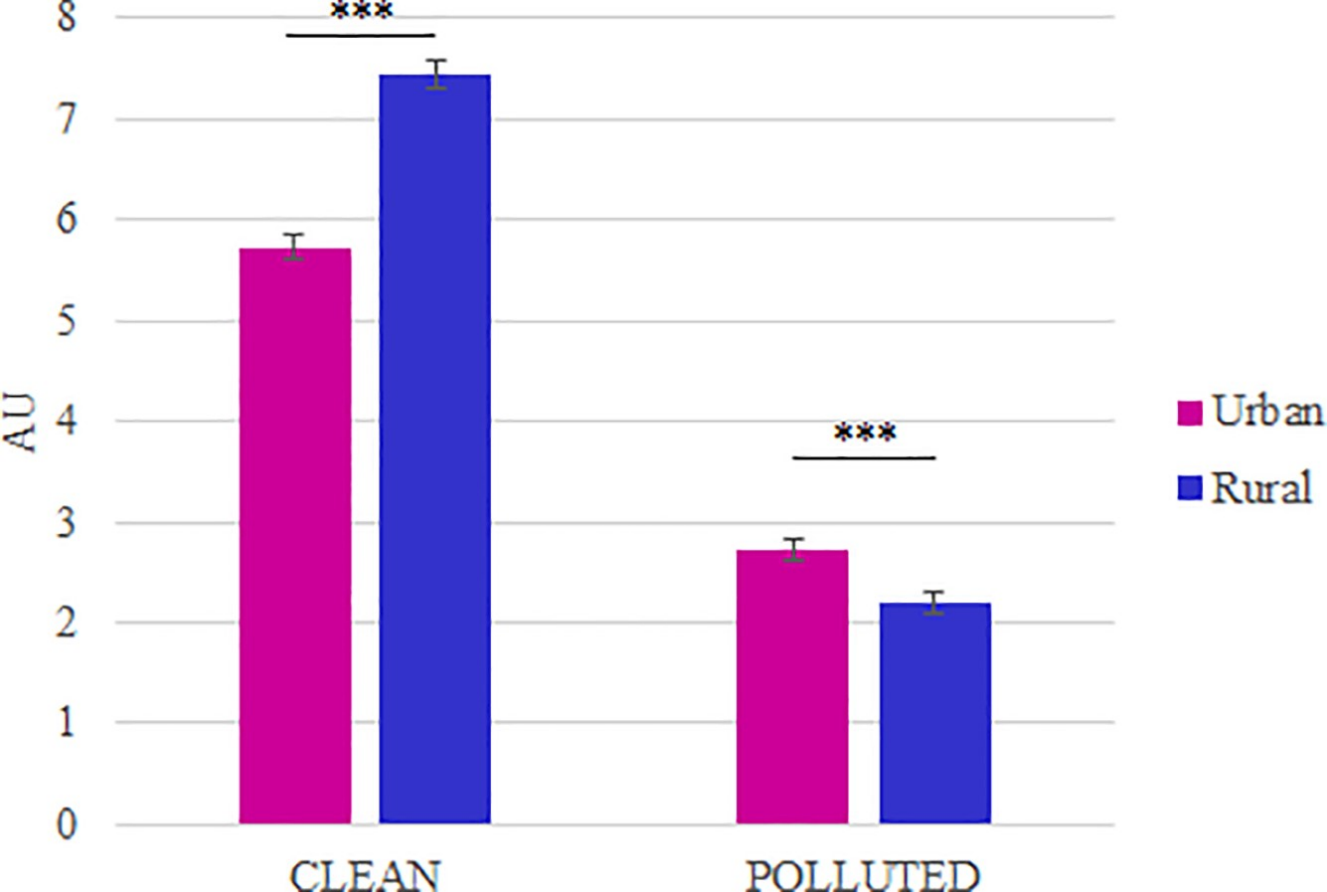
Mean evoked pleasure according to the environmental context (Urban VS Rural) in the “Clean” and “Polluted” conditions (mean ± SEM). In each condition, we have 29 pictures in an urban context and 29 pictures in a rural context. We found an interaction effect between condition and context (p = 6.63 x 10^−23^). Results showed an effect of environmental context in the “Clean” condition (p = 4.62 x 10^−23^) but also in the “Polluted” condition (p = 5.32 x 10^−08^). Significant differences are shown as *** p < 0.001.

Our results showed an interaction effect between the “Condition” factor and the environmental context (urban vs rural; p<0.0001). We found an effect of the environmental context in the “Clean” condition (p<0.0001) and in the “Polluted” condition (p<0.0001) [Fig 6]. Thus, in the “Clean” condition, the evoked pleasure is statistically higher in a rural context compared to an urban one. In the same way, in the “Polluted” condition, the evoked pleasure was significantly lower in a rural context compared to an urban one.

#### Gender effect

**Fig 7:**
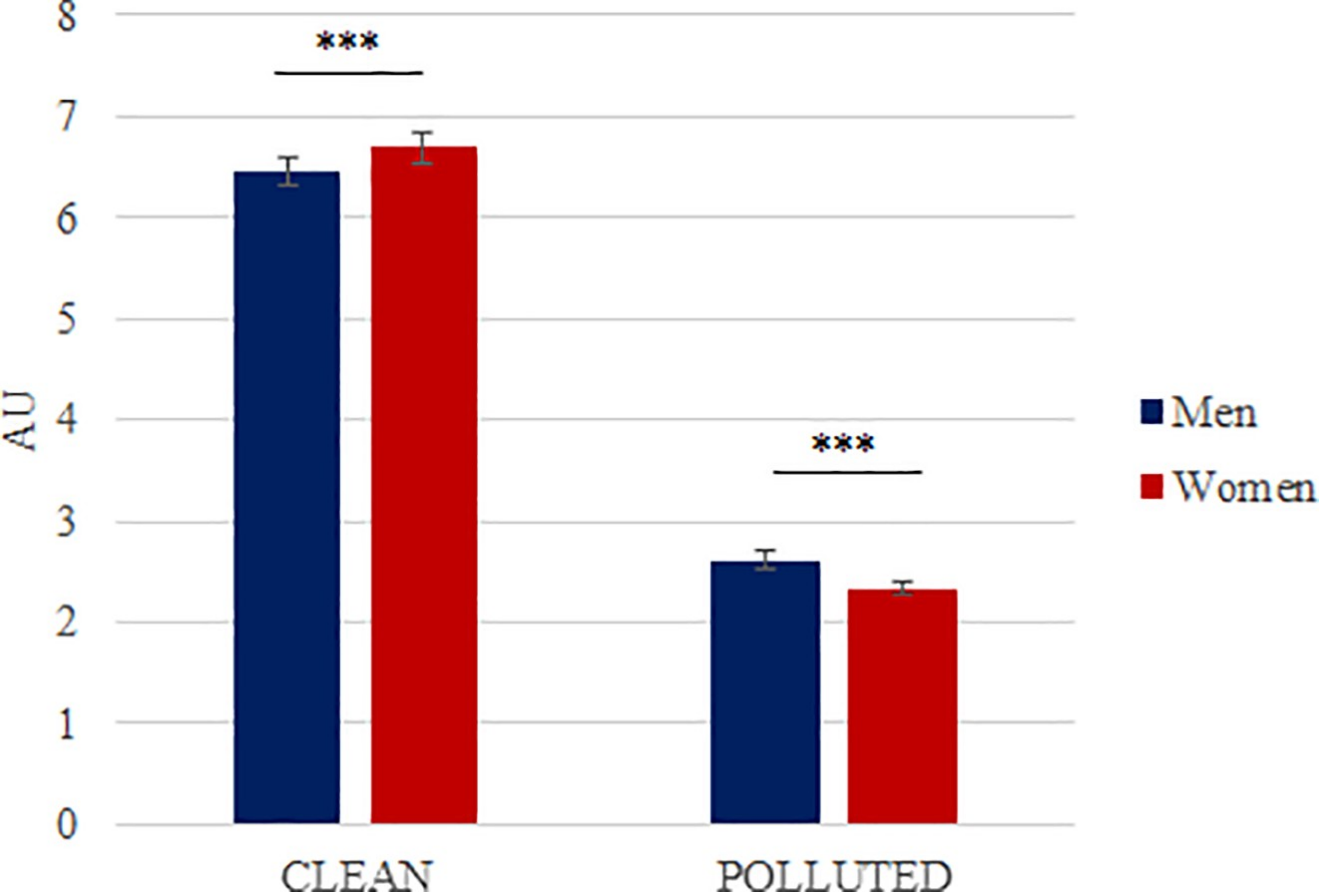
Mean evoked pleasure according to gender in the “Clean” and “Polluted” conditions (mean ± SEM). Results obtained on N = 58 pictures. We found an interaction effect between condition and gender (p = 3.50 x 10^−09^). Results showed an effect of gender in the “Clean” condition (p = 3.10 x 10^−05^) but also in the “Polluted” condition (p = 3.14 x 10^−06^). Significant differences are shown as *** p < 0.001.

Our analysis on the evoked pleasure showed an interaction effect between the “Condition” factor and gender (p<0.0001). We found a gender effect in the “Clean” condition (p<0.0001) but also in the “Polluted” condition (p<0.0001) but effects were in opposite directions [Fig 7]. Indeed, women express a pleasure significantly higher in front of a “Clean” condition pictures and a pleasure significantly lower in front of pictures of the “Polluted” condition than men.

#### Age effect

**Fig 8:**
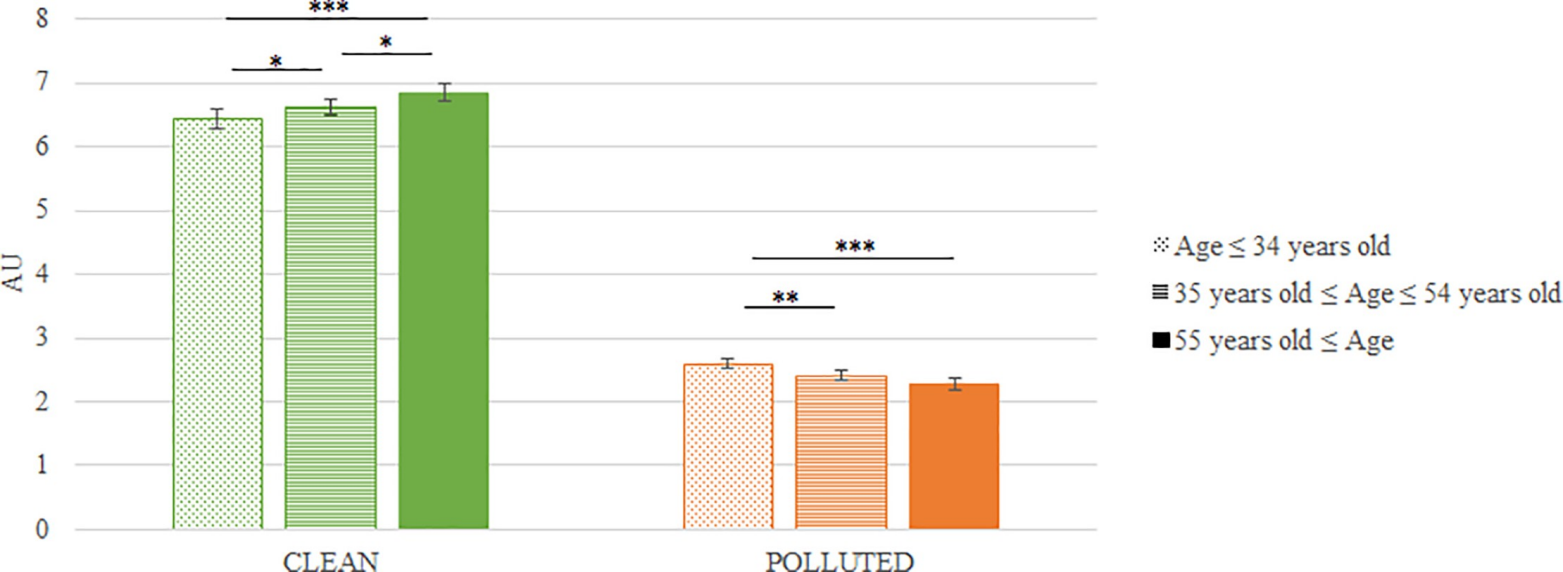
Mean evoked pleasure according to age's categories in the “Clean” and “Polluted” conditions (mean ± SEM). Results obtained on N = 58 pictures. Results showed an interaction effect between condition and age (p = 1.94 x 10^−15^). “Clean” condition: we found an effect of the category of age (p = 3.18 x 10^−08^) with a difference “Less than 34 years old” vs “35 to 54 years old” (p = 0.04), “Less than 34 years old” vs “More than 55 years old” (p = 1.1 x 10^−06^) and “35 to 54 years old” vs “More than 55 years old” (p = 0.02). “Polluted” condition: we found an effect of the category of age (p = 6.1 x 10^−15^) with a difference “Less than 34 years old” vs “35 to 54 years old” (p = 0.006) and “Less than 34 years old” vs “More than 55 years old” (p = 2.4 x 10^−06^) but no difference “35 to 54 years old” vs “More than 55 years old” (p = 0.10). Significant differences are shown as * p < 0.05, ** p < 0.01 and *** p < 0.001.

Our results on the evoked pleasure showed a significant interaction between the “Condition” factor and age categories (p<0.0001). In the “Clean” condition, we found an impact of age (p<0.0001) and post-hoc analyses revealed significant differences between “Less than 34 years old” and “35 to 54 years old” categories (p = 0.04), between “Less than 34 years old” and “More than 55 years old” categories (1.1 x 10^−06^) and between “35 to 54 years old” and “More than 55 years old” categories (p = 0.02). In the “Polluted” condition, we found an impact of age (p = 6.1 x 10^−15^) and post-hoc analyses revealed significant differences between “Less than 34 years old” and “35 to 54 years old” categories (p = 0.006), between “Less than 34 years old” and “More than 55 years old” categories (2.4 x 10^−06^) but no significant difference between “35 to 54 years old” and “More than 55 years old” categories (p = 0.1) [Fig 8].

## Discussion

We established a clear distinction between “Clean” environments and “Polluted” ones according to the pleasure and approach desire ratings they induced. Based on our inclusion criteria, we selected 30 scenes for the “Clean” condition and 23 for the “Polluted” condition from a larger pictures database. Based on our observations and the participants’ debriefing, the concept of approach/withdrawal and its relevance must be reevaluated in the context of environmental pollution. Indeed, in front of scenes estimated by the participants to be “weakly” polluted, participants expressed a natural tendency to go forward, as if to act (see the picture named Urb.POLLUTED23 in the S1 Data). This approach desire led to a reduction in the number of scenes that could be included in the “Polluted” condition. When we suppressed this approach/withdrawal index, we included 33 scenes in the “Clean” condition and 56 in the “Polluted” condition. This observation raised the question of the relevance of this approach/withdrawal index in this context, despite its traditional use for the evaluation of emotional pictures. Moreover, our results allowed to characterize a third category of more neutral images that induce neither significant pleasure/displeasure nor approach/withdrawal desire. This type of scene will be useful to the pursuit of our project, particularly in posturographic studies, in which this scene category will serve as a control for motor activity. Other studies have provided interesting results on the modulation of motor control by emotional stimuli regarding the approach-withdrawal index. Borgomaneri et al. [16] provided neurophysiological support that emotion perception is closely linked to action systems and that negative events require motor reactions to be more urgently mobilized. More recently, Fini et al. [17] demonstrated that stimulus valence directly elicits specific action tendencies very early (already at 400 ms) with a necessary visual feedback to occur. These results will be of particular interest for further studies addressing the question of the postural responses to pollution.

We then tested the impact of some characteristics of the presented scene, such as urban versus rural features, on participants. Our results showed that in the “Clean” condition, rural scenes evoked higher pleasure than urban ones, whereas, in the “Polluted” condition, rural scenes evoked significantly lower pleasure than urban ones. In rural polluted scenes, the emotional content seemed to have a stronger negative impact on participants. Here, a limitation of the study is a Clean/Polluted–Rural/Urban potential confound through an association between Clean to Rural and Polluted to Urban environments. To avoid this limitation, further studies should only take into account pictures pertaining to one condition, urban or rural.

We further pursued our analyses to identify potential differences in terms of population characteristics such as gender (male versus female) or age categories (less than 34 years old, between 35 and 54 years old, and over 55 years old). Our analyses revealed that women expressed higher pleasure in the “Clean” condition and lower pleasure in the “Polluted” condition than men. Female participants tended to be more sensitive to the emotional content of the environmental condition. This capacity for empathy could explain a stronger link between women and their environment, making them more sensitive to pollution. We also identified an impact of age on the results. In the “Clean” and “Polluted” conditions, older participants reported respectively more and less pleasure than younger participants. Thus, sensitivity to environmental conditions seems to increase with age. However, additional studies with larger numbers of subjects would be necessary to verify whether other factors can be added to age. The youngest participants are likely to be more accustomed to pollution in the environment than the older ones and will have grown up with the politics of protection toward the environment. However, the elderly are likely to have experienced more environmental change. Their knowledge of earlier environmental conditions may impact the way that they perceive the environment and their understanding of the importance of an increased consideration of environment in society.

A landscape is widely recognized as a multilayered concept that encompasses both objective and subjective dimensions [18, 19, 20, 21, 22, 23]. In 1976, Meinig suggested that any landscape “is composed not only of what lies before our eyes but what lies within our heads” [24]. He focused attention on the significance that individuals attach to landscapes, whereby sensory inputs seem to be modified by personal history, making landscape perception an active construction of the human brain.

Traditionally, perception has been defined as the way in which an individual observes, understands, interprets, and evaluates an object, action, experience, individual, policy or outcome [25, 26, 27, 28, 29]. Multiple factors, such as context (e.g., culture or livelihood), past experiences, individual and collective attributes (e.g., gender or ethnicity), values, norms, beliefs, preferences, knowledge, and motivations mediate and influence perceptions [30, 31, 32, 33, 34]. As a result, different individuals can perceive the same situation in vastly different ways. Moreover, perceptions can change over time, and judgments are subject to persuasion [33]. These ideas correspond with our study’s results, which reveal a direct impact of gender and age on the expression of pleasure induced by environmental images. Indeed, gender and age impact perception and directly affect individuals’ interactions with situations.

As noted in the introduction, the internal representation of the environment is influenced by former experiences. This representation influences interpretation of the real environment but also behavior [35]. In the course of their lives, people develop intricate and rich cognitive structures that embody their visions of nature and its relationship with humans. In this framework, people could be placed on a continuum according to their views about the influence of humans in natural landscapes. This can explain how a rural context that has been less impacted by human activities could be characterized as more pleasant than an urban one that has been more impacted by humans (and indeed, literally constructed by humans).

One limitation of our experimental design in the natural context of environment appreciation is that viewing photographs is not similar to a first-hand experience of the presented visual scene. As noted by Susan Sontag, “Photographic images tend to substrate feeling from something we experience firsthand and the feelings they do arouse are, largely, not those we have in real life” [36]. Hodgson and Thayer expressed the idea that people may forge their own views of their environment, including what is outside the frame that is presented to them [36]. In our case, the perceiver is in a stationary position, like a camera at a fixed point in the environment. Thus, the perceiver is “remote” from the scene and is clearly a detached spectator rather than an engaged agent [37]. In these conditions, we cannot affirm that every participant imagined themselves into the presented situation as they may have done in genuine circumstances. The real experience of an environment requires a succession of temporally discrete stimuli inputs, which together provide information about what can be seen across a succession of eye fixations. This experienced “continuity” takes part in a mental constructive process. Using static representations (pictures) to explore landscape’s perception raised several questions. A first one is to determine whether human reactions to environments represented by pictures are valid indicators of reactions that would occur if people were to visit those environments and view them directly. Consistently, correlations between photo-based and direct “on-site” assessments have been of 0.80 or greater [38]. From an ecological point of view, landscapes are conceptualized as arenas for action. By imagining themselves as actors in the presented situation, someone’s experience of perception appeals to active engagement, leading to the consideration of the functional possibility that the observed landscape could afford. This also presents a new way of thinking about one’s experience with the landscape. Berleant’s [39] expression is instructive about this way of thinking: “Perceiving the environment from within, as it were, looking not at it but being in it, nature becomes something quite different. It is transformed into a realm in which we live as participants, not as observers” (Berleant, [39]: 83). By changing one’s status, one can change one’s way of thinking and one’s motivation to act. Therefore, encouraging people to change their connection with a situation could impact their perception of the environment and ultimately convince them to act in order to change what they see (if it is not what they want to see).

For landscape quality assessment, four components have been found to exert an effect on results: the sample of participants/observers used to represent a defined population of observers; the landscape representation medium (i.e., slides, prints, or on-site views); the observer’s answer format (e.g., paired-comparison, rankings, and ratings); and the time that respondents take to view the scenes [40]. Using psychophysical methods to assess the beauty of landscape scenes requires several factors to be taken into count. Firstly, scenic beauty is the perceptual and judgmental processes of a human observer in interaction with the relevant physical features of the landscape. Secondly, the perceived scenic beauty—which is not directly observable—must be inferred from overt evaluative responses made by observers. Thirdly, the perceptual judgments of the public provide an appropriate basis for assessing beauty and a public survey or consumer evaluation approach can be considered to be valid for assessing the scenic beauty of the public landscape [38]. Another model—the phenomenological model—places greater emphasis on individual subjective feelings, expectations, and interpretations in which landscape perception is conceptualized as an intimate encounter between a person and the environment [41].

Landscapes constitute highly complex stimuli whose perception requires a strong component of searching and selection of information by the subject. Many studies have focused on the relevant characteristics of a scene, such as the perceived naturalness of the scene. In a previous study, Kaplan and colleagues [42] reported a dramatic preference for nature scenes to urban scenes. In our experiment, we observed the same effect on the pleasure scale. The influence of the significance assigned to a landscape on an individual’s judgment of beauty could explain why a landscape that is thought to be human-influenced is judged to be less beautiful. Thus, the human evaluation of beauty cannot be predicted from the environmental characteristics alone. The preference for natural over human-influenced environments has been reported by previous cross-cultural [43, 44] and psychophysiological studies [45, 46].

In conclusion, this initial work on human reactions to the perception of pollution demonstrates that one’s perception of a “polluted” landscape can generate an emotional reaction that may promote an action. This first step is important in understanding the mismatch between the global social understanding that environmental pollution requires a behavioral change and the incapacity of long-term and significant changes in individuals’ behavior. The selection of relevant pictures of “Clean” and “Polluted” environmental scenes established in the current study may, therefore, constitute a solid basis for further studies.

## Supporting information

S1 Table (DOCX)

S1 Data (ZIP)

## References

[1] Menatti L., & Heft H. (2020). Editorial: Changing Perspectives on Landscape Perception: Seeking Common Ground Between the Psychological Sciences and the Humanities. *Frontiers in Psychology*, 11 10.3389/fpsyg.2020.00159 PMC7026469 32116955

[2] Husserl E. (1913). *Ideas*: *General Introduction to Pure Phenomenology*. Routledge.

[3] Merleau-Ponty M. (1962). *Phenomenology of Perception*. Routledge 10.4324/9780203981139

[4] Varela F. J., Thompson E., & Rosch E. (1991). *The embodied mind*: *Cognitive science and human experience* (p. xx, 308). The MIT Press.

[5] Lelard T., Montalan B., Morel M. F., Krystkowiak P., Ahmaidi S., Godefroy O., et al (2013). Postural correlates with painful situations. *Frontiers in Human Neuroscience*, 7 10.3389/fnhum.2013.00004 PMC3564009 23386816

[6] Lelard T., Godefroy O., Ahmaidi S., Krystkowiak P., & Mouras H. (2017). Mental Simulation of Painful Situations Has an Impact on Posture and Psychophysiological Parameters. *Frontiers in Psychology*, 8 10.3389/fpsyg.2017.02012 PMC5702461 29209250

[7] Lelard T., Stins J., & Mouras H. (2019). Postural responses to emotional visual stimuli. *Neurophysiologie Clinique = Clinical Neurophysiology*, 49(2), 109‑114. 10.1016/j.neucli.2019.01.005 30711434

[8] Mouras H. (2016). Effect of arousal on perception as studied through the lens of the motor correlates of sexual arousal. *Behavioral and Brain Sciences*, 39 10.1017/S0140525X15001880 28347369

[9] Darwin C. (1872). *The expression of the emotions in man and animals*. John Murray. 10.1037/10001-000

[10] Coombes S. A., Corcos D. M., Pavuluri M. N., & Vaillancourt D. E. (2012). Maintaining force control despite changes in emotional context engages dorsomedial prefrontal and premotor cortex. *Cerebral Cortex (New York*, *N*.*Y*.: *1991)*, 22(3), 616‑627. 10.1093/cercor/bhr141 PMC3278319 21677029

[11] Michalak J., Troje N. F., Fischer J., Vollmar P., Heidenreich T., & Schulte D. (2009). Embodiment of sadness and depression—Gait patterns associated with dysphoric mood. *Psychosomatic Medicine*, 71(5), 580‑587. 10.1097/PSY.0b013e3181a2515c 19414617

[12] Naugle K. M., Hass C. J., Joyner J., Coombes S. A., & Janelle C. M. (2011). Emotional state affects the initiation of forward gait. *Emotion (Washington*, *D*.*C*.*)*, 11(2), 267‑277. 10.1037/a0022577 21500896

[13] Schmidt L., Cléry-Melin M.-L., Lafargue G., Valabrègue R., Fossati P., Dubois B., et al (2009). Get aroused and be stronger: Emotional facilitation of physical effort in the human brain. *The Journal of Neuroscience*: *The Official Journal of the Society for Neuroscience*, 29(30), 9450‑9457. 10.1523/JNEUROSCI.1951-09.2009 19641108 PMC6666541

[14] Lang P. J., Bradley M. M., & Cuthbert B. N. (2008). *International Affective Picture System (IAPS)*: *Affective ratings of pictures and instruction manuel* [Technical Report A-8].

[15] Mouras H., & Lelard T. (2018). Importance of Temporal Analyzes for the Exploration of the Posturographic Correlates of Emotional Processing. *Frontiers in Behavioral Neuroscience*, 12, 277 10.3389/fnbeh.2018.00277 30498436 PMC6249305

[16] Borgomaneri S., Gazzola V., & Avenanti A. (2014). Temporal dynamics of motor cortex excitability during perception of natural emotional scenes. *Social Cognitive and Affective Neuroscience*, 9 *(*10*)*, 1451–1457 10.1093/scan/nst139 23945998 PMC4187264

[17] Fini C., Bardi L., Brass M., & Moors A. (2020, 1 19). Support from a TMS/MEP study for a direct link between positive/negative stimuli and approach/avoidance tendencies. 10.31234/osf.io/semv8

[18] Antrop M. (2000). Background concepts for integrated landscape analysis. *Agriculture*, *Ecosystems & Environment*, 77, 17‑28. 10.1016/S0167-8809(99)00089-4

[19] Hunziker M., Buchecker M., & Hartig T. (2007). *Space and Place–Two Aspects of the Human-landscape Relationship* (p. 47‑62). 10.1007/978-1-4020-4436-6_5

[20] Nassauer J. (2011). Care and stewardship: From home to planet. *Landscape and Urban Planning*, 100, 321‑323. 10.1016/j.landurbplan.2011.02.022

[21] Naveh Z. (2000). What is holistic landscape ecology? A conceptual introduction. *Landscape and Urban Planning*, 50, 7‑26. 10.1016/S0169-2046(00)00077-3

[22] Naveh Z. (2007). Landscape ecology and sustainability. *Landscape Ecology*, 22, 1437‑1440. 10.1007/s10980-007-9171-x

[23] Tveit M., Ode Sang Å., & Fry G. (2006). Key concepts in a framework for analysing visual landscape character. *Landscape Research—LANDSC RES*, 31, 229‑255. 10.1080/01426390600783269

[24] Meinig D. (1979). The Beholding Eye Ten Versions of the Same Scene. *The interpretation of ordinary landscapes*: *geographical essays*, 66.

[25] Bennett N. (2016). Using perceptions as evidence to improve conservation and environmental management. *Conservation Biology*, 30 10.1111/cobi.12681 26801337

[26] Fini C., Costantini M., & Committeri G. (2014). Sharing Space: The Presence of Other Bodies Extends the Space Judged as Near. *PLOS ONE*, 9(12), e114719 10.1371/journal.pone.0114719 25493627 PMC4262430

[27] Fini C., Brass M., & Committeri G. (2015). Social scaling of extrapersonal space: Target objects are judged as closer when the reference frame is a human agent with available movement potentialities. *Cognition*, 134, 50–56. 10.1016/j.cognition.2014.08.014 25460378

[28] Fini C., Bardi L., Troje N. F., Committeri G., & Brass M. (2017). Priming biological motion changes extrapersonal space categorization. *Acta Psychologica*, 172, 77‑83. 10.1016/j.actpsy.2016.11.006 27940025

[29] Giorgia C., Valentina S., Francesco De Pasquale, Massimiliano S., & Fini C. (2020). *Functional autonomy affects elderly spatial perception in body-centered coordinates*. 20 10.1155/2020/5694790 PMC7053486 32148961

[30] Levine J., Chan K., & Satterfield T. (2015). From rational actor to efficient complexity manager: Exorcising the ghost of Homo economicus with a unified synthesis of cognition research. *Ecological Economics*, 114, 22‑32. 10.1016/j.ecolecon.2015.03.010

[31] Moon K., & Blackman D. (2014). A guide to understanding social science research for natural scientists. *Conservation Biology*: *The Journal of the Society for Conservation Biology*, 28(5), 1167‑1177. 10.1111/cobi.12326 24962114

[32] Munhall P. L. (2008). Perception. In *The SAGE Encyclopedia of Qualitative Research Methods* (p. 607‑607). SAGE Publications, Inc 10.4135/9781412963909

[33] Satterfield T., Kandlikar M., Beaudrie C., Conti J., & Harthorn B. (2009). Anticipating the Perceived Risk of Nanotechnologies. *Nature nanotechnology*, 4, 883 10.1038/nnano.2009.369 19893527

[34] Slovic P. (2000). *The perception of risk*. Earthscan Publications.

[35] Kaplan, S. (1976). Adaptation, structure, and knowledge. In *Environmental Knowing* (p. 32‑45). G. T. Moore and R. G. Golledge.

[36] Hodgson R. W., & Thayer R. L. (1980). Implied human influence reduces landscape beauty. *Landscape Planning*, 7(2), 171‑179. 10.1016/0304-3924(80)90014-3

[37] Dewey J. (1948). On the Aesthetics of Dewey. *The Journal of Aesthetics and Art Criticism*, 6(3), 203‑207. JSTOR. 10.2307/426476

[38] Daniel T. C. (1990). Measuring the quality of the natural environment: A psychophysical approach. *American Psychologist*, 45(5), 633‑637. 10.1037/0003-066X.45.5.633

[39] Berleant A. (1992). *The Aesthetics of Environment*. Temple University Press.

[40] Brown T., C., & Daniel T. C. (1987). *Context effects in perceived environmental quality assessment*: *Scene selection and landscape quality ratings*. 7, 233‑250.

[41] Daniel T. C., & Vining J. (1983). Methodological Issues in the Assessment of Landscape Quality. In *Behaviour and the Natural Environment* (Plenum Press, p. Chapter 2, 39–83). Altman I. and Wohwil J.

[42] Kaplan R. (1975). Some methods and strategies in the prediction of preference. In *Landscape assessment*: *Valeurs*, *perceptions and resources*. (Stroudsburg, PA: Dowden, Hutchinson and Ross, p. 118‑129). Zube E. H., Brush R. O.and Fabos J. G.

[43] Hull R. B., & Reveli G. R. B. (1989). Cross-cultural comparison of landscape scenic beauty evaluations: A case study in Bali. *Journal of Environmental Psychology*, 9(3), 177‑191. 10.1016/S0272-4944(89)80033-7

[44] Purcell A. T., Lamb R. J., Mainardi Peron E., & Falchero S. (1994). Preference or preferences for landscape? *Journal of Environmental Psychology*, 14(3), 195‑209. 10.1016/S0272-4944(94)80056-1

[45] Pitt D. G., & Zube E. H. (1987). Management of natural environments. In *Handbook of Environmental Psychology* (p. 1009‑1051). Stokols D.& Altman I.

[46] Ulrich R., Simons R., Losito B., Fiorito E., Miles M., & Zelson M. (1991). Stress Recovery During Exposure to Natural and Urban Environments. Journal of Environmental Psychology. 11: 201–230. Journal of Environmental Psychology, 11, 201‑230. 10.1016/S0272-4944(05)80184-7

