# Supplementary material for: Evoked pleasure and approach-avoidance in response to pollution
Source: PLoS One. 2020 Jun 25;15(6):e0234210. doi: 10.1371/journal.pone.0234210 (PMC7316332; doi:10.1371/journal.pone.0234210)
Supplement: S1 Table — (DOCX) [file pone.0234210.s001.docx]

| ***Tests*** | ***F*** | ***p*** | ***Size Effect*** | |  |
| --- | --- | --- | --- | --- | --- |
| ***GLOBAL EFFECT*** |  |  |  |  |  |
| *Global pleasure* | *667,244* | *1,41E-30* | *-2,49* | *large* | *(d)* |
| *Global approach desire* | *120,79* | *7,99E-30* | *-1,37* | *large* | *(d)* |
| ***COMPLEMENTARY EFFECTS*** | *Scenes* |  |  |  |  |
|  | ***Pleasure*** |  |  |  |  |
| *Condition(Clean/Polluted)* | *663,52* | *1,60E-30* | *-2,49* | *large* | *(d)* |
| *Feature(Urban/Rural)* | *92,24* | *6,14E-13* | *0,23* | *small* | *(d)* |
| *Presence of individuals (Individuals)* | *4,85* | *3,22E-02* | *-0,003* | *negligeable* | *(d)* |
| *Condition:Feature* | *304,31* | *6,63E-23* | *3,01E-01* | *large* | *(ges)* |
|  | ***Approach desire*** | |  |  |  |
| *Condition(Clean/Polluted)* | *115,81* | *1,67E-14* | *-1,37* | *large* | *(d)* |
| *Feature(Urban/Rural)* | *110,55* | *3,71E-14* | *0,35* | *small* | *(d)* |
| *Presence of individuals (Individuals)* | *11,86* | *1,18E-03* | *0,008* | *negligeable* | *(d)* |
| *Condition:Feature* | *186,14* | *2,60E-18* | *1,19E-01* | *large* | *(ges)* |
| ***COMPLEMENTARY EFFECTS*** | *Subjects* |  |  |  |  |
|  | ***Pleasure*** |  |  |  |  |
| *Condition(Clean/Polluted)* | *9348,42* | *< 2E-16* | *-2,49* | *large* | *(d)* |
| *Condition:Gender* | *34,99* | *3,50E-09* | *0,006* | *small* | *(ges)* |
| *Condition:Age* | *34,07* | *1,94E-15* | *0,01* | *negligeable* | *(ges)* |
| *Condition:Gender:Age* | *6,39* | *0,002* | *0,002* | *negligeable* | *(ges)* |
|  | ***Approach desire*** |  |  |  |  |
| *Condition(Clean/Polluted)* | *272,72* | *< 2E-16* | *-1,37* | *large* | *(d)* |
| *Gender* | *5,74* | *0,02* | *-0,05* | *negligeable* | *(d)* |
| *Age Categories (Age)* | *42,82* | *< 2E-16* | *0,01* | *negligeable* | *(ges)* |
| *Condition:Gender* | *10,31* | *0,001* | *0,002* | *negligeable* | *(ges)* |
| *Condition:Age* | *77,91* | *< 2E-16* | *0,03* | *negligeable* | *(ges)* |
| *Gender:Age* | *14,57* | *4,90E-07* | *0,005* | *negligeable* | *(ges)* |
| *Condition:Gender:Age* | *17,45* | *2,78E-08* | *0,006* | *negligeable* | *(ges)* |
