# Supplementary figures and images for: Evoked pleasure and approach-avoidance in response to pollution (part 2 of 2)
Source: PLoS One. 2020 Jun 25;15(6):e0234210. doi: 10.1371/journal.pone.0234210 (PMC7316332; doi:10.1371/journal.pone.0234210)

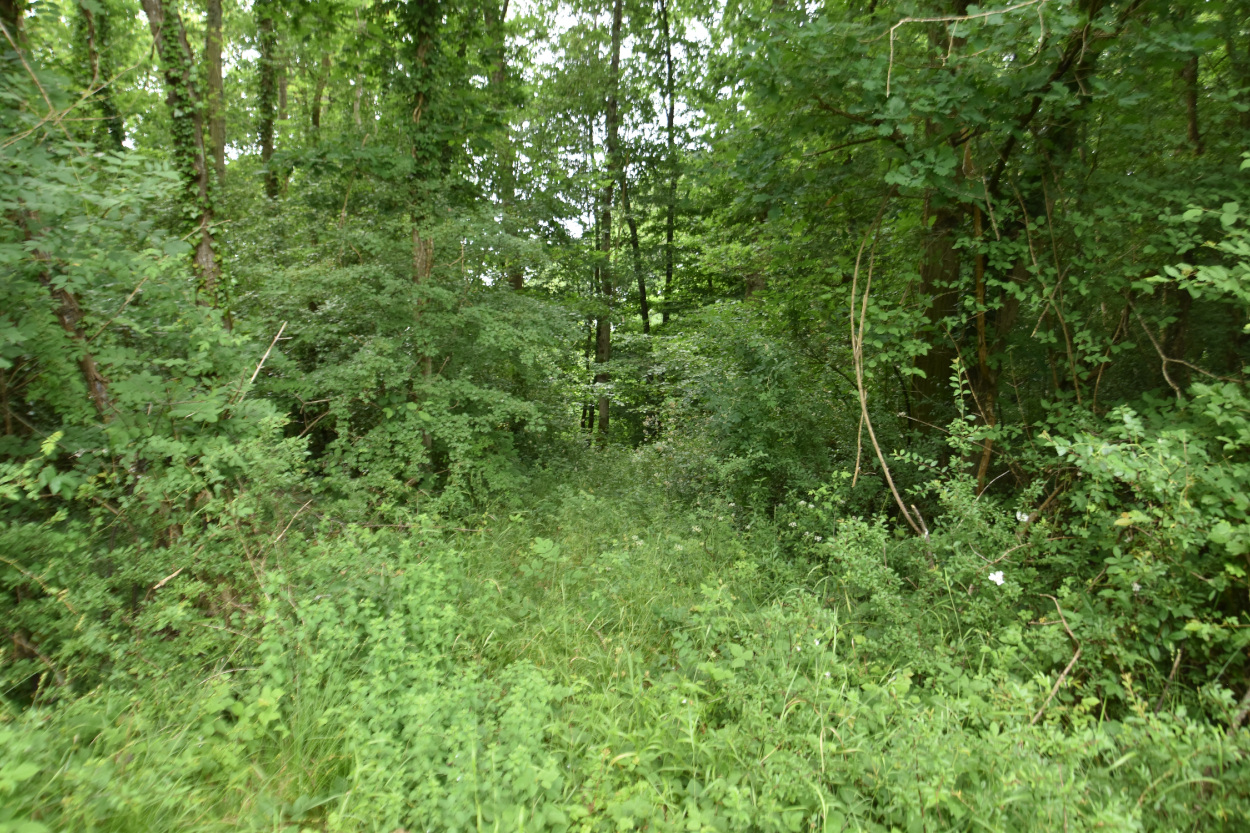

Supplement: S1 Data — (ZIP) [file pone.0234210.s002.zip › Pictures_DataBase_Environment/Clean/Clean - Rural/Clean - Rural - Without Individuals/Rur.PROPRE04.1.JPG]

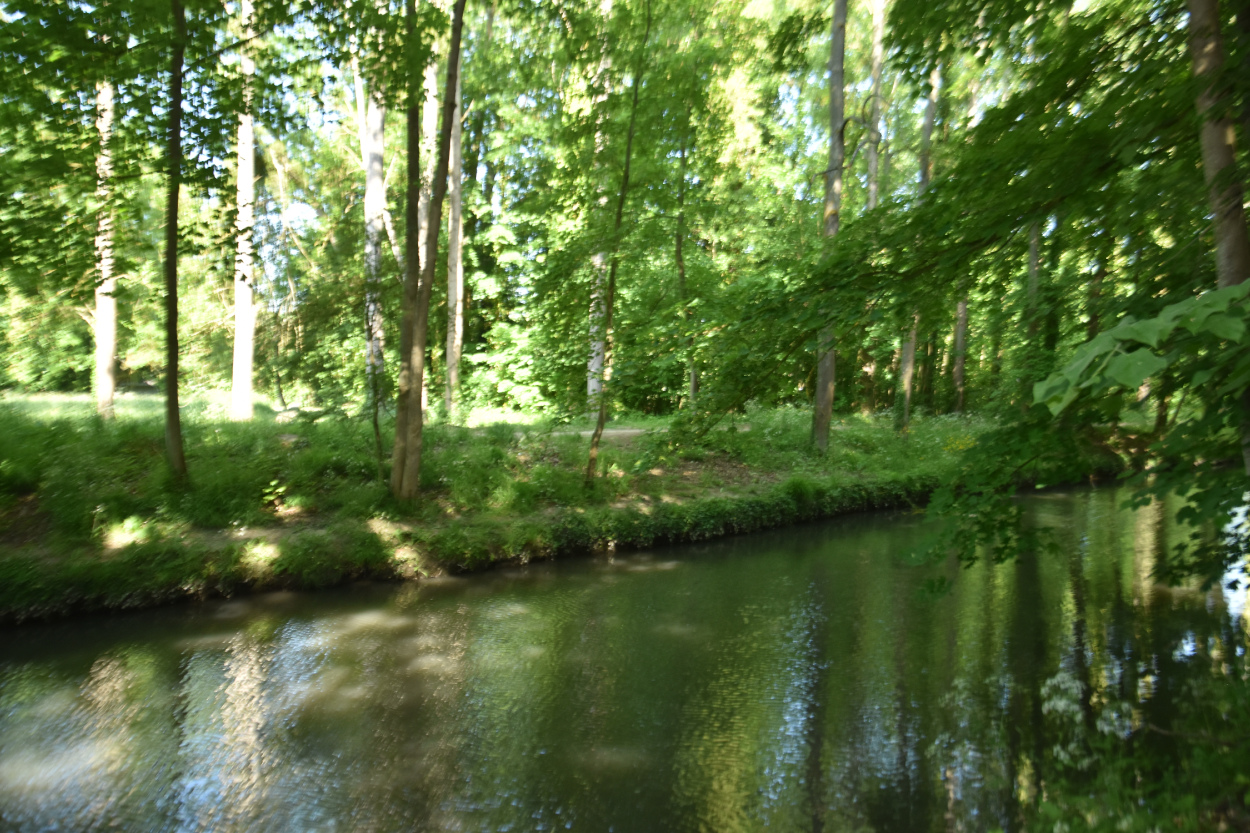

Supplement: S1 Data — (ZIP) [file pone.0234210.s002.zip › Pictures_DataBase_Environment/Clean/Clean - Rural/Clean - Rural - Without Individuals/Rur.PROPRE06.1.JPG]

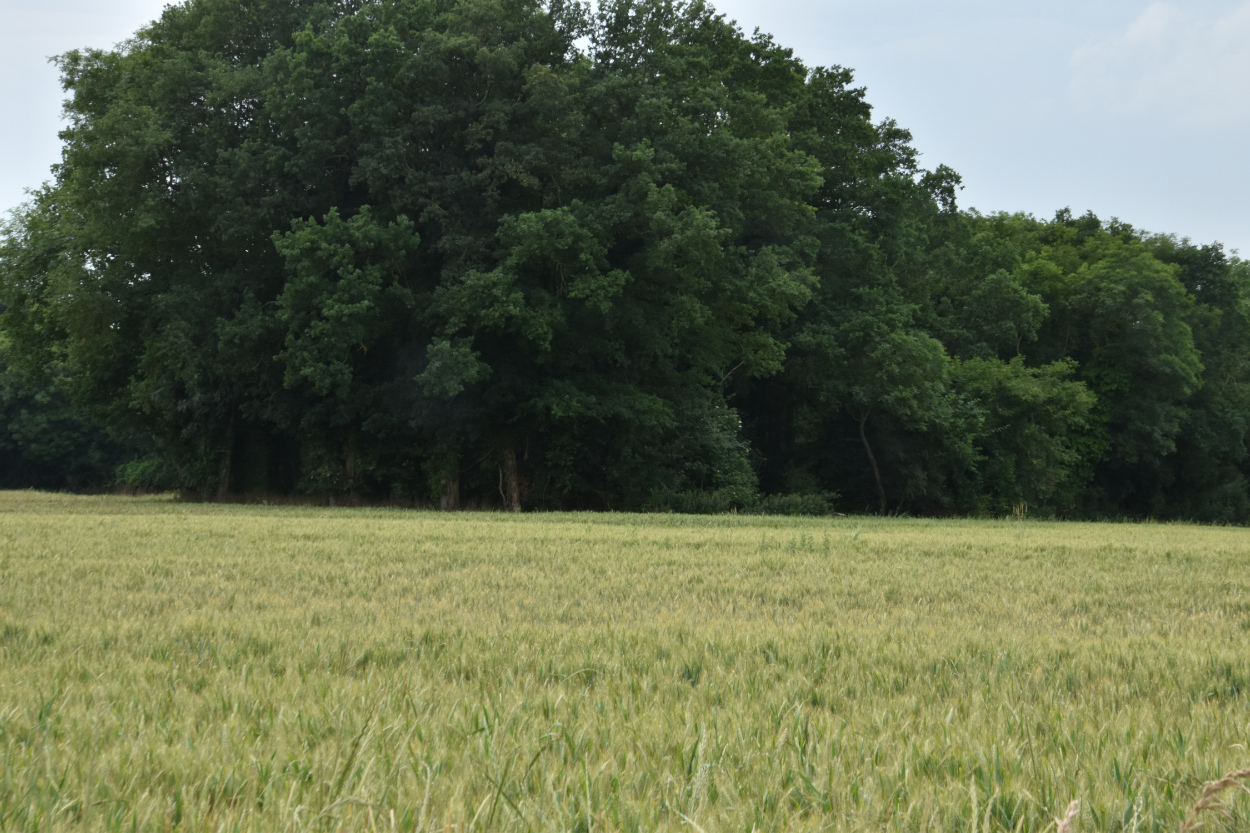

Supplement: S1 Data — (ZIP) [file pone.0234210.s002.zip › Pictures_DataBase_Environment/Clean/Clean - Rural/Clean - Rural - Without Individuals/Rur.PROPRE01.1.JPG]

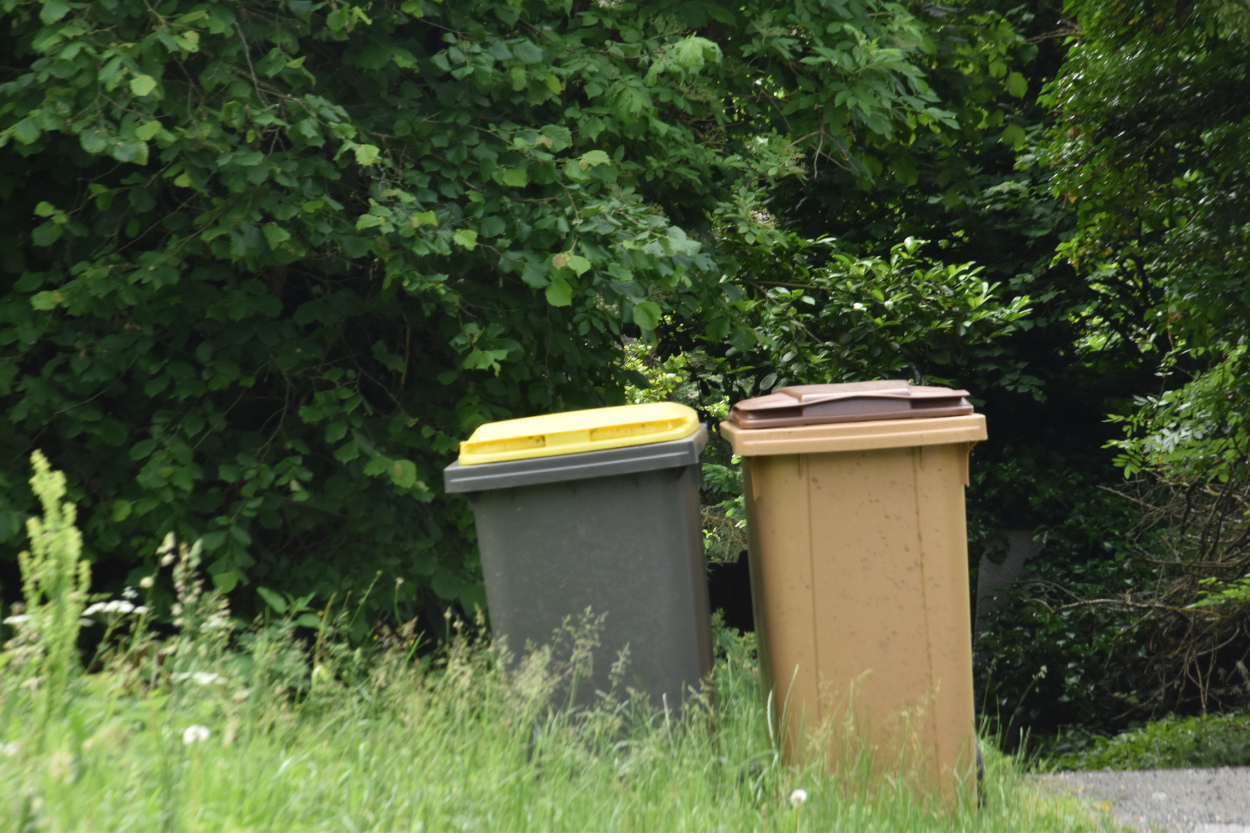

Supplement: S1 Data — (ZIP) [file pone.0234210.s002.zip › Pictures_DataBase_Environment/Clean/Clean - Rural/Clean - Rural - Without Individuals/Rur.PROPRE03.1.JPG]

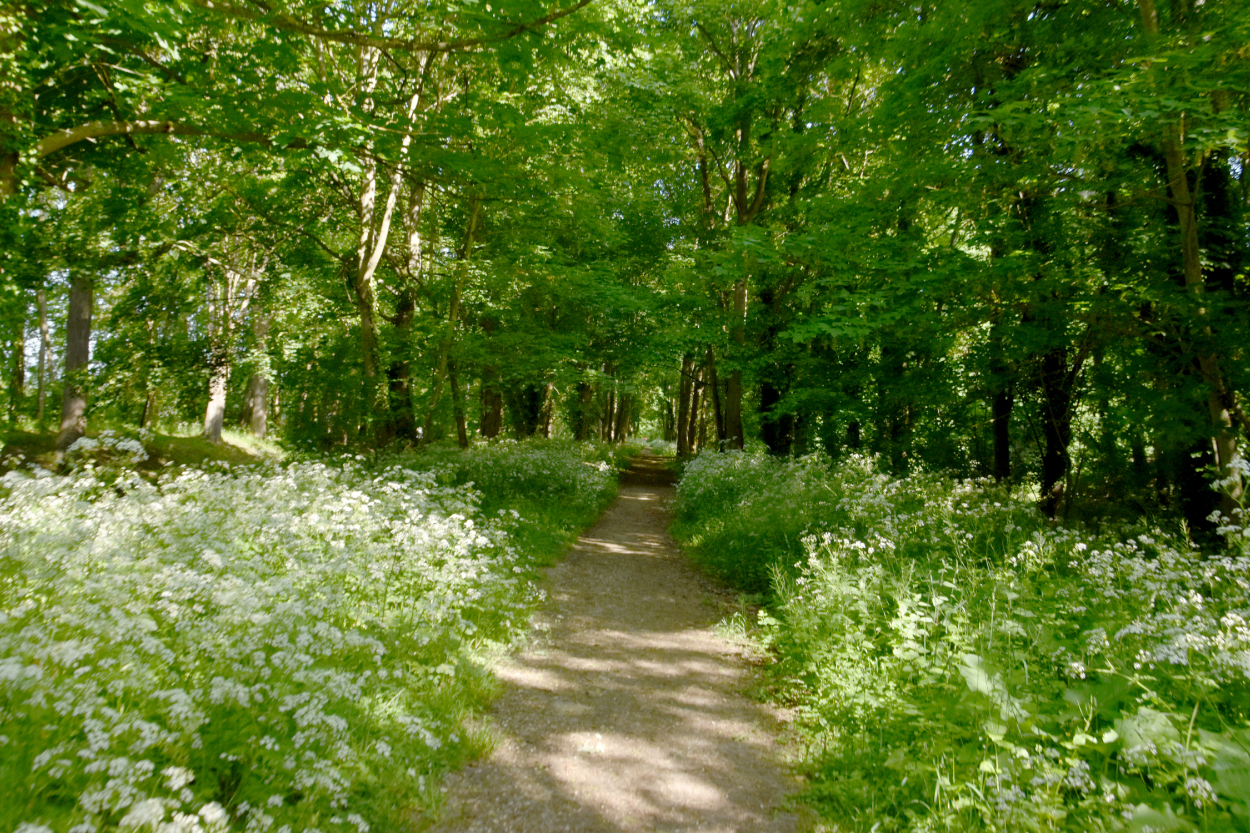

Supplement: S1 Data — (ZIP) [file pone.0234210.s002.zip › Pictures_DataBase_Environment/Clean/Clean - Rural/Clean - Rural - Without Individuals/Rur.PROPRE07.1.JPG]

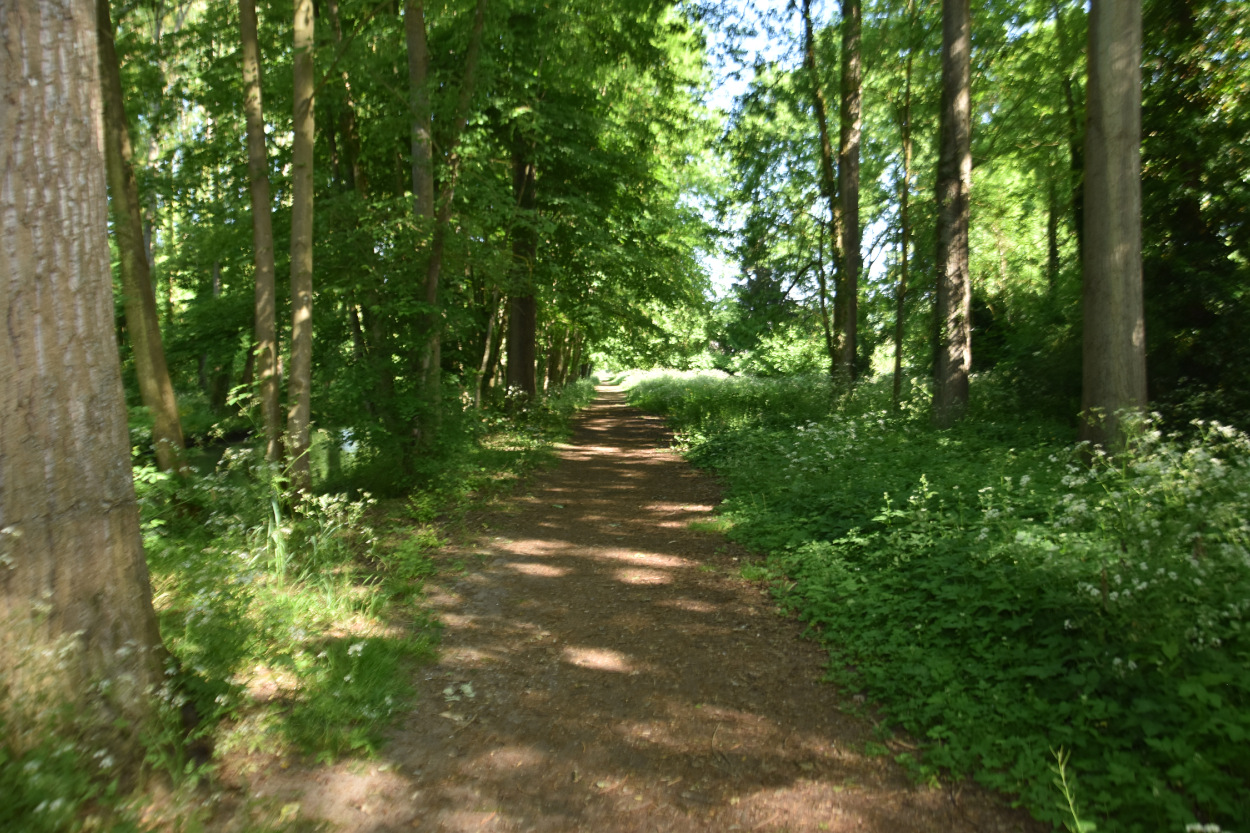

Supplement: S1 Data — (ZIP) [file pone.0234210.s002.zip › Pictures_DataBase_Environment/Clean/Clean - Rural/Clean - Rural - Without Individuals/Rur.PROPRE05.1.JPG]

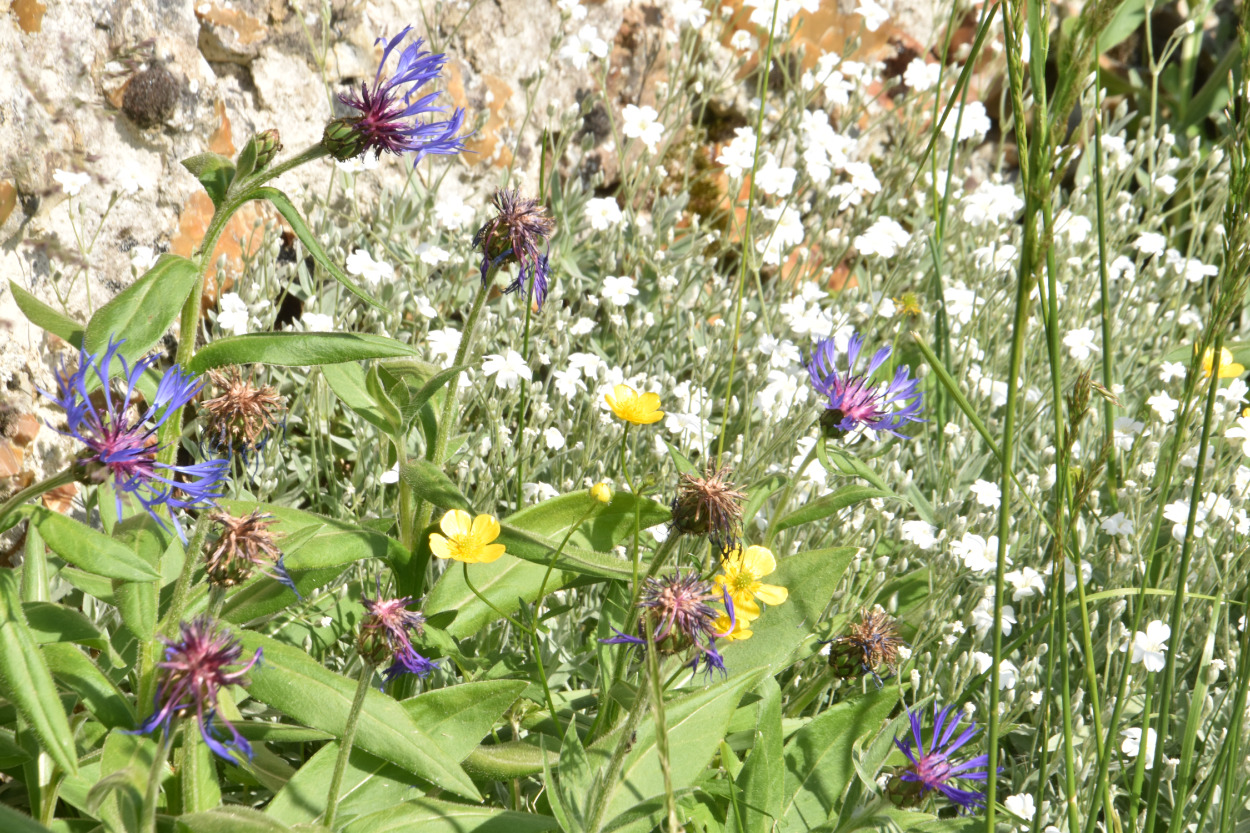

Supplement: S1 Data — (ZIP) [file pone.0234210.s002.zip › Pictures_DataBase_Environment/Clean/Clean - Rural/Clean - Rural - Without Individuals/Rur.PROPRE10.1.JPG]

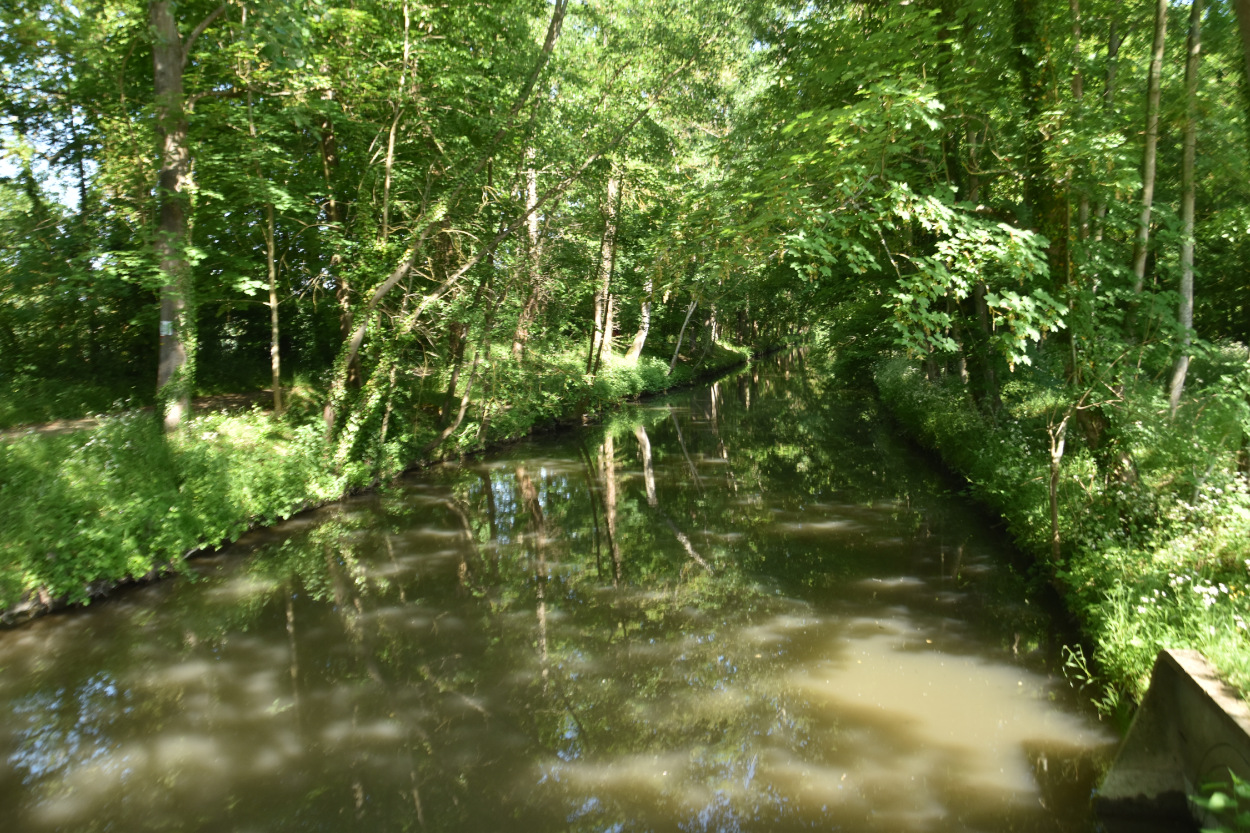

Supplement: S1 Data — (ZIP) [file pone.0234210.s002.zip › Pictures_DataBase_Environment/Clean/Clean - Rural/Clean - Rural - Without Individuals/Rur.PROPRE09.1.JPG]

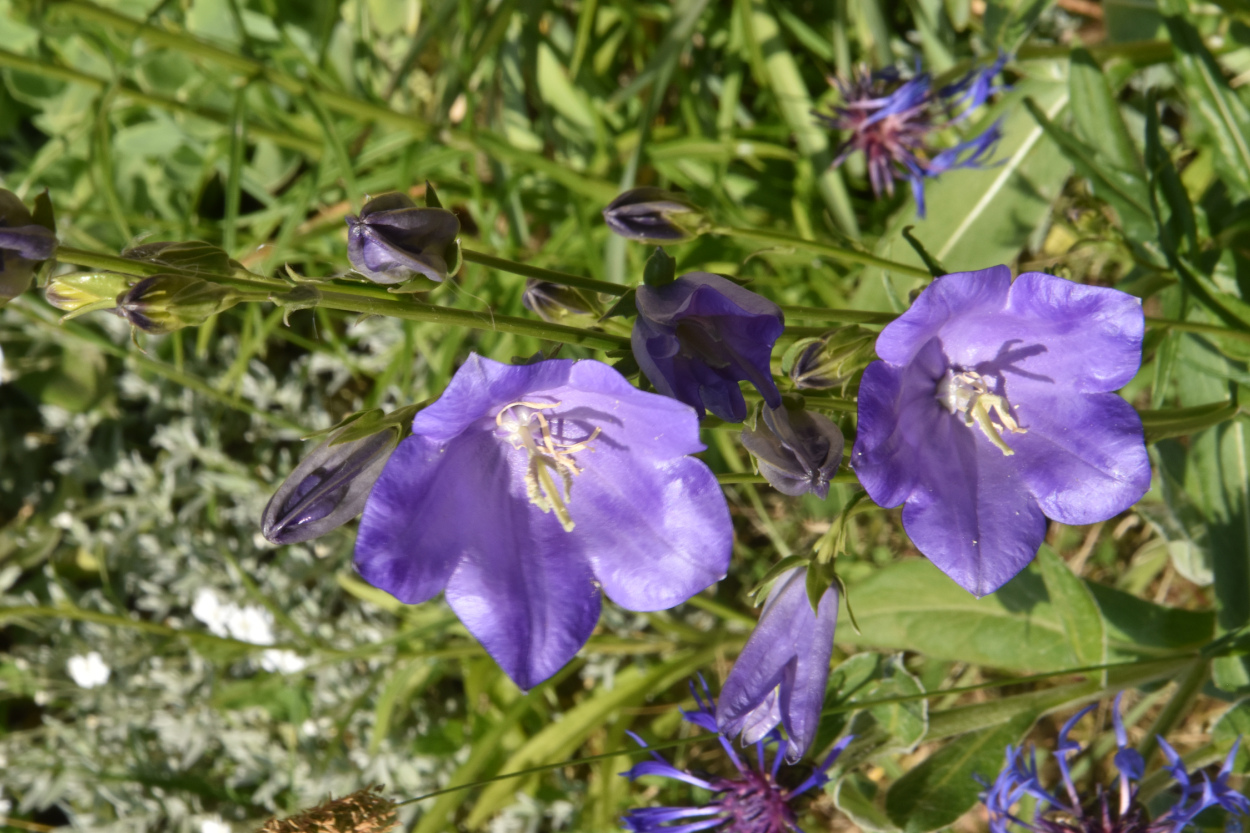

Supplement: S1 Data — (ZIP) [file pone.0234210.s002.zip › Pictures_DataBase_Environment/Clean/Clean - Rural/Clean - Rural - Without Individuals/Rur.PROPRE12.1.JPG]

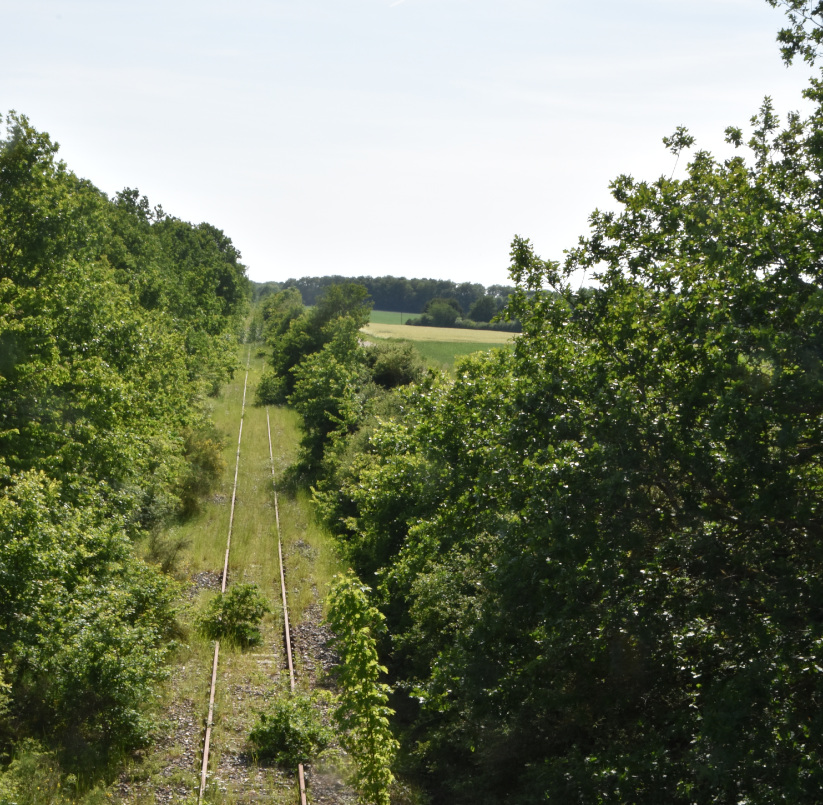

Supplement: S1 Data — (ZIP) [file pone.0234210.s002.zip › Pictures_DataBase_Environment/Clean/Clean - Rural/Clean - Rural - Without Individuals/Rur.PROPRE16.1.JPG]

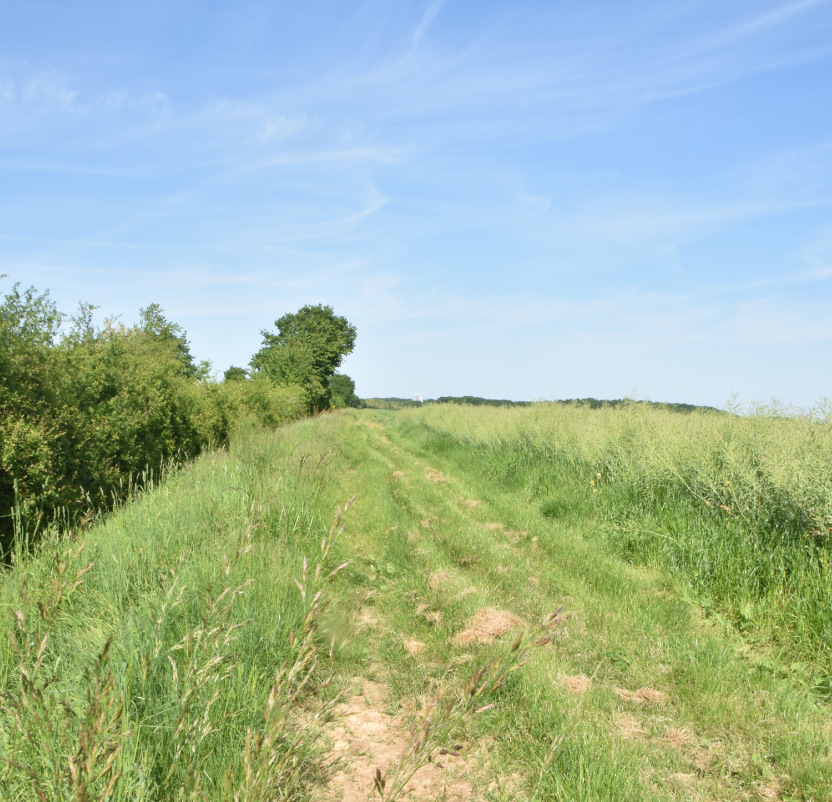

Supplement: S1 Data — (ZIP) [file pone.0234210.s002.zip › Pictures_DataBase_Environment/Clean/Clean - Rural/Clean - Rural - Without Individuals/Rur.PROPRE14.1.JPG]

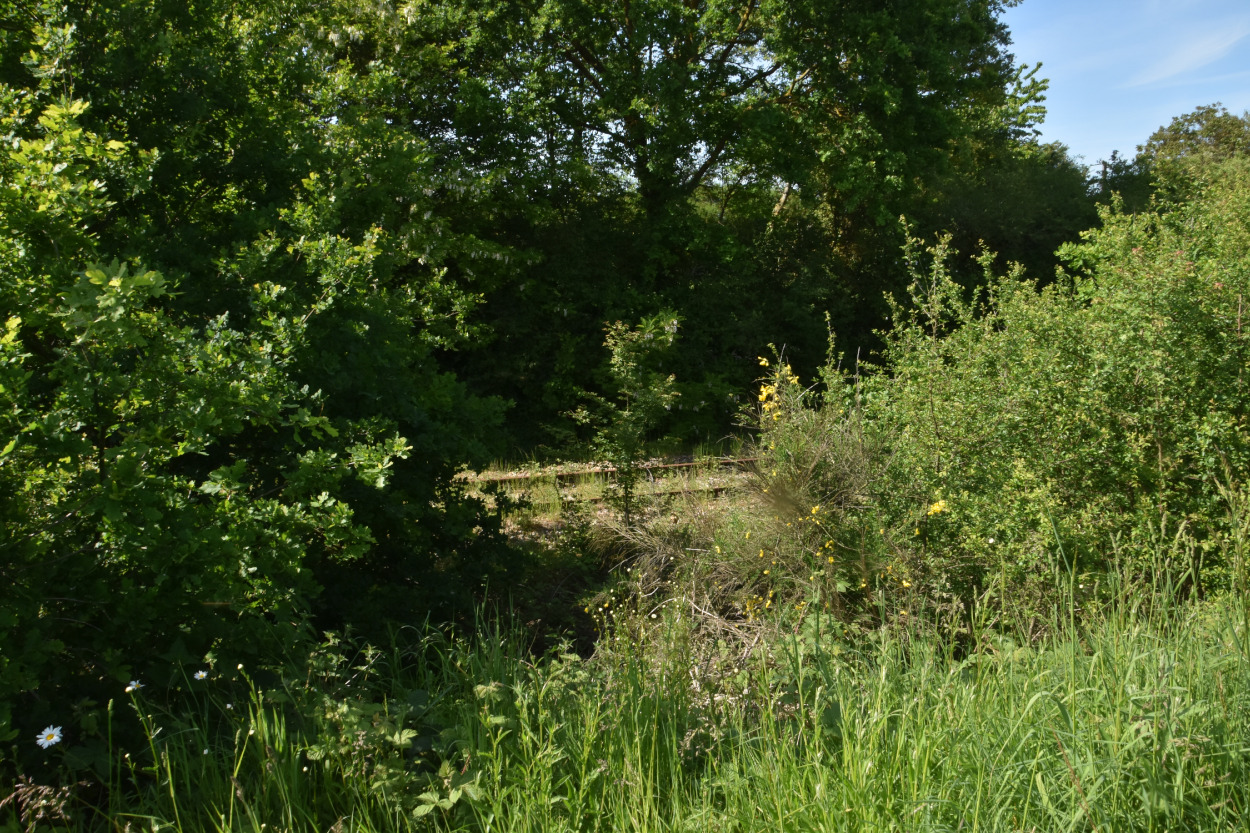

Supplement: S1 Data — (ZIP) [file pone.0234210.s002.zip › Pictures_DataBase_Environment/Clean/Clean - Rural/Clean - Rural - Without Individuals/Rur.PROPRE13.1.JPG]

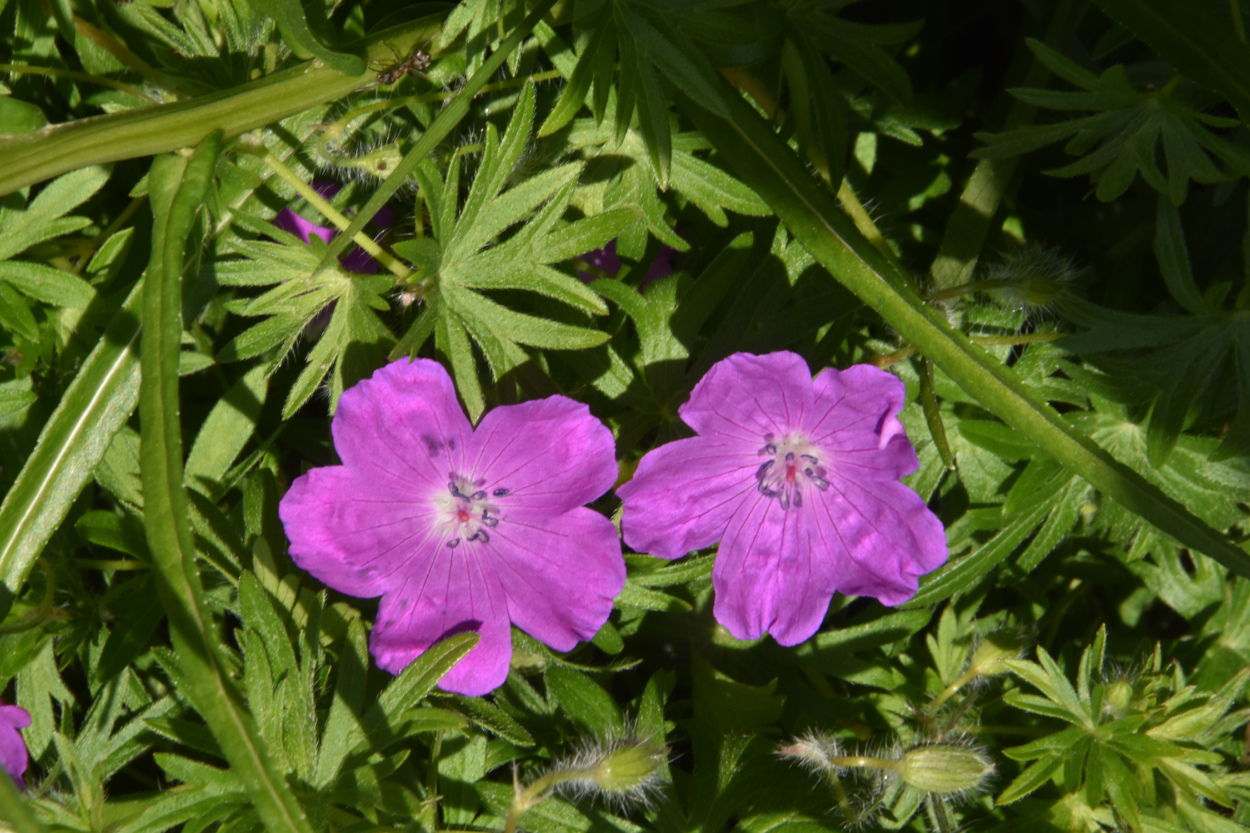

Supplement: S1 Data — (ZIP) [file pone.0234210.s002.zip › Pictures_DataBase_Environment/Clean/Clean - Rural/Clean - Rural - Without Individuals/Rur.PROPRE11.1.JPG]

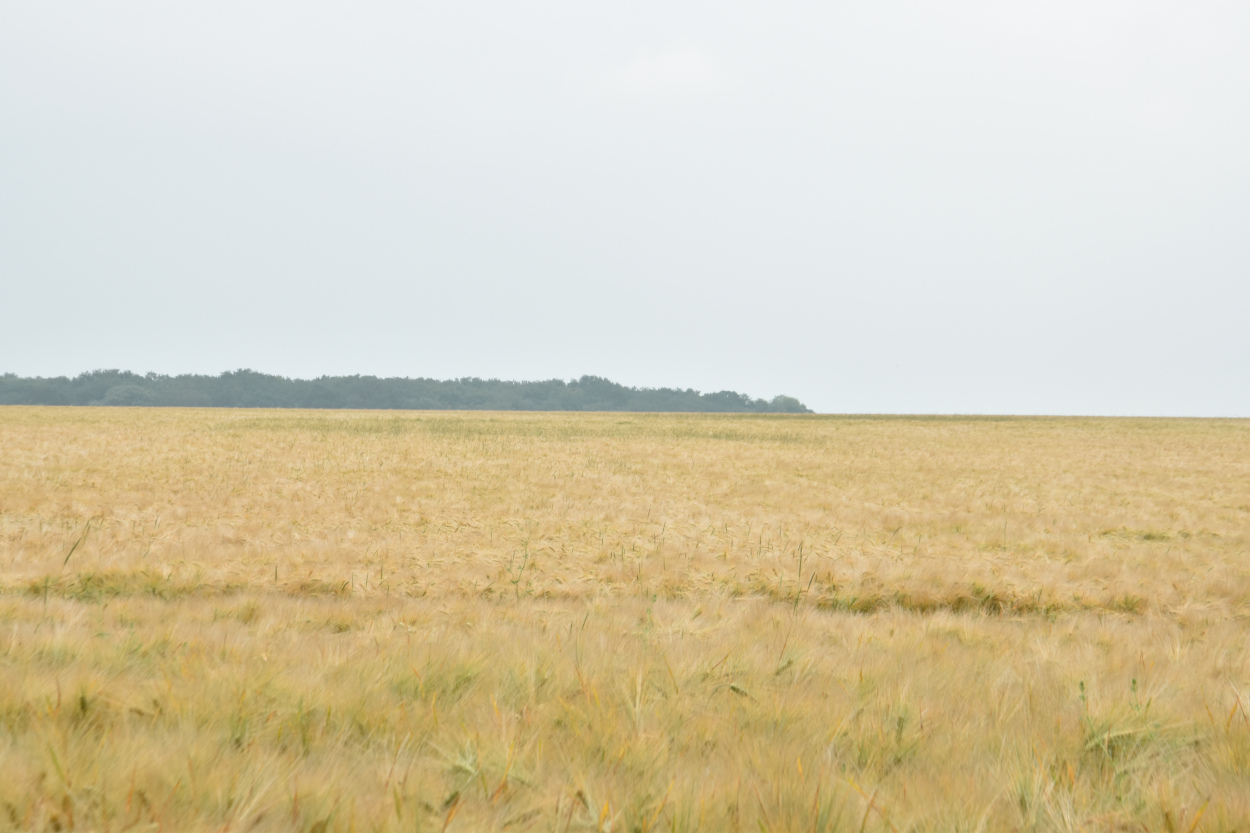

Supplement: S1 Data — (ZIP) [file pone.0234210.s002.zip › Pictures_DataBase_Environment/Clean/Clean - Rural/Clean - Rural - Without Individuals/Rur.PROPRE08.1.JPG]

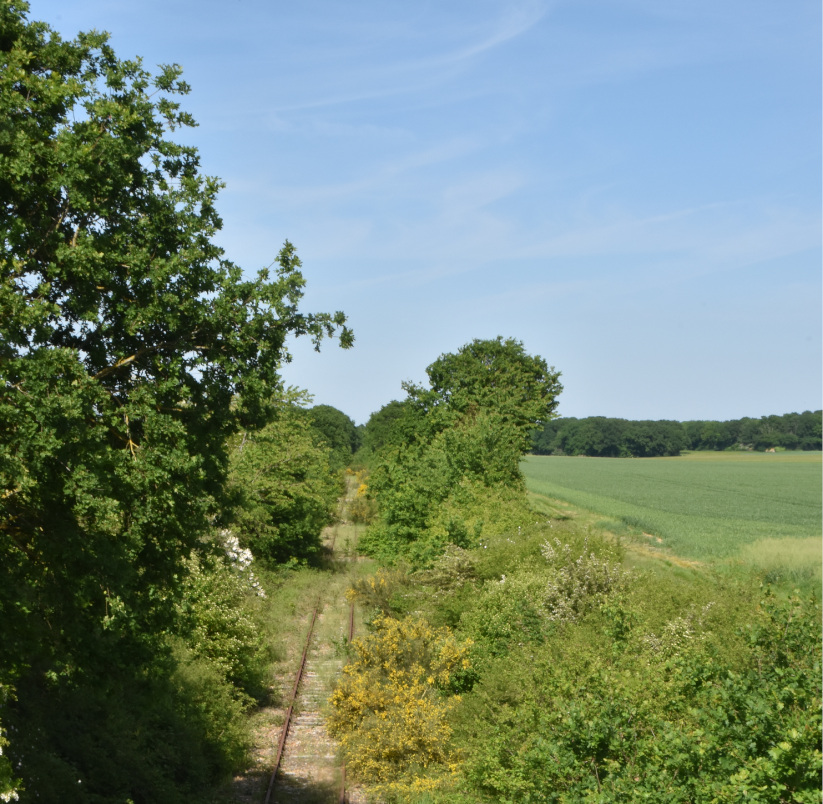

Supplement: S1 Data — (ZIP) [file pone.0234210.s002.zip › Pictures_DataBase_Environment/Clean/Clean - Rural/Clean - Rural - Without Individuals/Rur.PROPRE15.1.JPG]

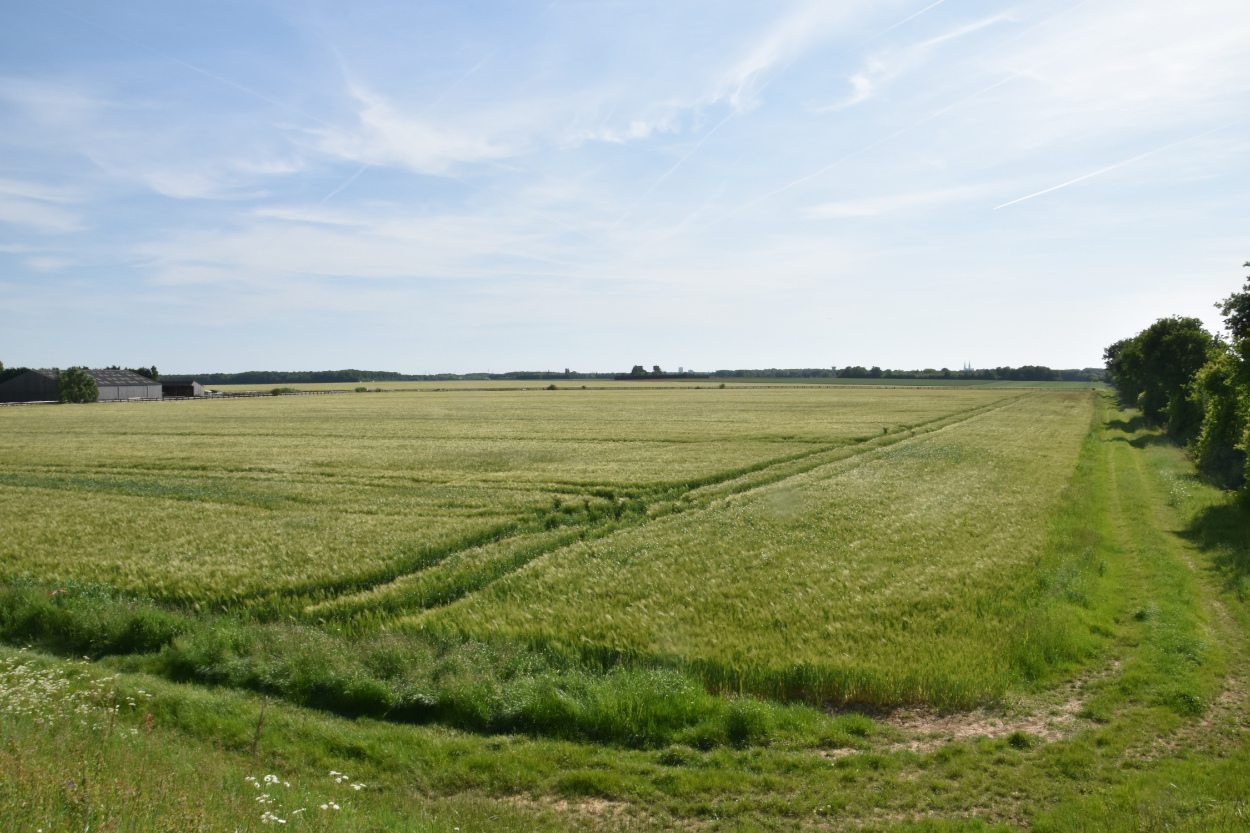

Supplement: S1 Data — (ZIP) [file pone.0234210.s002.zip › Pictures_DataBase_Environment/Clean/Clean - Rural/Clean - Rural - Without Individuals/Rur.PROPRE17.1.JPG]
