# Supplementary figures and images for: Evoked pleasure and approach-avoidance in response to pollution (part 1 of 2)
Source: PLoS One. 2020 Jun 25;15(6):e0234210. doi: 10.1371/journal.pone.0234210 (PMC7316332; doi:10.1371/journal.pone.0234210)

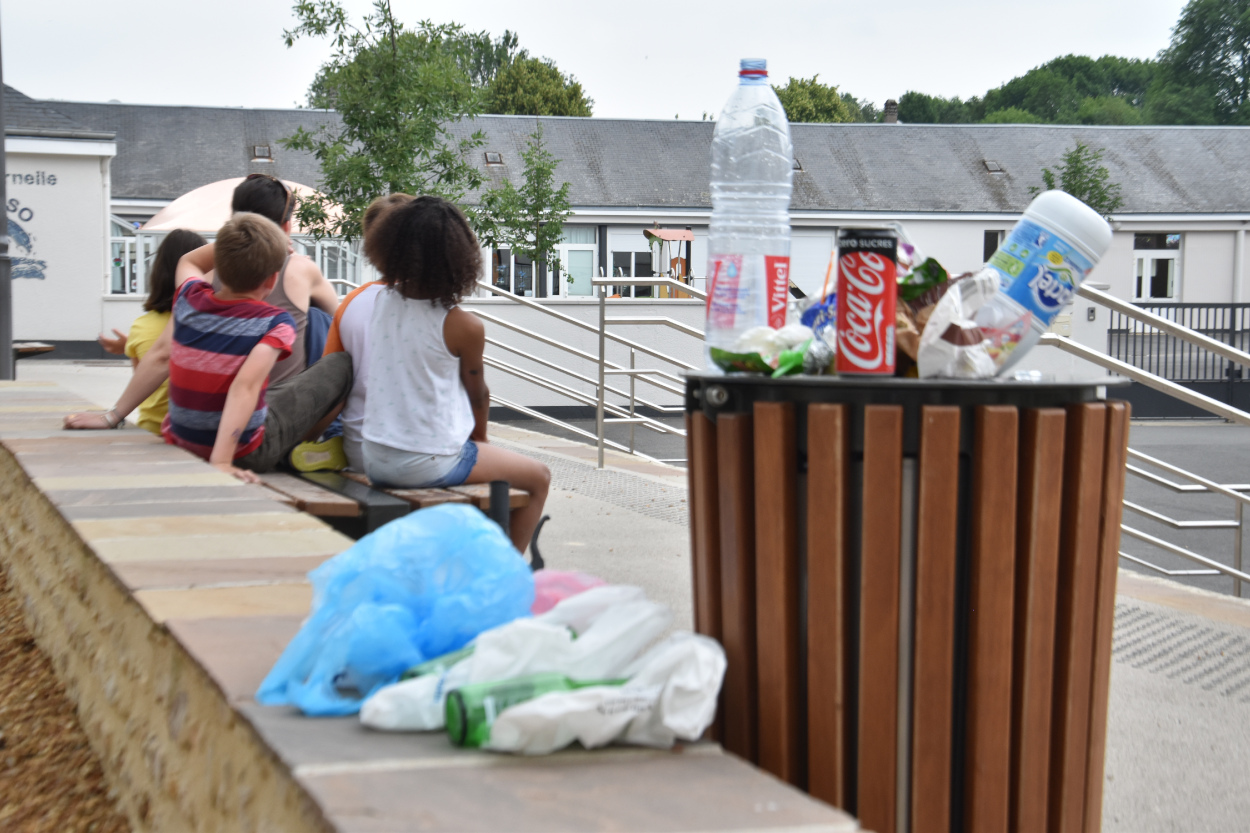

Supplement: S1 Data — (ZIP) [file pone.0234210.s002.zip › Pictures_DataBase_Environment/Polluted/Polluted - Urban/Polluted - Urban - With Individuals/Urb.Pers.SALE02.1.JPG]

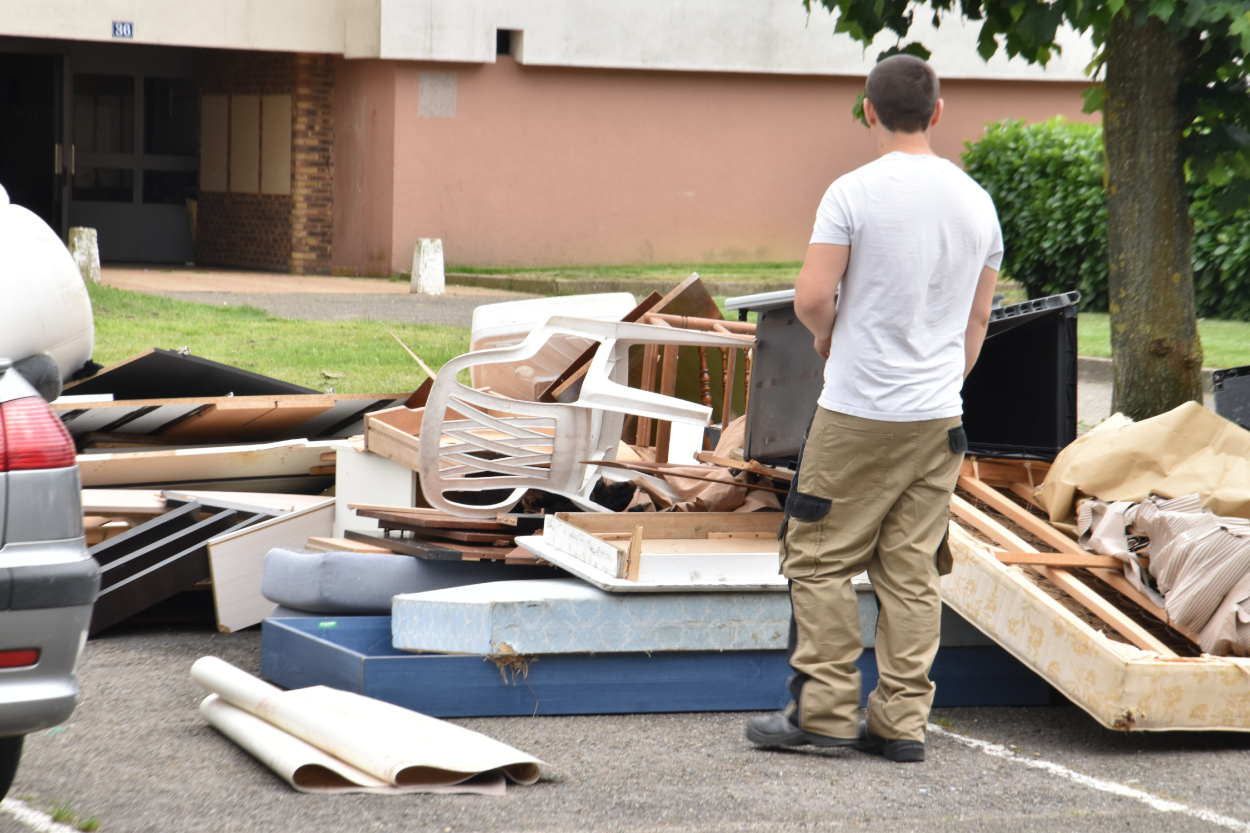

Supplement: S1 Data — (ZIP) [file pone.0234210.s002.zip › Pictures_DataBase_Environment/Polluted/Polluted - Urban/Polluted - Urban - With Individuals/Urb.Pers.SALE04.1.JPG]

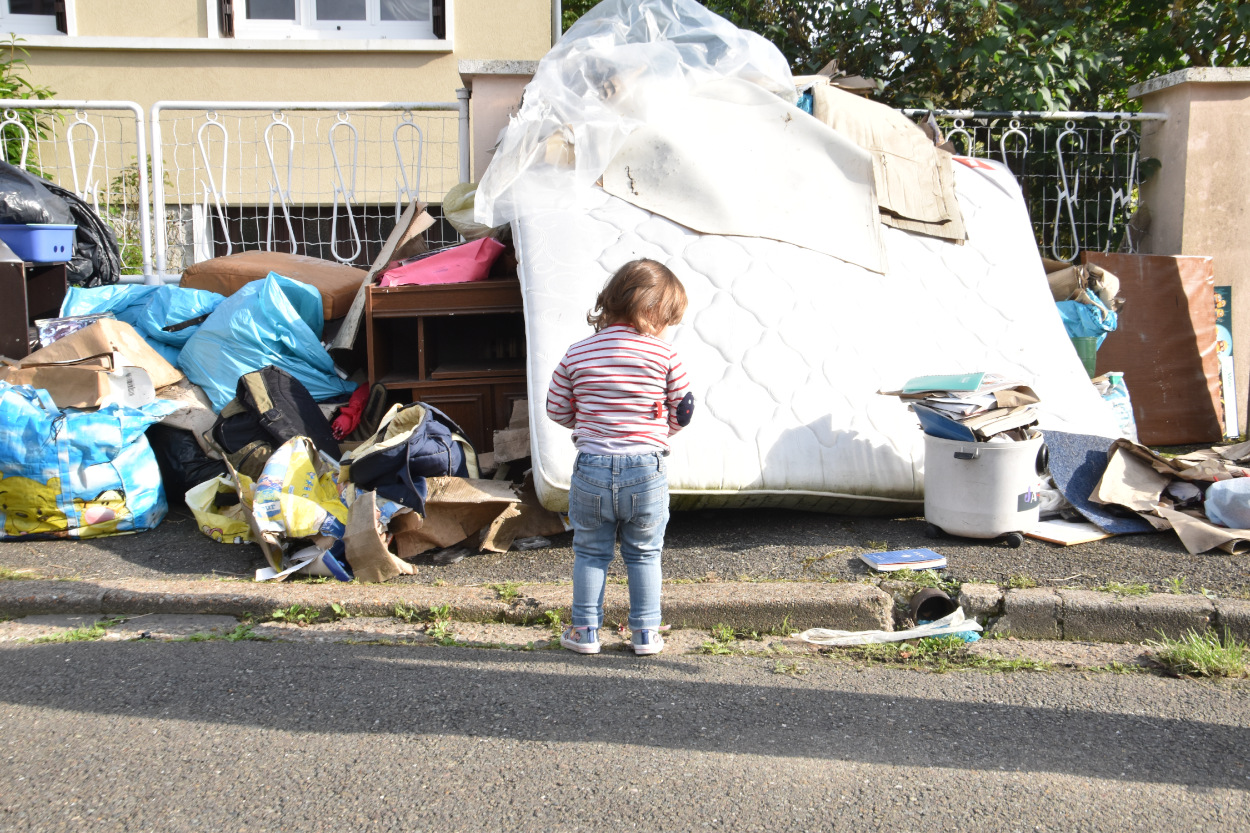

Supplement: S1 Data — (ZIP) [file pone.0234210.s002.zip › Pictures_DataBase_Environment/Polluted/Polluted - Urban/Polluted - Urban - With Individuals/Urb.Pers.SALE06.1.JPG]

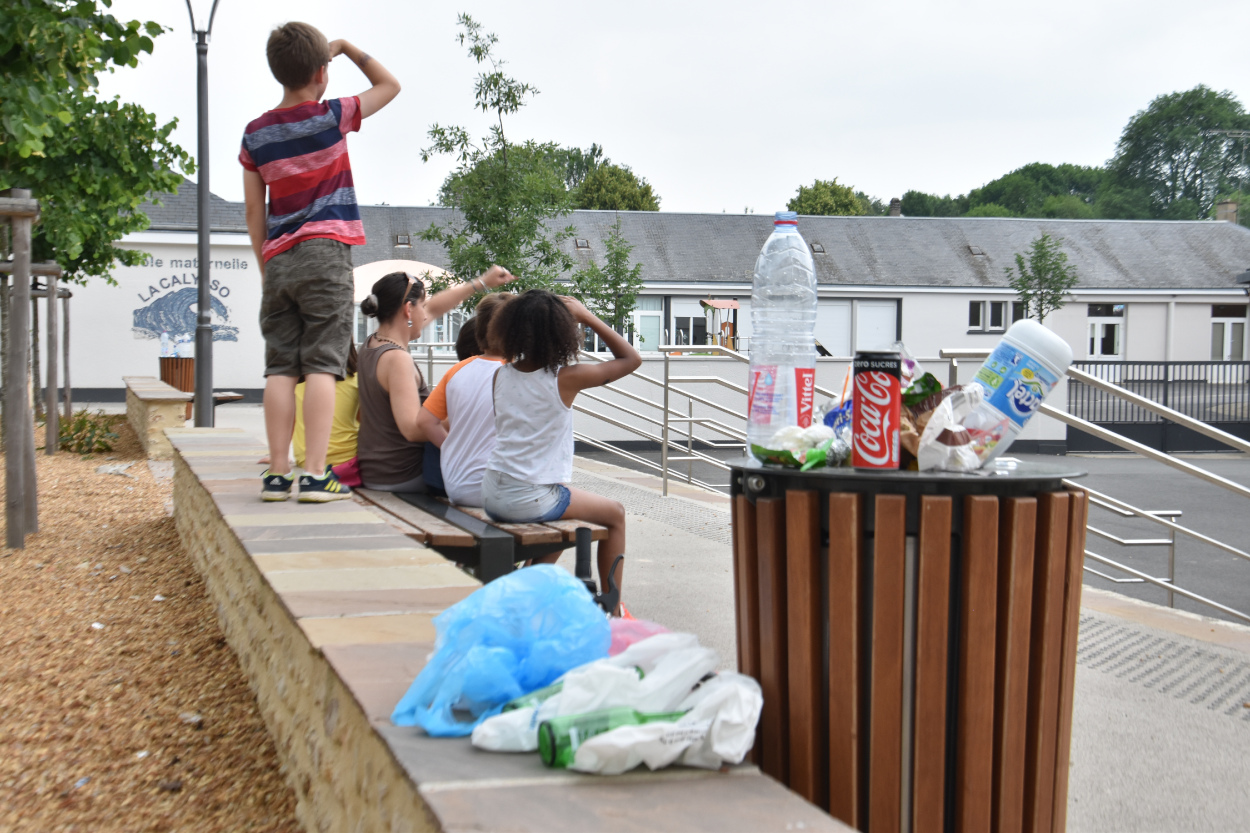

Supplement: S1 Data — (ZIP) [file pone.0234210.s002.zip › Pictures_DataBase_Environment/Polluted/Polluted - Urban/Polluted - Urban - With Individuals/Urb.Pers.SALE01.1.JPG]

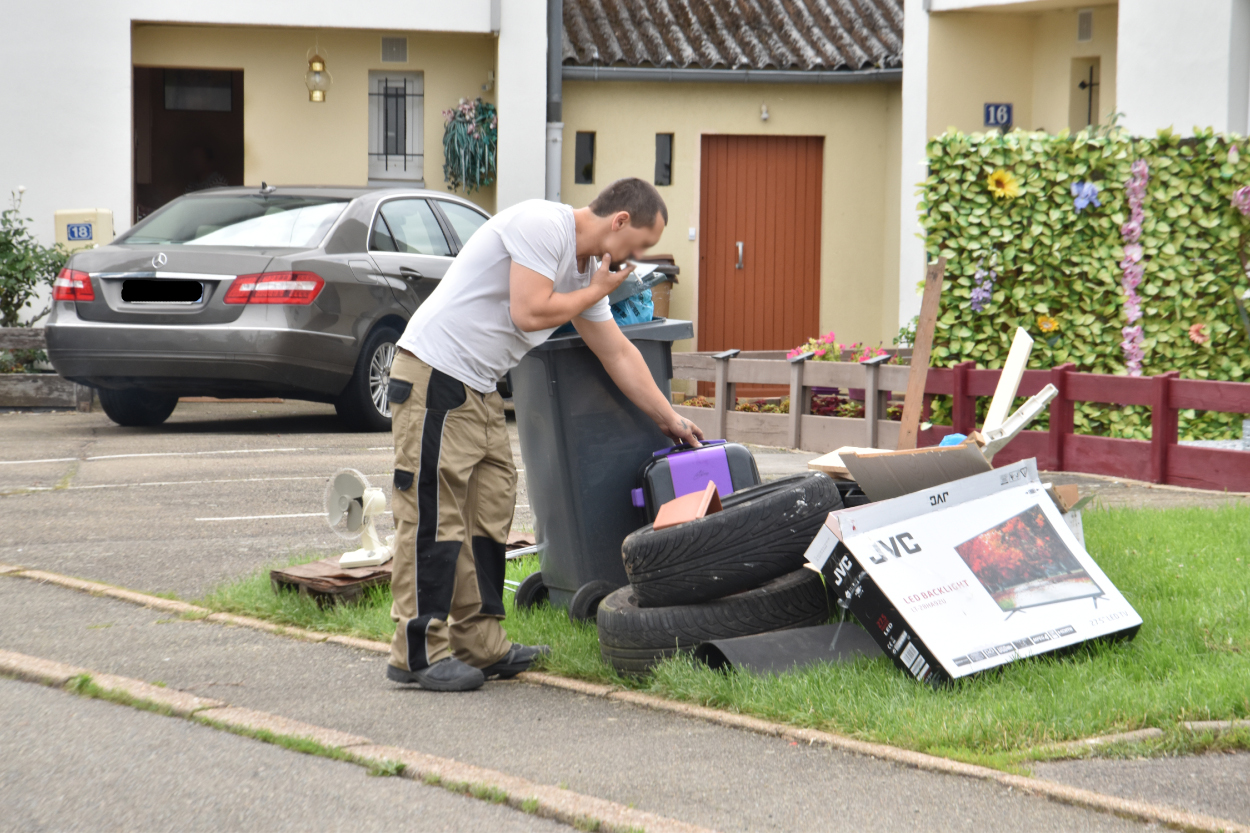

Supplement: S1 Data — (ZIP) [file pone.0234210.s002.zip › Pictures_DataBase_Environment/Polluted/Polluted - Urban/Polluted - Urban - With Individuals/Urb.Pers.SALE03.1.jpg]

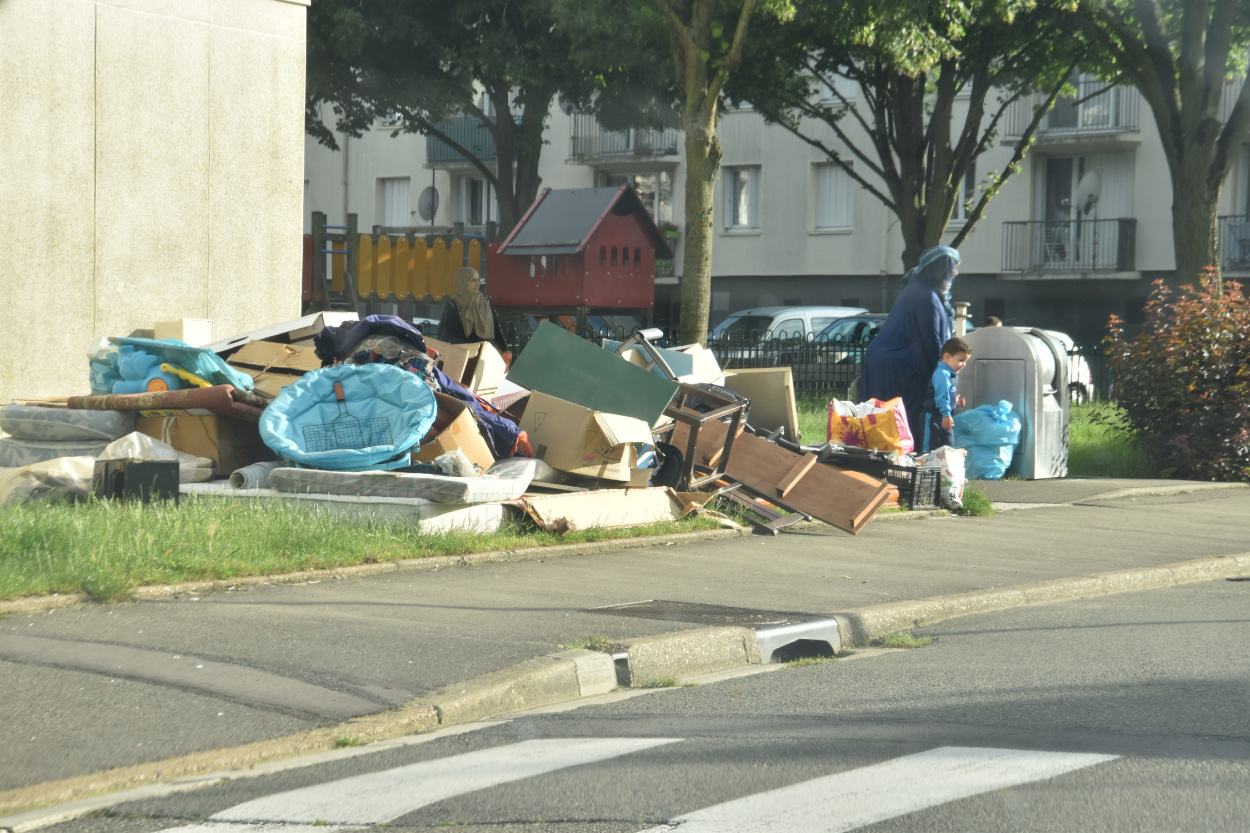

Supplement: S1 Data — (ZIP) [file pone.0234210.s002.zip › Pictures_DataBase_Environment/Polluted/Polluted - Urban/Polluted - Urban - With Individuals/Urb.Pers.SALE07.1.JPG]

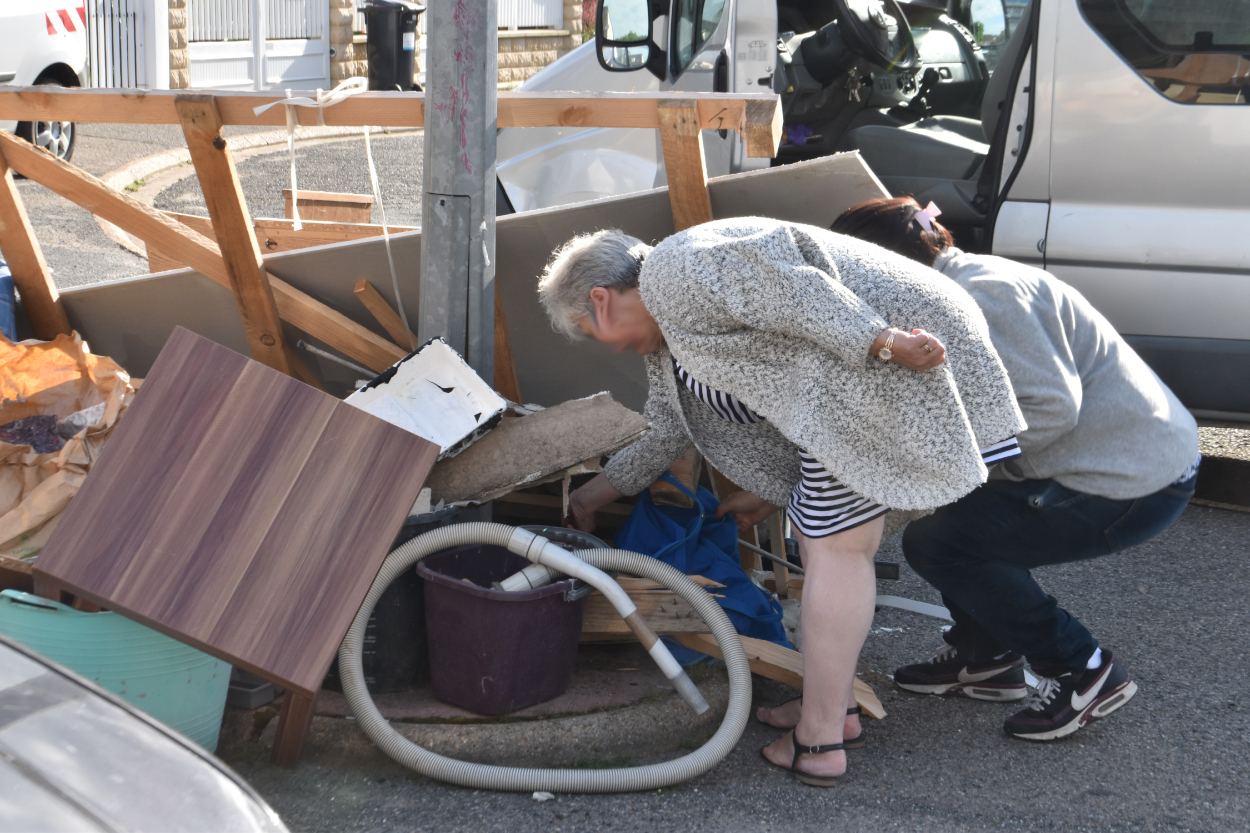

Supplement: S1 Data — (ZIP) [file pone.0234210.s002.zip › Pictures_DataBase_Environment/Polluted/Polluted - Urban/Polluted - Urban - With Individuals/Urb.Pers.SALE05.1.jpg]

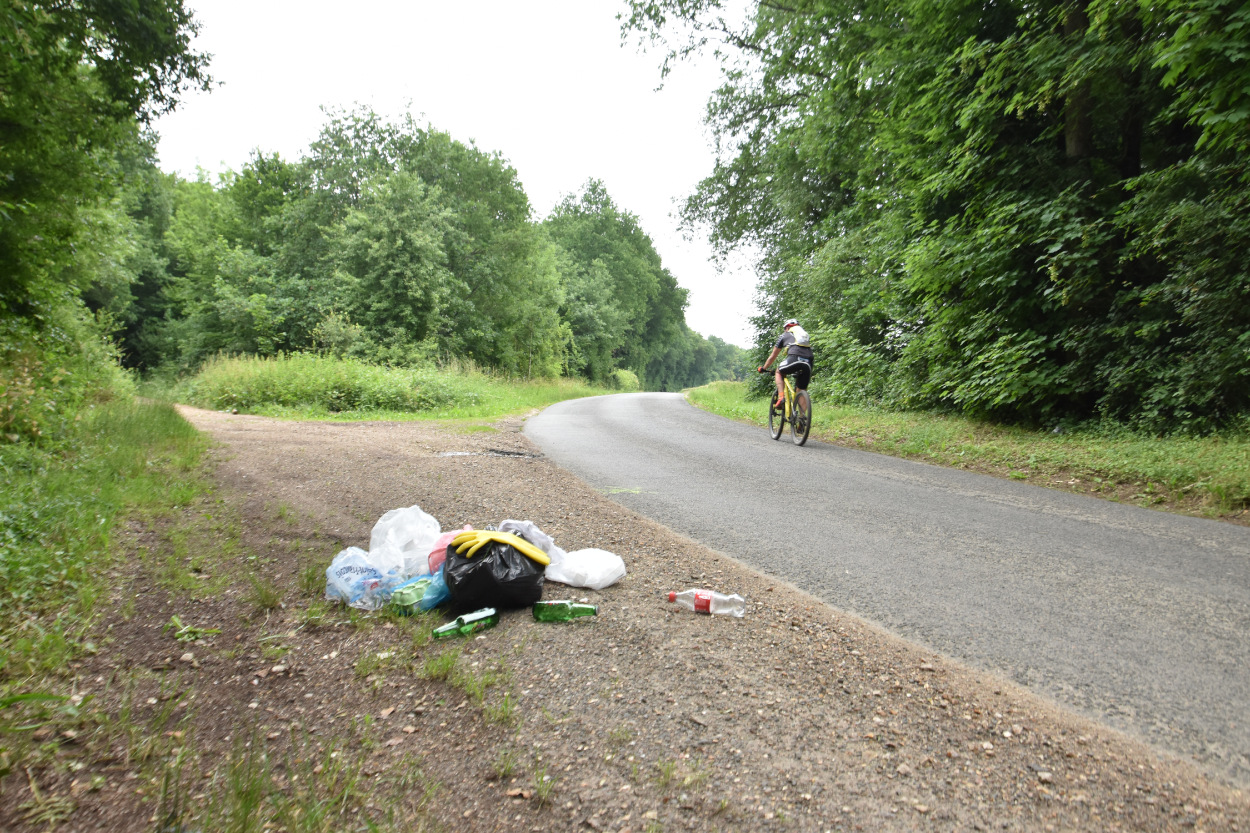

Supplement: S1 Data — (ZIP) [file pone.0234210.s002.zip › Pictures_DataBase_Environment/Polluted/Polluted - Urban/Polluted - Urban - With Individuals/Urb.Pers.SALE08.1.JPG]

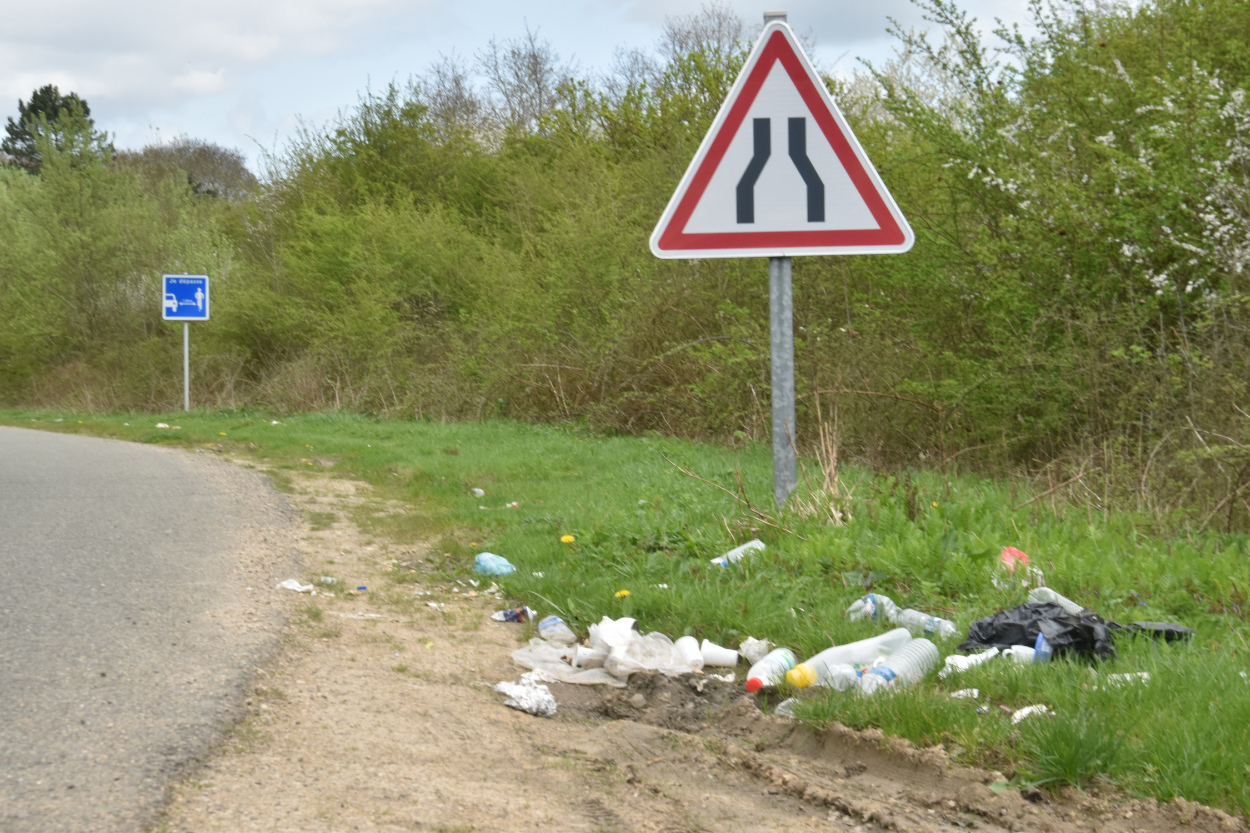

Supplement: S1 Data — (ZIP) [file pone.0234210.s002.zip › Pictures_DataBase_Environment/Polluted/Polluted - Urban/Polluted - Urban - Without Individuals/Urb.SALE24.1.JPG]

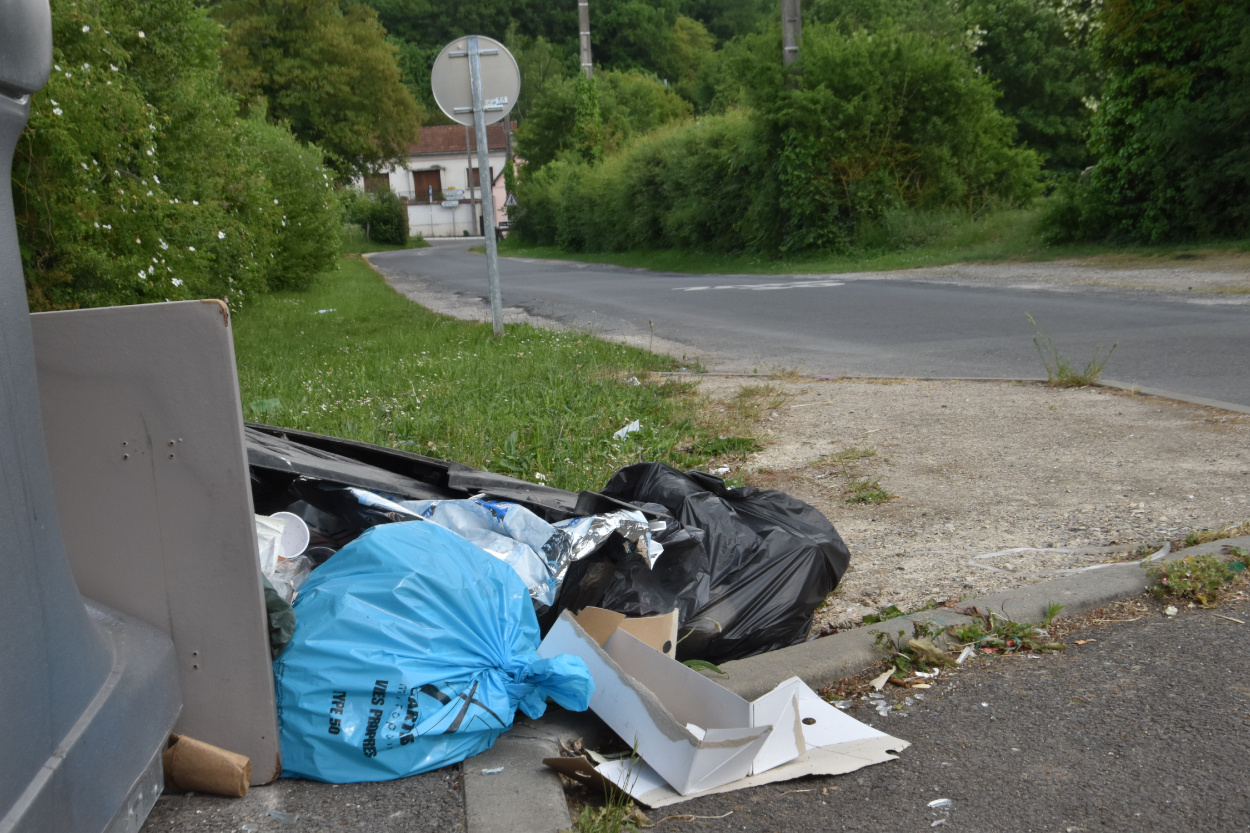

Supplement: S1 Data — (ZIP) [file pone.0234210.s002.zip › Pictures_DataBase_Environment/Polluted/Polluted - Urban/Polluted - Urban - Without Individuals/Urb.SALE19.1.JPG]

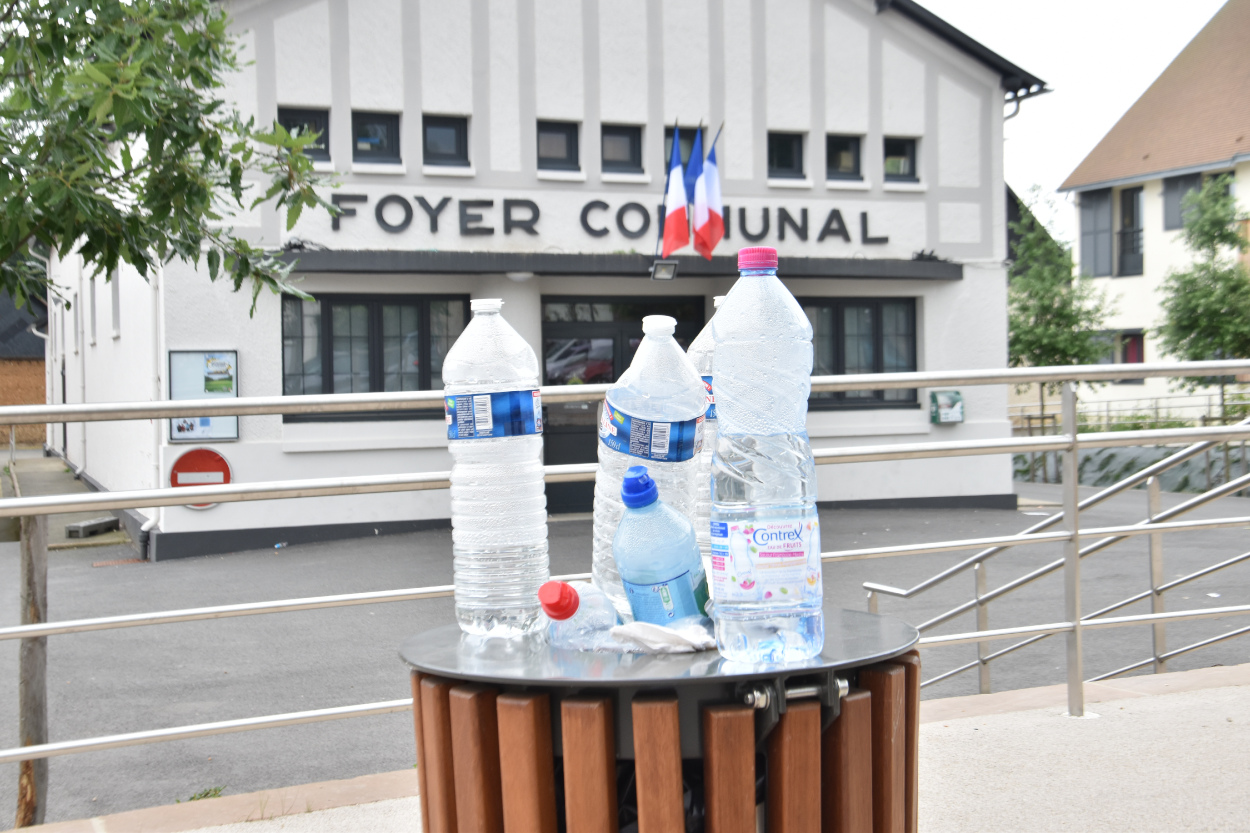

Supplement: S1 Data — (ZIP) [file pone.0234210.s002.zip › Pictures_DataBase_Environment/Polluted/Polluted - Urban/Polluted - Urban - Without Individuals/Urb.SALE02.1.JPG]

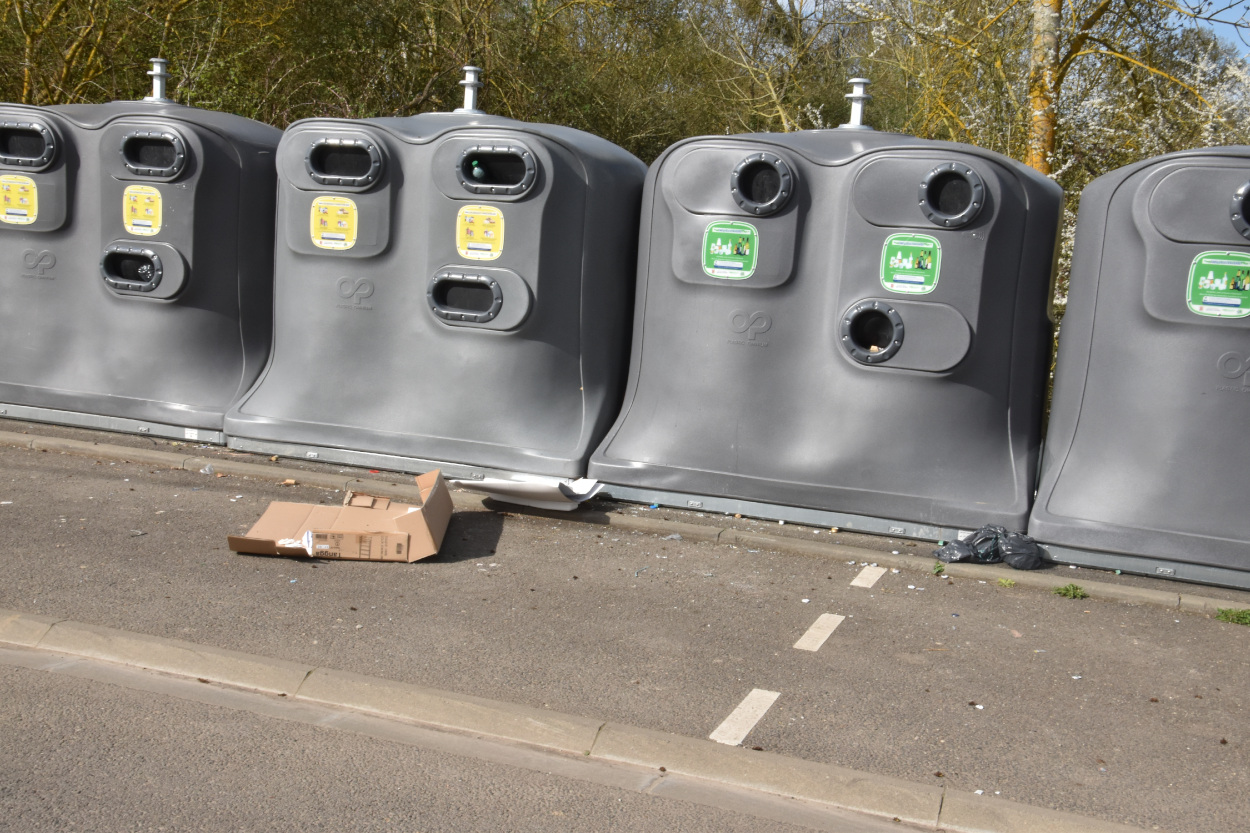

Supplement: S1 Data — (ZIP) [file pone.0234210.s002.zip › Pictures_DataBase_Environment/Polluted/Polluted - Urban/Polluted - Urban - Without Individuals/Urb.SALE06.1.JPG]

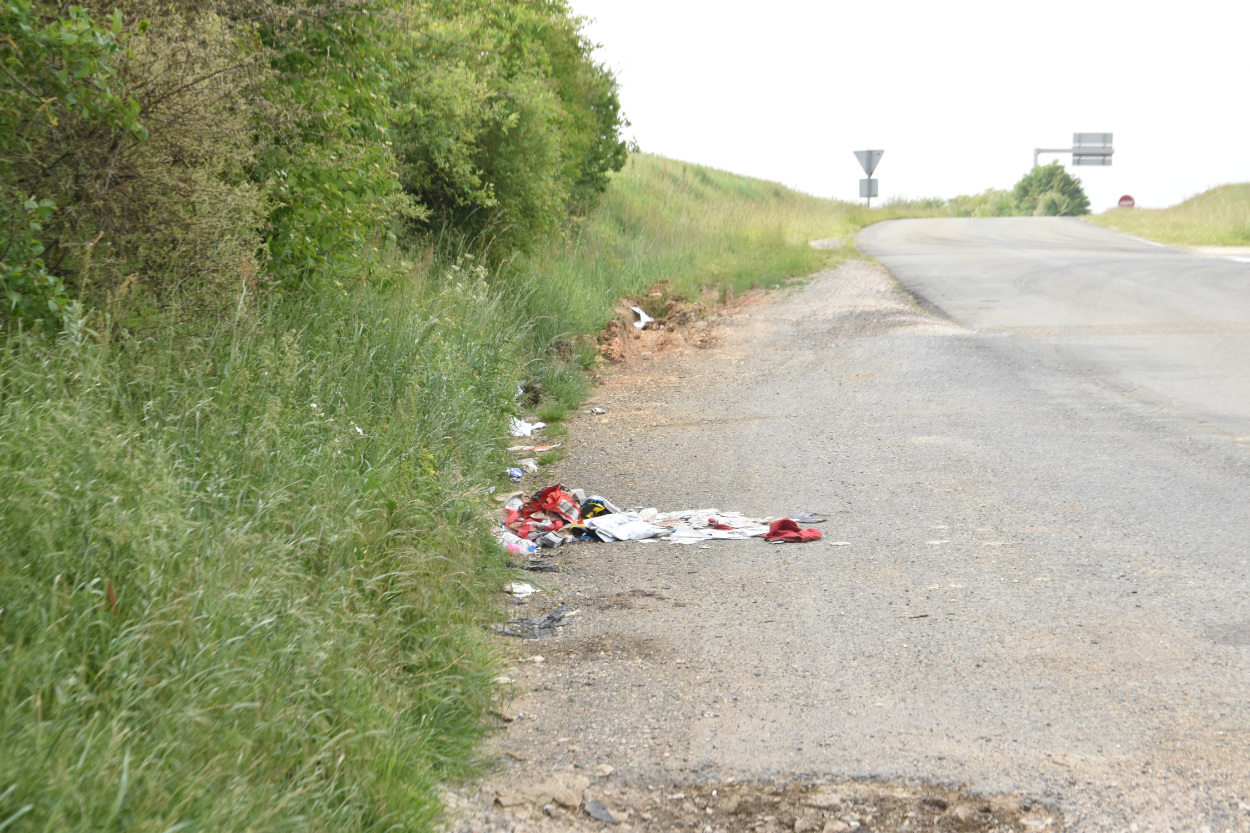

Supplement: S1 Data — (ZIP) [file pone.0234210.s002.zip › Pictures_DataBase_Environment/Polluted/Polluted - Urban/Polluted - Urban - Without Individuals/Urb.SALE22.1.JPG]

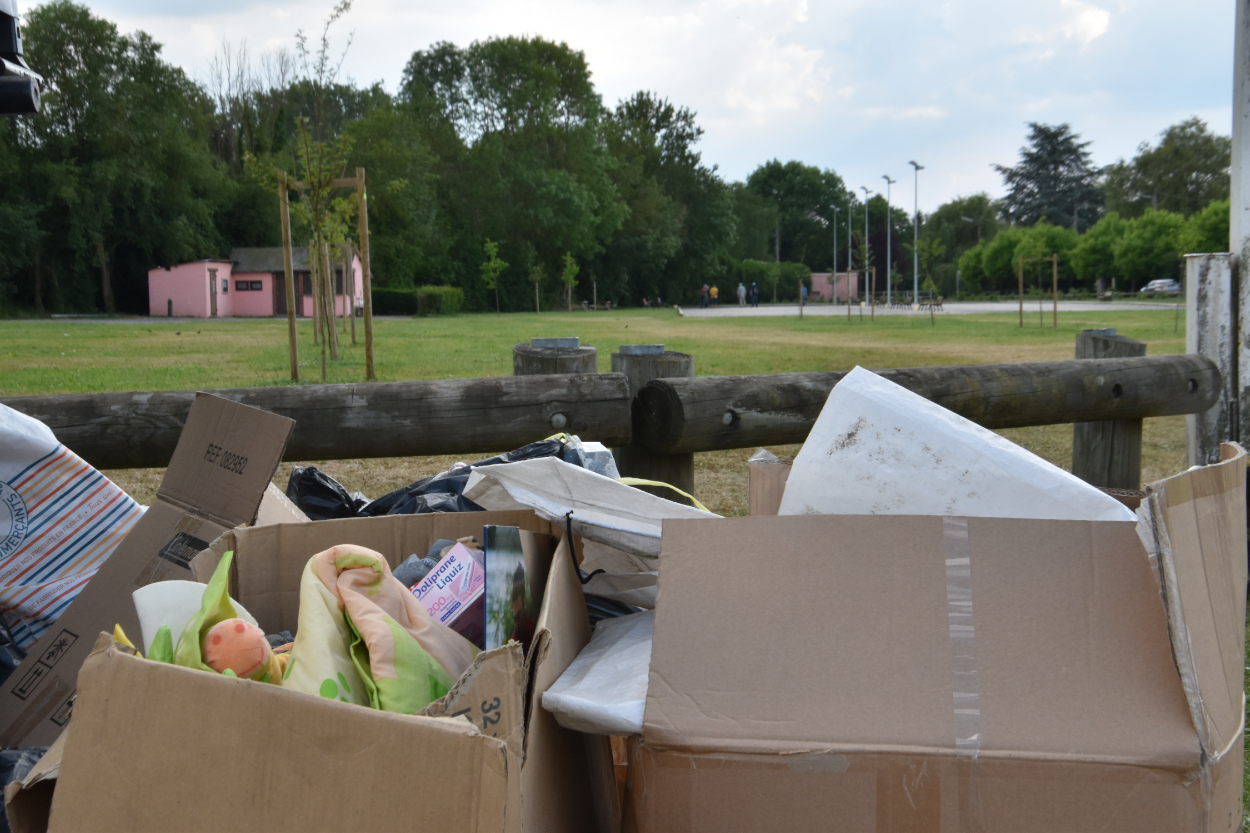

Supplement: S1 Data — (ZIP) [file pone.0234210.s002.zip › Pictures_DataBase_Environment/Polluted/Polluted - Urban/Polluted - Urban - Without Individuals/Urb.SALE20.1.JPG]

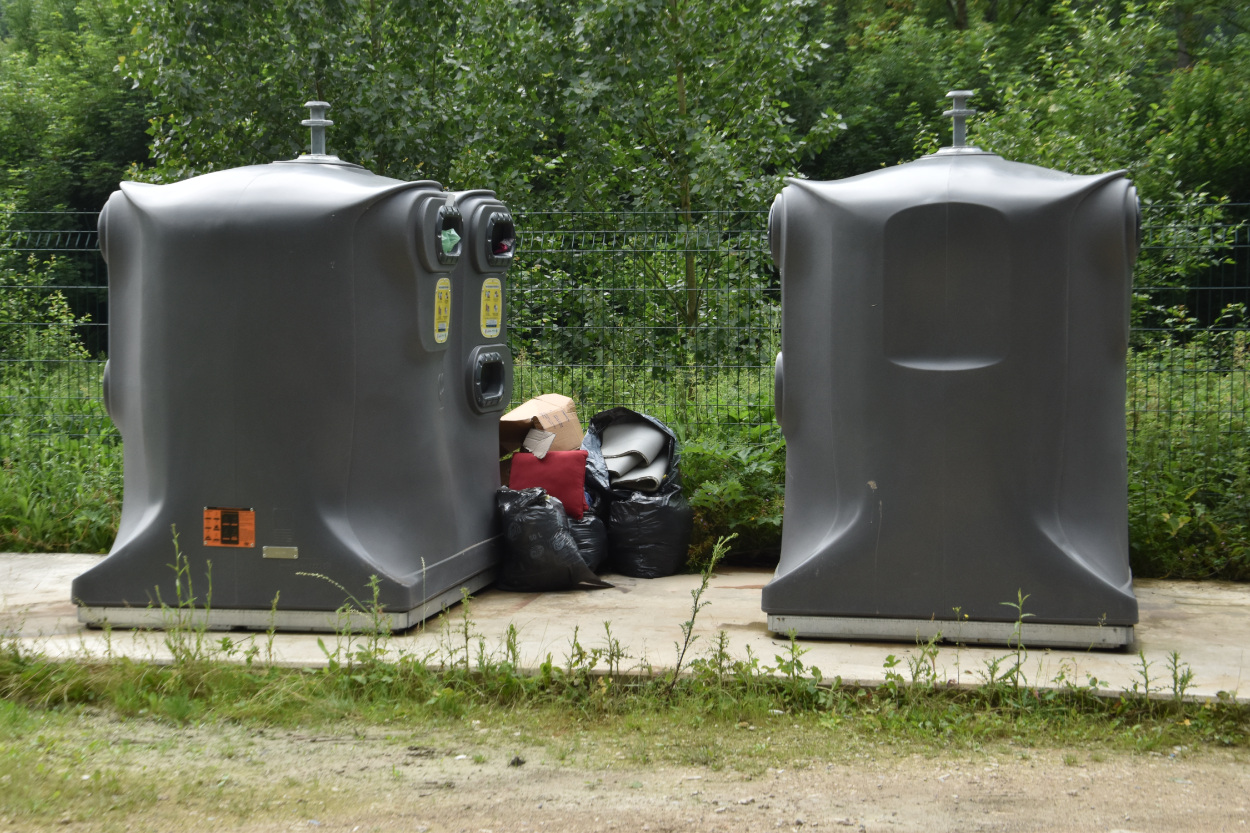

Supplement: S1 Data — (ZIP) [file pone.0234210.s002.zip › Pictures_DataBase_Environment/Polluted/Polluted - Urban/Polluted - Urban - Without Individuals/Urb.SALE04.1.JPG]

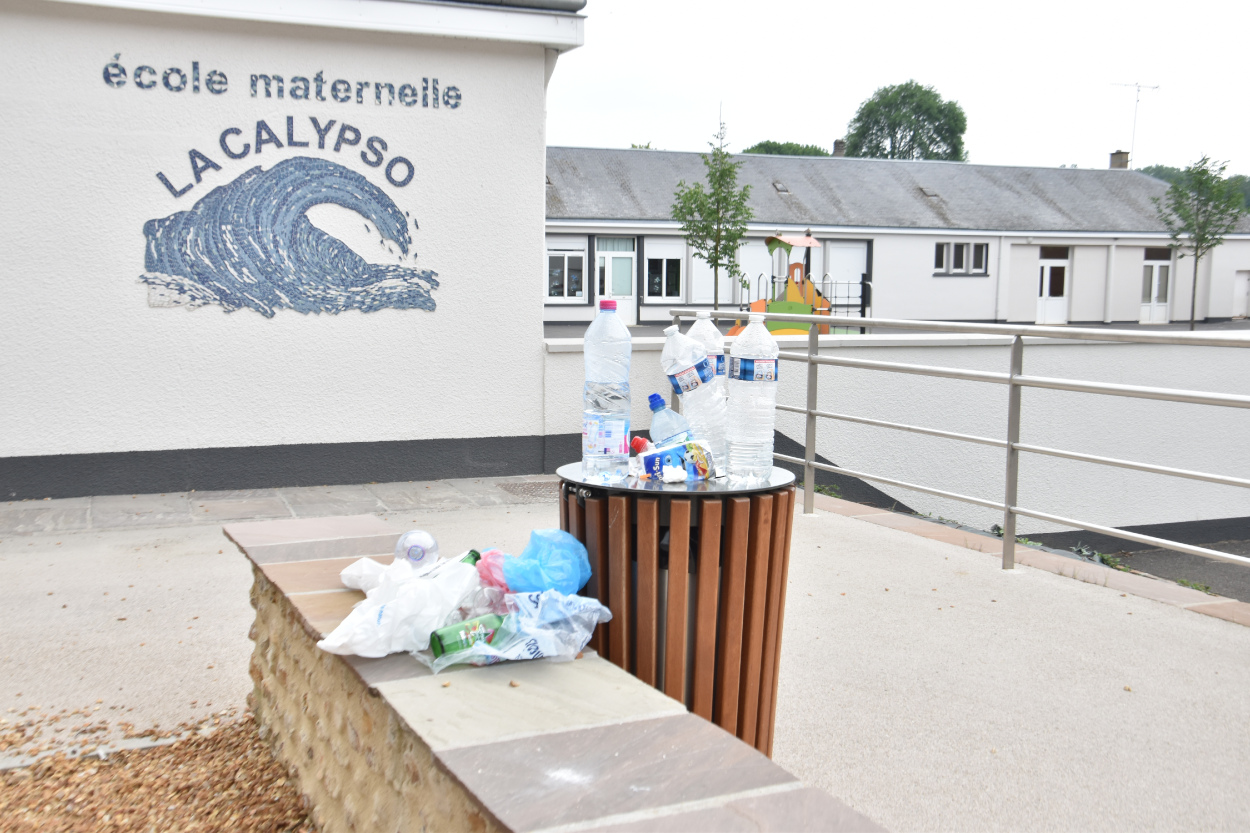

Supplement: S1 Data — (ZIP) [file pone.0234210.s002.zip › Pictures_DataBase_Environment/Polluted/Polluted - Urban/Polluted - Urban - Without Individuals/Urb.SALE03.1.JPG]

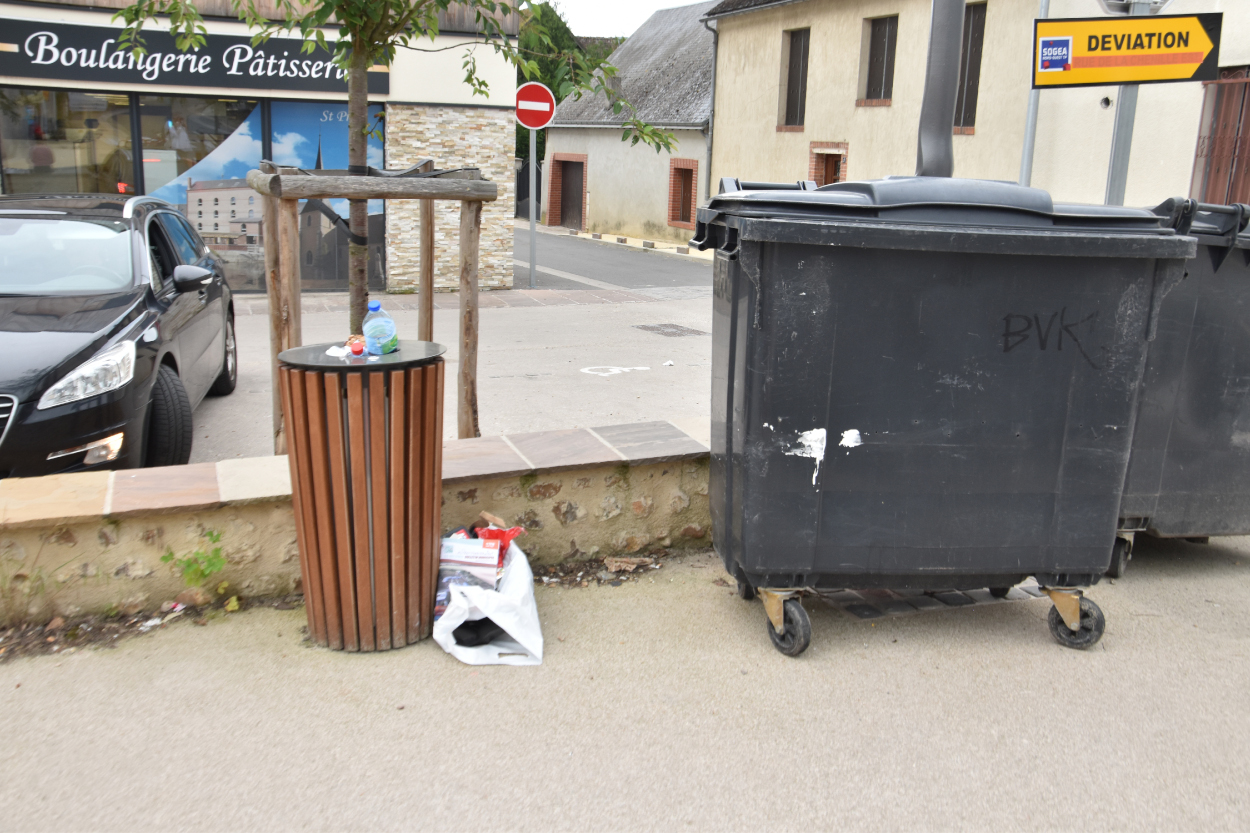

Supplement: S1 Data — (ZIP) [file pone.0234210.s002.zip › Pictures_DataBase_Environment/Polluted/Polluted - Urban/Polluted - Urban - Without Individuals/Urb.SALE01.1.JPG]

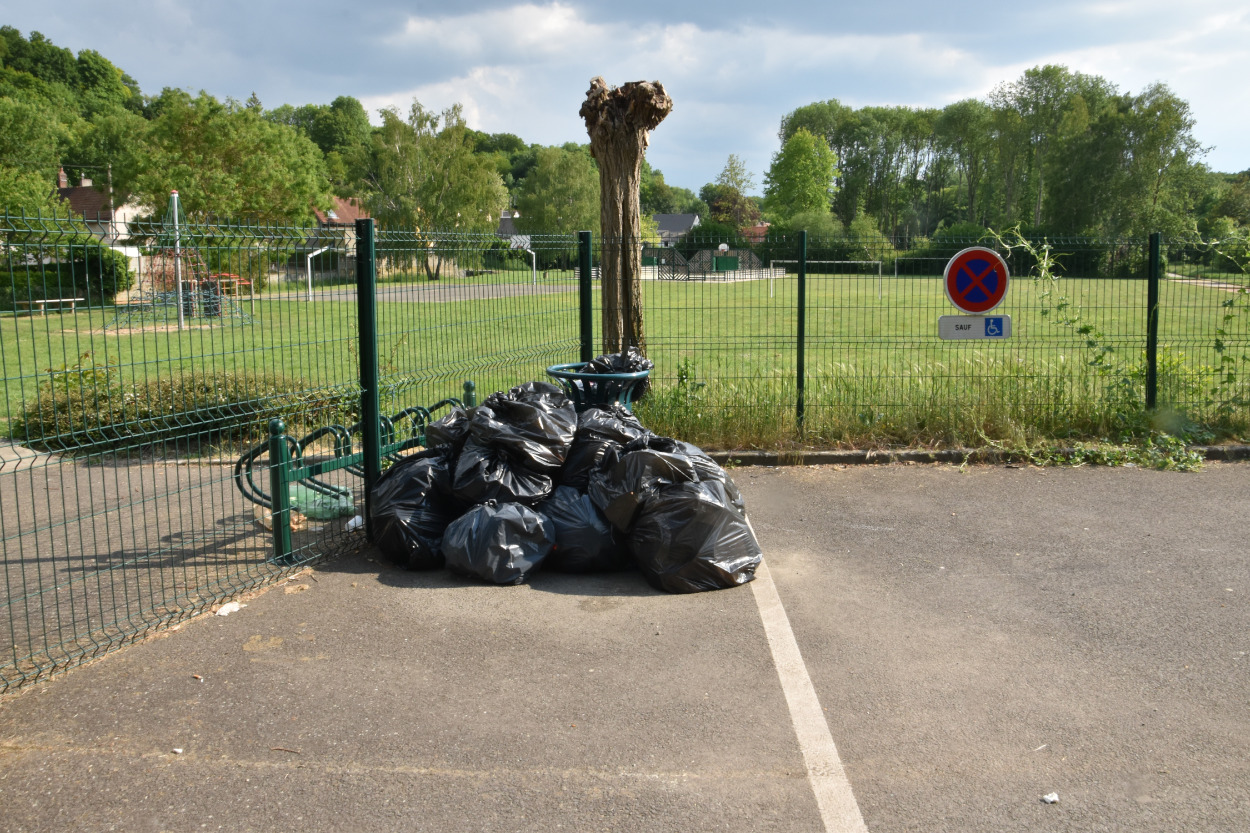

Supplement: S1 Data — (ZIP) [file pone.0234210.s002.zip › Pictures_DataBase_Environment/Polluted/Polluted - Urban/Polluted - Urban - Without Individuals/Urb.SALE18.1.JPG]

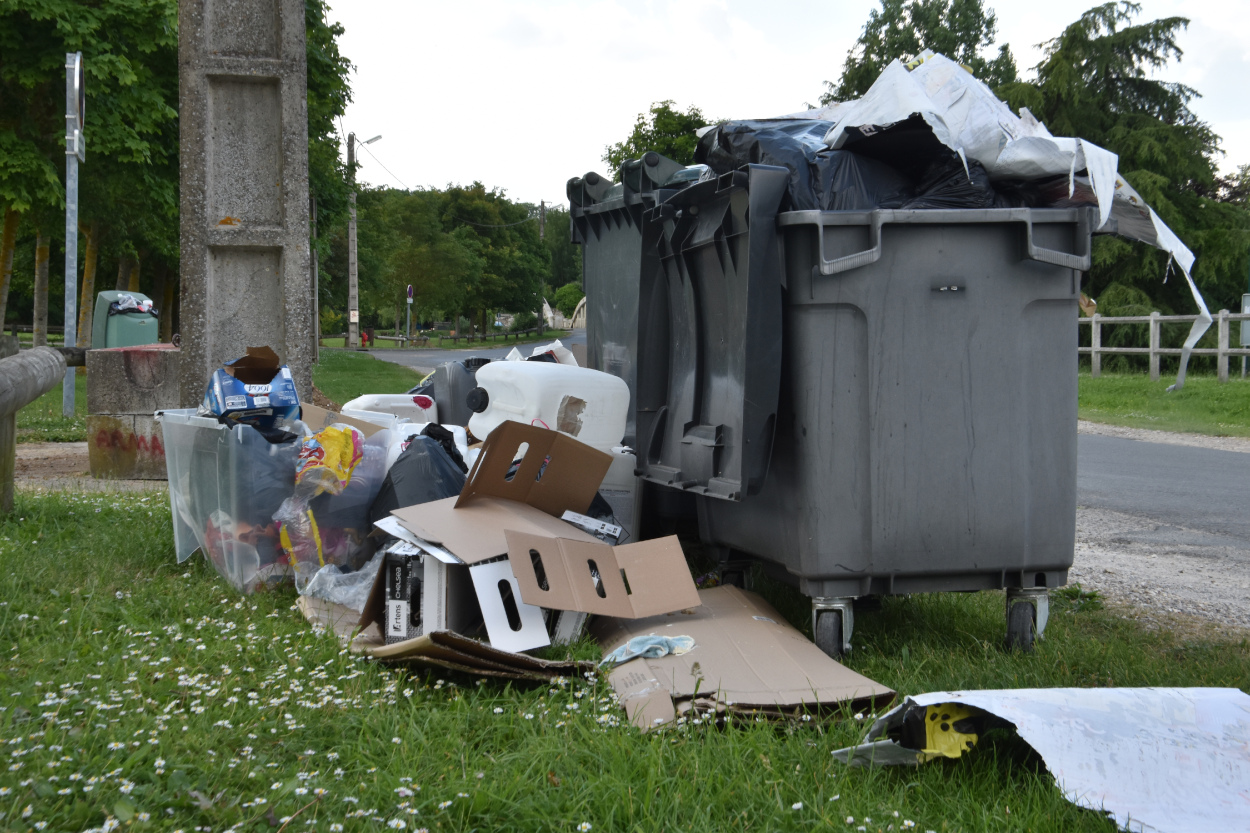

Supplement: S1 Data — (ZIP) [file pone.0234210.s002.zip › Pictures_DataBase_Environment/Polluted/Polluted - Urban/Polluted - Urban - Without Individuals/Urb.SALE21.1.JPG]

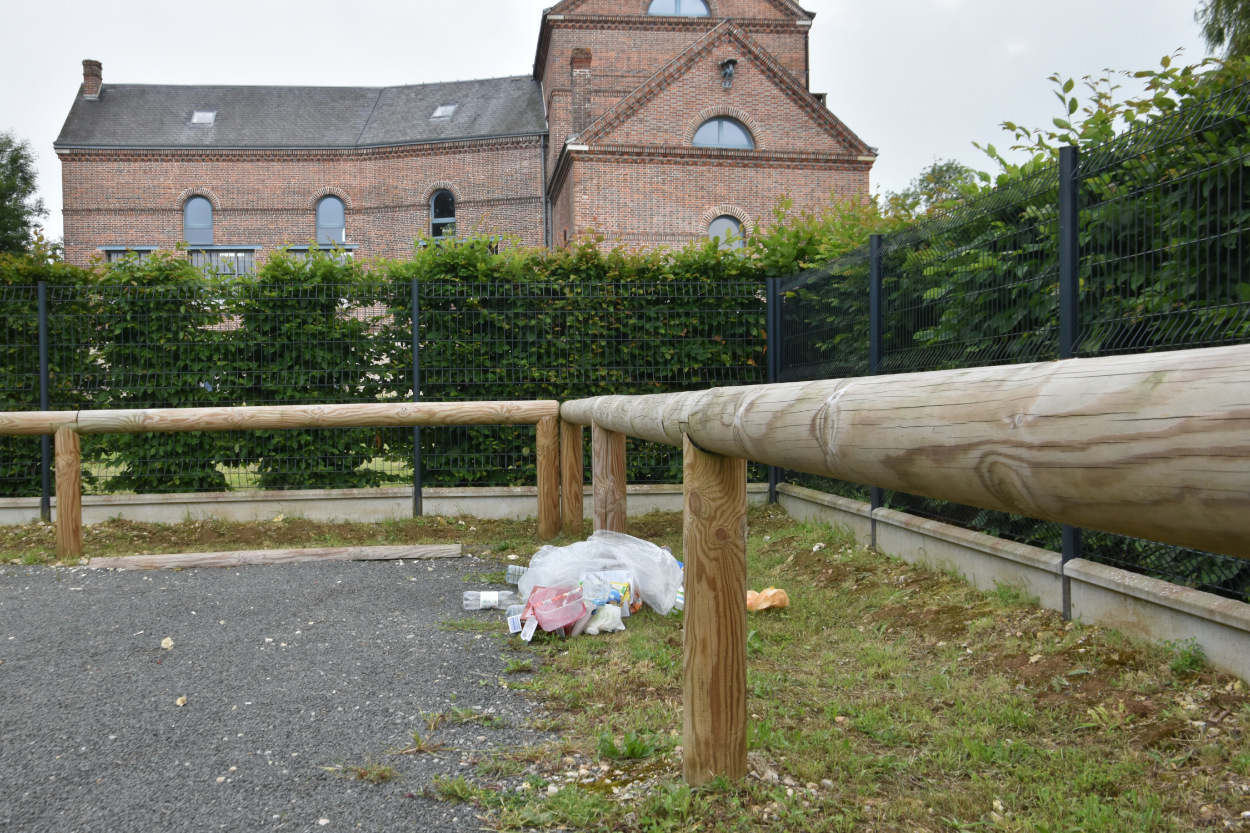

Supplement: S1 Data — (ZIP) [file pone.0234210.s002.zip › Pictures_DataBase_Environment/Polluted/Polluted - Urban/Polluted - Urban - Without Individuals/Urb.SALE05.1.JPG]

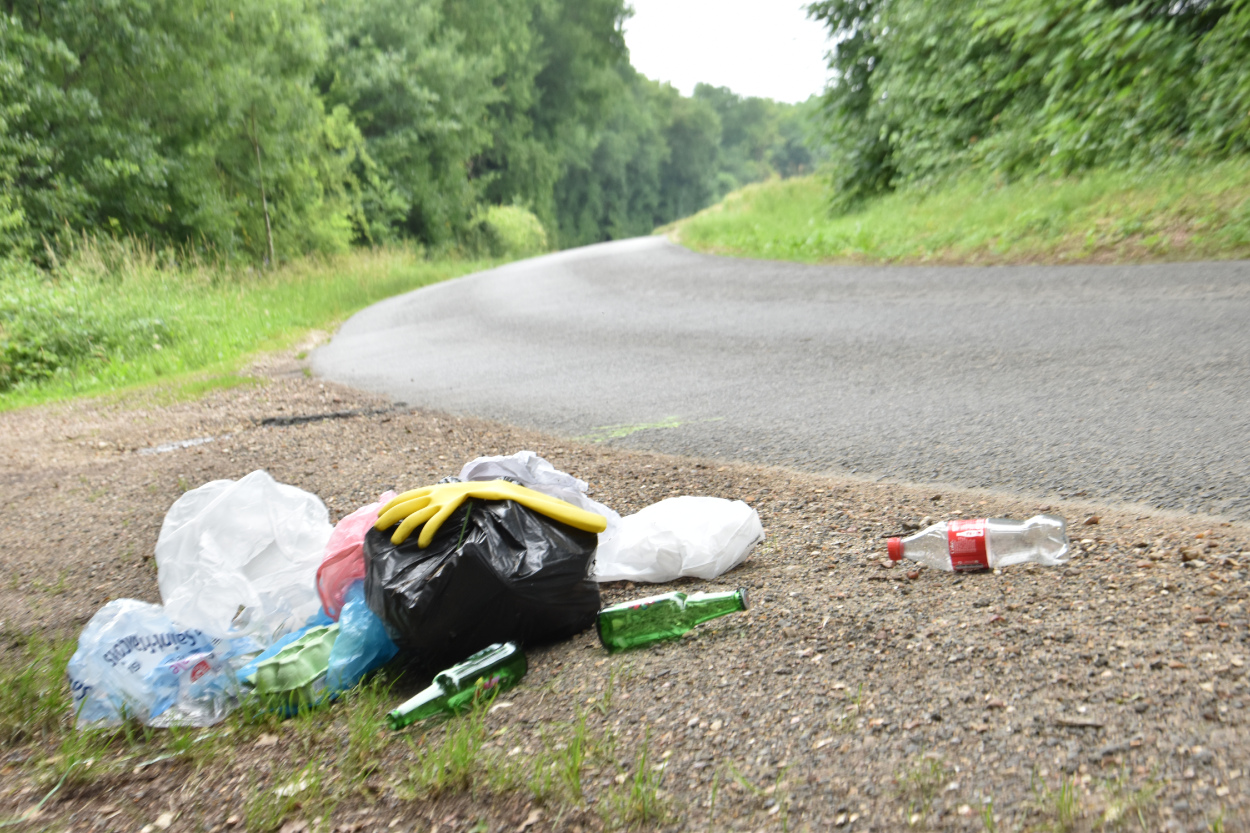

Supplement: S1 Data — (ZIP) [file pone.0234210.s002.zip › Pictures_DataBase_Environment/Polluted/Polluted - Urban/Polluted - Urban - Without Individuals/Urb.SALE23.1.JPG]

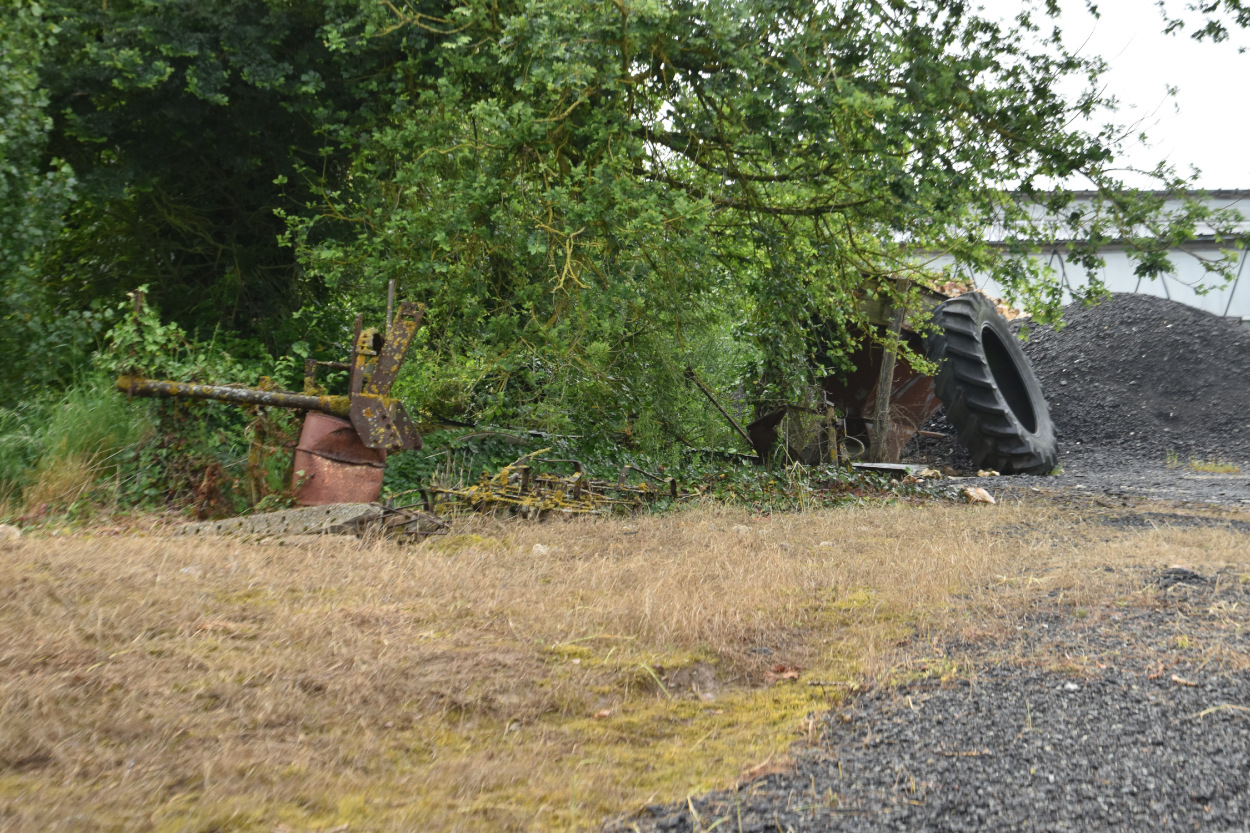

Supplement: S1 Data — (ZIP) [file pone.0234210.s002.zip › Pictures_DataBase_Environment/Polluted/Polluted - Urban/Polluted - Urban - Without Individuals/Urb.SALE12.1.JPG]

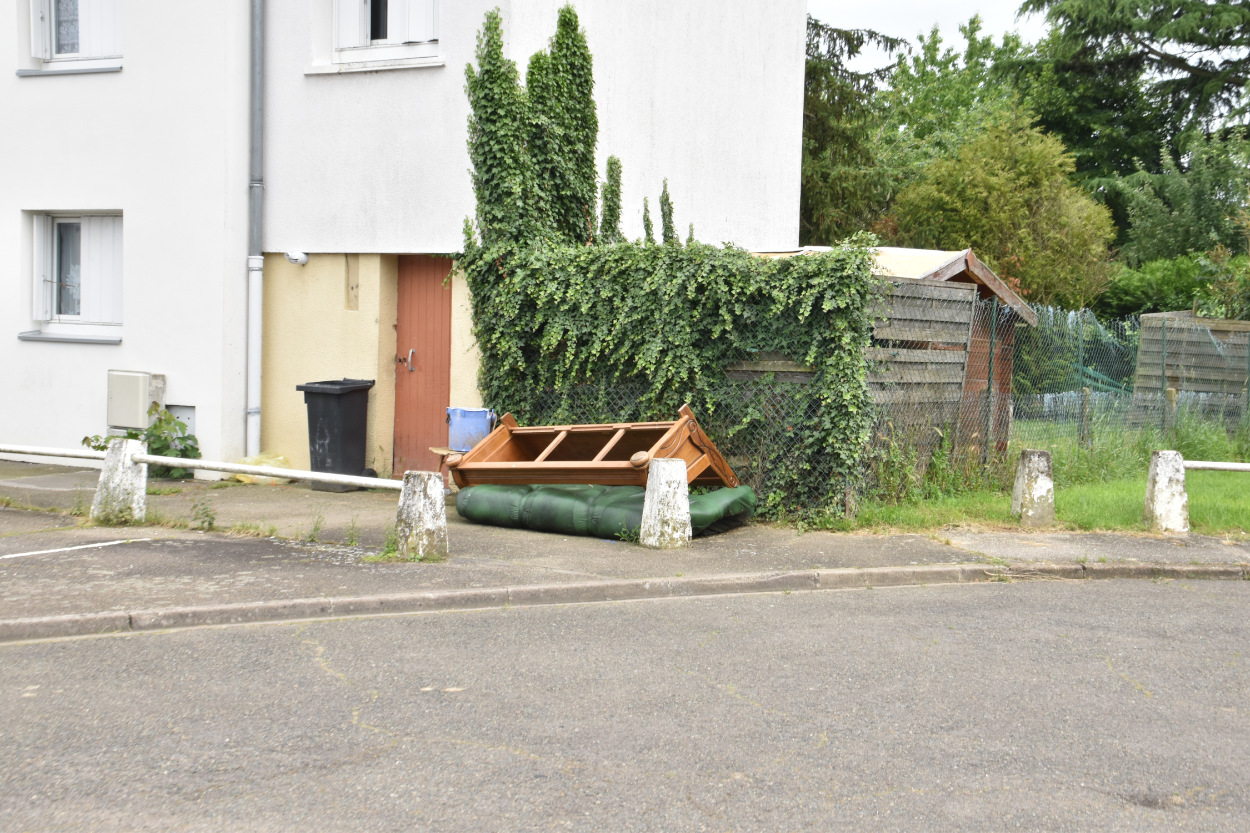

Supplement: S1 Data — (ZIP) [file pone.0234210.s002.zip › Pictures_DataBase_Environment/Polluted/Polluted - Urban/Polluted - Urban - Without Individuals/Urb.SALE09.1.JPG]

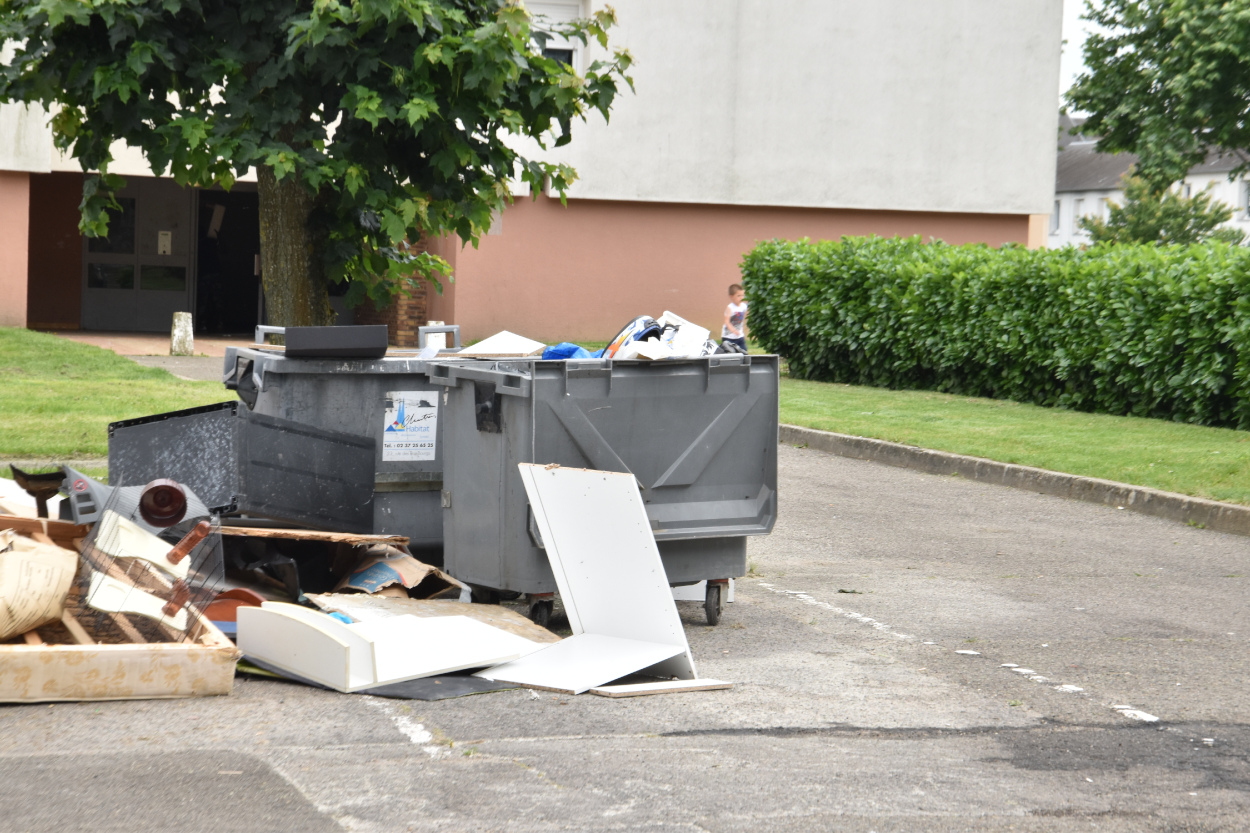

Supplement: S1 Data — (ZIP) [file pone.0234210.s002.zip › Pictures_DataBase_Environment/Polluted/Polluted - Urban/Polluted - Urban - Without Individuals/Urb.SALE10.1.JPG]

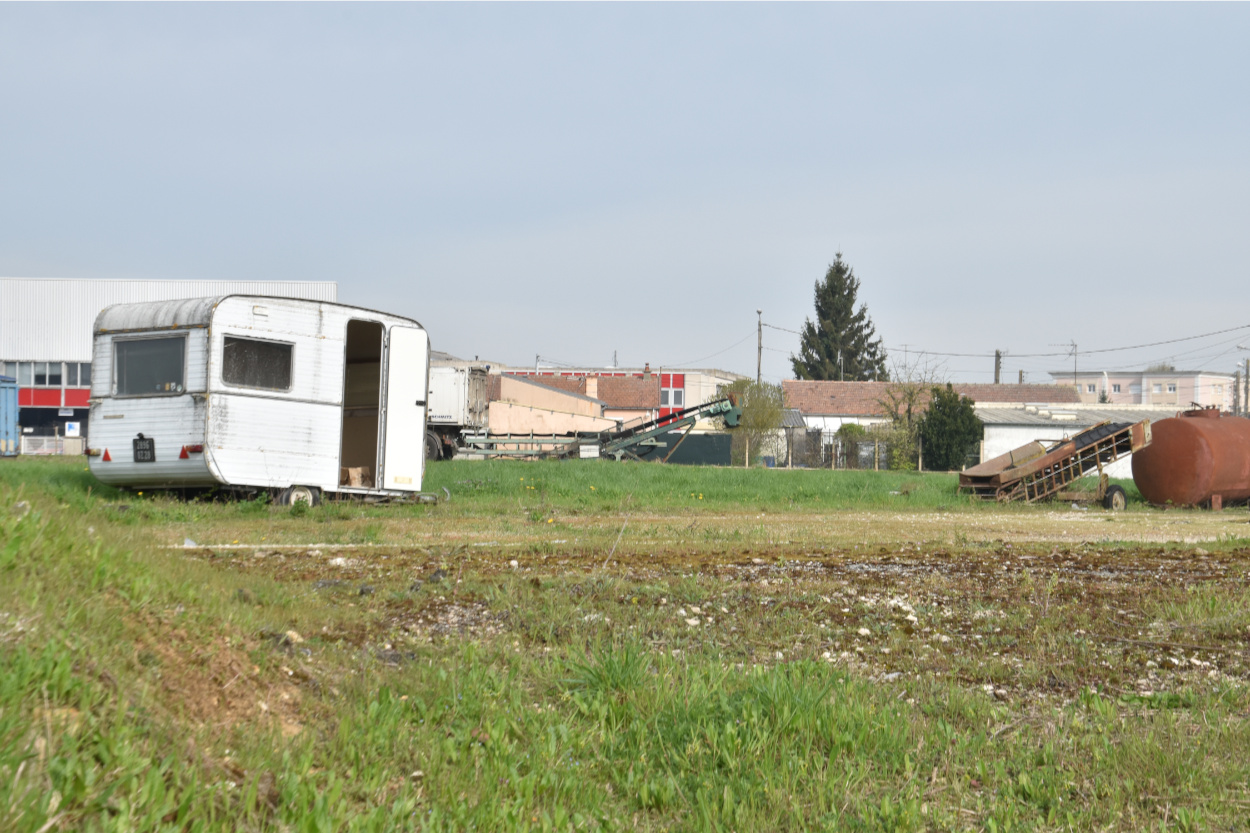

Supplement: S1 Data — (ZIP) [file pone.0234210.s002.zip › Pictures_DataBase_Environment/Polluted/Polluted - Urban/Polluted - Urban - Without Individuals/Urb.SALE16.1.JPG]

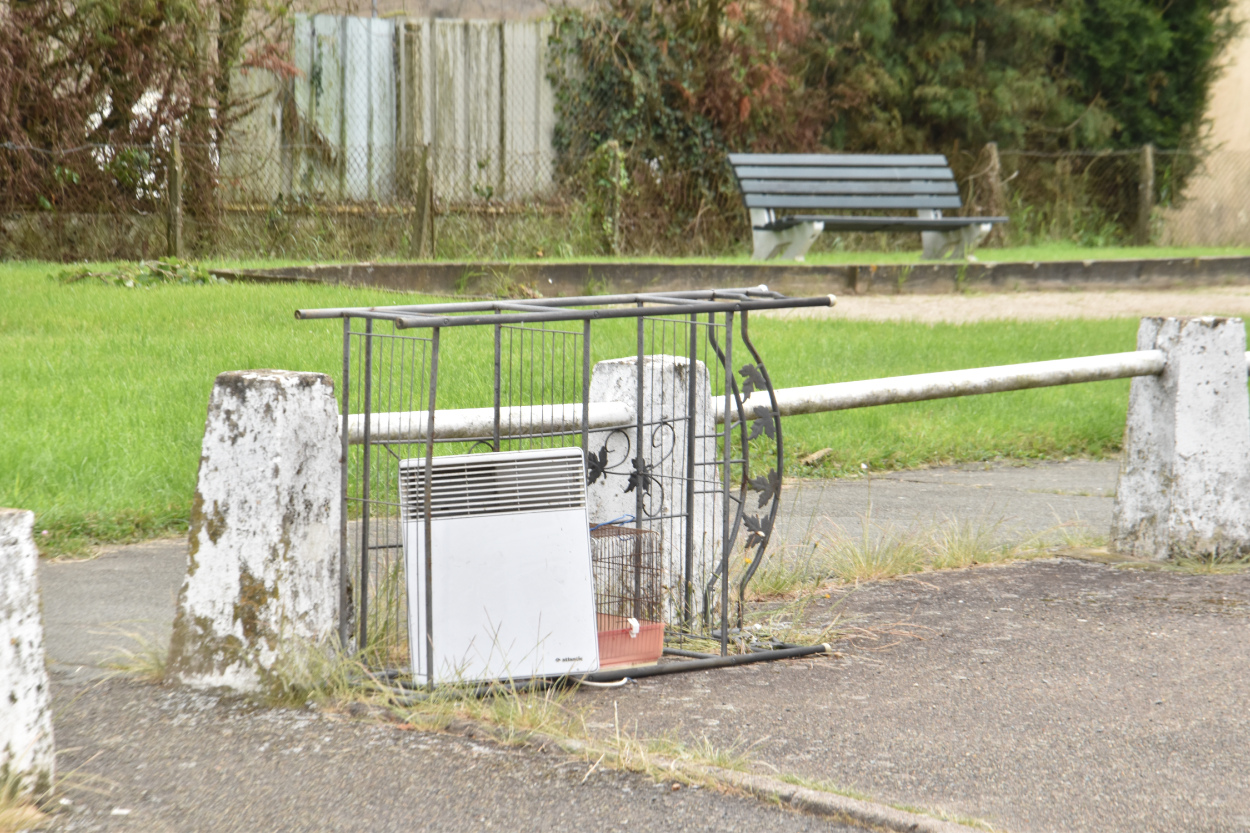

Supplement: S1 Data — (ZIP) [file pone.0234210.s002.zip › Pictures_DataBase_Environment/Polluted/Polluted - Urban/Polluted - Urban - Without Individuals/Urb.SALE08.1.JPG]

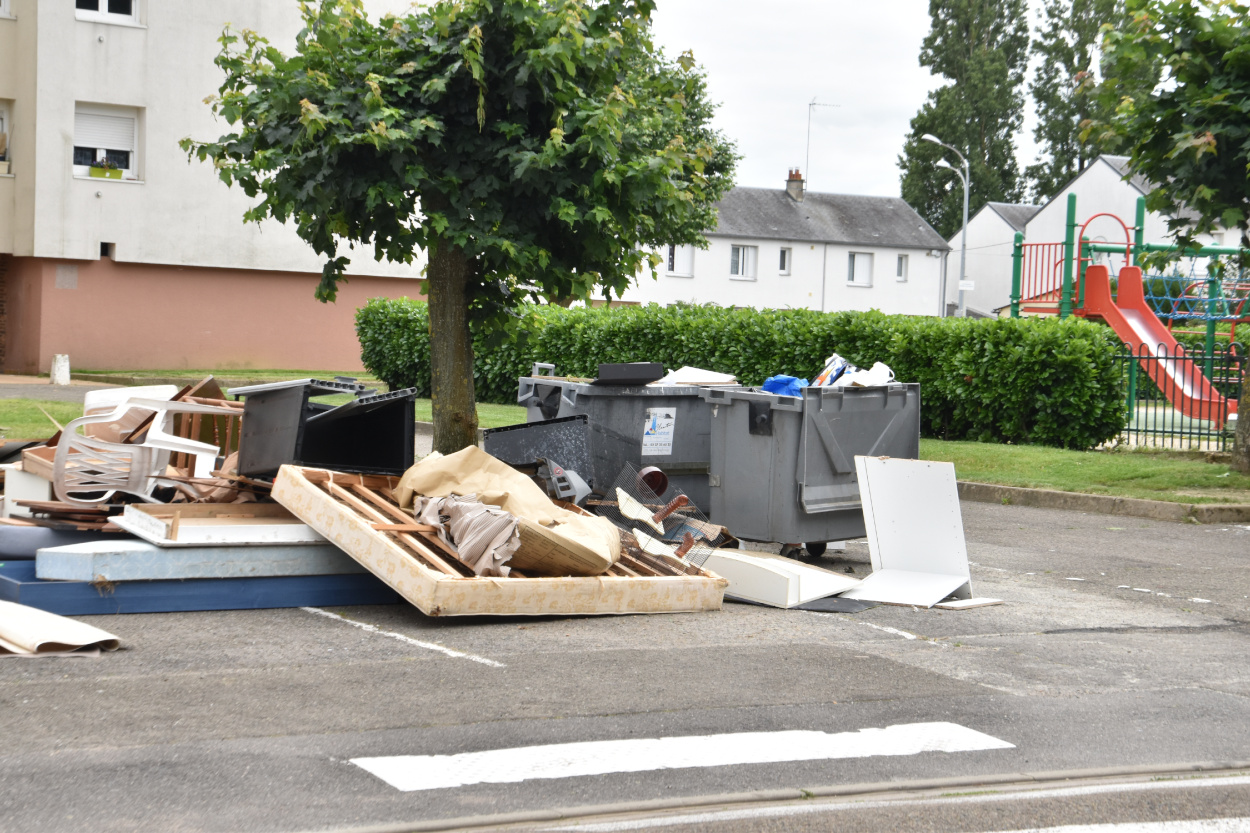

Supplement: S1 Data — (ZIP) [file pone.0234210.s002.zip › Pictures_DataBase_Environment/Polluted/Polluted - Urban/Polluted - Urban - Without Individuals/Urb.SALE11.1.JPG]

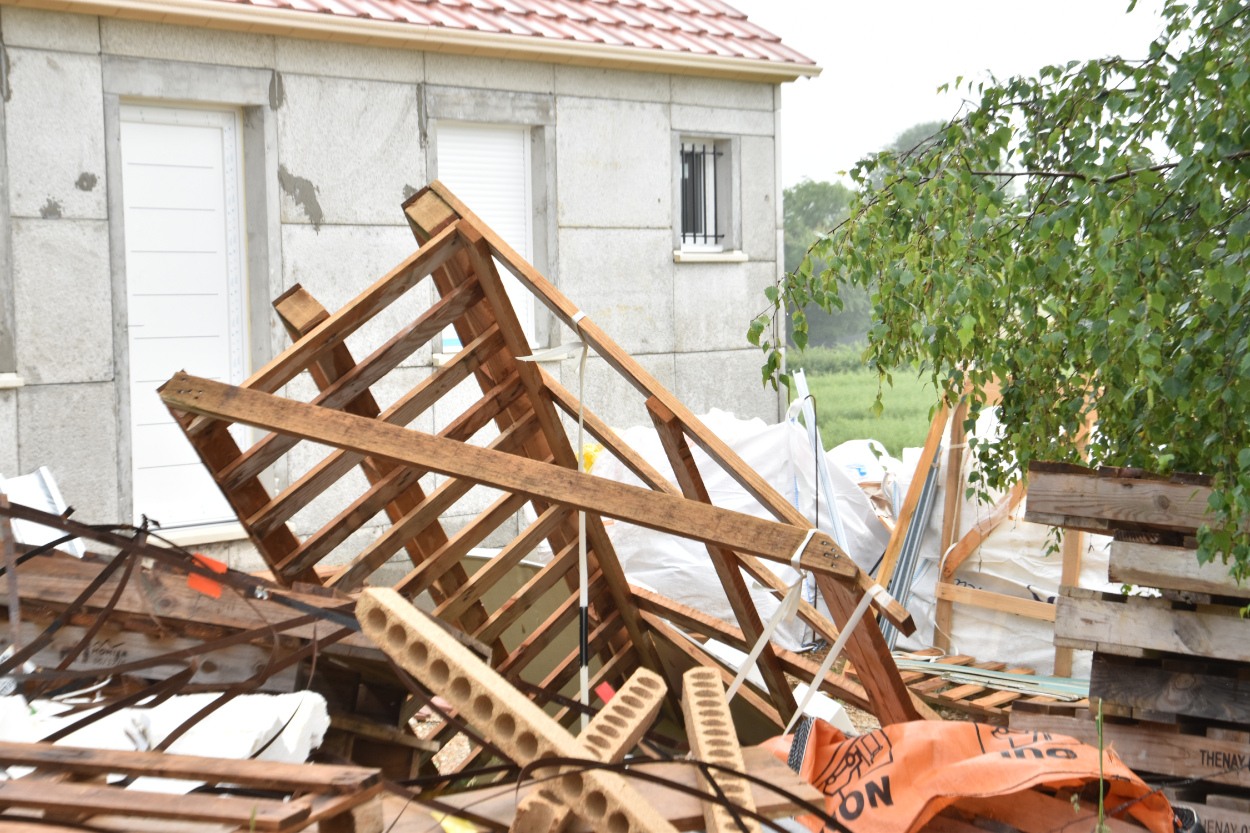

Supplement: S1 Data — (ZIP) [file pone.0234210.s002.zip › Pictures_DataBase_Environment/Polluted/Polluted - Urban/Polluted - Urban - Without Individuals/Urb.SALE13.1.JPG]

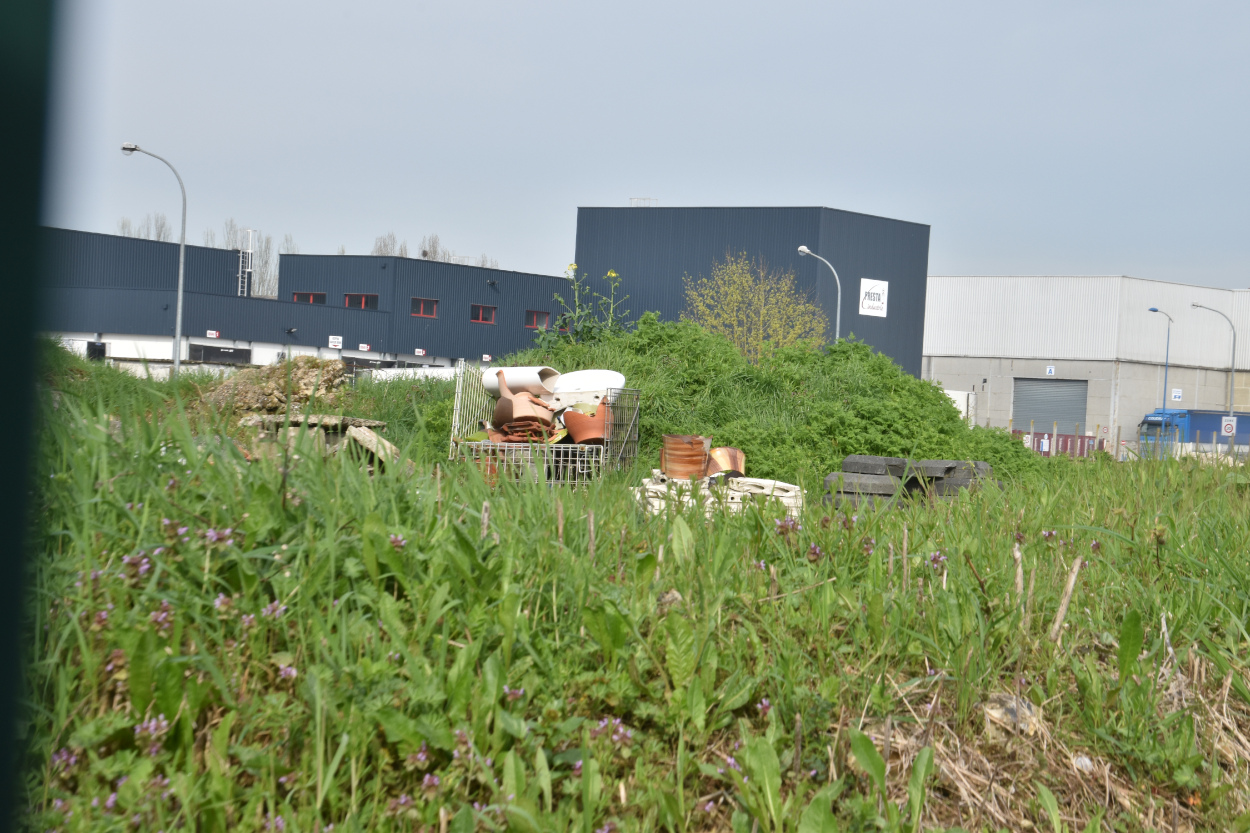

Supplement: S1 Data — (ZIP) [file pone.0234210.s002.zip › Pictures_DataBase_Environment/Polluted/Polluted - Urban/Polluted - Urban - Without Individuals/Urb.SALE15.1.JPG]

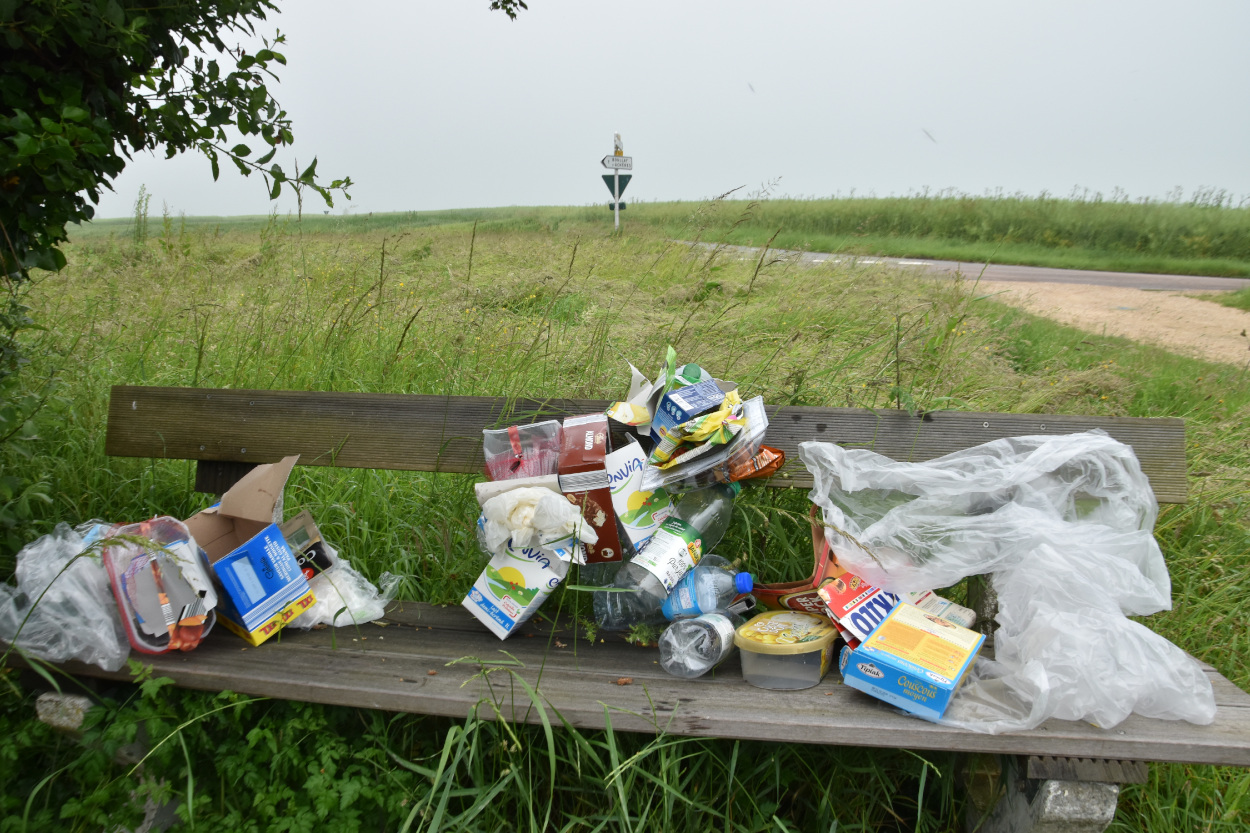

Supplement: S1 Data — (ZIP) [file pone.0234210.s002.zip › Pictures_DataBase_Environment/Polluted/Polluted - Rural/Polluted - Rural - Without Individuals/Rur.SALE14.1.JPG]

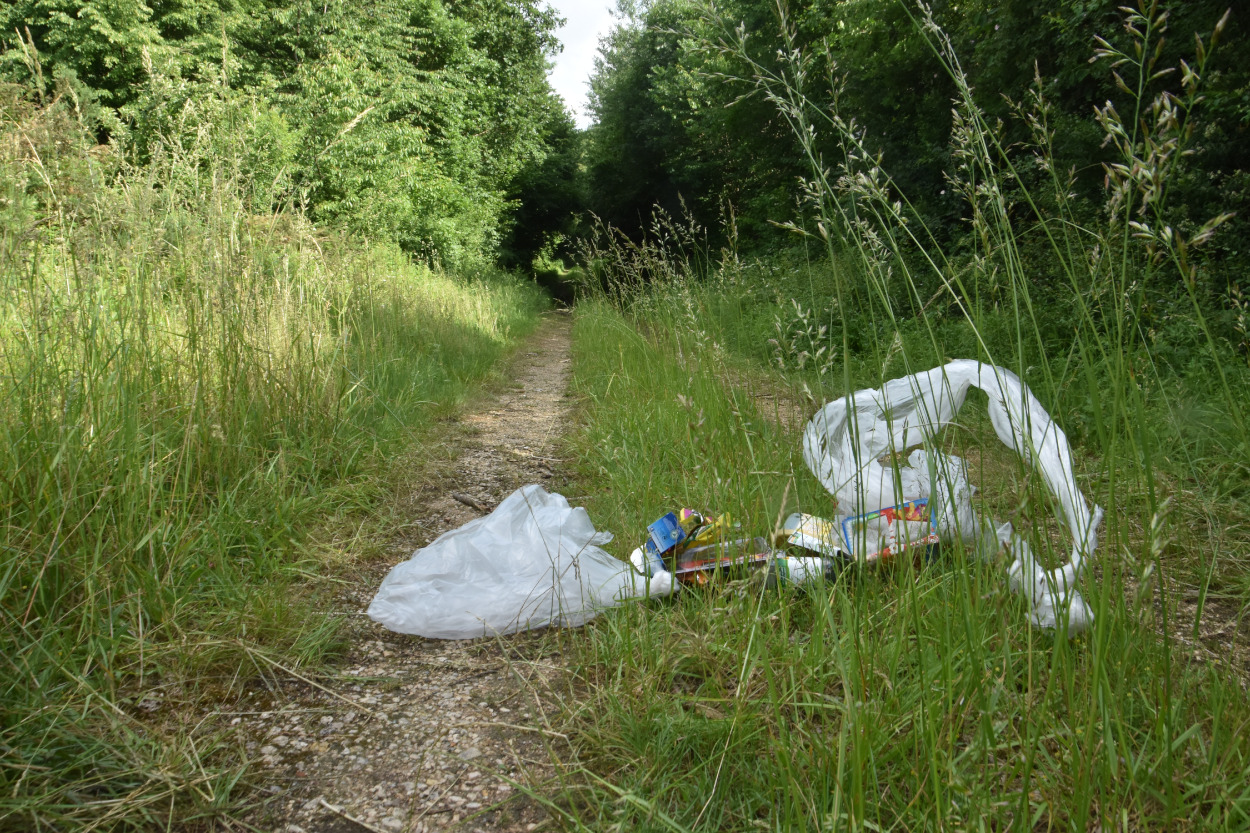

Supplement: S1 Data — (ZIP) [file pone.0234210.s002.zip › Pictures_DataBase_Environment/Polluted/Polluted - Rural/Polluted - Rural - Without Individuals/Rur.SALE16.1.JPG]

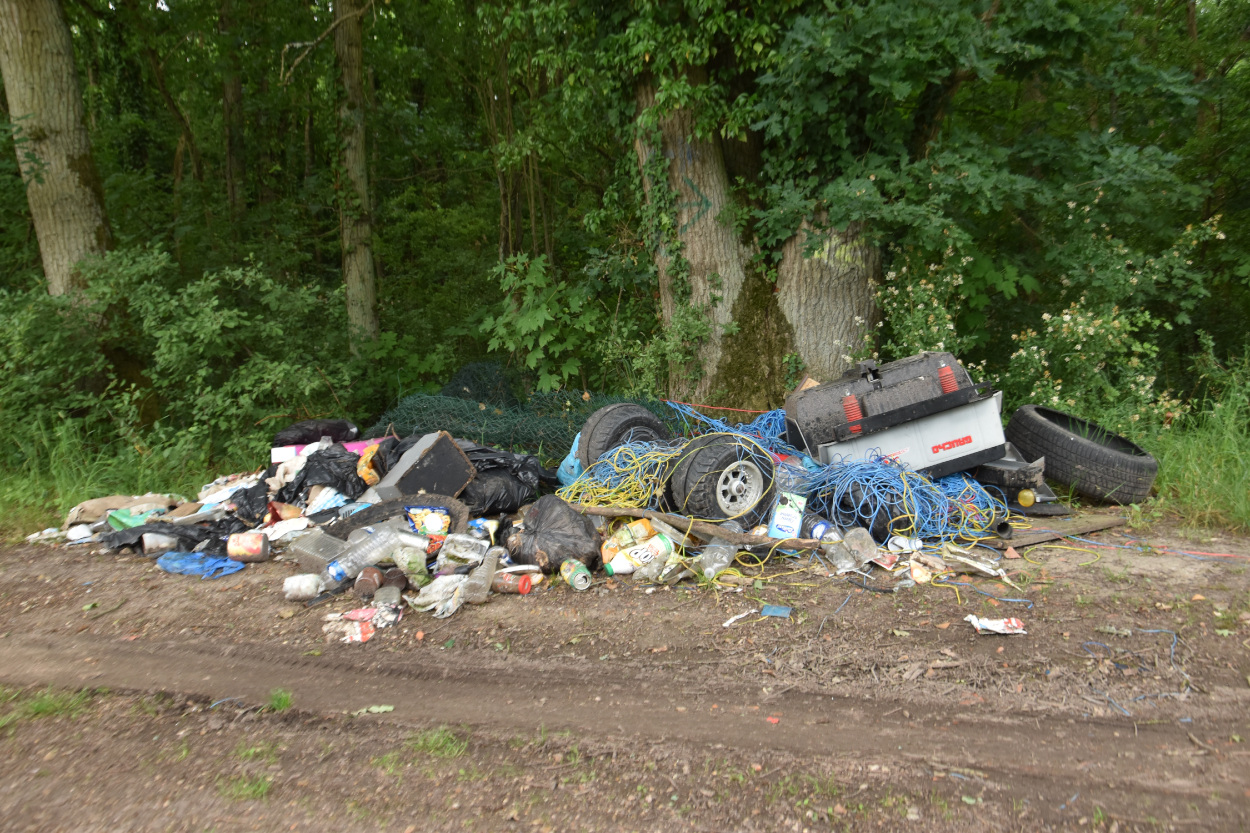

Supplement: S1 Data — (ZIP) [file pone.0234210.s002.zip › Pictures_DataBase_Environment/Polluted/Polluted - Rural/Polluted - Rural - Without Individuals/Rur.SALE12.1.JPG]

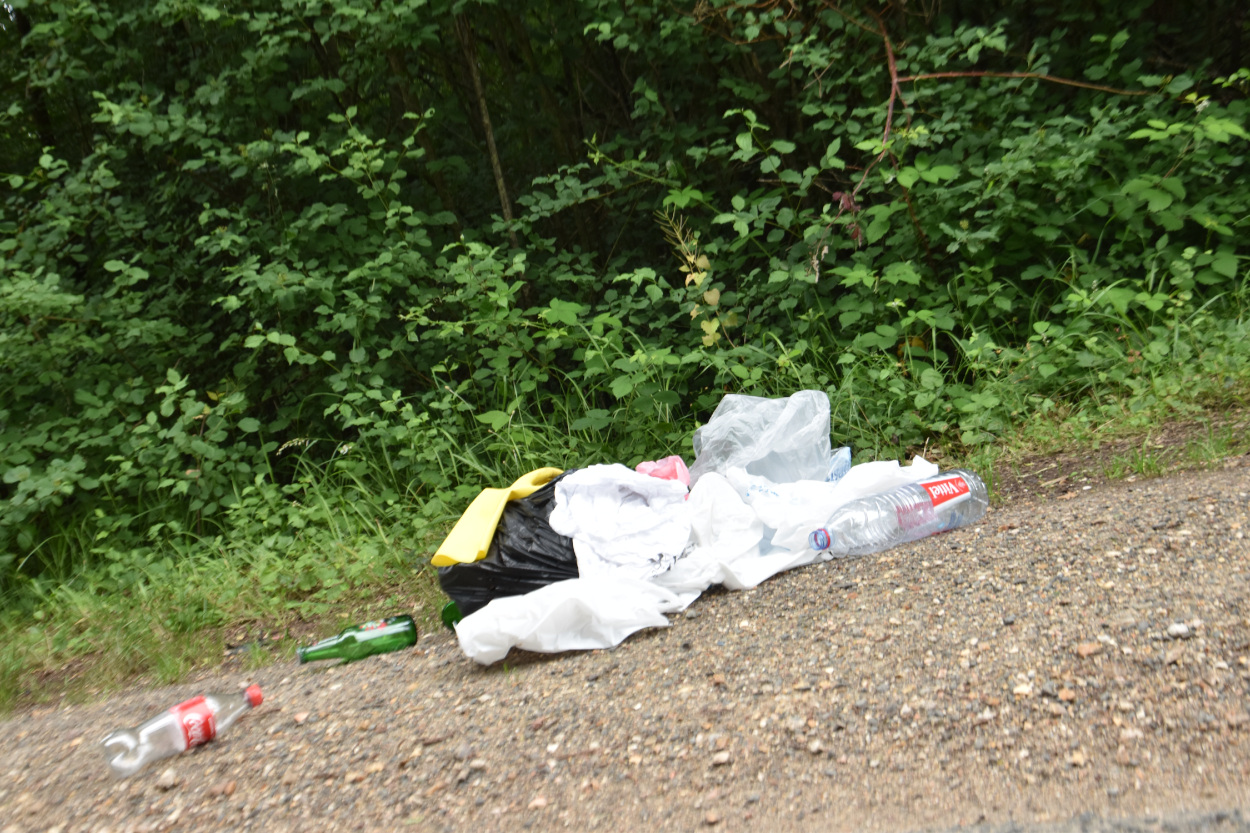

Supplement: S1 Data — (ZIP) [file pone.0234210.s002.zip › Pictures_DataBase_Environment/Polluted/Polluted - Rural/Polluted - Rural - Without Individuals/Rur.SALE09.1.JPG]

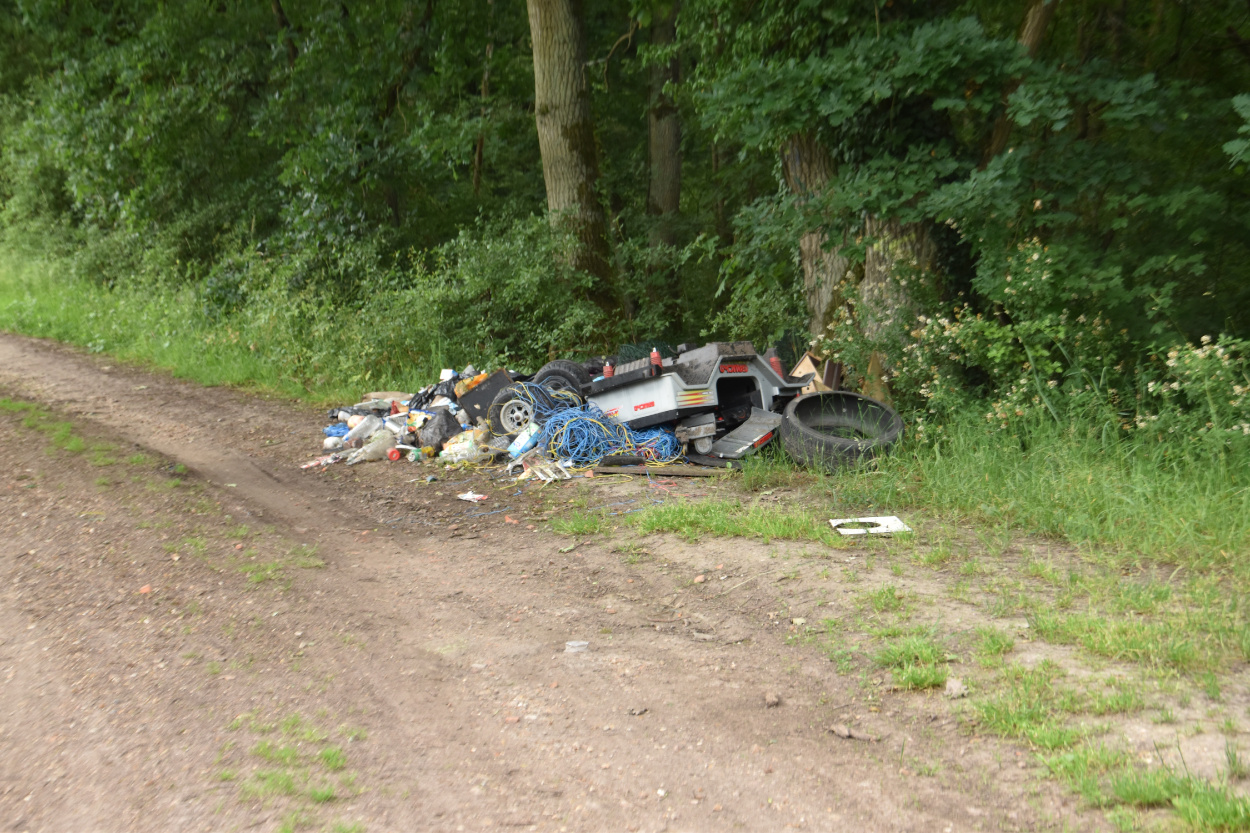

Supplement: S1 Data — (ZIP) [file pone.0234210.s002.zip › Pictures_DataBase_Environment/Polluted/Polluted - Rural/Polluted - Rural - Without Individuals/Rur.SALE10.1.JPG]

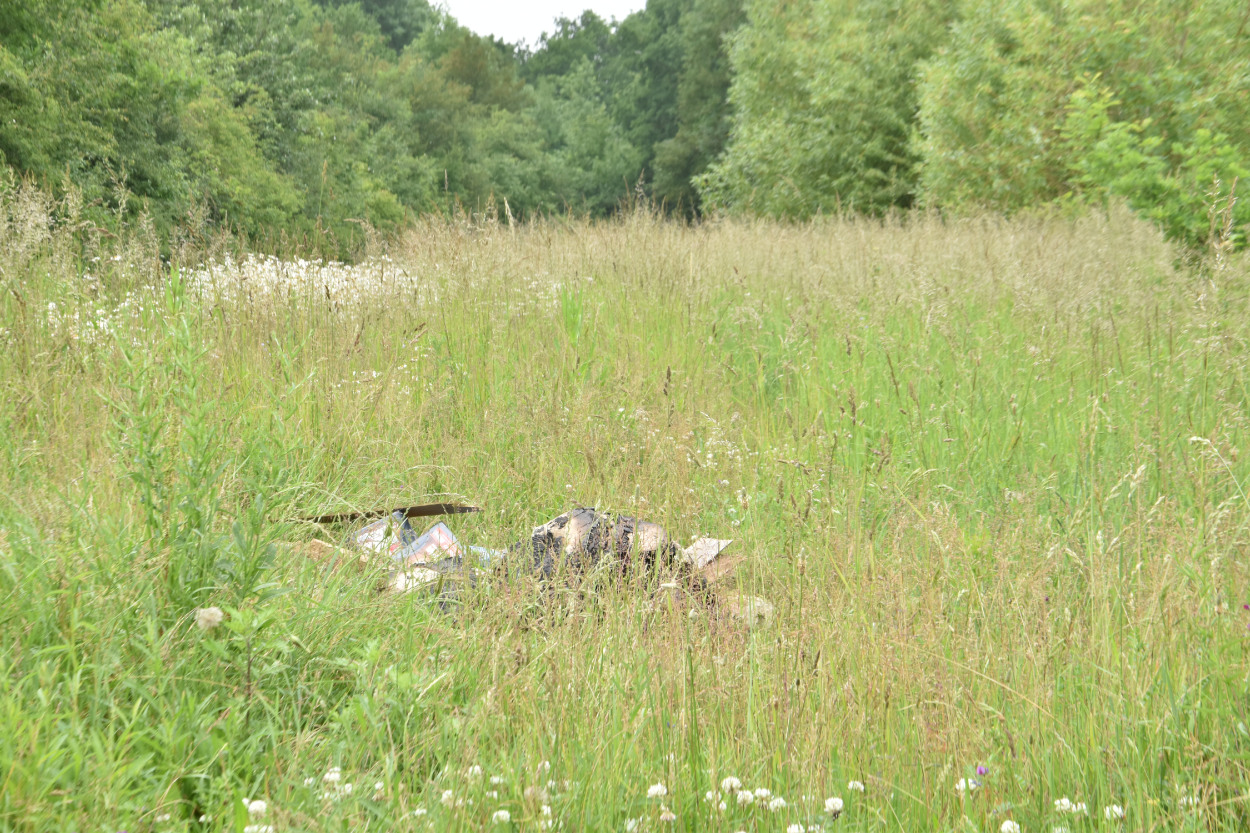

Supplement: S1 Data — (ZIP) [file pone.0234210.s002.zip › Pictures_DataBase_Environment/Polluted/Polluted - Rural/Polluted - Rural - Without Individuals/Rur.SALE17.1.JPG]

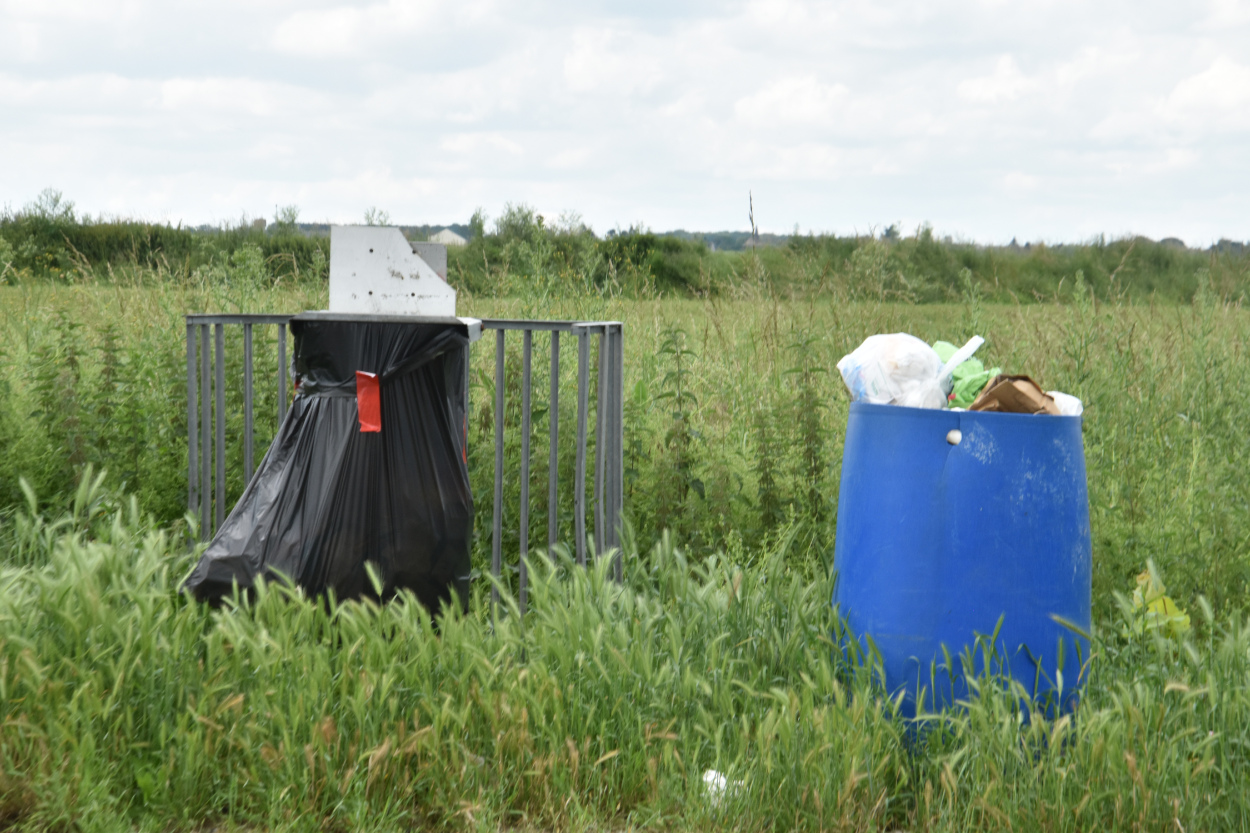

Supplement: S1 Data — (ZIP) [file pone.0234210.s002.zip › Pictures_DataBase_Environment/Polluted/Polluted - Rural/Polluted - Rural - Without Individuals/Rur.SALE15.1.JPG]

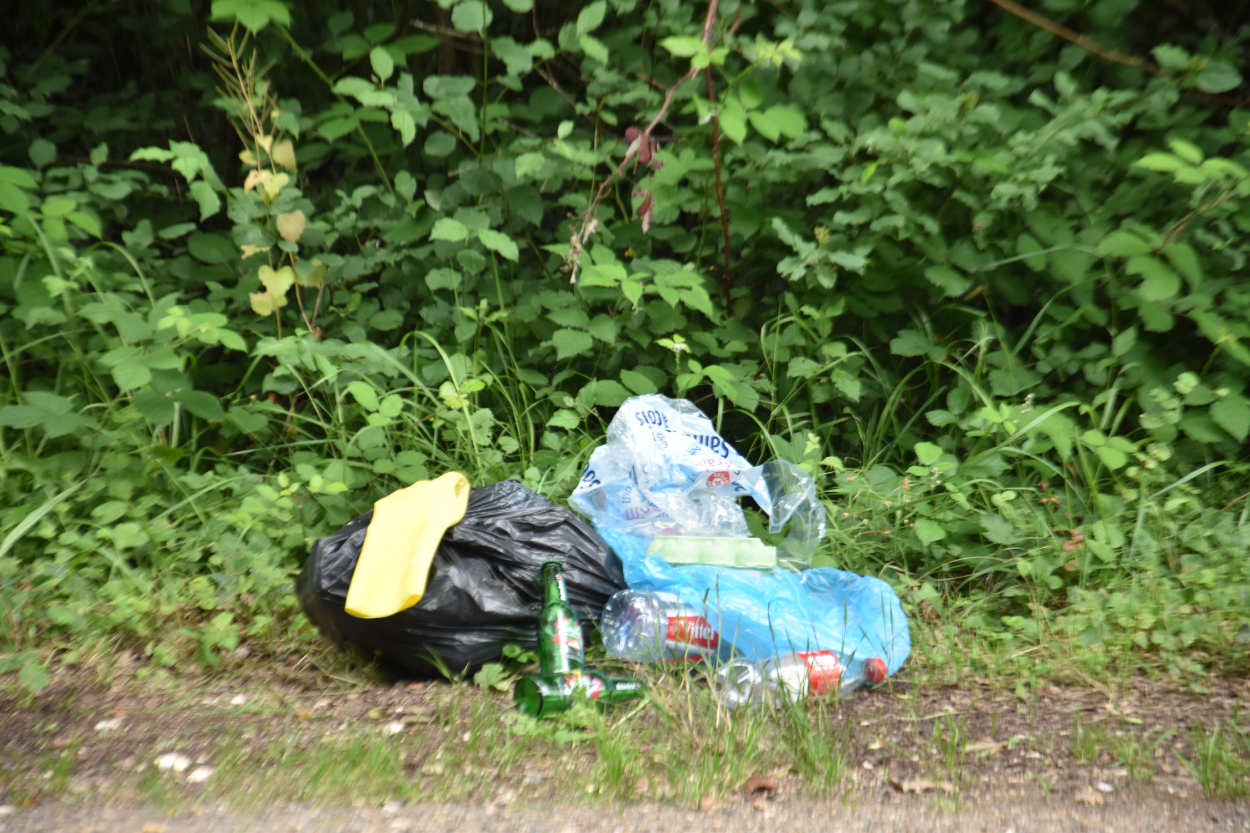

Supplement: S1 Data — (ZIP) [file pone.0234210.s002.zip › Pictures_DataBase_Environment/Polluted/Polluted - Rural/Polluted - Rural - Without Individuals/Rur.SALE08.1.JPG]

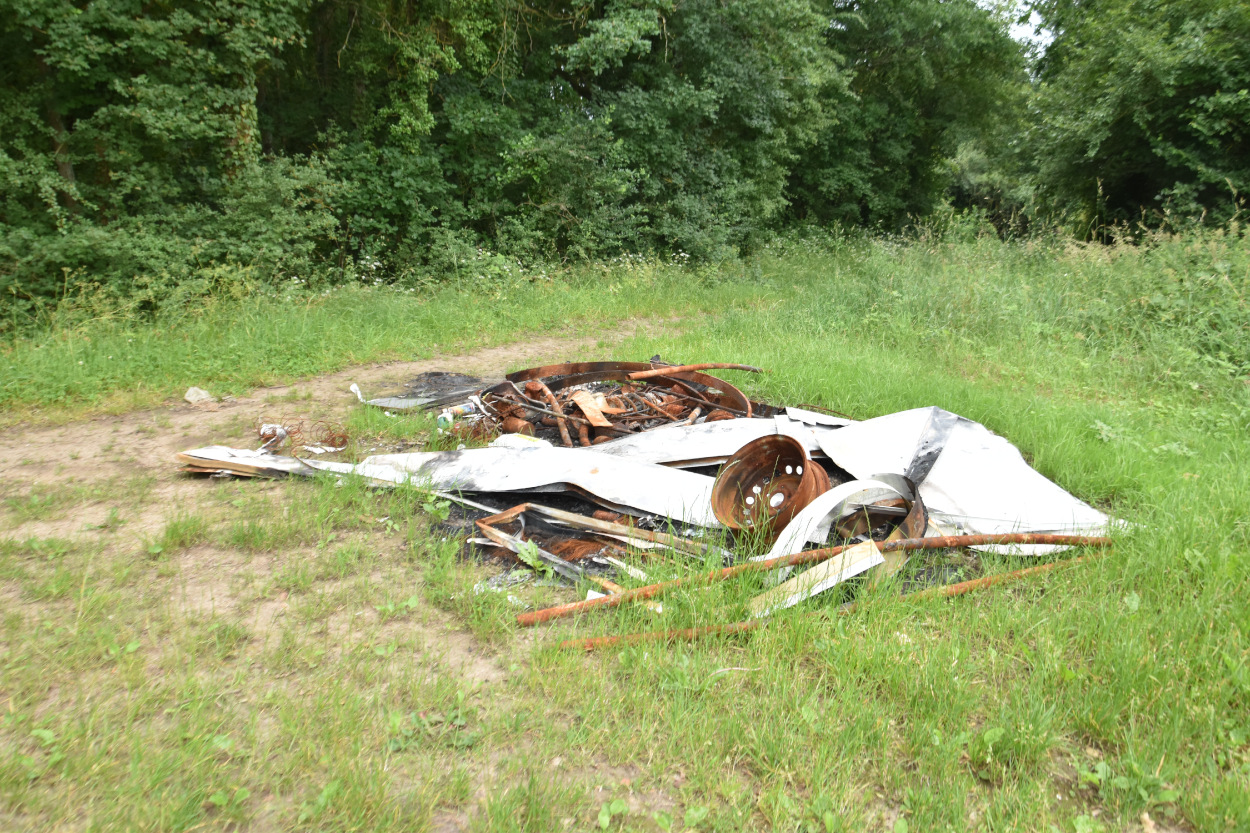

Supplement: S1 Data — (ZIP) [file pone.0234210.s002.zip › Pictures_DataBase_Environment/Polluted/Polluted - Rural/Polluted - Rural - Without Individuals/Rur.SALE11.1.JPG]

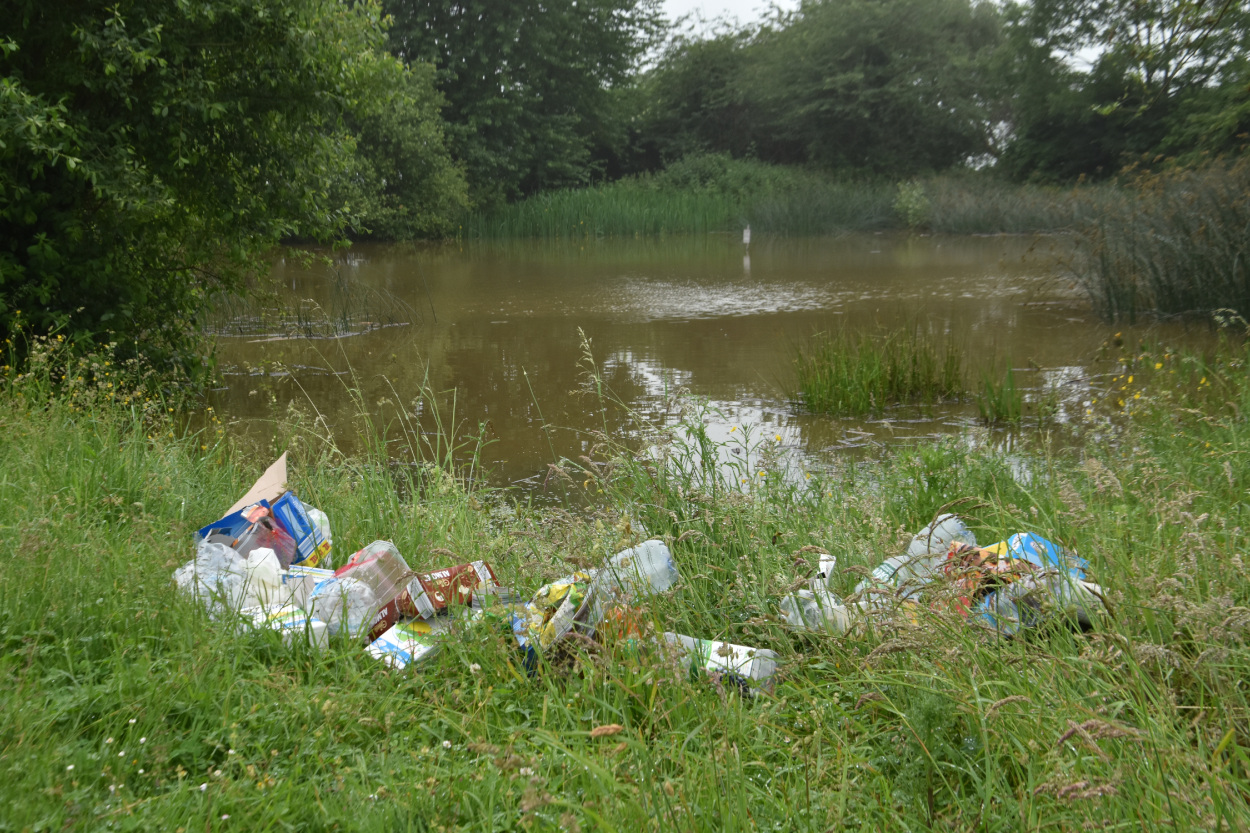

Supplement: S1 Data — (ZIP) [file pone.0234210.s002.zip › Pictures_DataBase_Environment/Polluted/Polluted - Rural/Polluted - Rural - Without Individuals/Rur.SALE13.1.JPG]

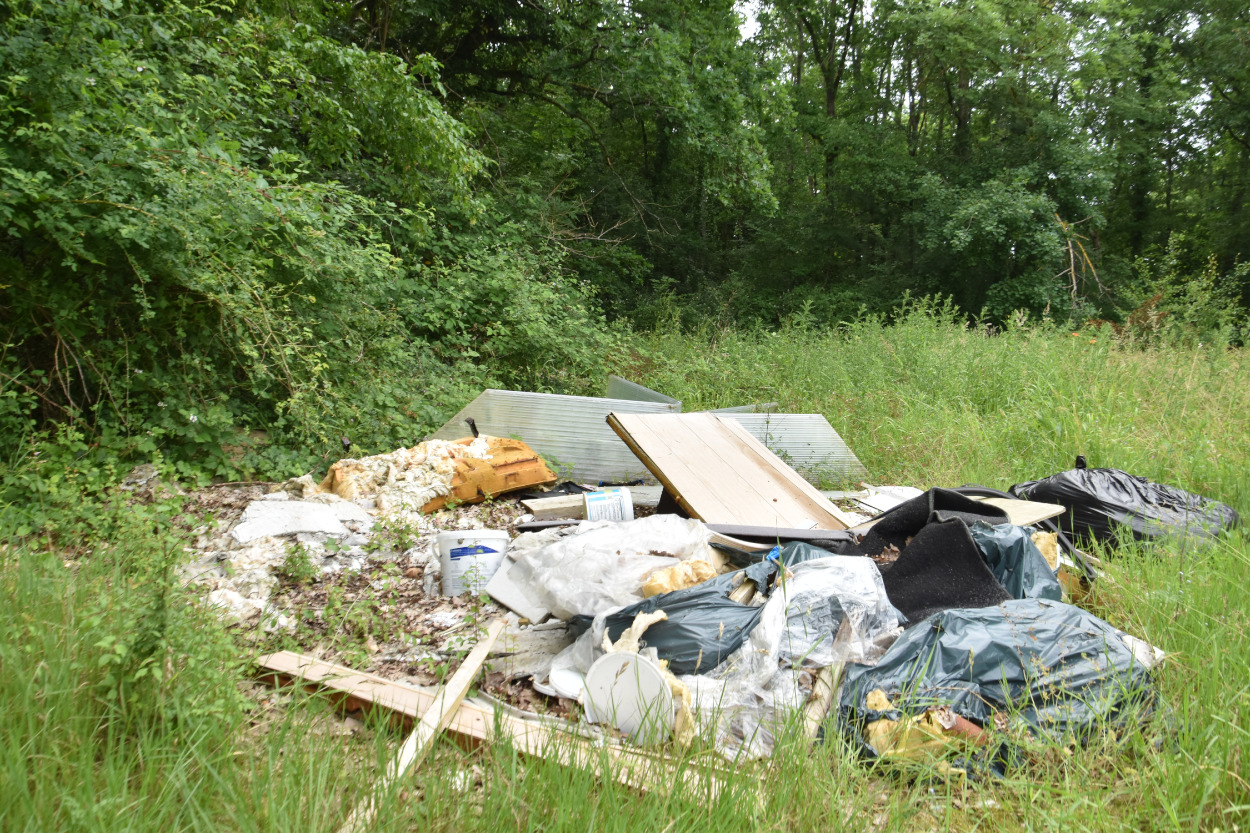

Supplement: S1 Data — (ZIP) [file pone.0234210.s002.zip › Pictures_DataBase_Environment/Polluted/Polluted - Rural/Polluted - Rural - Without Individuals/Rur.SALE06.1.JPG]

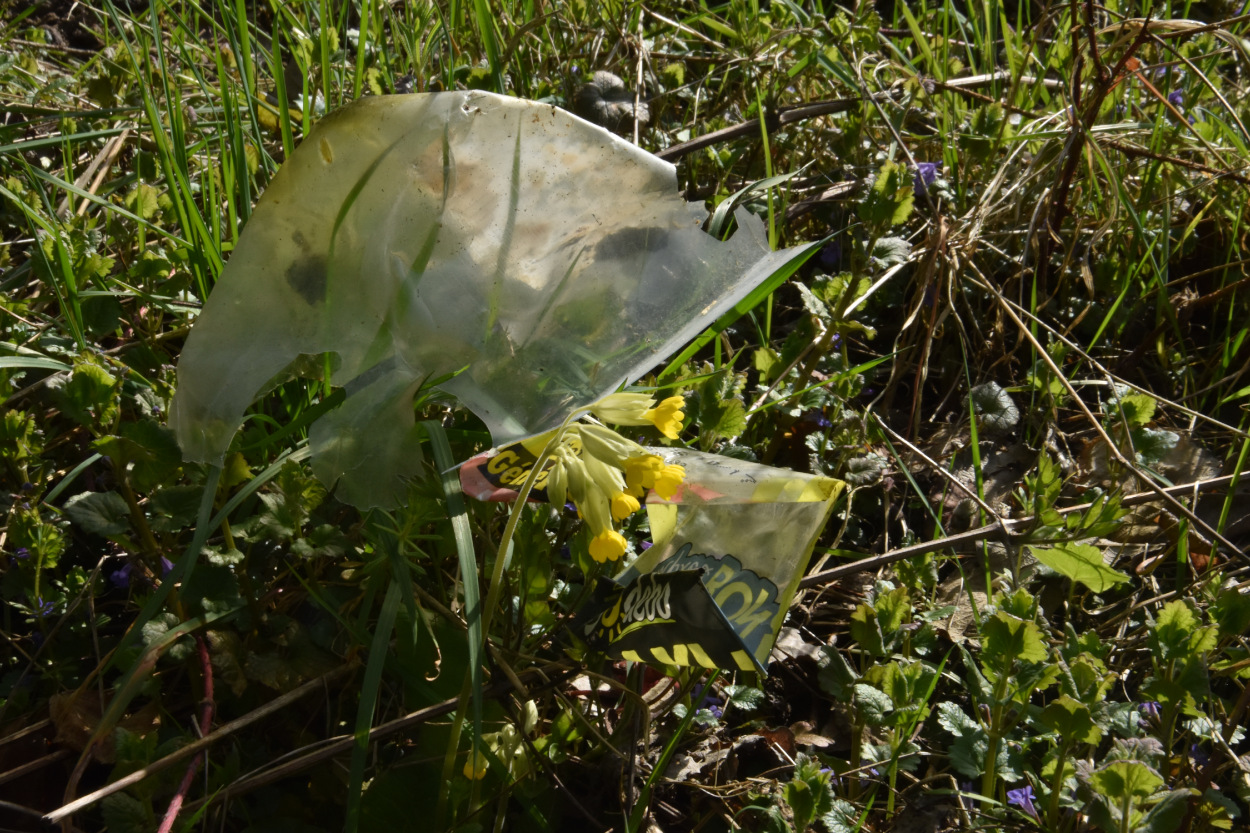

Supplement: S1 Data — (ZIP) [file pone.0234210.s002.zip › Pictures_DataBase_Environment/Polluted/Polluted - Rural/Polluted - Rural - Without Individuals/Rur.SALE22.1.JPG]

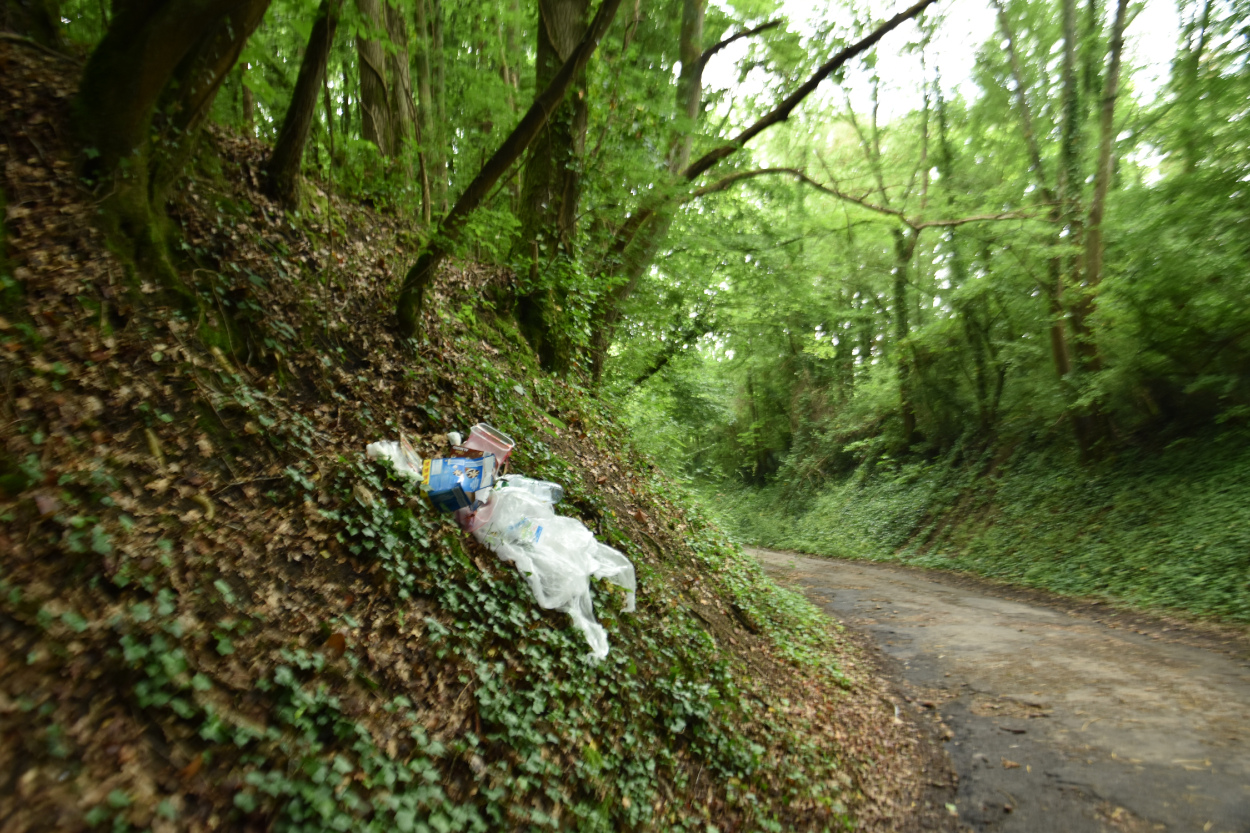

Supplement: S1 Data — (ZIP) [file pone.0234210.s002.zip › Pictures_DataBase_Environment/Polluted/Polluted - Rural/Polluted - Rural - Without Individuals/Rur.SALE20.1.JPG]

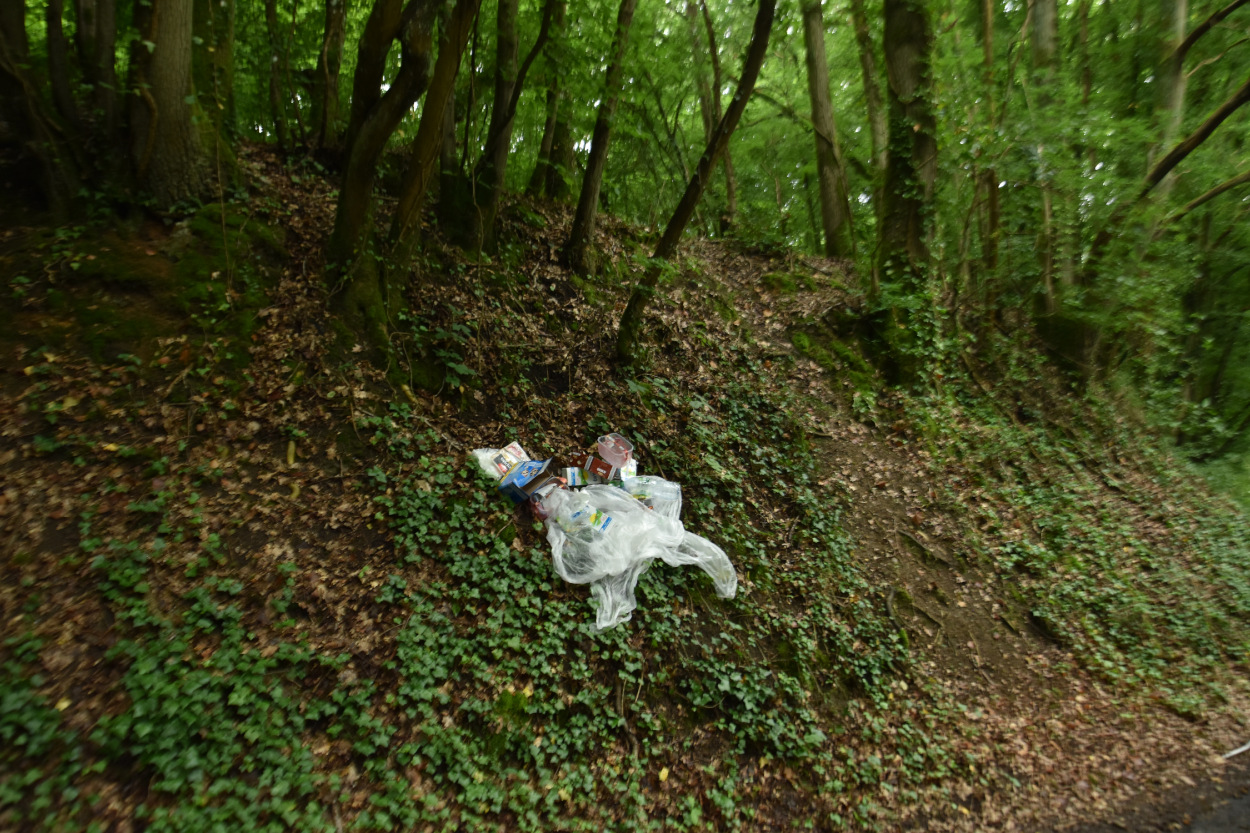

Supplement: S1 Data — (ZIP) [file pone.0234210.s002.zip › Pictures_DataBase_Environment/Polluted/Polluted - Rural/Polluted - Rural - Without Individuals/Rur.SALE19.1.JPG]

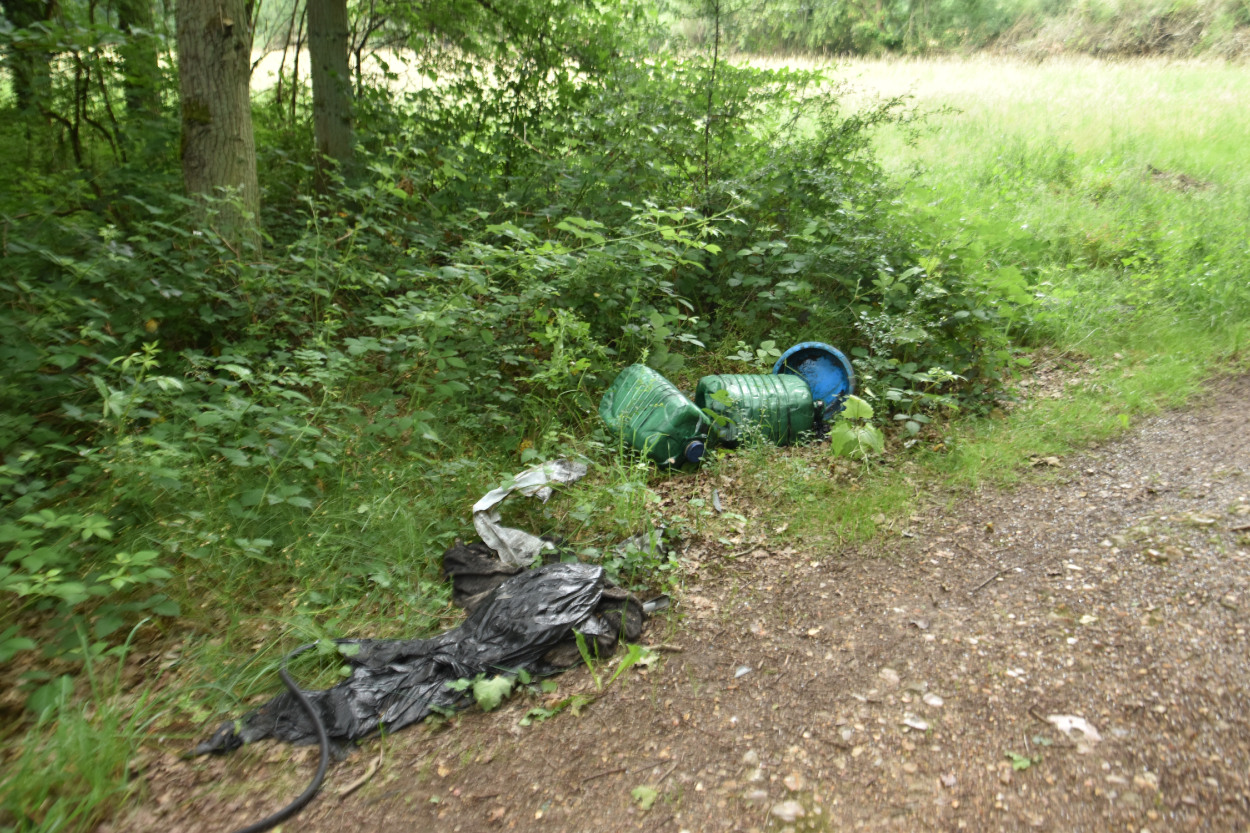

Supplement: S1 Data — (ZIP) [file pone.0234210.s002.zip › Pictures_DataBase_Environment/Polluted/Polluted - Rural/Polluted - Rural - Without Individuals/Rur.SALE02.1.JPG]

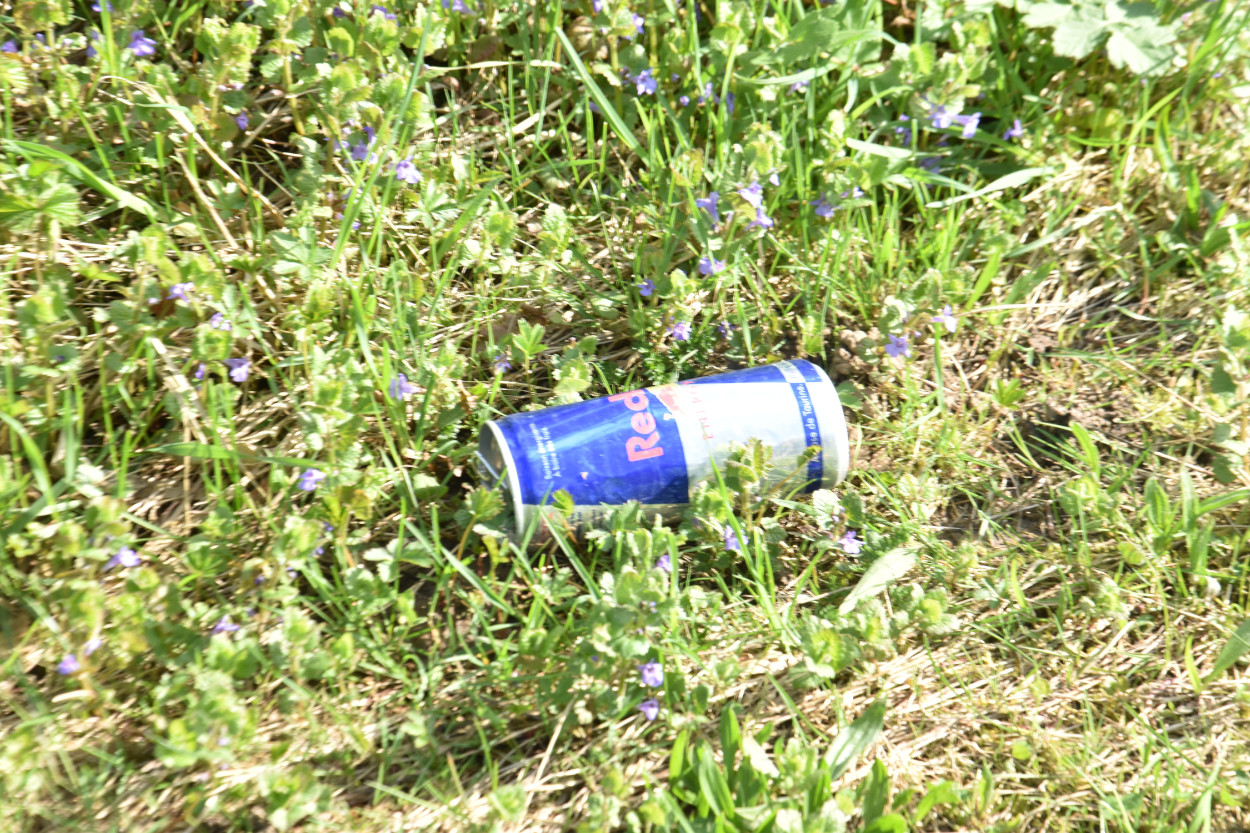

Supplement: S1 Data — (ZIP) [file pone.0234210.s002.zip › Pictures_DataBase_Environment/Polluted/Polluted - Rural/Polluted - Rural - Without Individuals/Rur.SALE21.1.JPG]

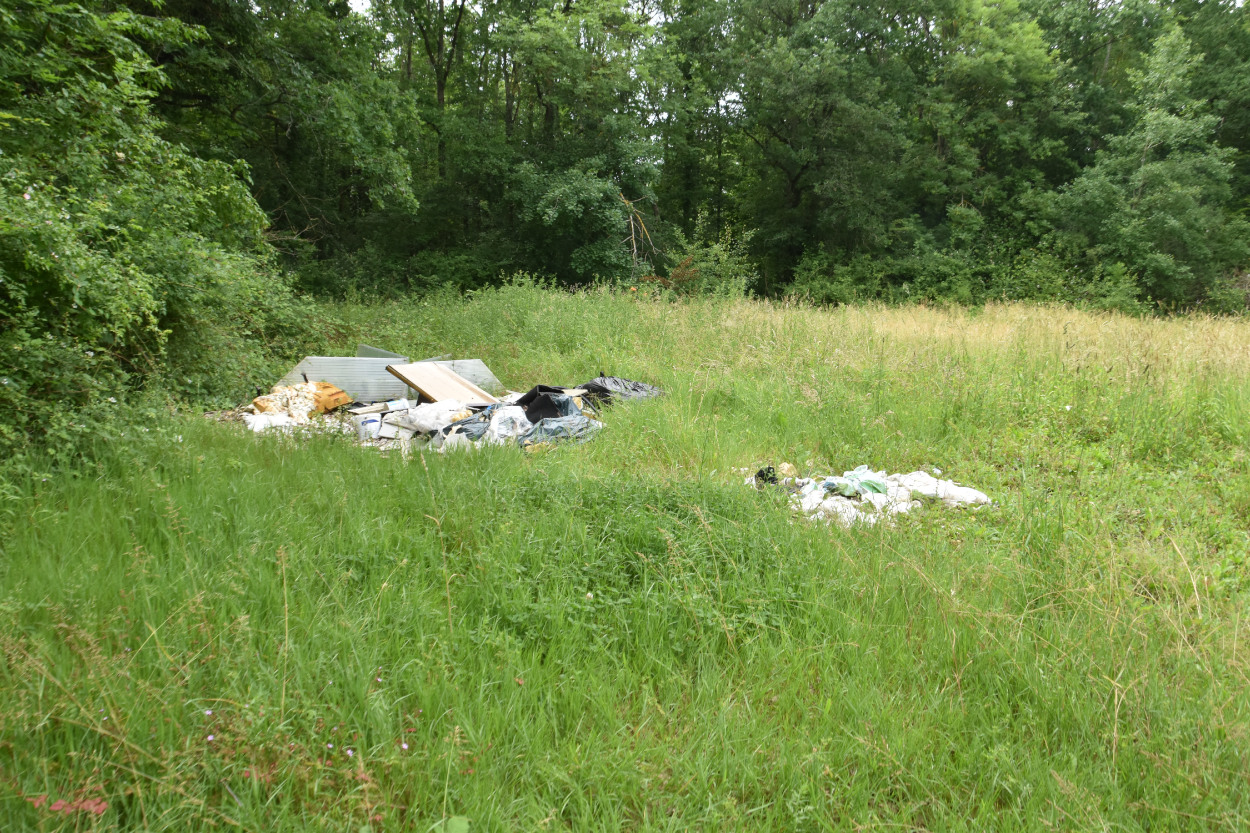

Supplement: S1 Data — (ZIP) [file pone.0234210.s002.zip › Pictures_DataBase_Environment/Polluted/Polluted - Rural/Polluted - Rural - Without Individuals/Rur.SALE05.1.JPG]

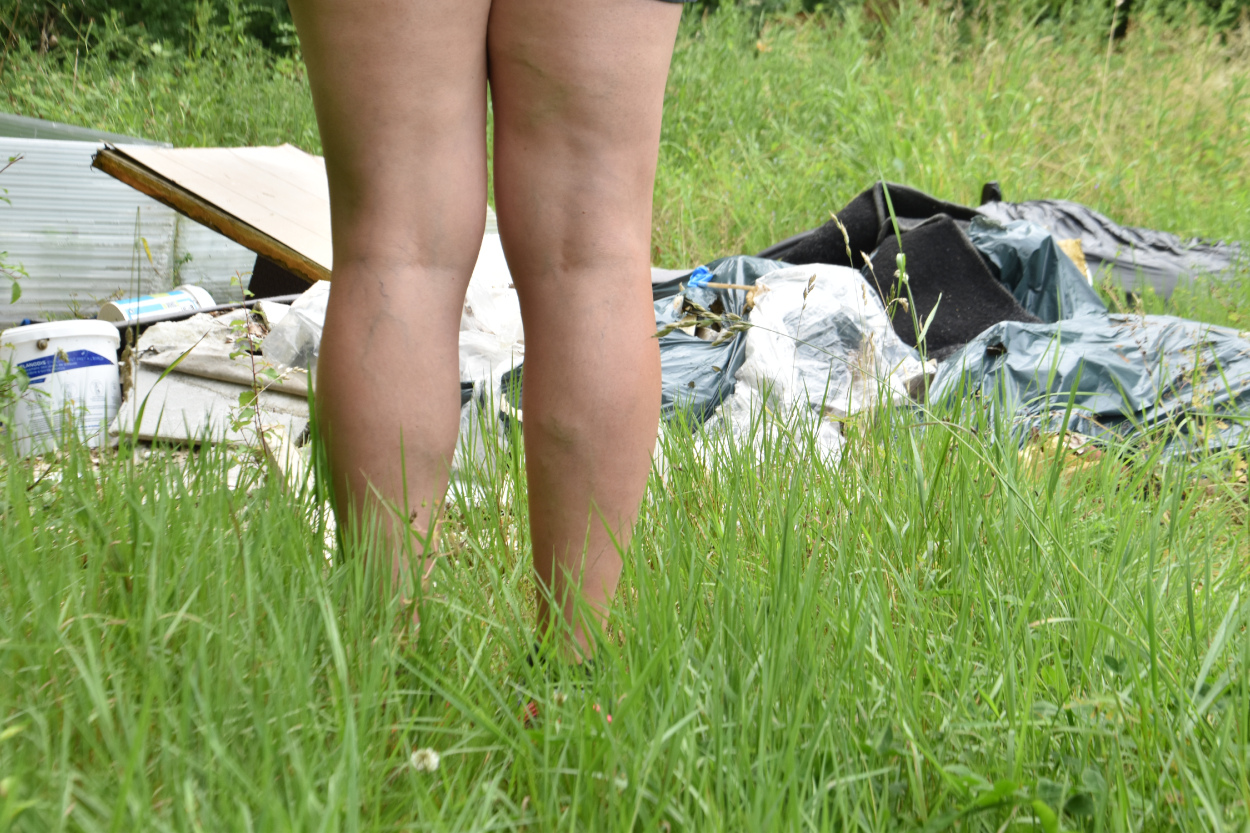

Supplement: S1 Data — (ZIP) [file pone.0234210.s002.zip › Pictures_DataBase_Environment/Polluted/Polluted - Rural/Polluted - Rural - With Individuals/Rur.Pers.SALE02.1.JPG]

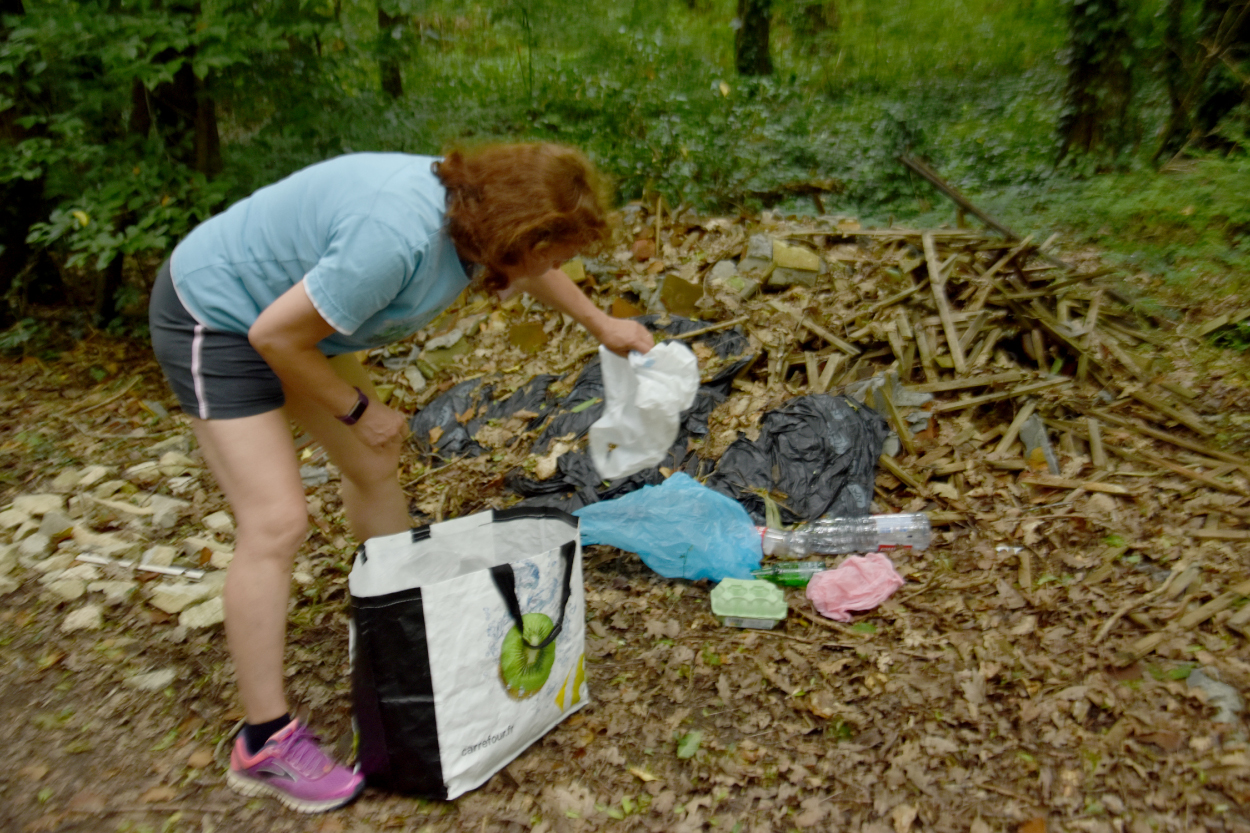

Supplement: S1 Data — (ZIP) [file pone.0234210.s002.zip › Pictures_DataBase_Environment/Polluted/Polluted - Rural/Polluted - Rural - With Individuals/Rur.Pers.SALE04.1.JPG]

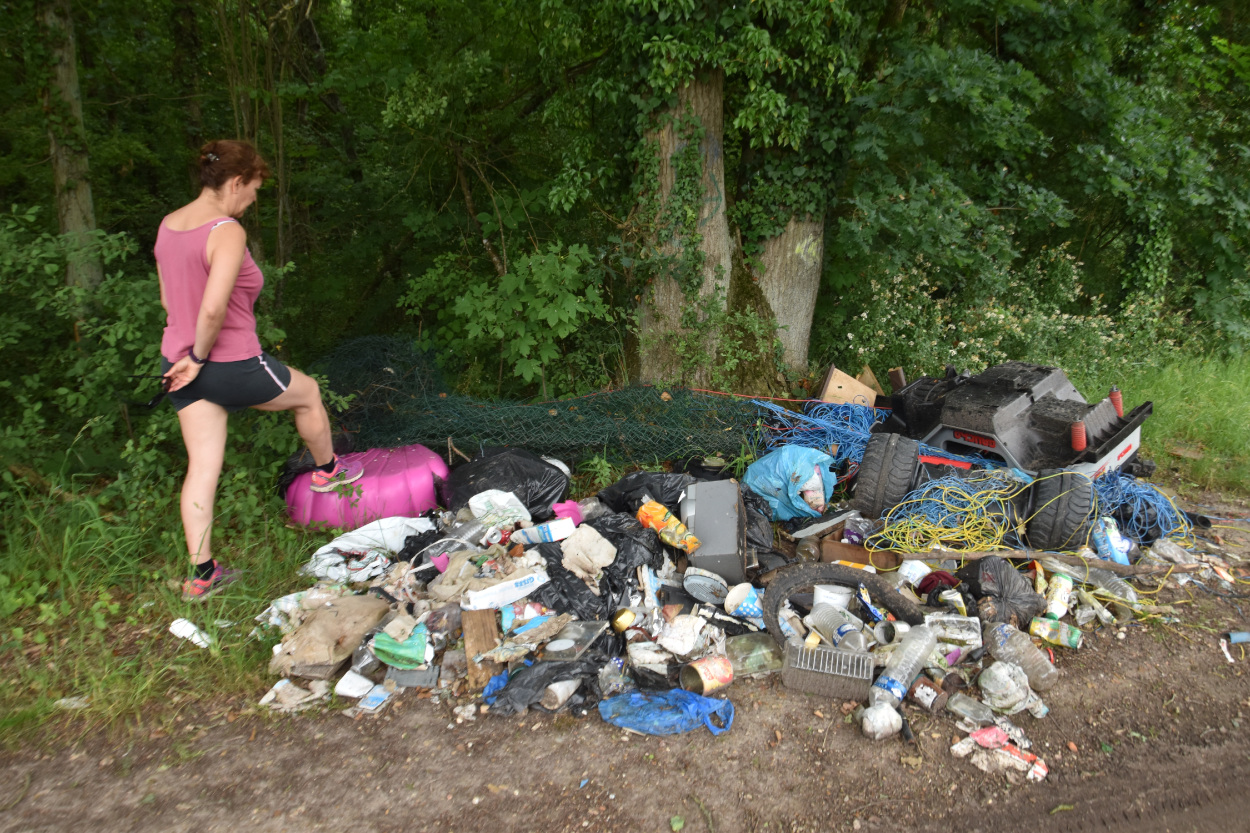

Supplement: S1 Data — (ZIP) [file pone.0234210.s002.zip › Pictures_DataBase_Environment/Polluted/Polluted - Rural/Polluted - Rural - With Individuals/Rur.Pers.SALE06.1.JPG]

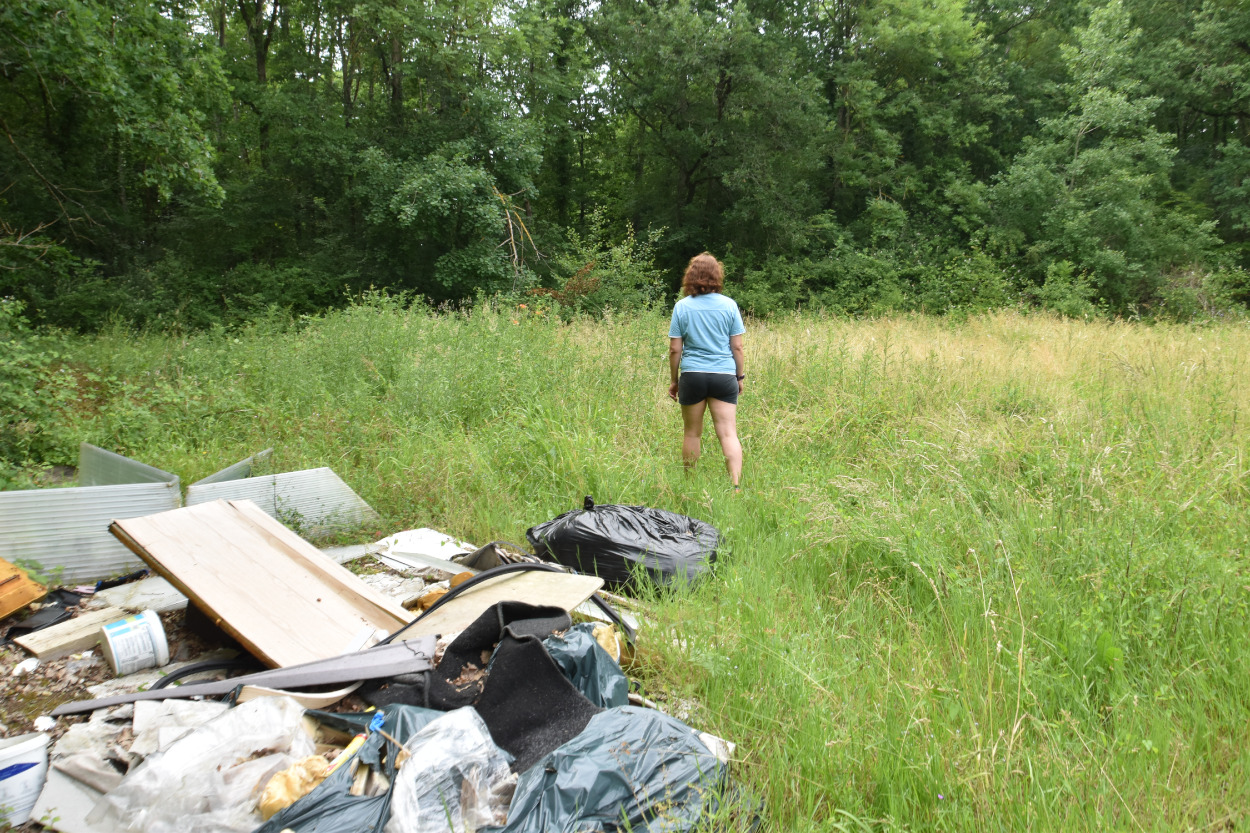

Supplement: S1 Data — (ZIP) [file pone.0234210.s002.zip › Pictures_DataBase_Environment/Polluted/Polluted - Rural/Polluted - Rural - With Individuals/Rur.Pers.SALE03.1.JPG]

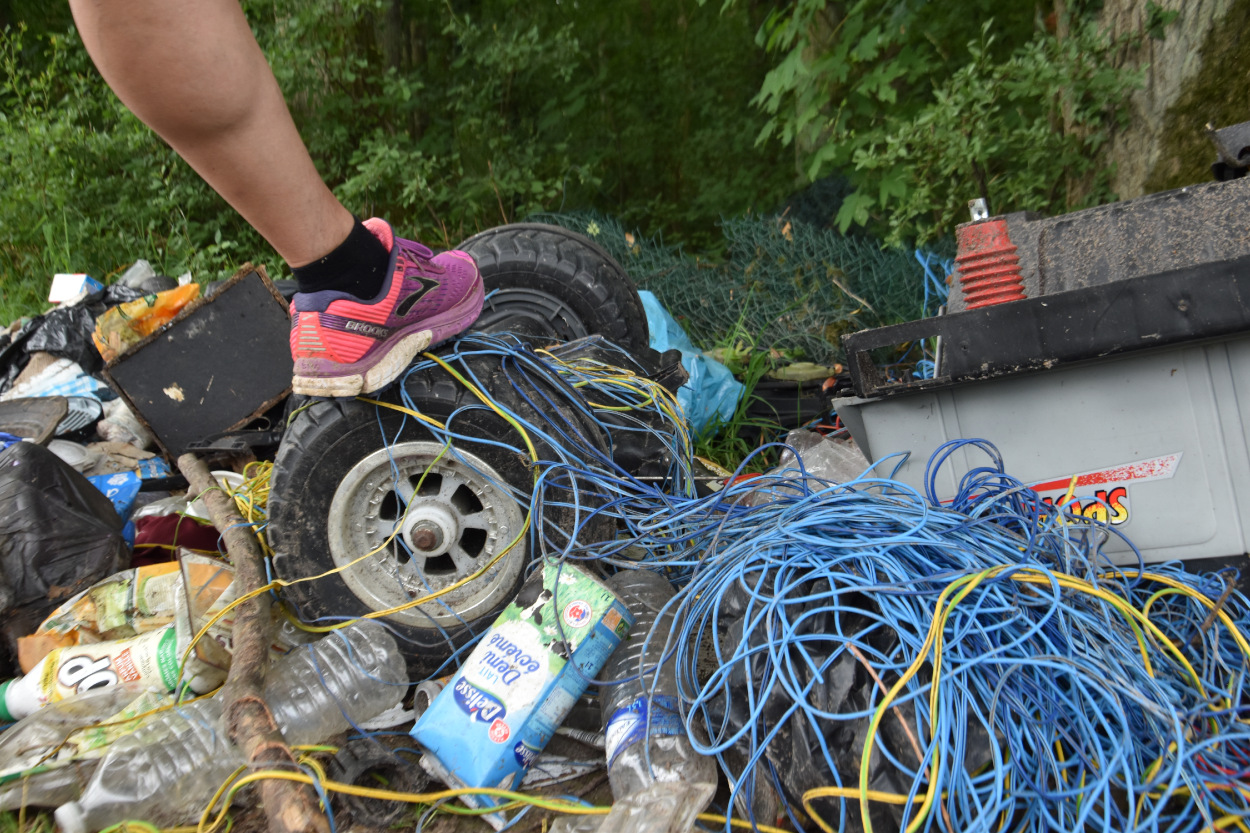

Supplement: S1 Data — (ZIP) [file pone.0234210.s002.zip › Pictures_DataBase_Environment/Polluted/Polluted - Rural/Polluted - Rural - With Individuals/Rur.Pers.SALE07.1.JPG]

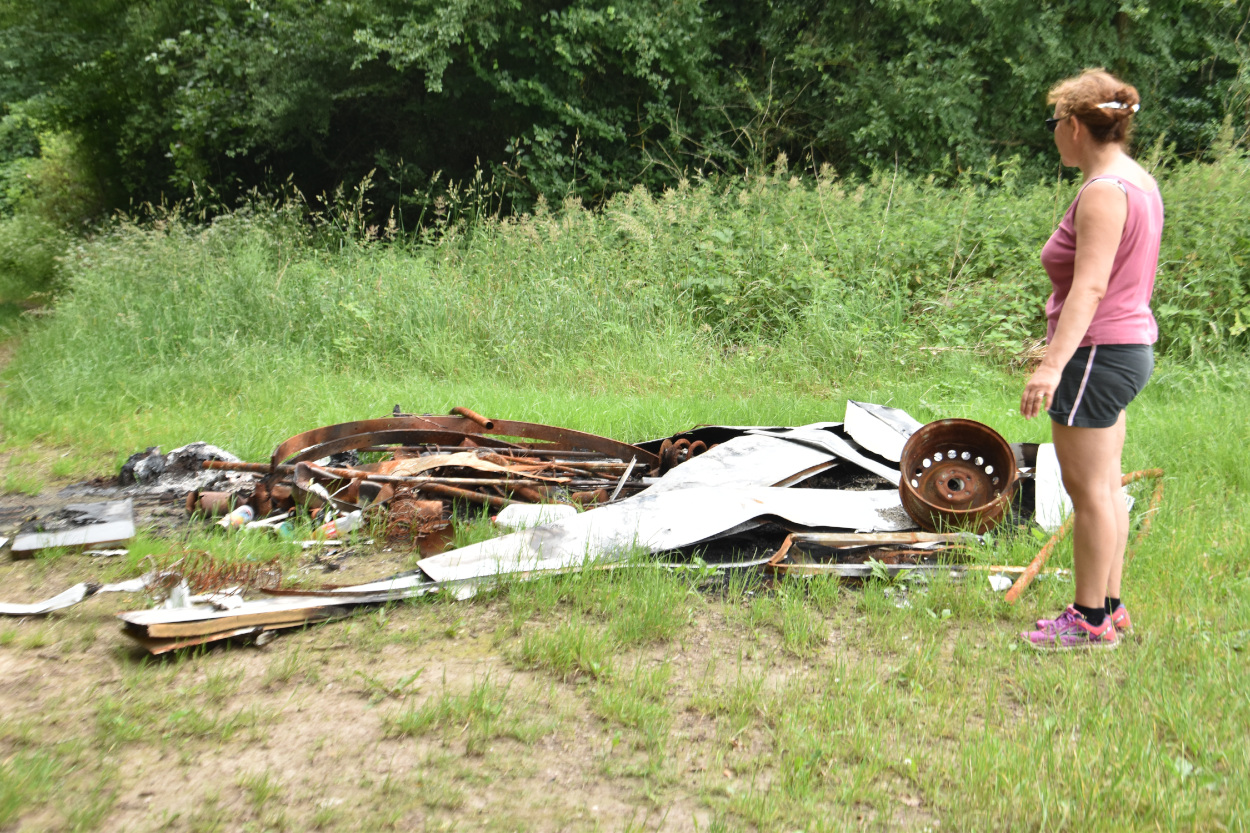

Supplement: S1 Data — (ZIP) [file pone.0234210.s002.zip › Pictures_DataBase_Environment/Polluted/Polluted - Rural/Polluted - Rural - With Individuals/Rur.Pers.SALE05.1.JPG]

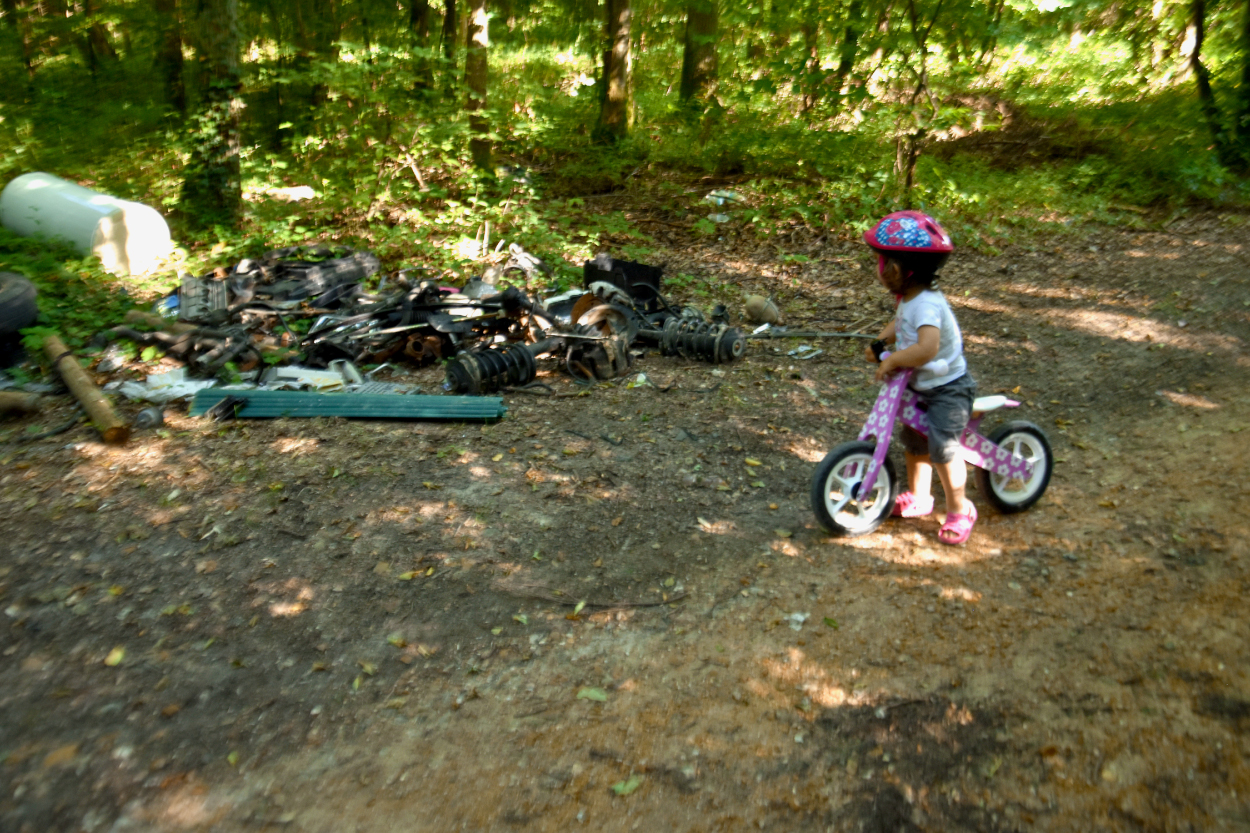

Supplement: S1 Data — (ZIP) [file pone.0234210.s002.zip › Pictures_DataBase_Environment/Polluted/Polluted - Rural/Polluted - Rural - With Individuals/Rur.Pers.SALE10.1.jpg]

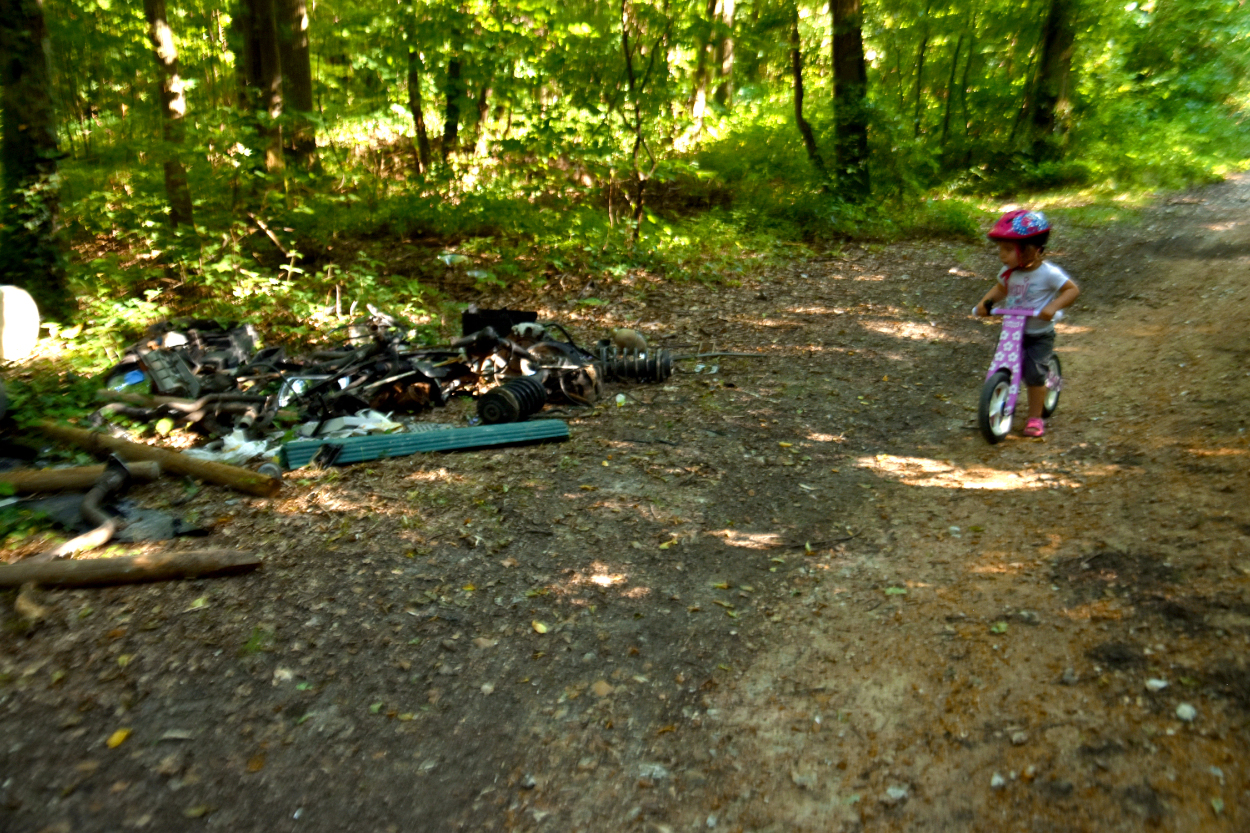

Supplement: S1 Data — (ZIP) [file pone.0234210.s002.zip › Pictures_DataBase_Environment/Polluted/Polluted - Rural/Polluted - Rural - With Individuals/Rur.Pers.SALE09.1.jpg]

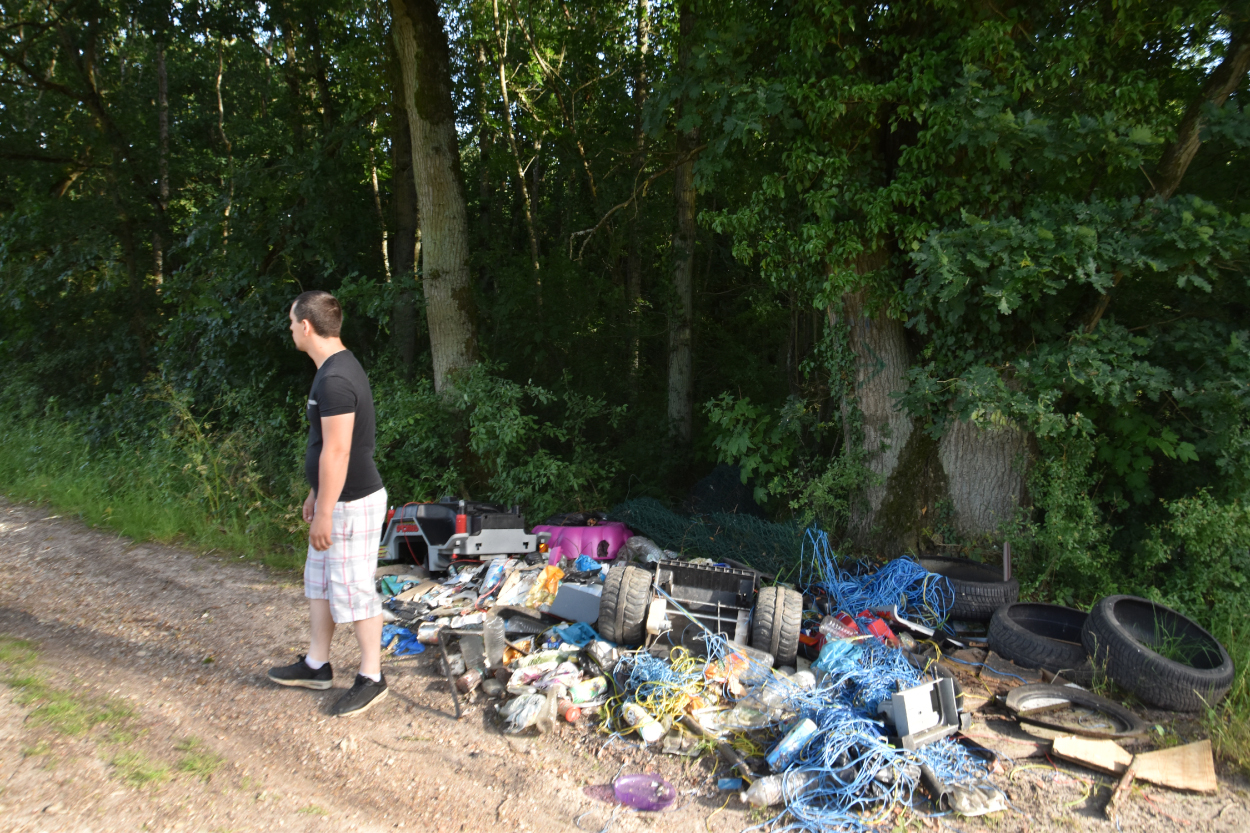

Supplement: S1 Data — (ZIP) [file pone.0234210.s002.zip › Pictures_DataBase_Environment/Polluted/Polluted - Rural/Polluted - Rural - With Individuals/Rur.Pers.SALE16.1.jpg]

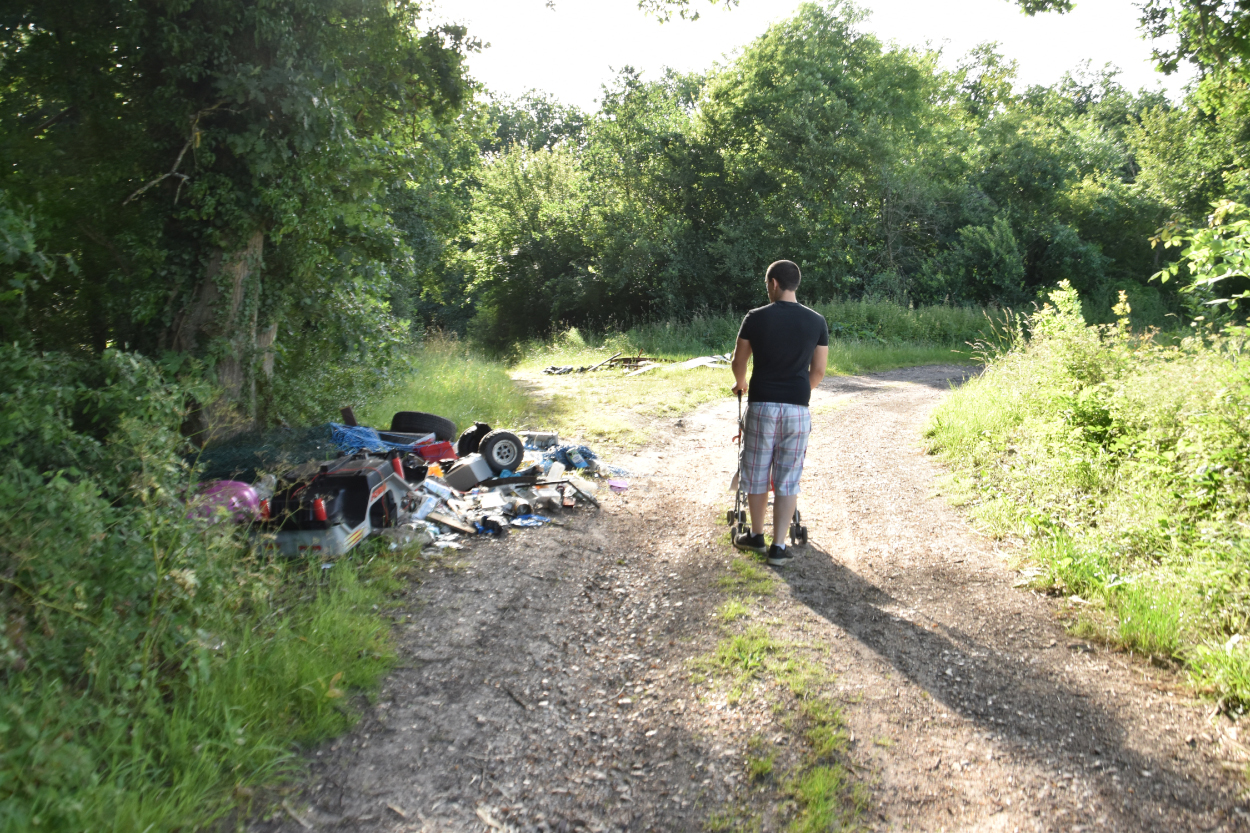

Supplement: S1 Data — (ZIP) [file pone.0234210.s002.zip › Pictures_DataBase_Environment/Polluted/Polluted - Rural/Polluted - Rural - With Individuals/Rur.Pers.SALE14.1.jpg]

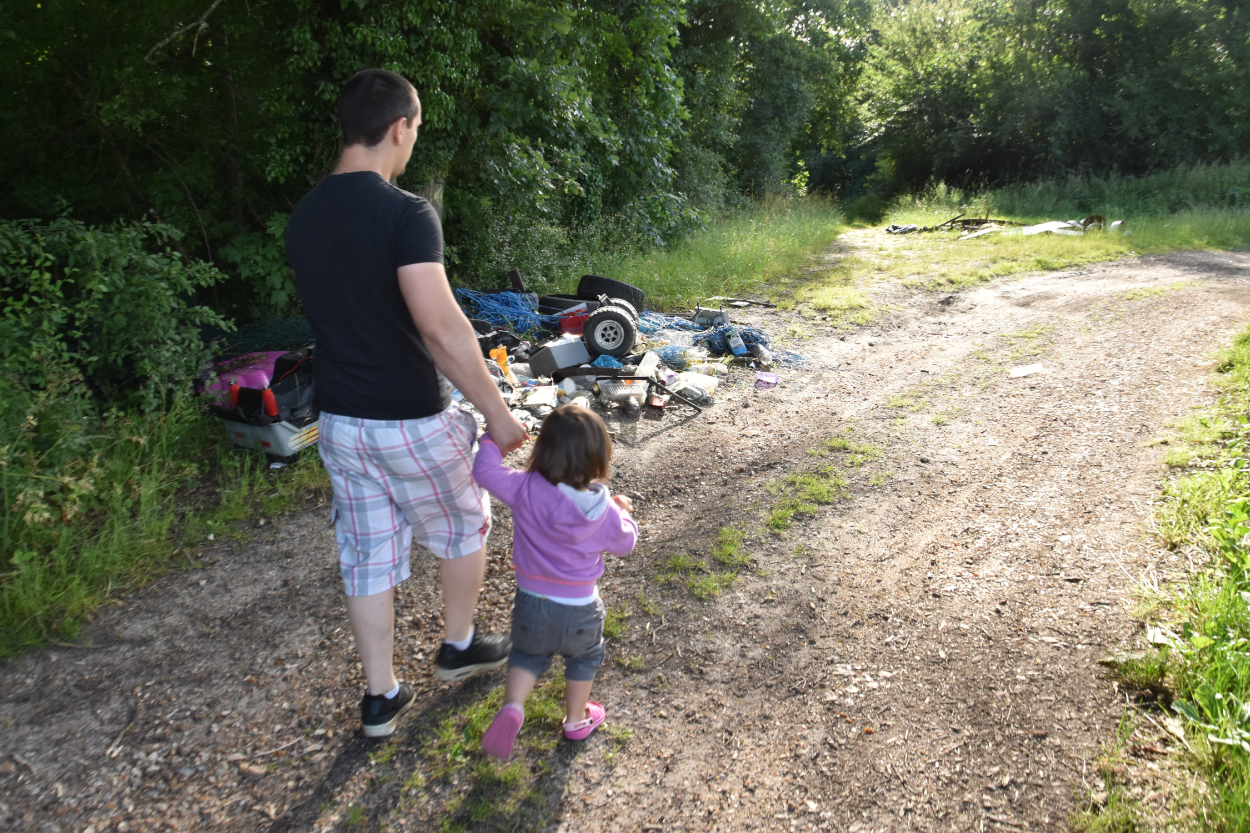

Supplement: S1 Data — (ZIP) [file pone.0234210.s002.zip › Pictures_DataBase_Environment/Polluted/Polluted - Rural/Polluted - Rural - With Individuals/Rur.Pers.SALE15.1.jpg]

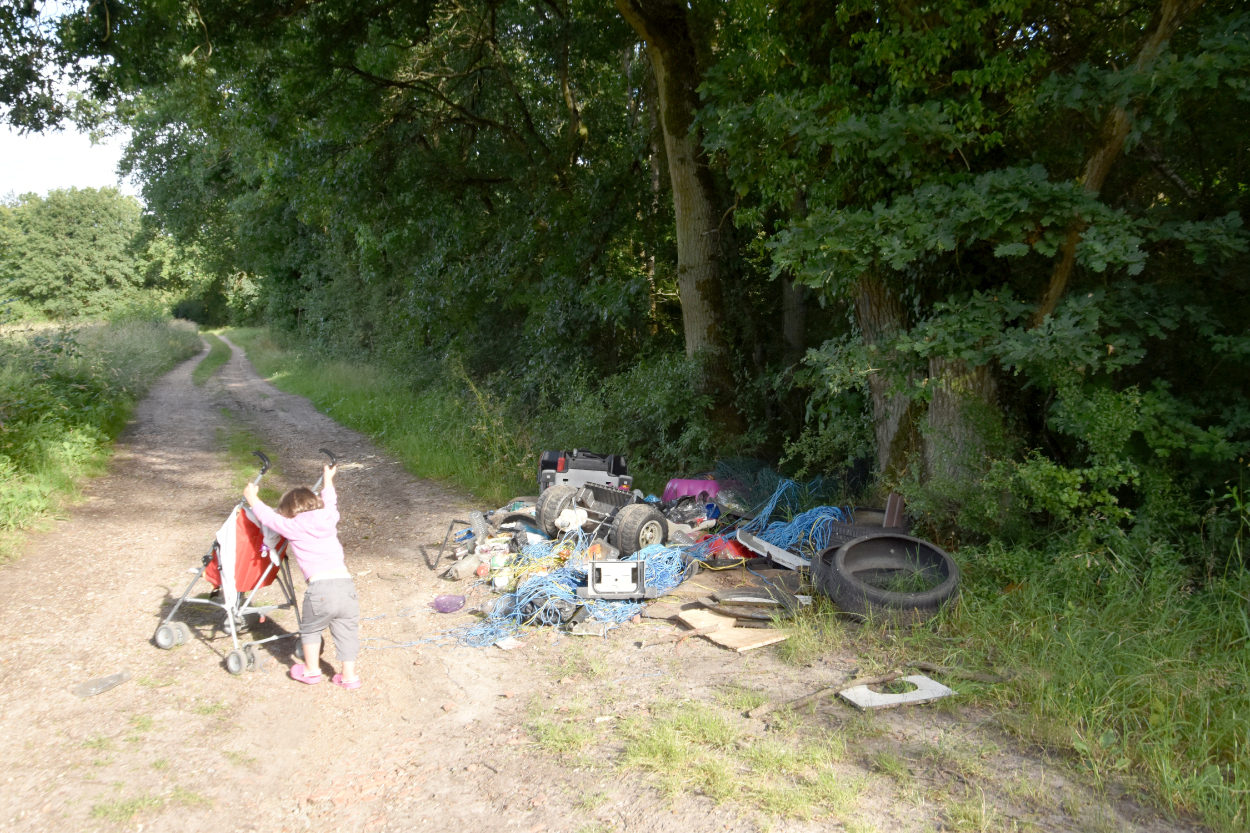

Supplement: S1 Data — (ZIP) [file pone.0234210.s002.zip › Pictures_DataBase_Environment/Polluted/Polluted - Rural/Polluted - Rural - With Individuals/Rur.Pers.SALE17.1.jpg]

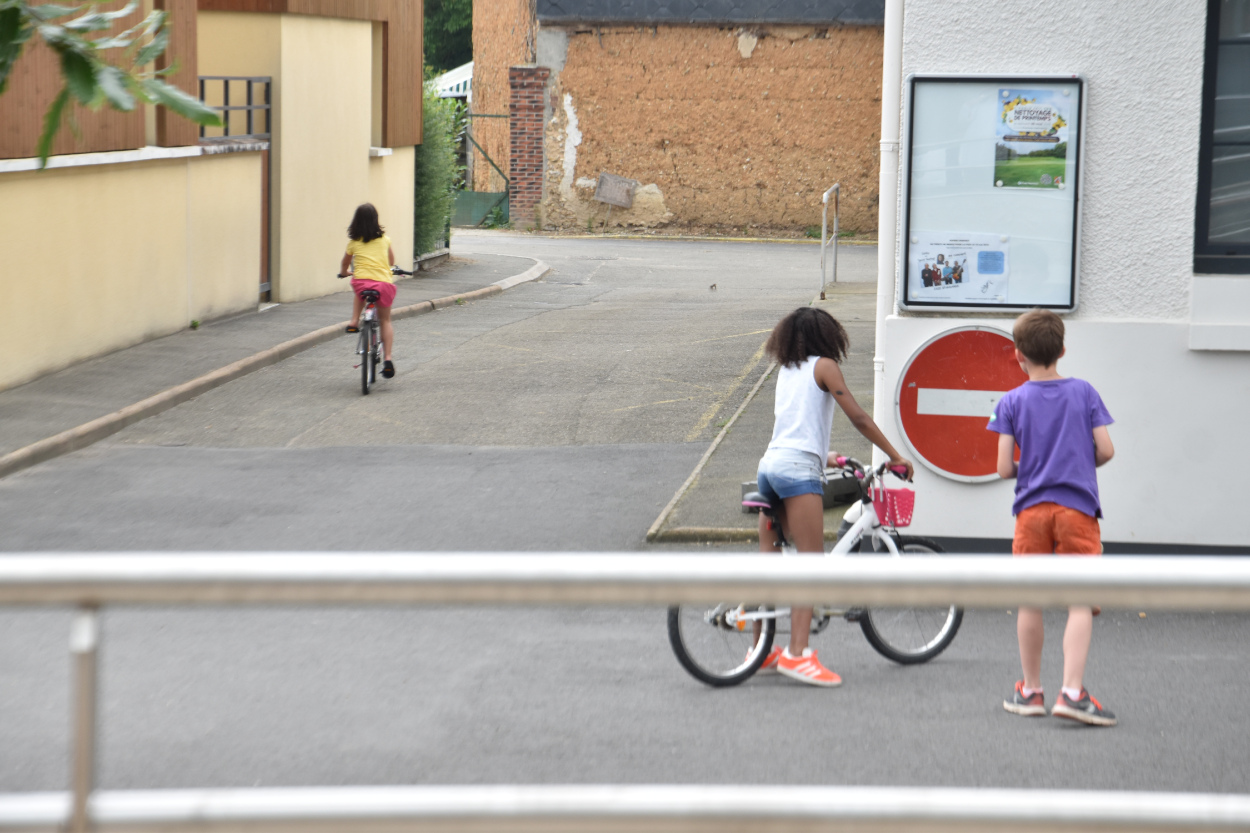

Supplement: S1 Data — (ZIP) [file pone.0234210.s002.zip › Pictures_DataBase_Environment/Clean/Clean - Urban/Clean - Urban - With Individuals/Urb.Pers.PROPRE03.1.JPG]

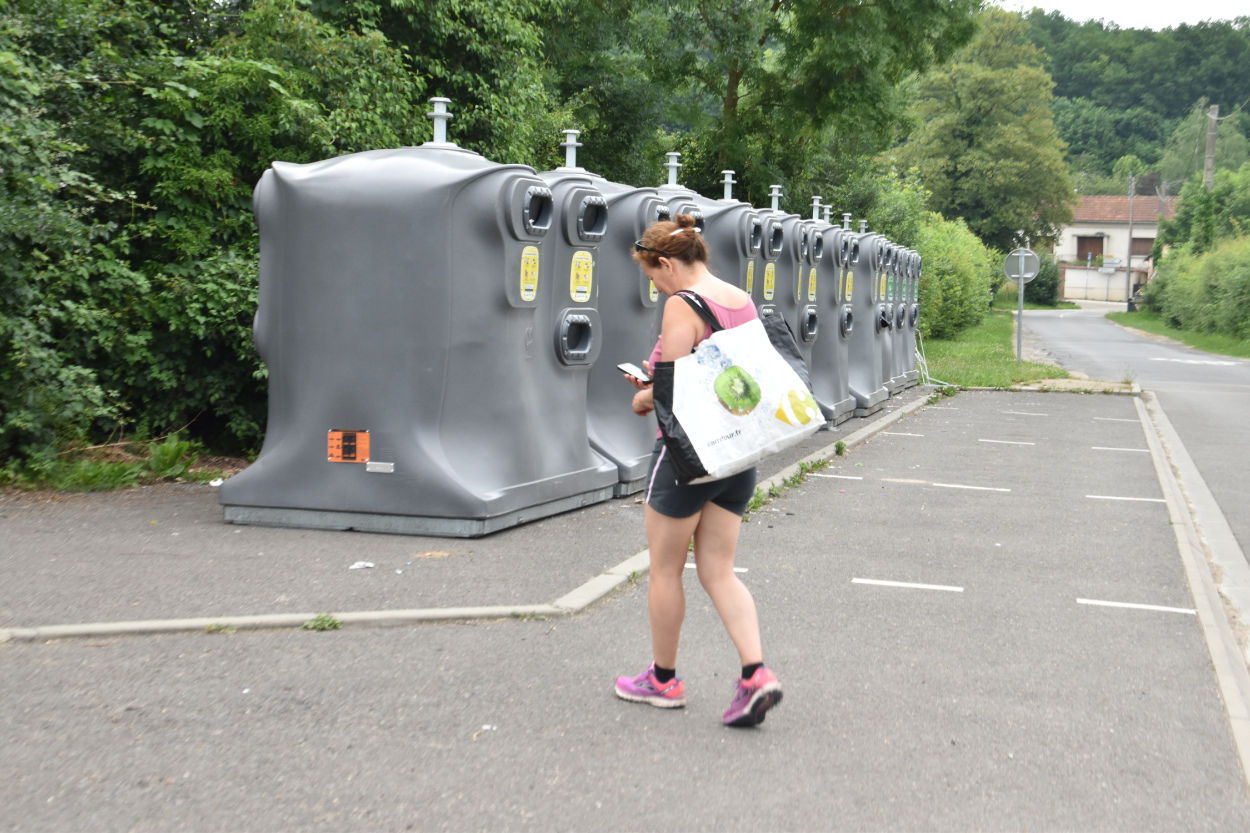

Supplement: S1 Data — (ZIP) [file pone.0234210.s002.zip › Pictures_DataBase_Environment/Clean/Clean - Urban/Clean - Urban - With Individuals/Urb.Pers.PROPRE01.1.JPG]

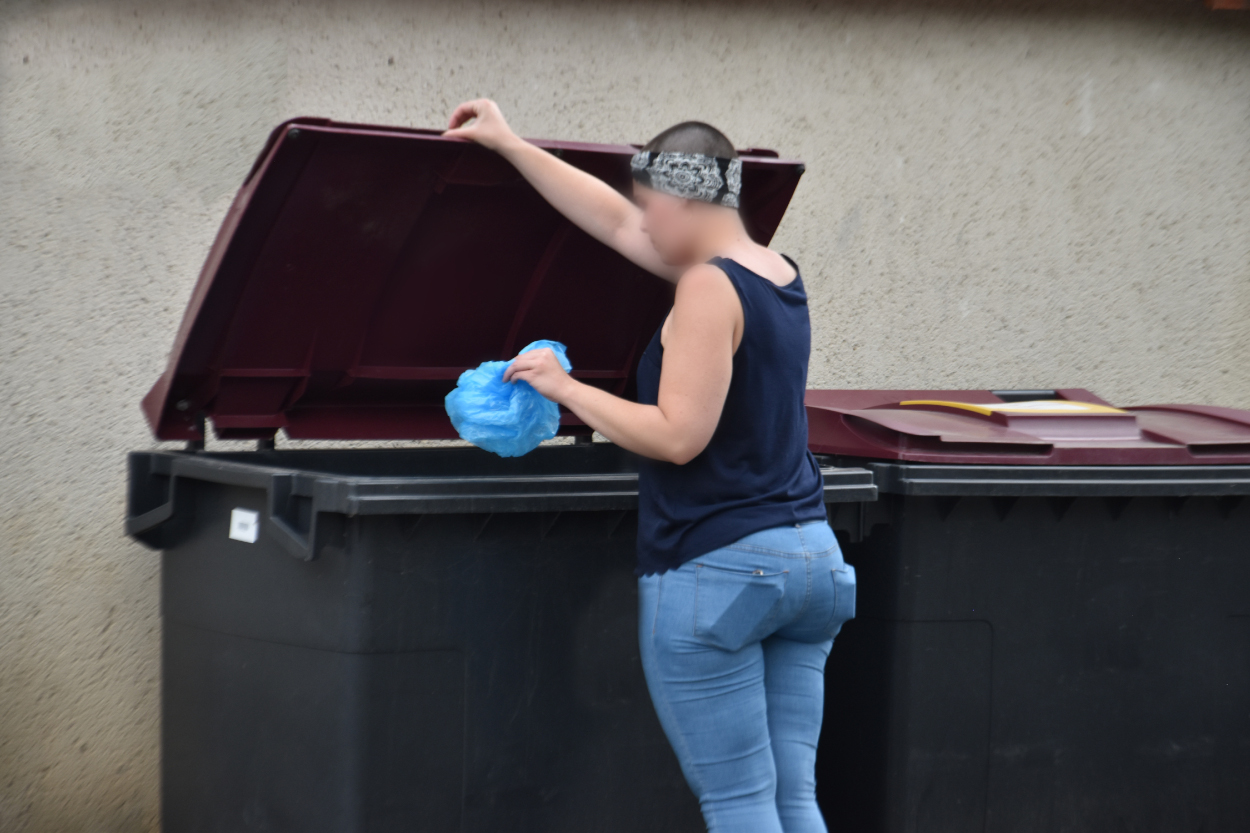

Supplement: S1 Data — (ZIP) [file pone.0234210.s002.zip › Pictures_DataBase_Environment/Clean/Clean - Urban/Clean - Urban - With Individuals/Urb.Pers.PROPRE05.1.JPG]

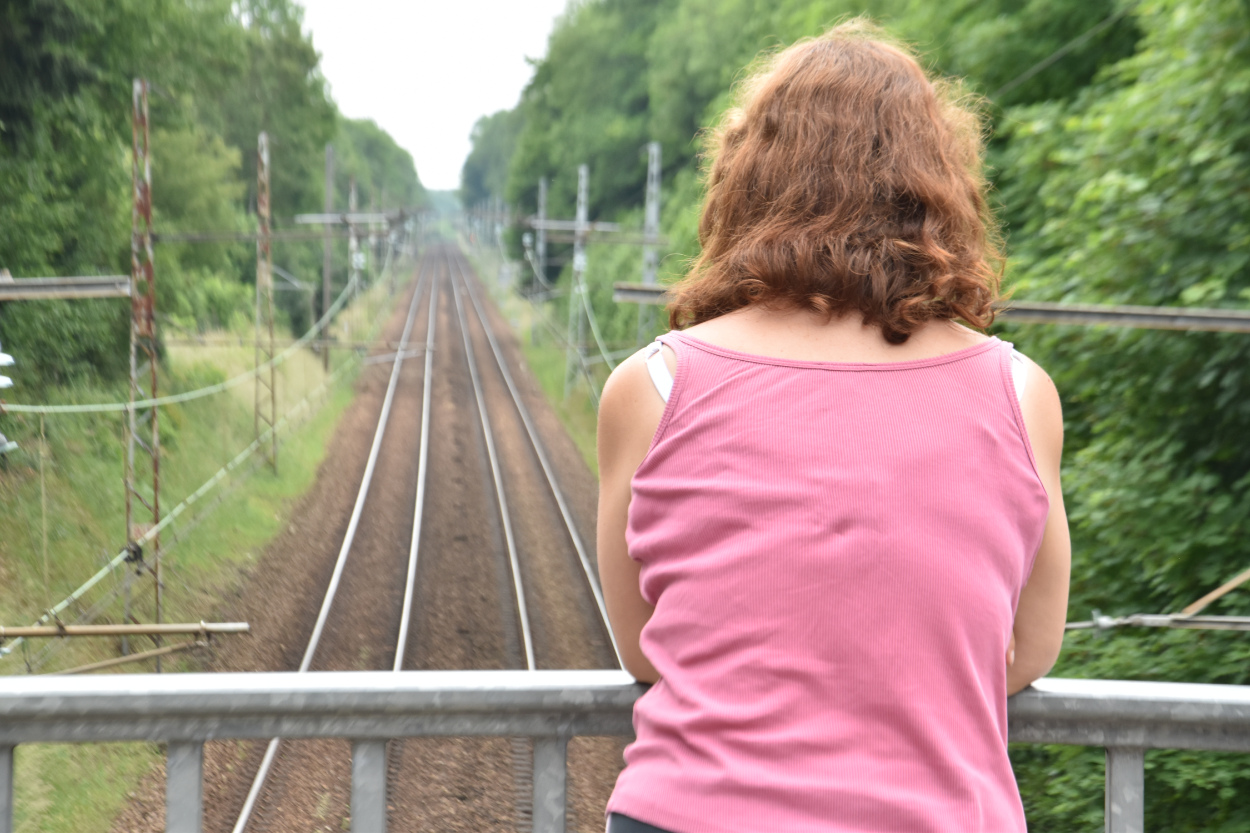

Supplement: S1 Data — (ZIP) [file pone.0234210.s002.zip › Pictures_DataBase_Environment/Clean/Clean - Urban/Clean - Urban - With Individuals/Urb.Pers.PROPRE07.1.JPG]

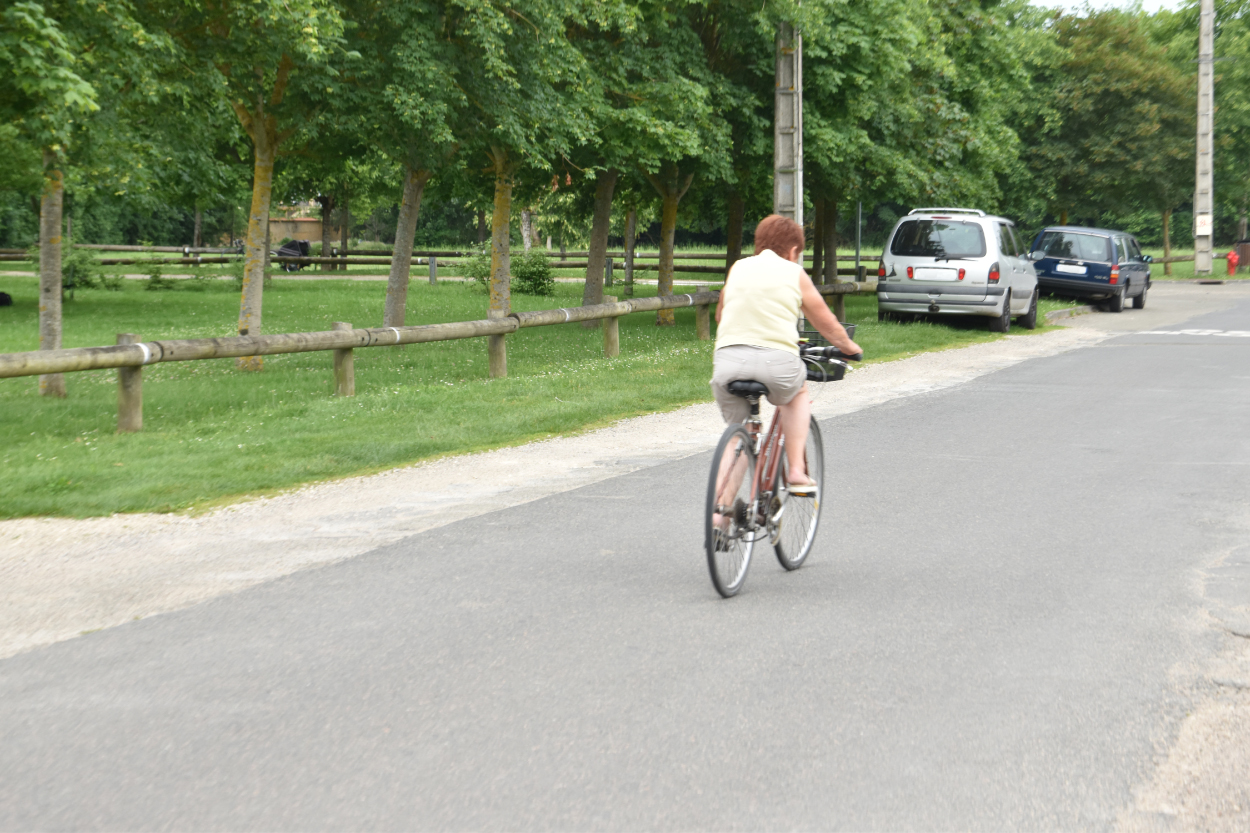

Supplement: S1 Data — (ZIP) [file pone.0234210.s002.zip › Pictures_DataBase_Environment/Clean/Clean - Urban/Clean - Urban - With Individuals/Urb.Pers.PROPRE02.1.JPG]

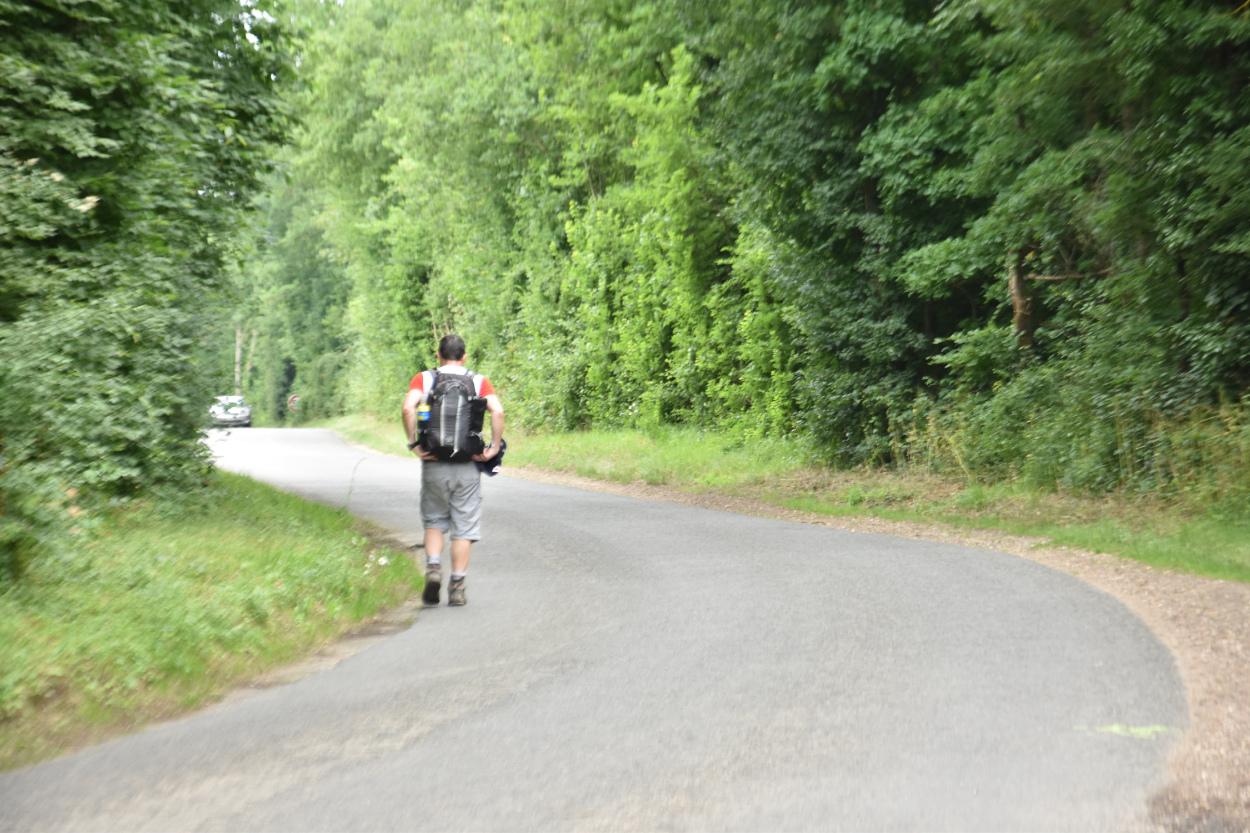

Supplement: S1 Data — (ZIP) [file pone.0234210.s002.zip › Pictures_DataBase_Environment/Clean/Clean - Urban/Clean - Urban - With Individuals/Urb.Pers.PROPRE06.1.JPG]

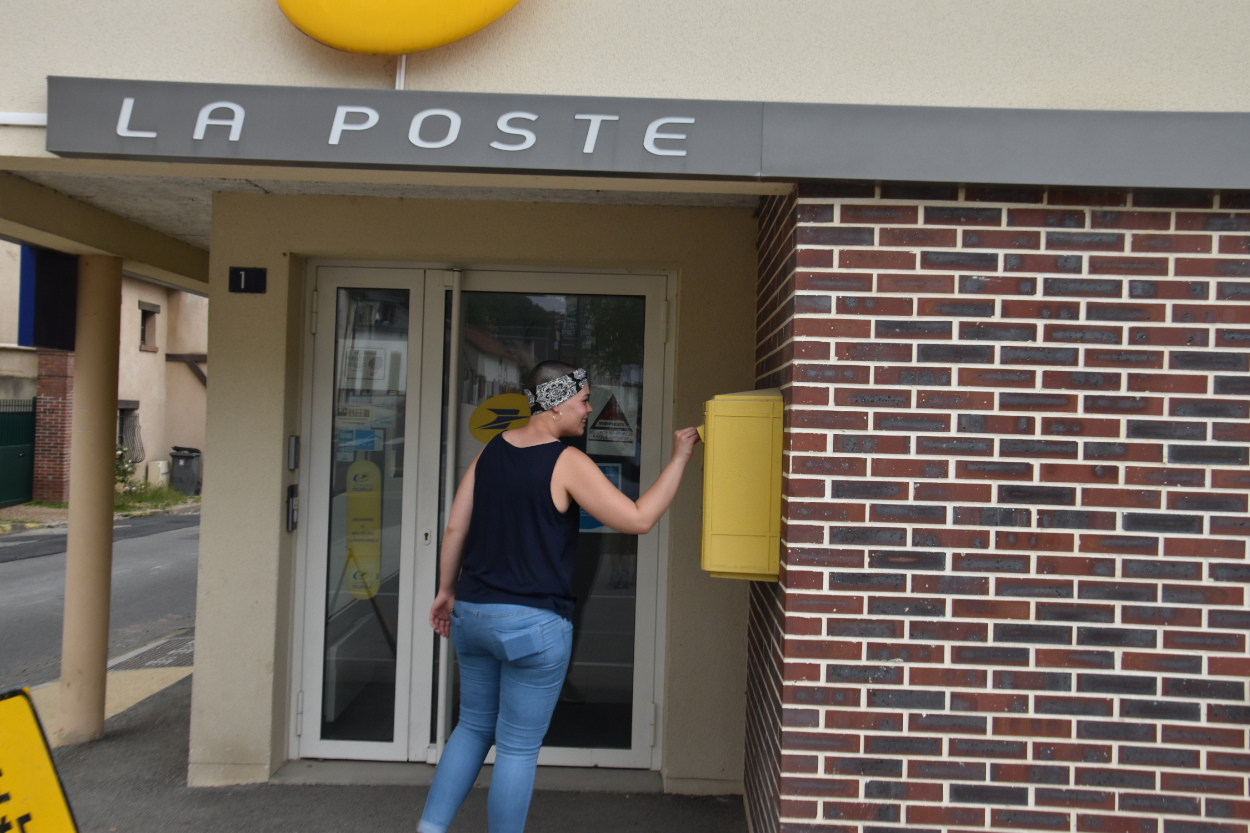

Supplement: S1 Data — (ZIP) [file pone.0234210.s002.zip › Pictures_DataBase_Environment/Clean/Clean - Urban/Clean - Urban - With Individuals/Urb.Pers.PROPRE04.1.JPG]

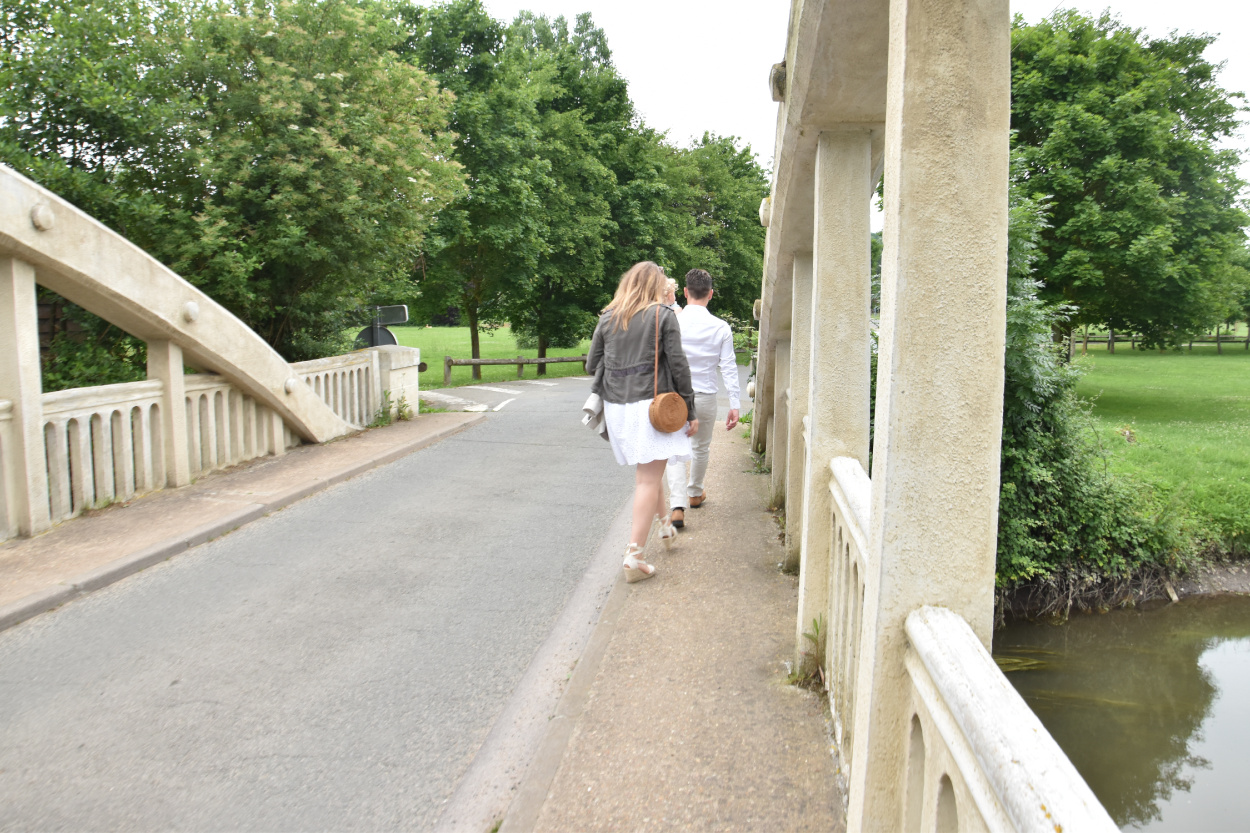

Supplement: S1 Data — (ZIP) [file pone.0234210.s002.zip › Pictures_DataBase_Environment/Clean/Clean - Urban/Clean - Urban - With Individuals/Urb.Pers.PROPRE08.1.JPG]

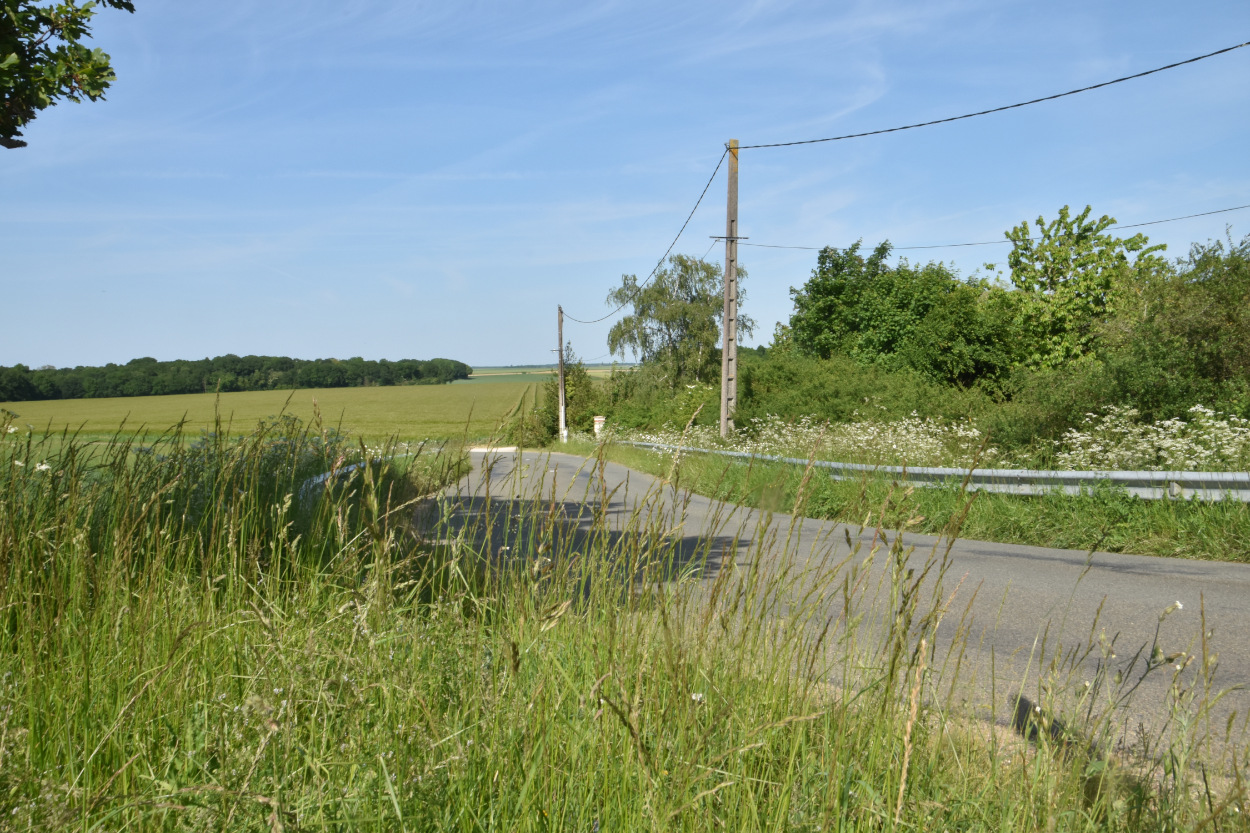

Supplement: S1 Data — (ZIP) [file pone.0234210.s002.zip › Pictures_DataBase_Environment/Clean/Clean - Urban/Clean - Urban - Without Individuals/Urb.PROPRE21.1.JPG]

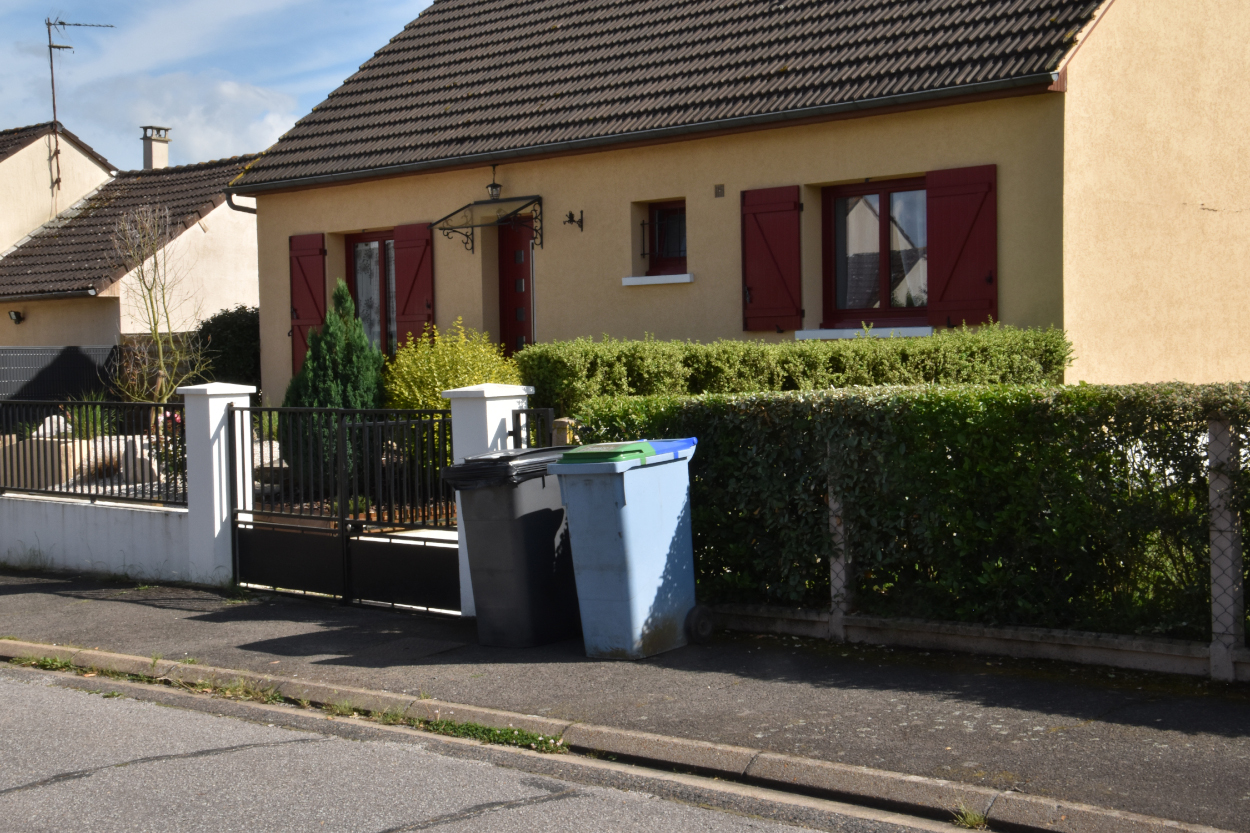

Supplement: S1 Data — (ZIP) [file pone.0234210.s002.zip › Pictures_DataBase_Environment/Clean/Clean - Urban/Clean - Urban - Without Individuals/Urb.PROPRE05.1.JPG]

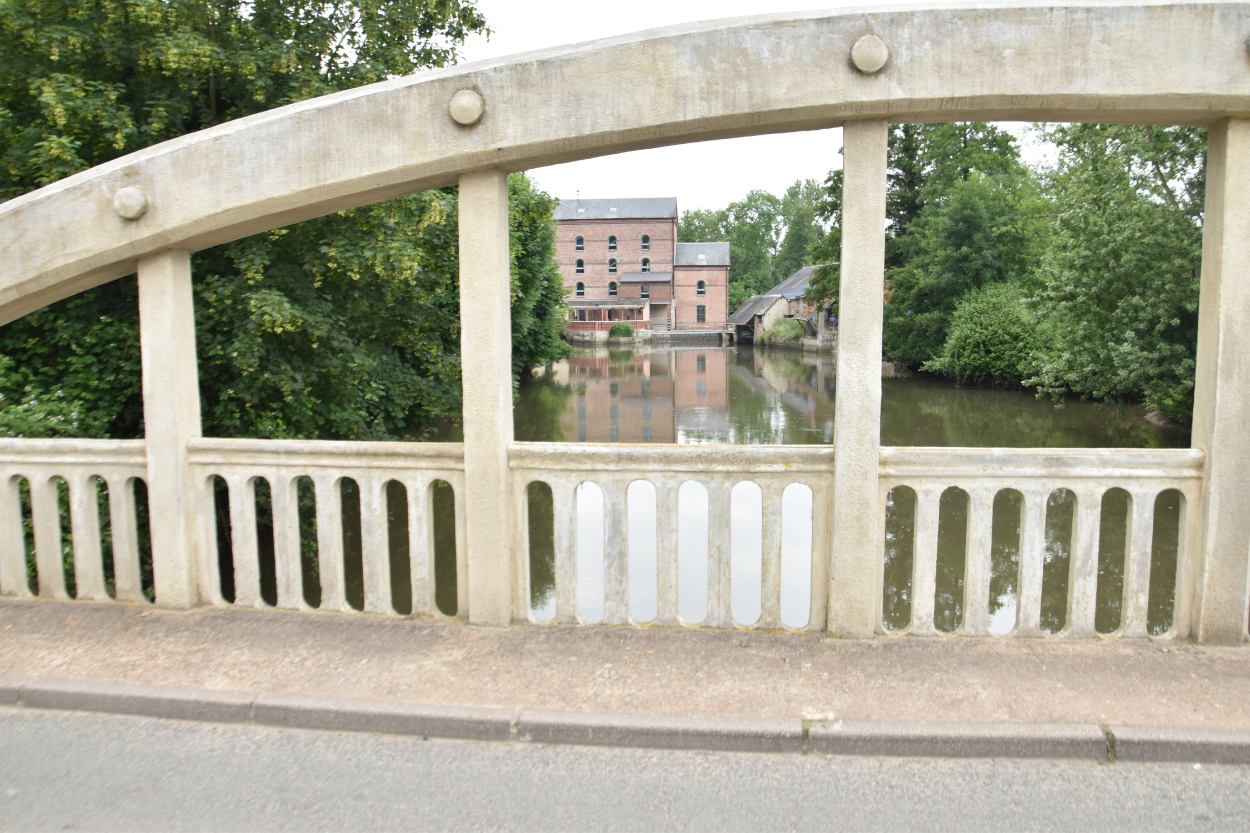

Supplement: S1 Data — (ZIP) [file pone.0234210.s002.zip › Pictures_DataBase_Environment/Clean/Clean - Urban/Clean - Urban - Without Individuals/Urb.PROPRE07.1.JPG]

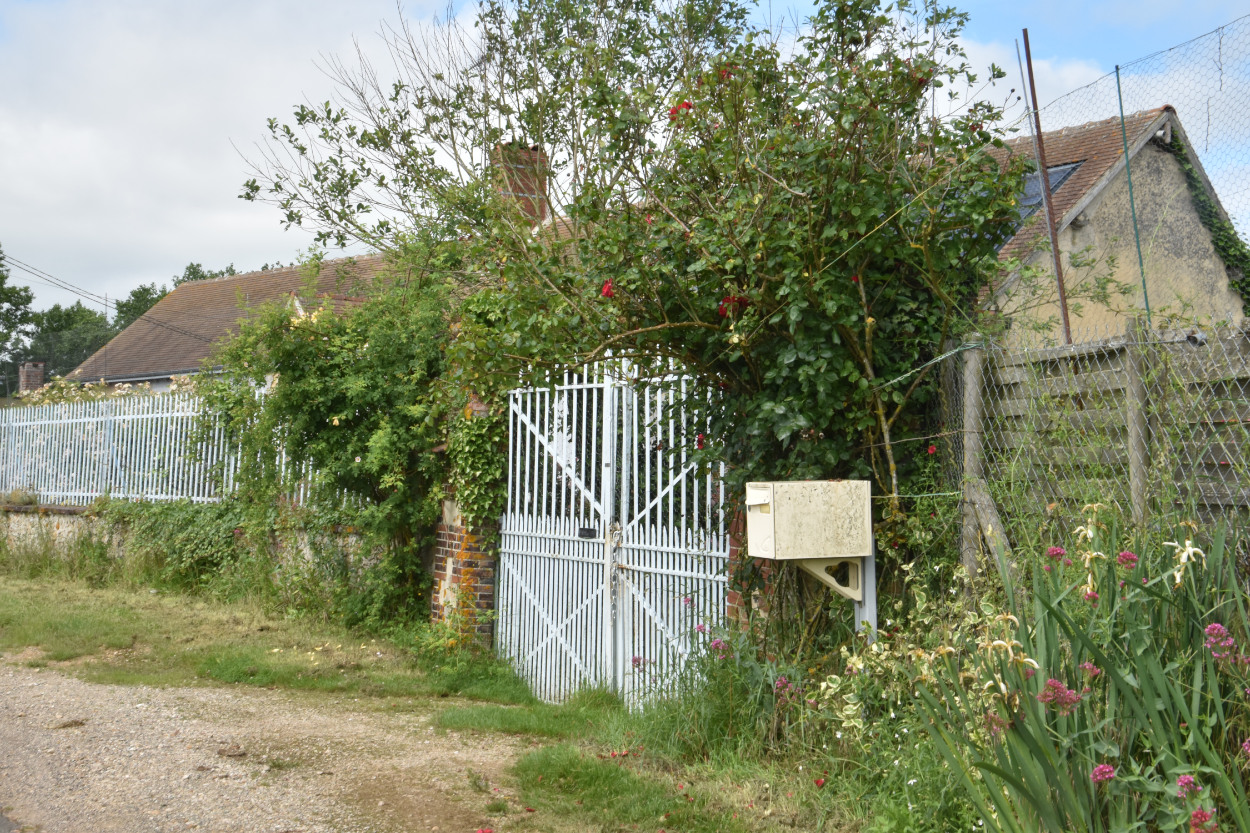

Supplement: S1 Data — (ZIP) [file pone.0234210.s002.zip › Pictures_DataBase_Environment/Clean/Clean - Urban/Clean - Urban - Without Individuals/Urb.PROPRE03.1.JPG]

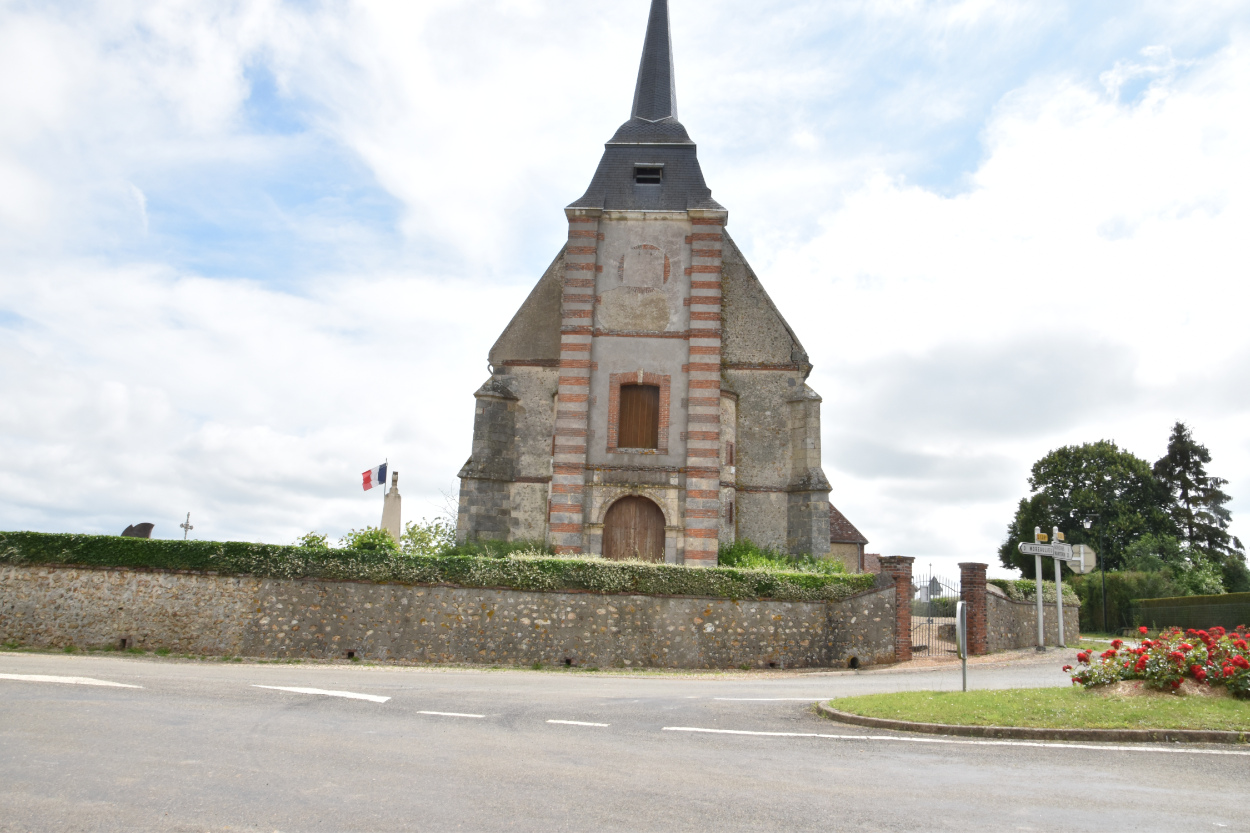

Supplement: S1 Data — (ZIP) [file pone.0234210.s002.zip › Pictures_DataBase_Environment/Clean/Clean - Urban/Clean - Urban - Without Individuals/Urb.PROPRE01.1.JPG]

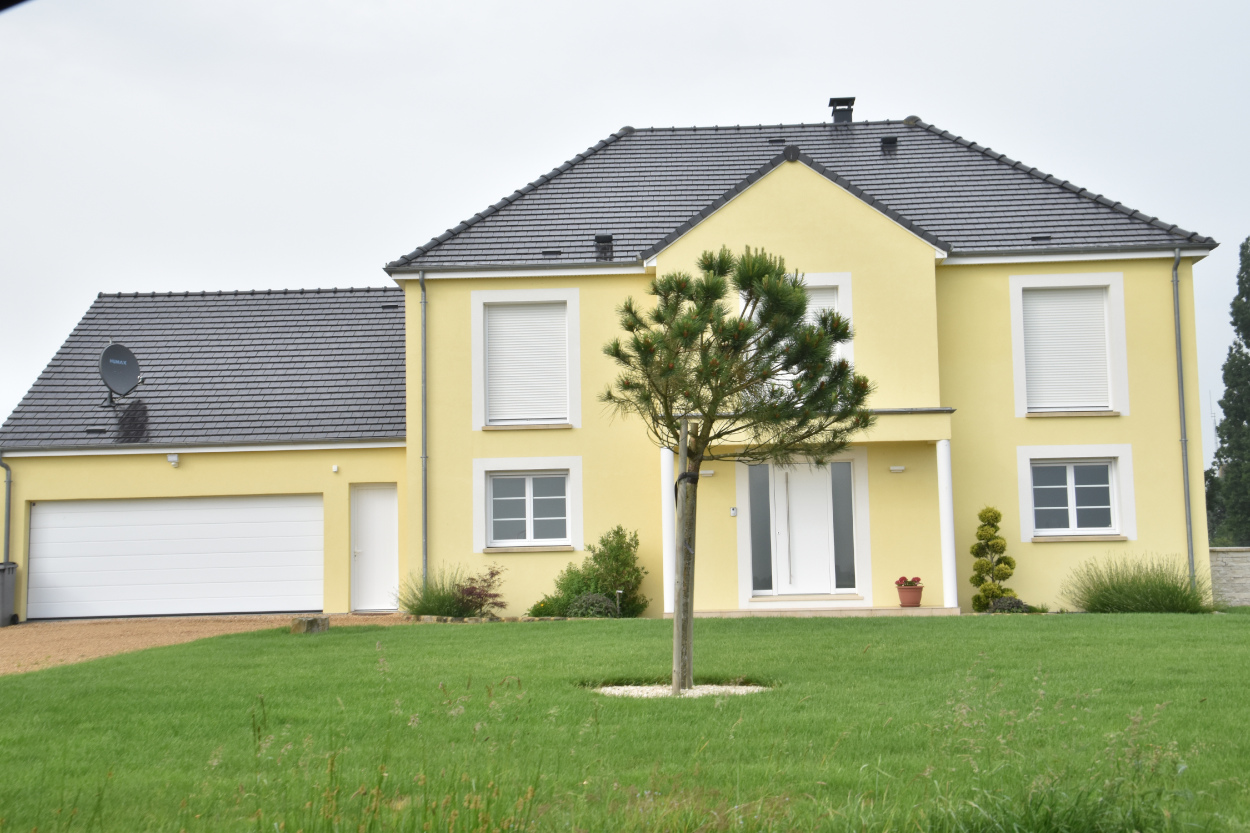

Supplement: S1 Data — (ZIP) [file pone.0234210.s002.zip › Pictures_DataBase_Environment/Clean/Clean - Urban/Clean - Urban - Without Individuals/Urb.PROPRE18.1.JPG]

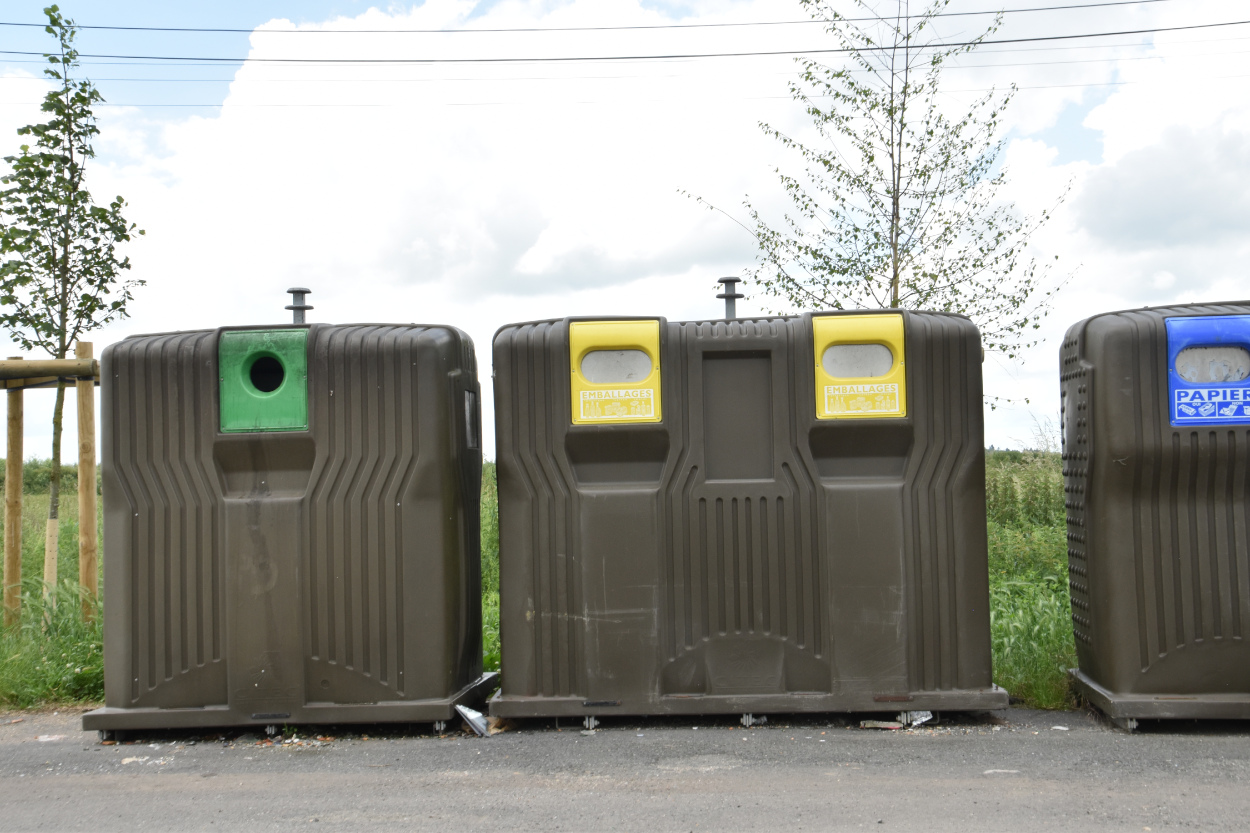

Supplement: S1 Data — (ZIP) [file pone.0234210.s002.zip › Pictures_DataBase_Environment/Clean/Clean - Urban/Clean - Urban - Without Individuals/Urb.PROPRE06.1.JPG]

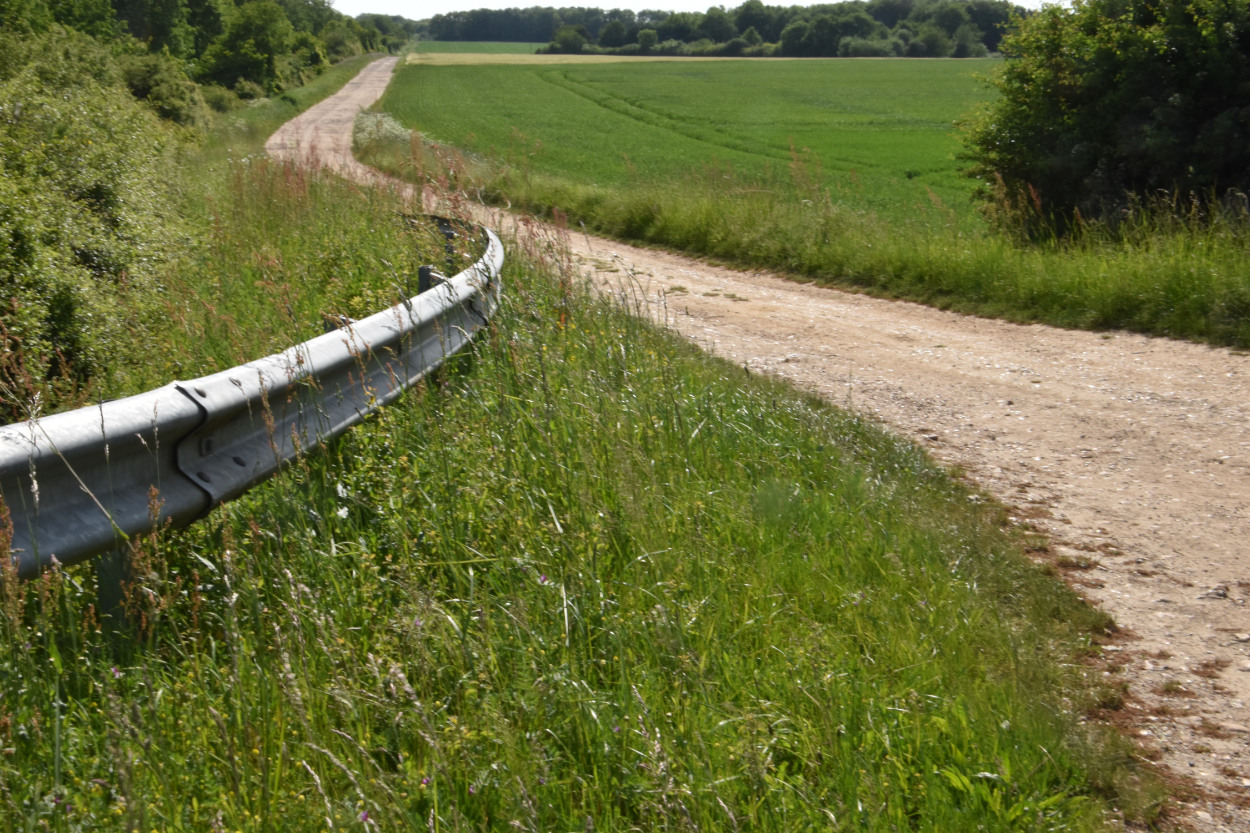

Supplement: S1 Data — (ZIP) [file pone.0234210.s002.zip › Pictures_DataBase_Environment/Clean/Clean - Urban/Clean - Urban - Without Individuals/Urb.PROPRE20.1.JPG]

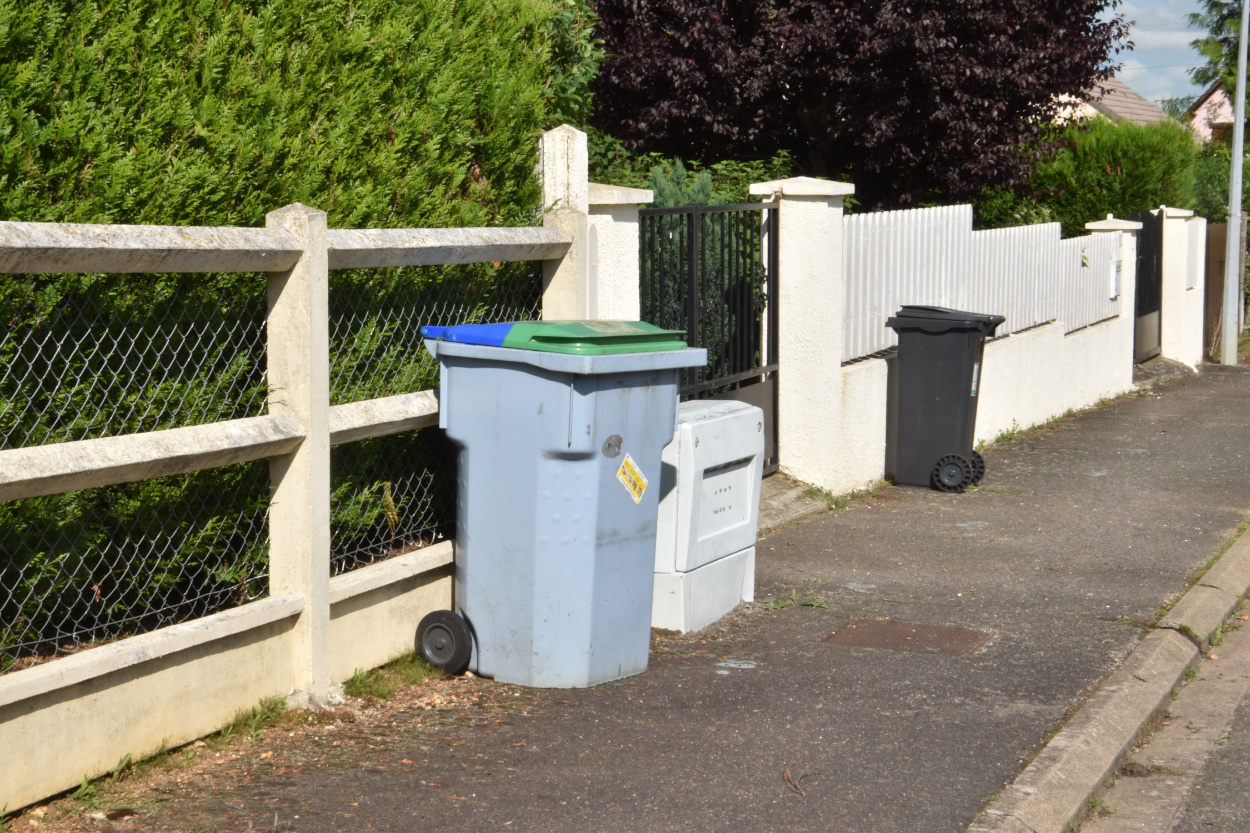

Supplement: S1 Data — (ZIP) [file pone.0234210.s002.zip › Pictures_DataBase_Environment/Clean/Clean - Urban/Clean - Urban - Without Individuals/Urb.PROPRE04.1.JPG]

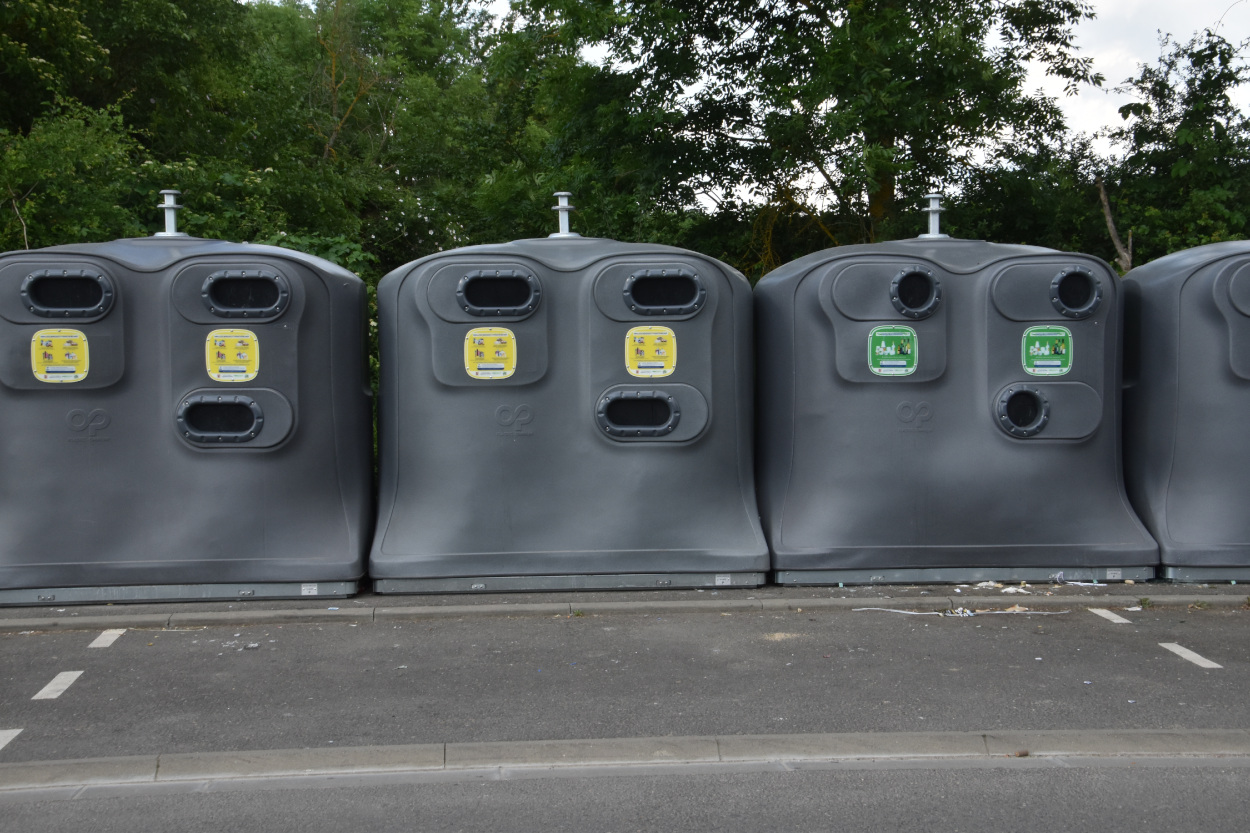

Supplement: S1 Data — (ZIP) [file pone.0234210.s002.zip › Pictures_DataBase_Environment/Clean/Clean - Urban/Clean - Urban - Without Individuals/Urb.PROPRE19.1.JPG]

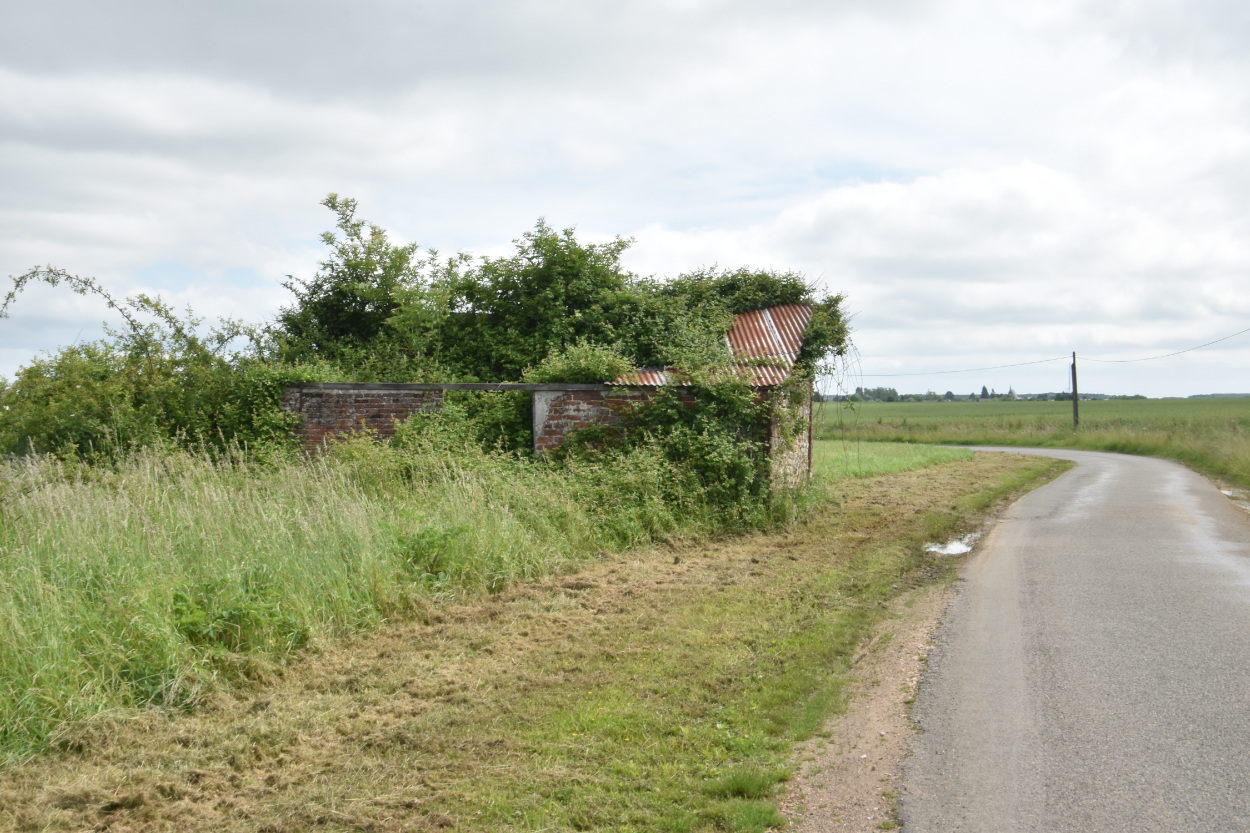

Supplement: S1 Data — (ZIP) [file pone.0234210.s002.zip › Pictures_DataBase_Environment/Clean/Clean - Urban/Clean - Urban - Without Individuals/Urb.PROPRE02.1.JPG]

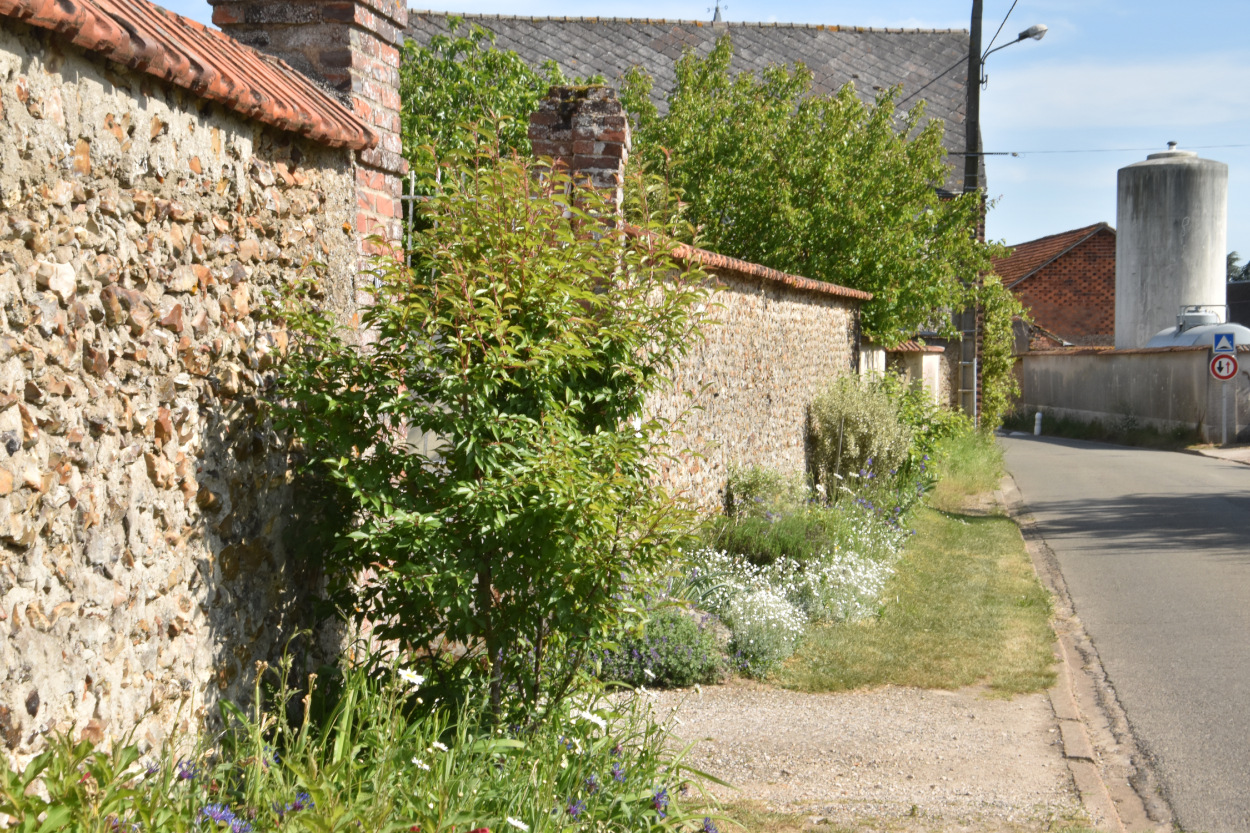

Supplement: S1 Data — (ZIP) [file pone.0234210.s002.zip › Pictures_DataBase_Environment/Clean/Clean - Urban/Clean - Urban - Without Individuals/Urb.PROPRE17.1.JPG]

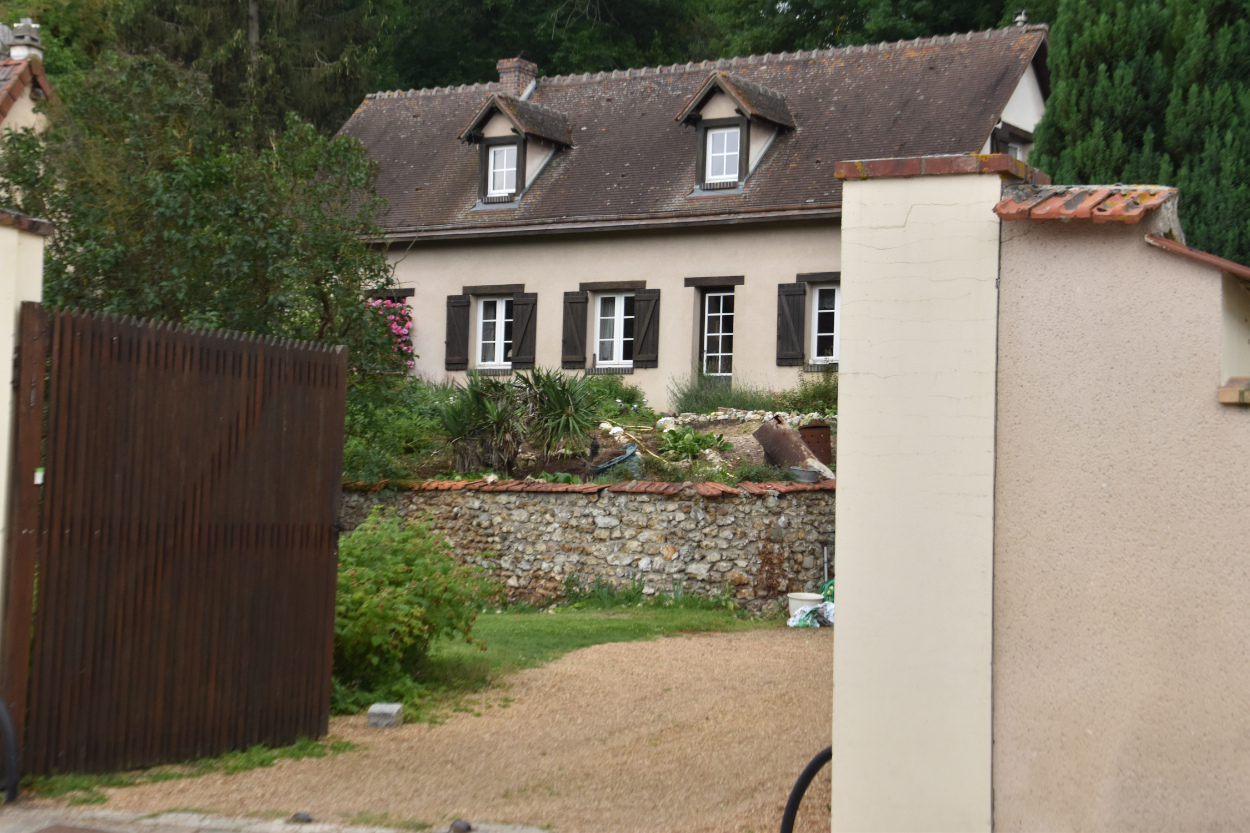

Supplement: S1 Data — (ZIP) [file pone.0234210.s002.zip › Pictures_DataBase_Environment/Clean/Clean - Urban/Clean - Urban - Without Individuals/Urb.PROPRE15.1.JPG]

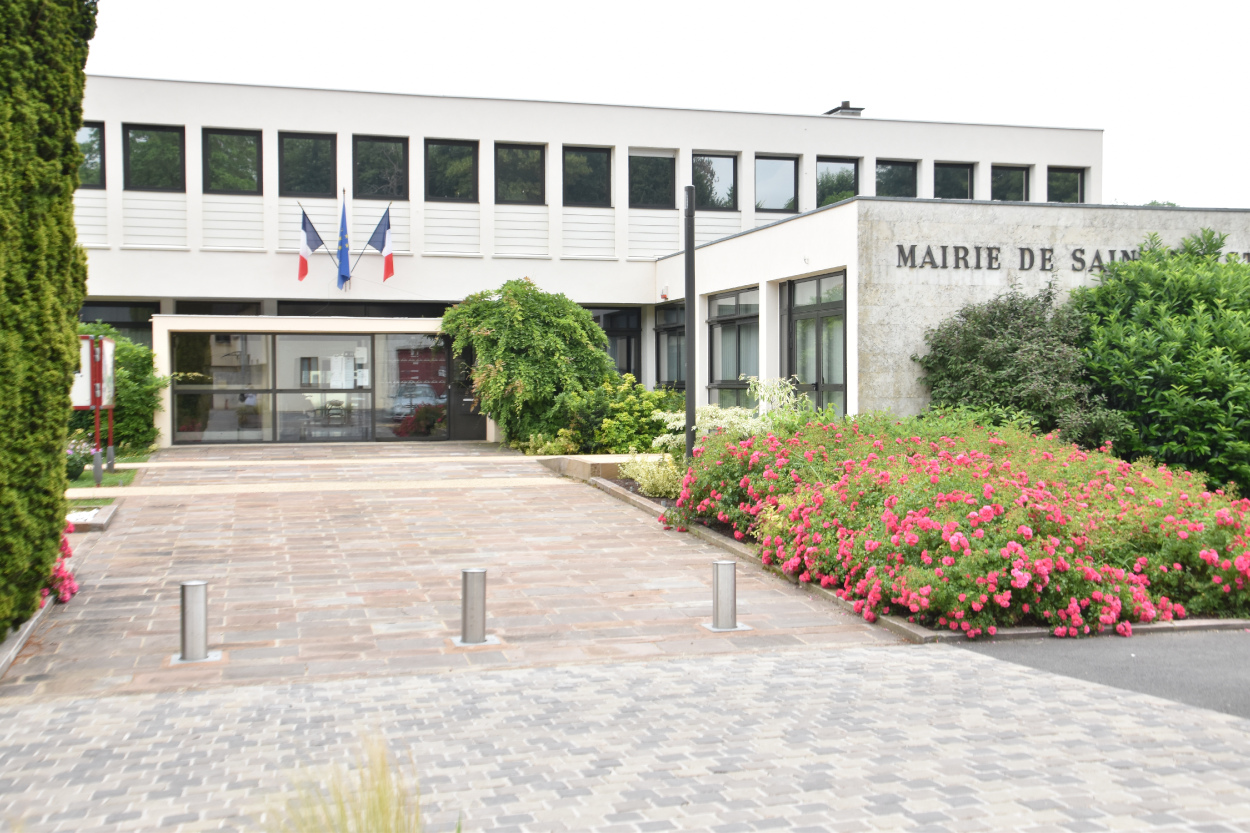

Supplement: S1 Data — (ZIP) [file pone.0234210.s002.zip › Pictures_DataBase_Environment/Clean/Clean - Urban/Clean - Urban - Without Individuals/Urb.PROPRE08.1.JPG]

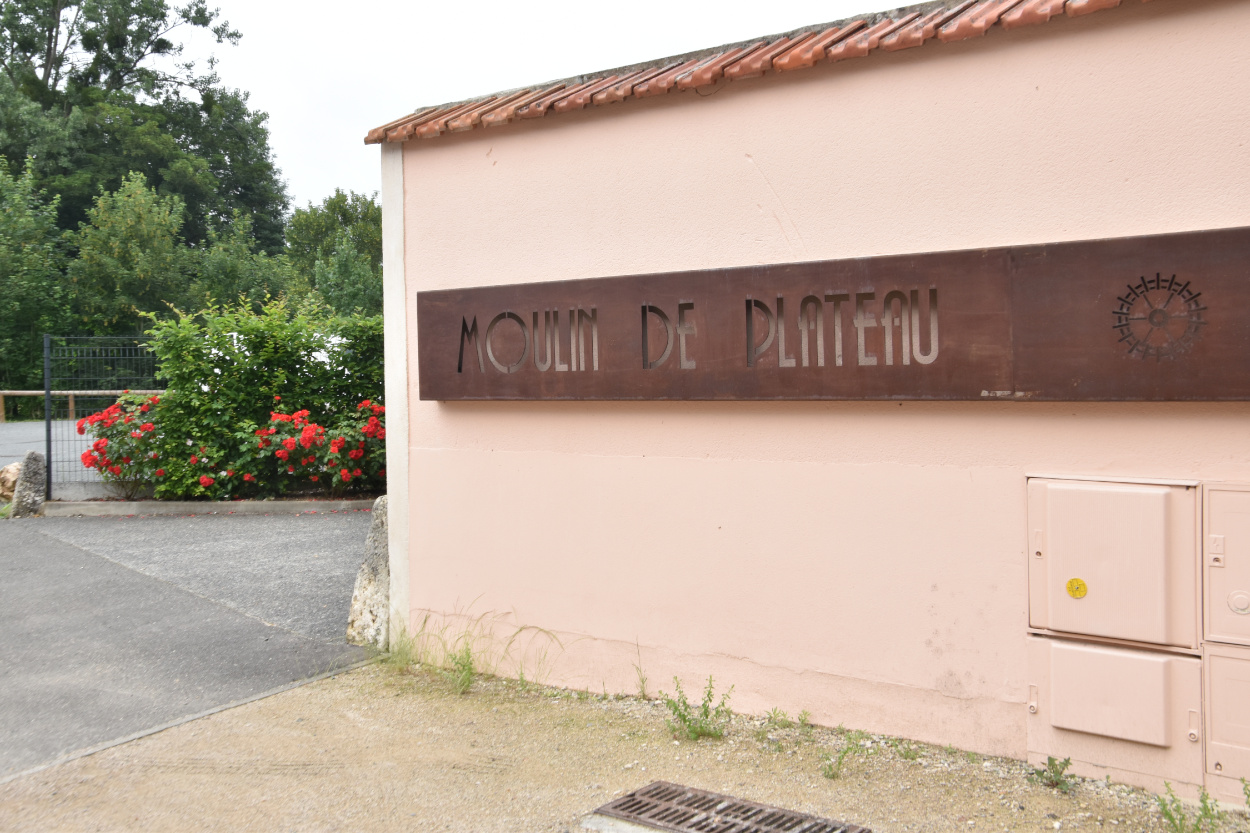

Supplement: S1 Data — (ZIP) [file pone.0234210.s002.zip › Pictures_DataBase_Environment/Clean/Clean - Urban/Clean - Urban - Without Individuals/Urb.PROPRE11.1.JPG]

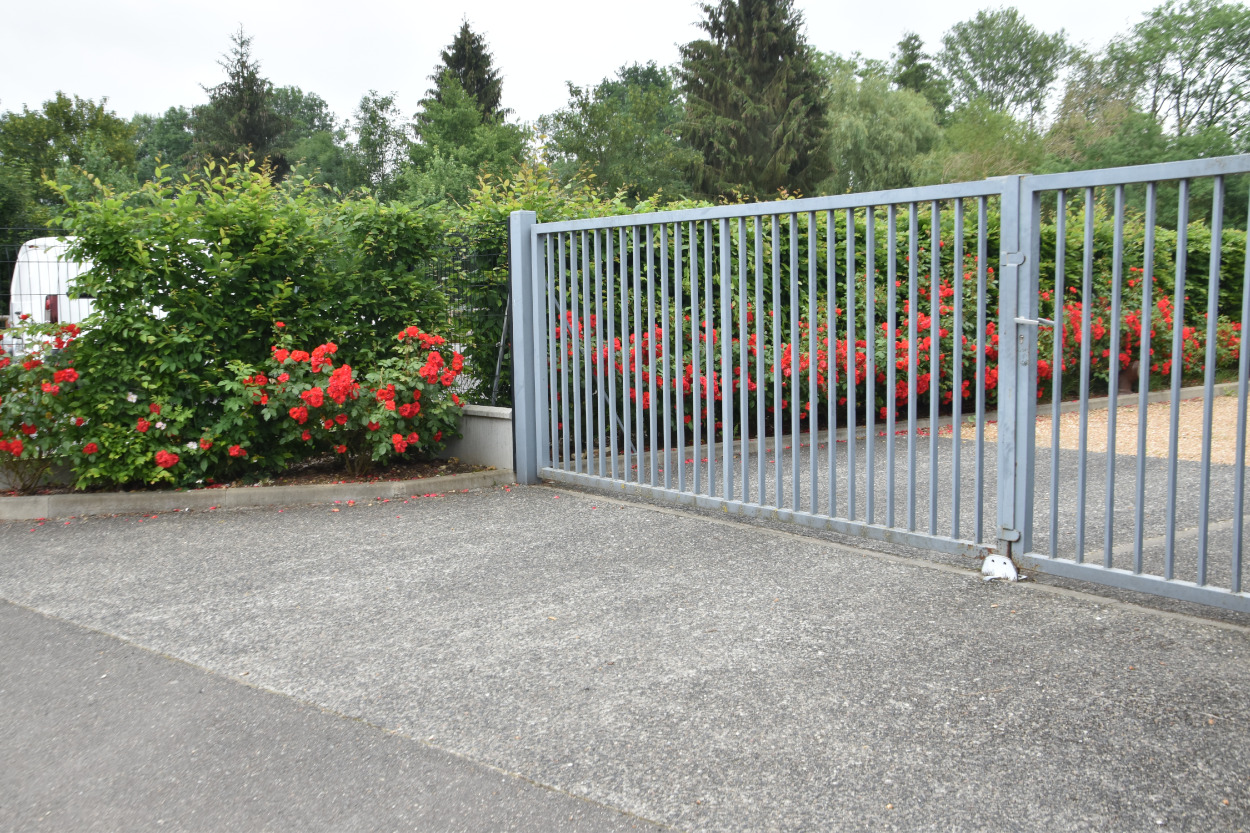

Supplement: S1 Data — (ZIP) [file pone.0234210.s002.zip › Pictures_DataBase_Environment/Clean/Clean - Urban/Clean - Urban - Without Individuals/Urb.PROPRE13.1.JPG]

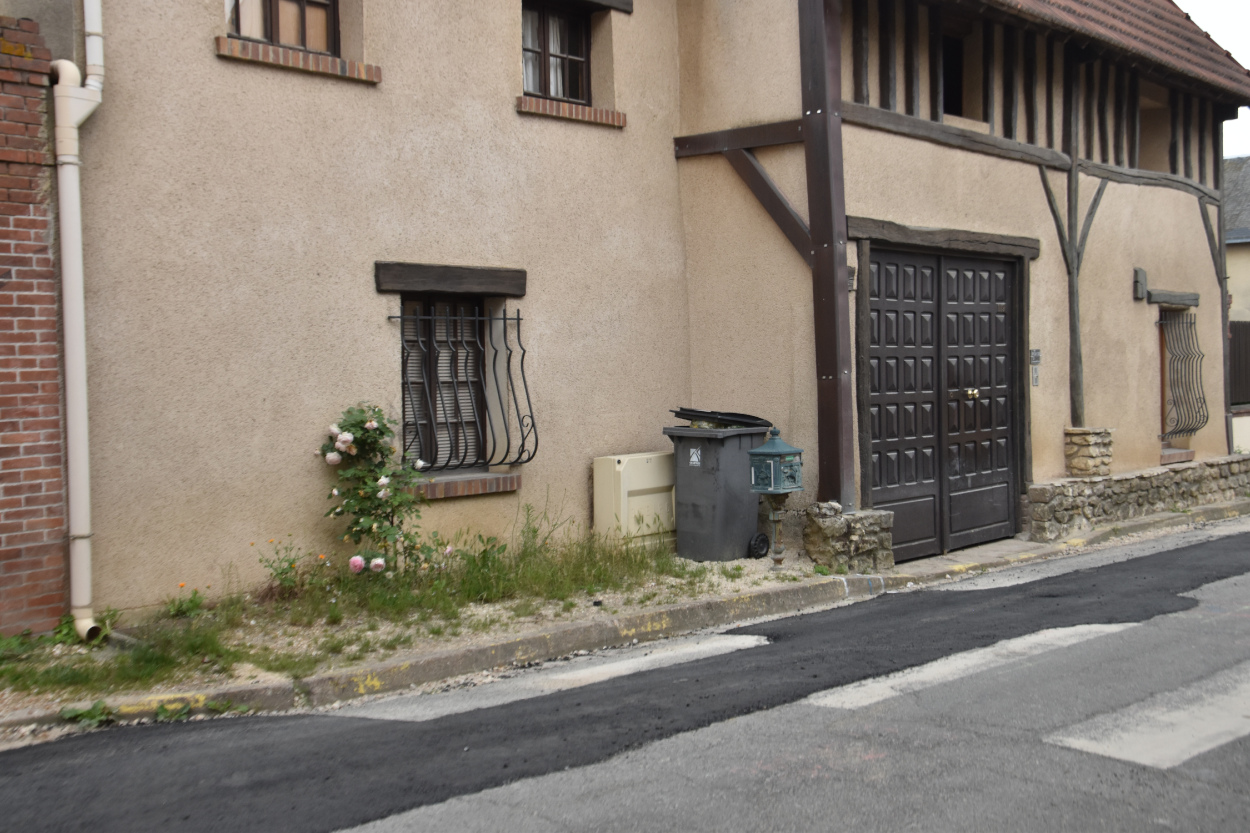

Supplement: S1 Data — (ZIP) [file pone.0234210.s002.zip › Pictures_DataBase_Environment/Clean/Clean - Urban/Clean - Urban - Without Individuals/Urb.PROPRE14.1.JPG]

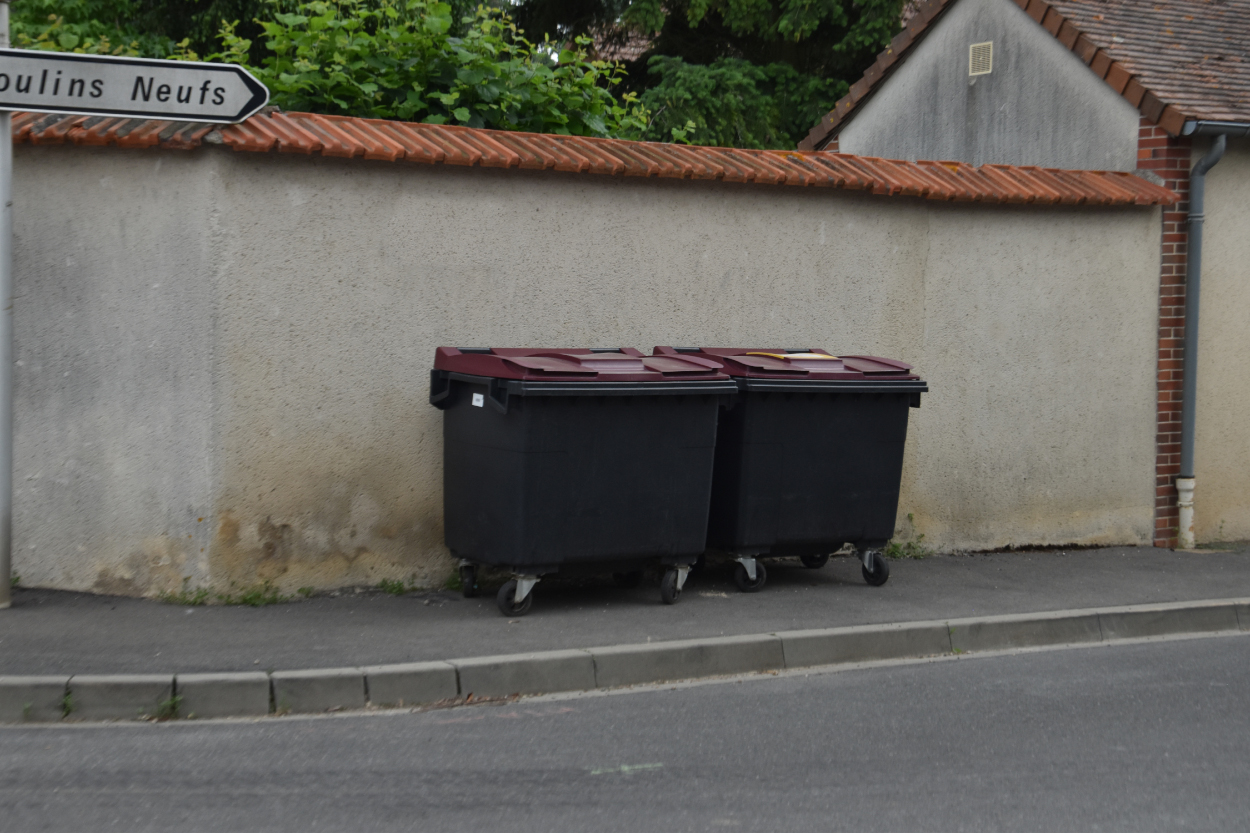

Supplement: S1 Data — (ZIP) [file pone.0234210.s002.zip › Pictures_DataBase_Environment/Clean/Clean - Urban/Clean - Urban - Without Individuals/Urb.PROPRE16.1.JPG]

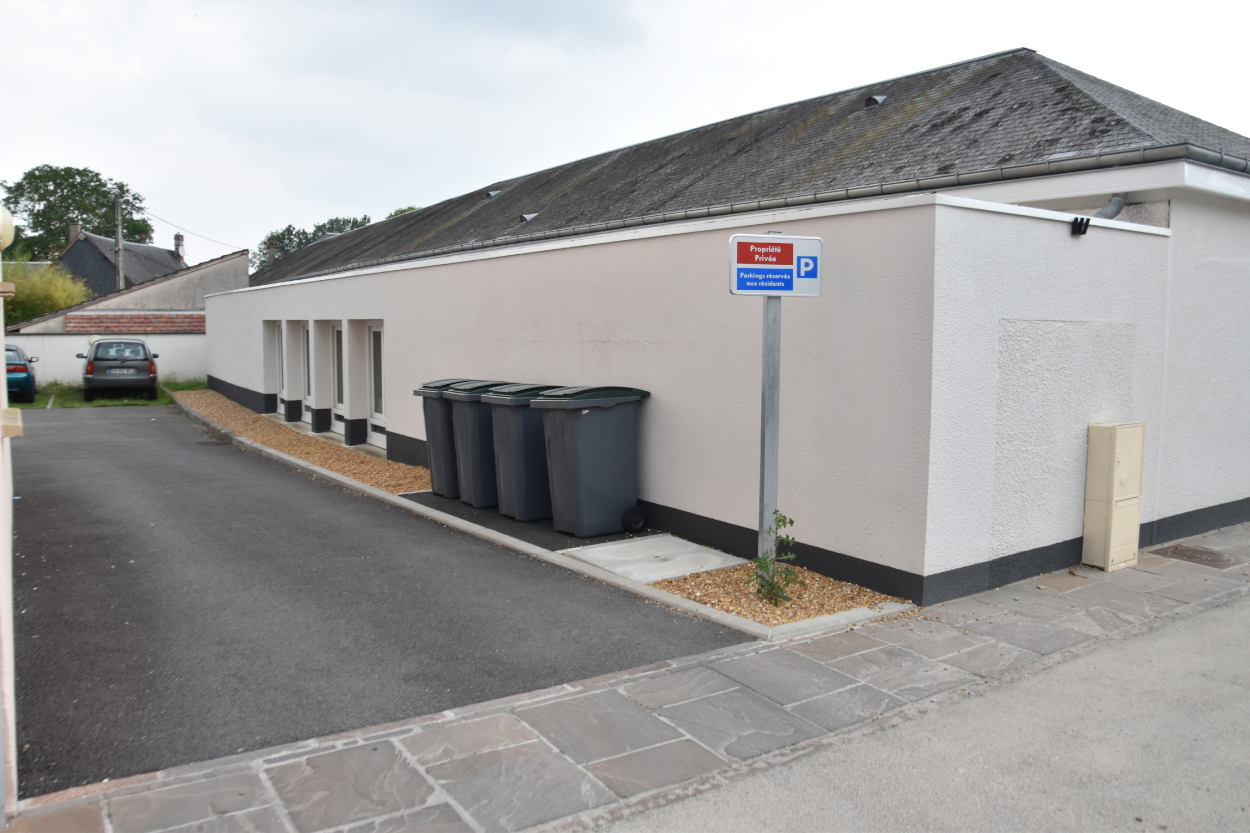

Supplement: S1 Data — (ZIP) [file pone.0234210.s002.zip › Pictures_DataBase_Environment/Clean/Clean - Urban/Clean - Urban - Without Individuals/Urb.PROPRE12.1.JPG]

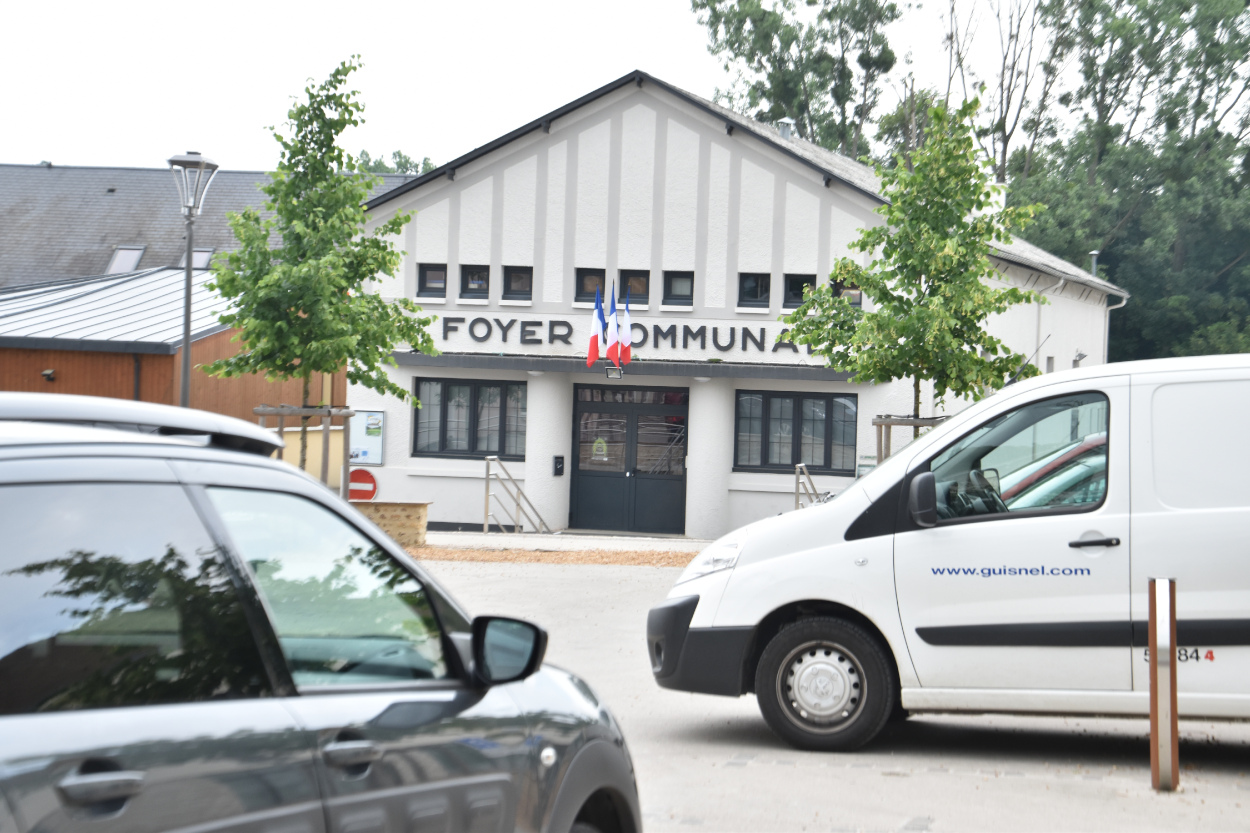

Supplement: S1 Data — (ZIP) [file pone.0234210.s002.zip › Pictures_DataBase_Environment/Clean/Clean - Urban/Clean - Urban - Without Individuals/Urb.PROPRE09.1.JPG]

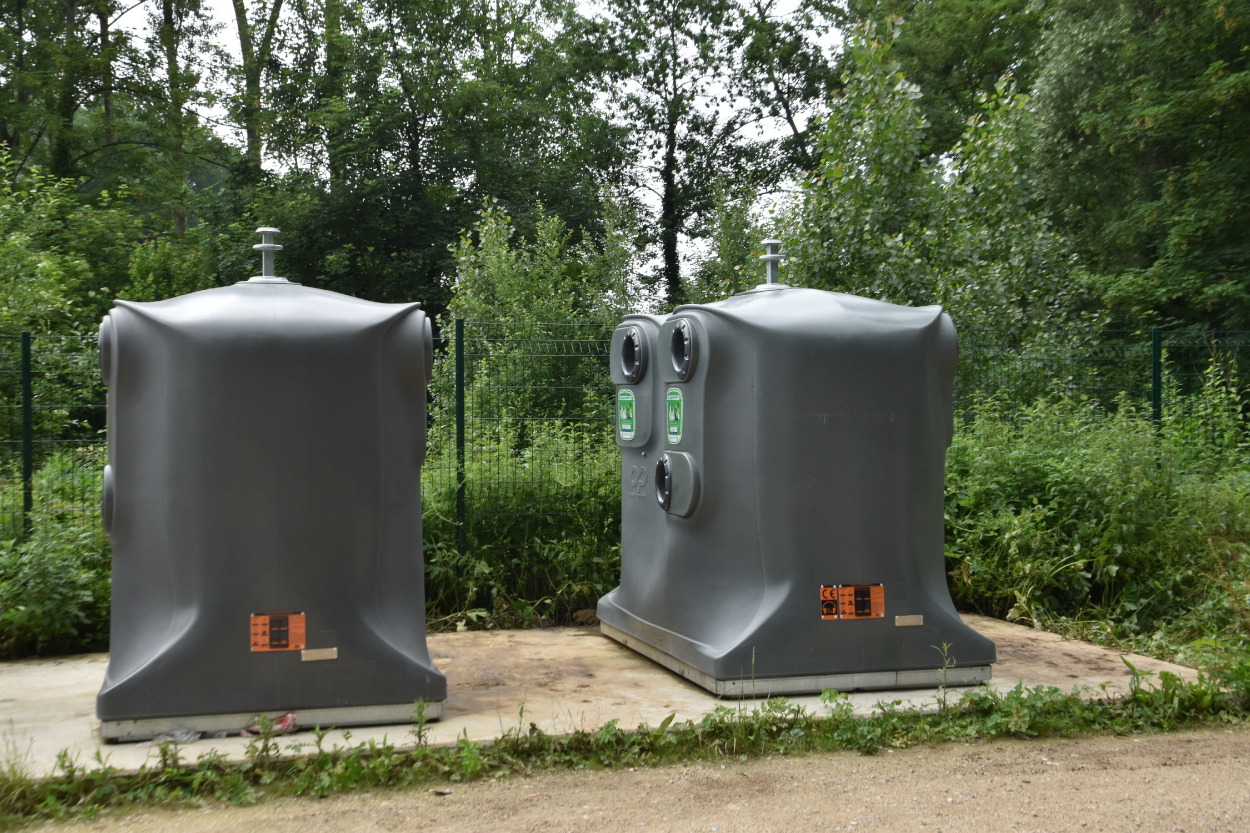

Supplement: S1 Data — (ZIP) [file pone.0234210.s002.zip › Pictures_DataBase_Environment/Clean/Clean - Urban/Clean - Urban - Without Individuals/Urb.PROPRE10.1.JPG]

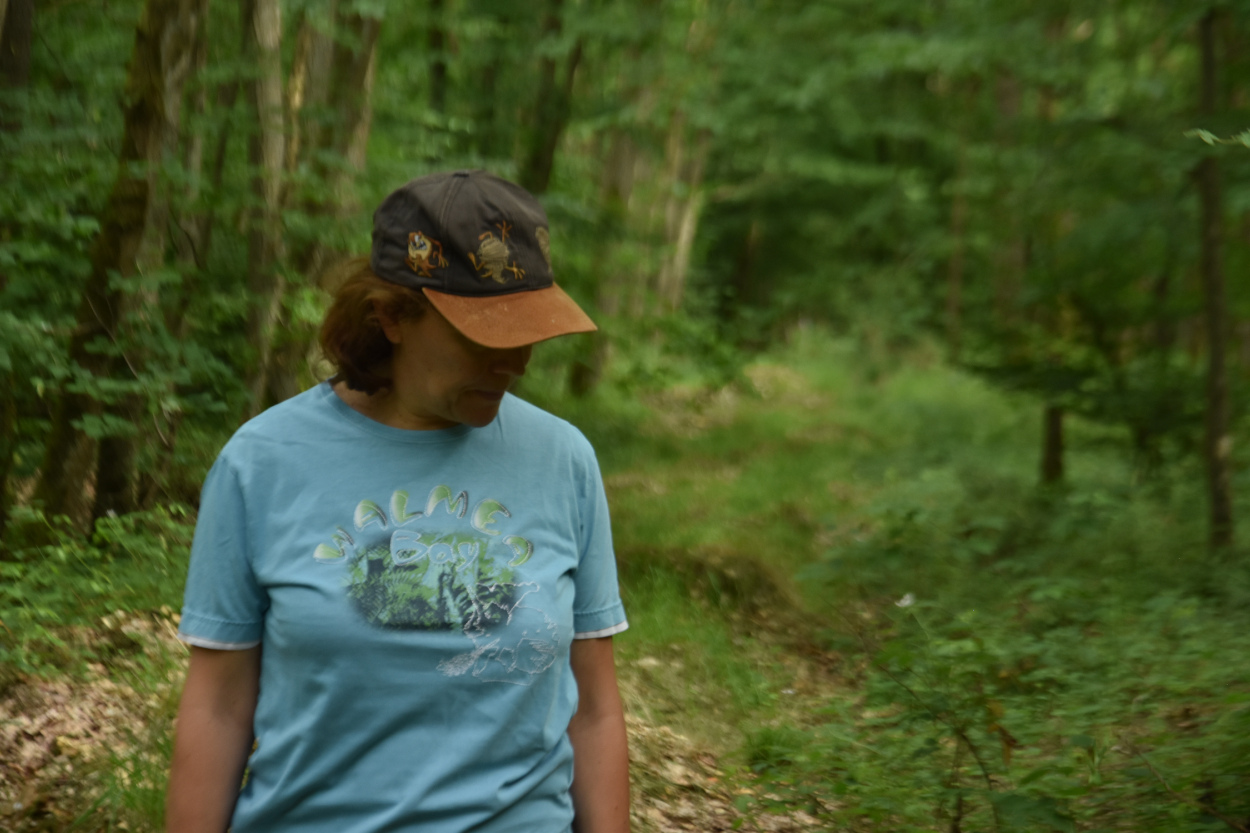

Supplement: S1 Data — (ZIP) [file pone.0234210.s002.zip › Pictures_DataBase_Environment/Clean/Clean - Rural/Clean - Rural - With Individuals/Rur.Pers.PROPRE02.1.JPG]

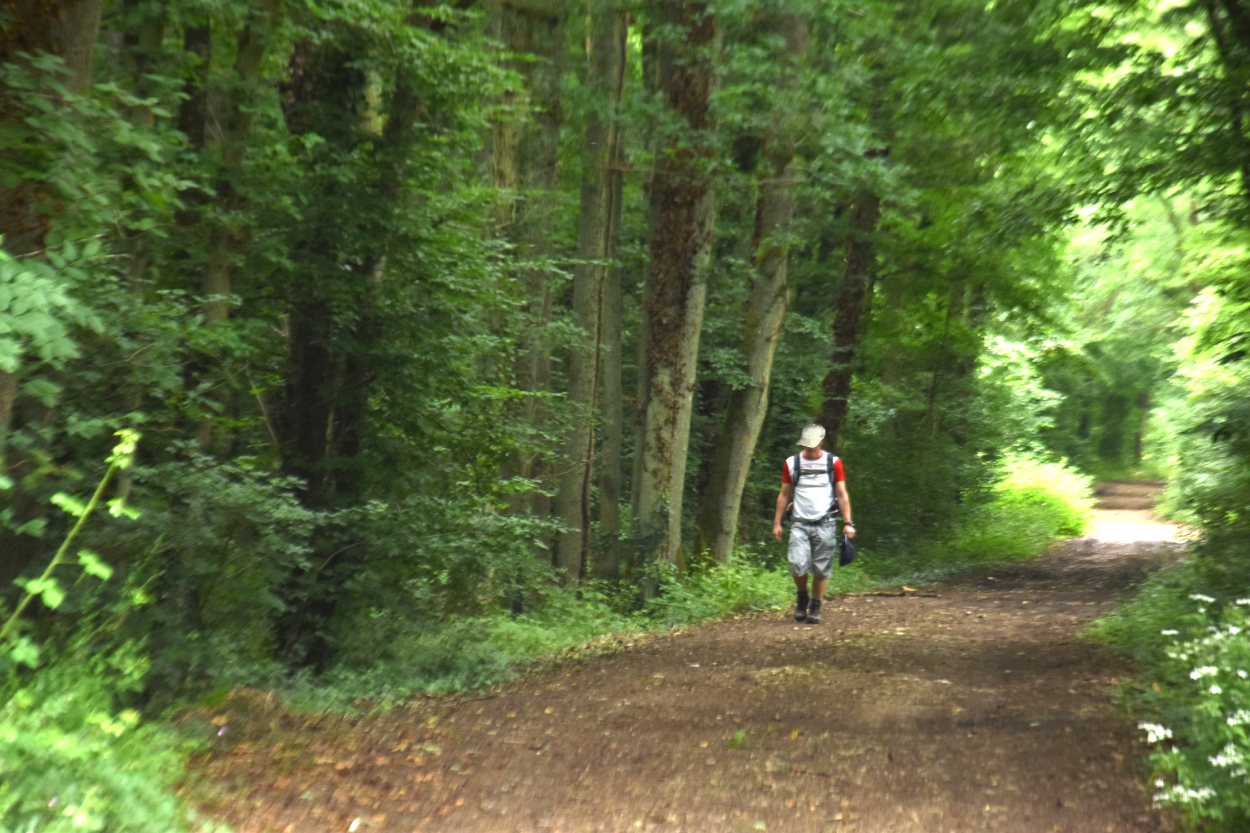

Supplement: S1 Data — (ZIP) [file pone.0234210.s002.zip › Pictures_DataBase_Environment/Clean/Clean - Rural/Clean - Rural - With Individuals/Rur.Pers.PROPRE06.1.JPG]

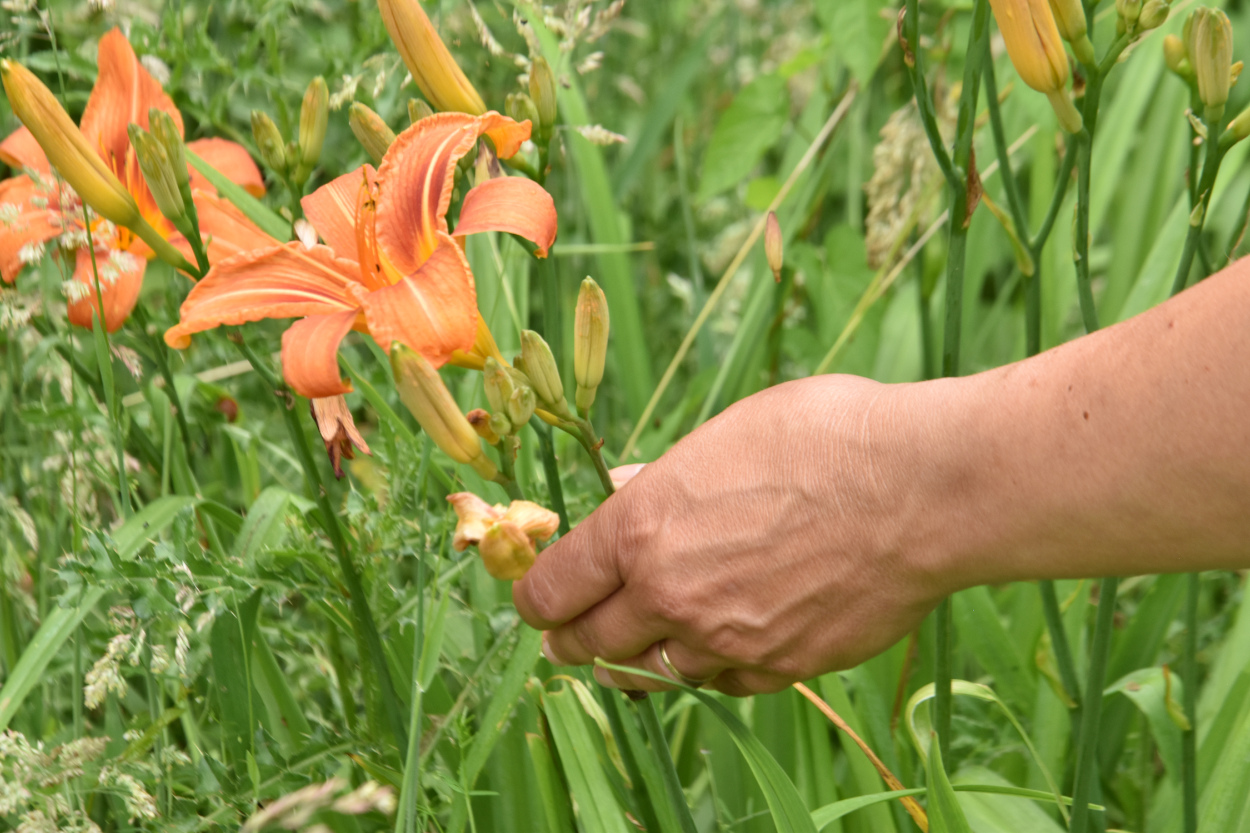

Supplement: S1 Data — (ZIP) [file pone.0234210.s002.zip › Pictures_DataBase_Environment/Clean/Clean - Rural/Clean - Rural - With Individuals/Rur.Pers.PROPRE04.1.JPG]

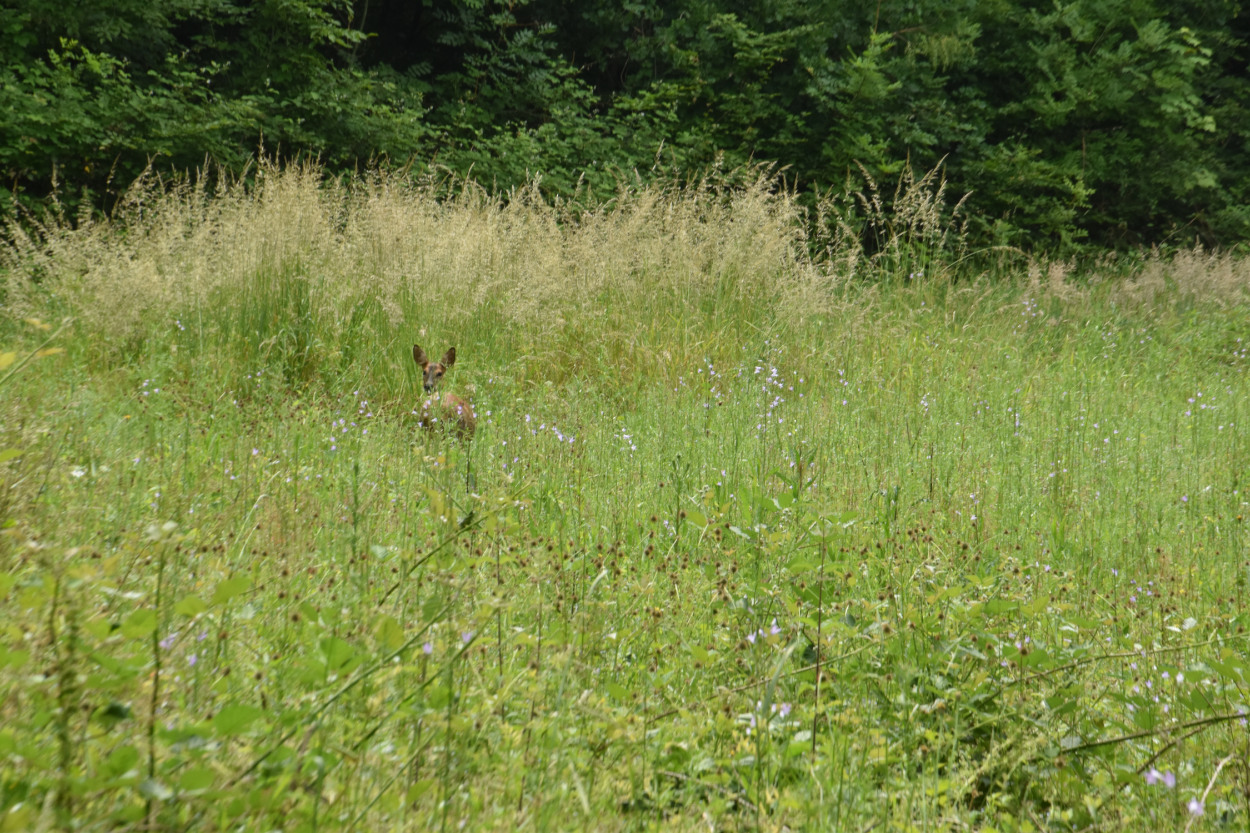

Supplement: S1 Data — (ZIP) [file pone.0234210.s002.zip › Pictures_DataBase_Environment/Clean/Clean - Rural/Clean - Rural - With Individuals/Rur.Pers.PROPRE03.1.JPG]

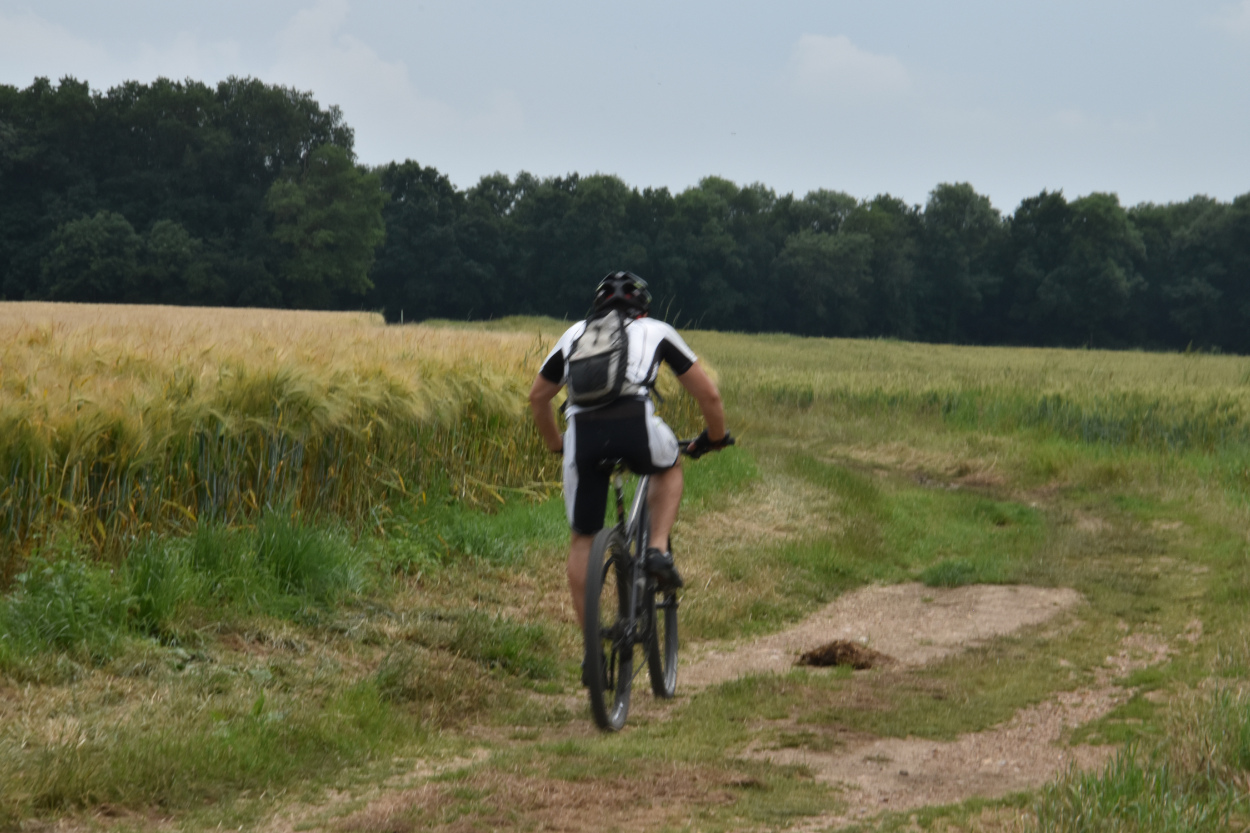

Supplement: S1 Data — (ZIP) [file pone.0234210.s002.zip › Pictures_DataBase_Environment/Clean/Clean - Rural/Clean - Rural - With Individuals/Rur.Pers.PROPRE01.1.JPG]

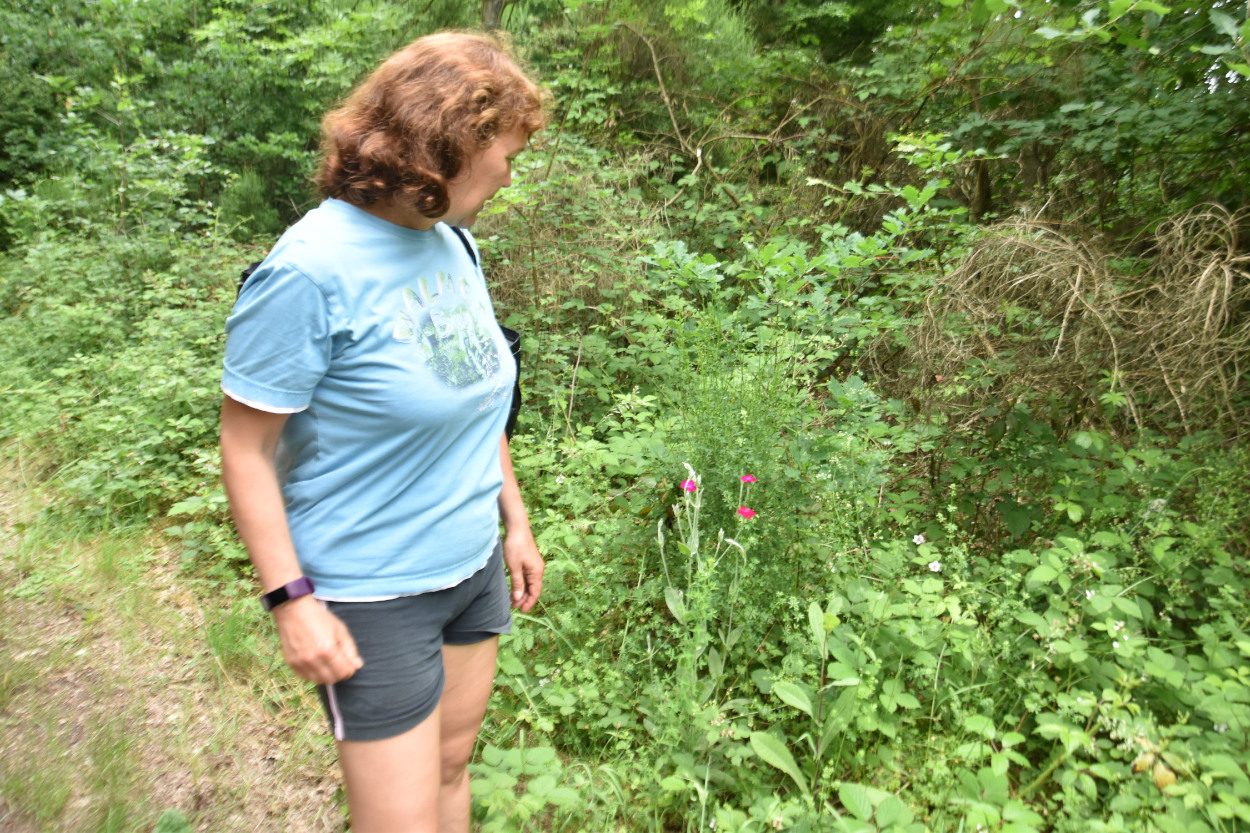

Supplement: S1 Data — (ZIP) [file pone.0234210.s002.zip › Pictures_DataBase_Environment/Clean/Clean - Rural/Clean - Rural - With Individuals/Rur.Pers.PROPRE05.1.JPG]

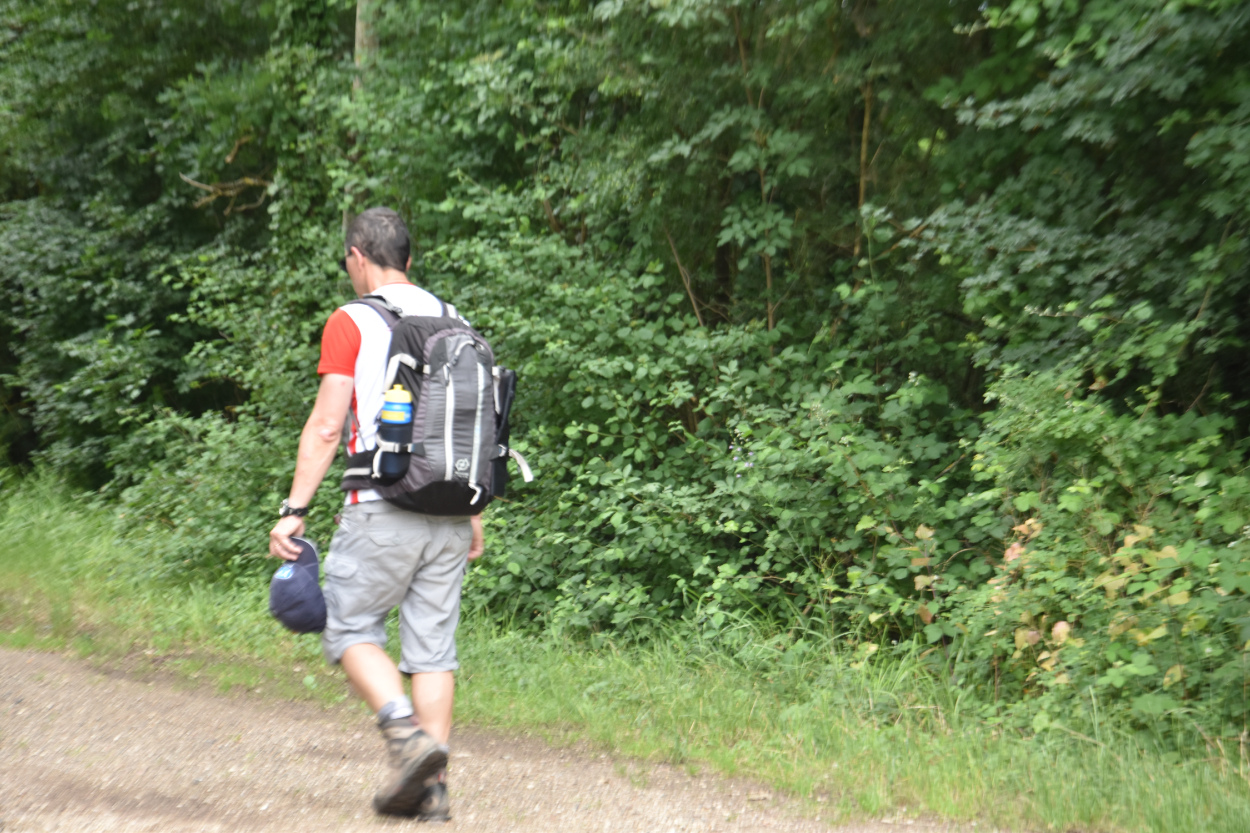

Supplement: S1 Data — (ZIP) [file pone.0234210.s002.zip › Pictures_DataBase_Environment/Clean/Clean - Rural/Clean - Rural - With Individuals/Rur.Pers.PROPRE07.1.JPG]

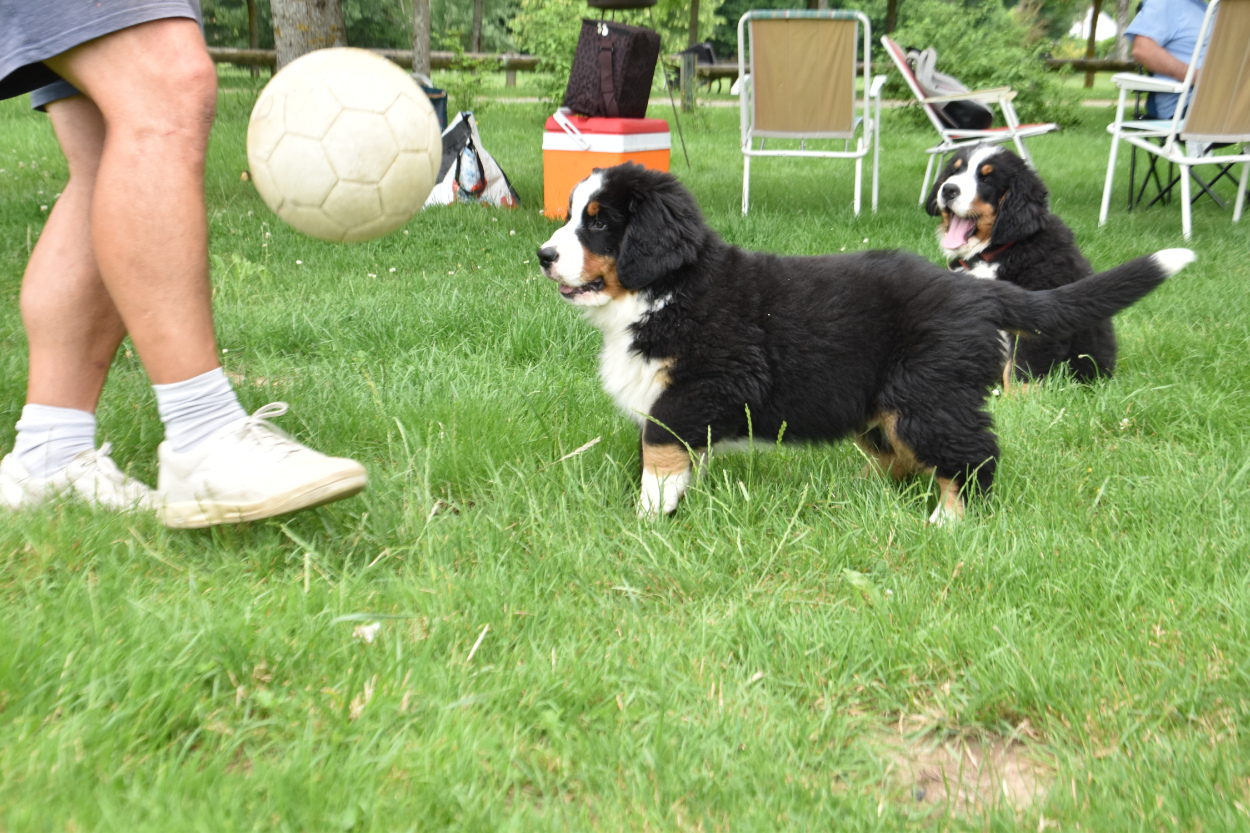

Supplement: S1 Data — (ZIP) [file pone.0234210.s002.zip › Pictures_DataBase_Environment/Clean/Clean - Rural/Clean - Rural - With Individuals/Rur.Pers.PROPRE12.1.JPG]

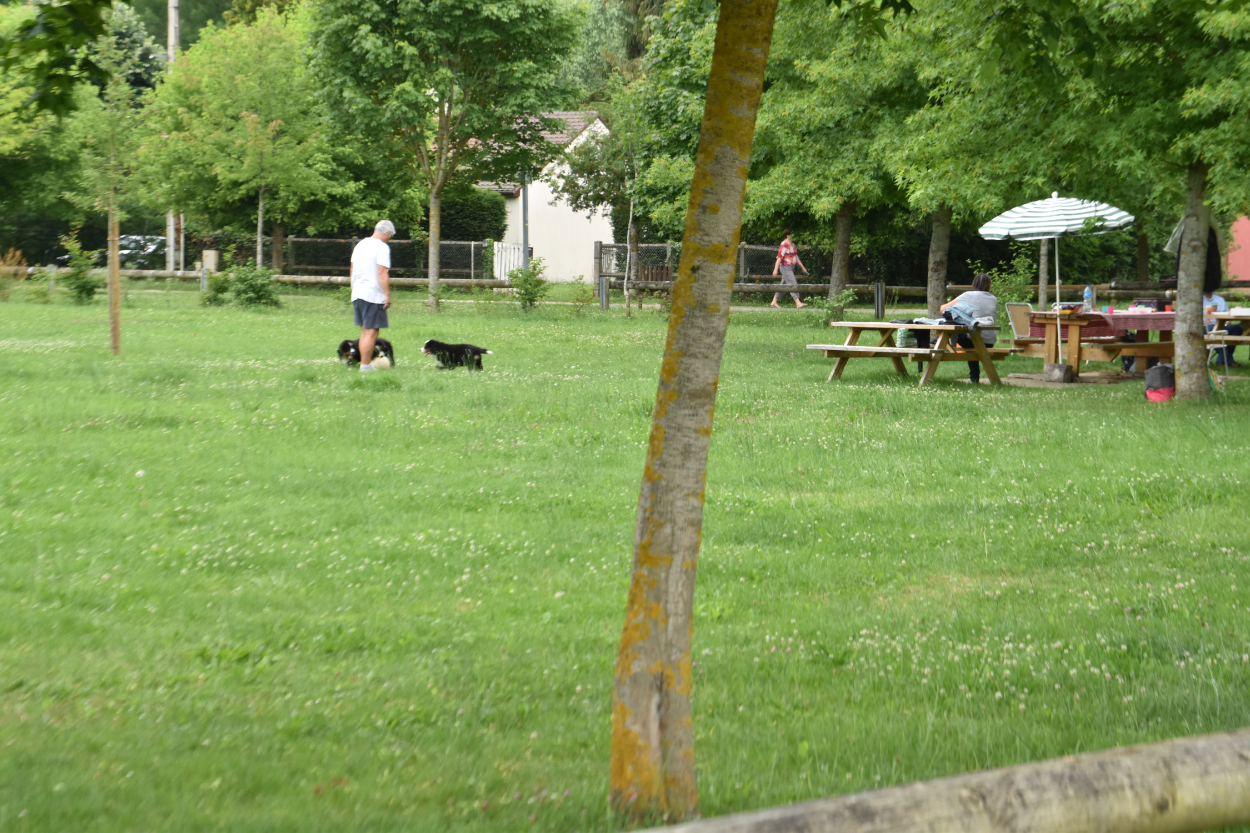

Supplement: S1 Data — (ZIP) [file pone.0234210.s002.zip › Pictures_DataBase_Environment/Clean/Clean - Rural/Clean - Rural - With Individuals/Rur.Pers.PROPRE09.1.JPG]

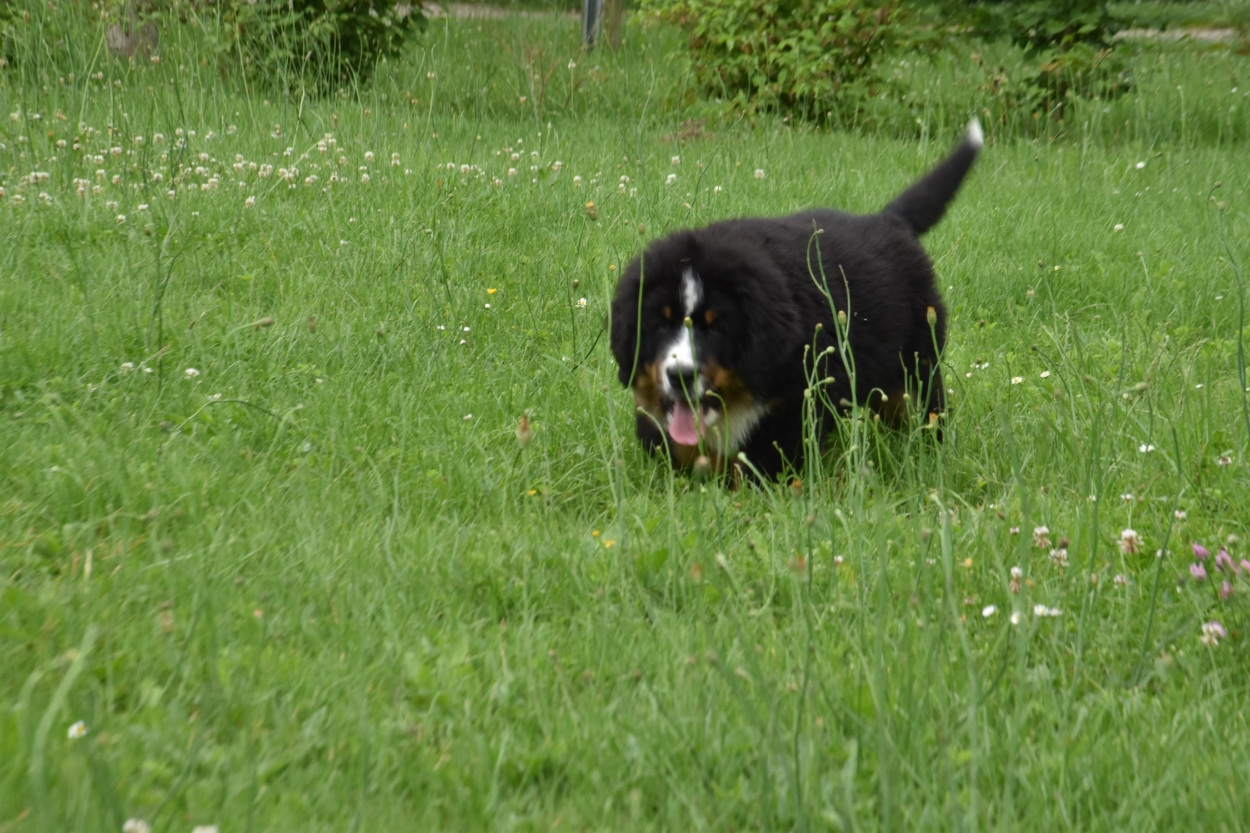

Supplement: S1 Data — (ZIP) [file pone.0234210.s002.zip › Pictures_DataBase_Environment/Clean/Clean - Rural/Clean - Rural - With Individuals/Rur.Pers.PROPRE10.1.JPG]

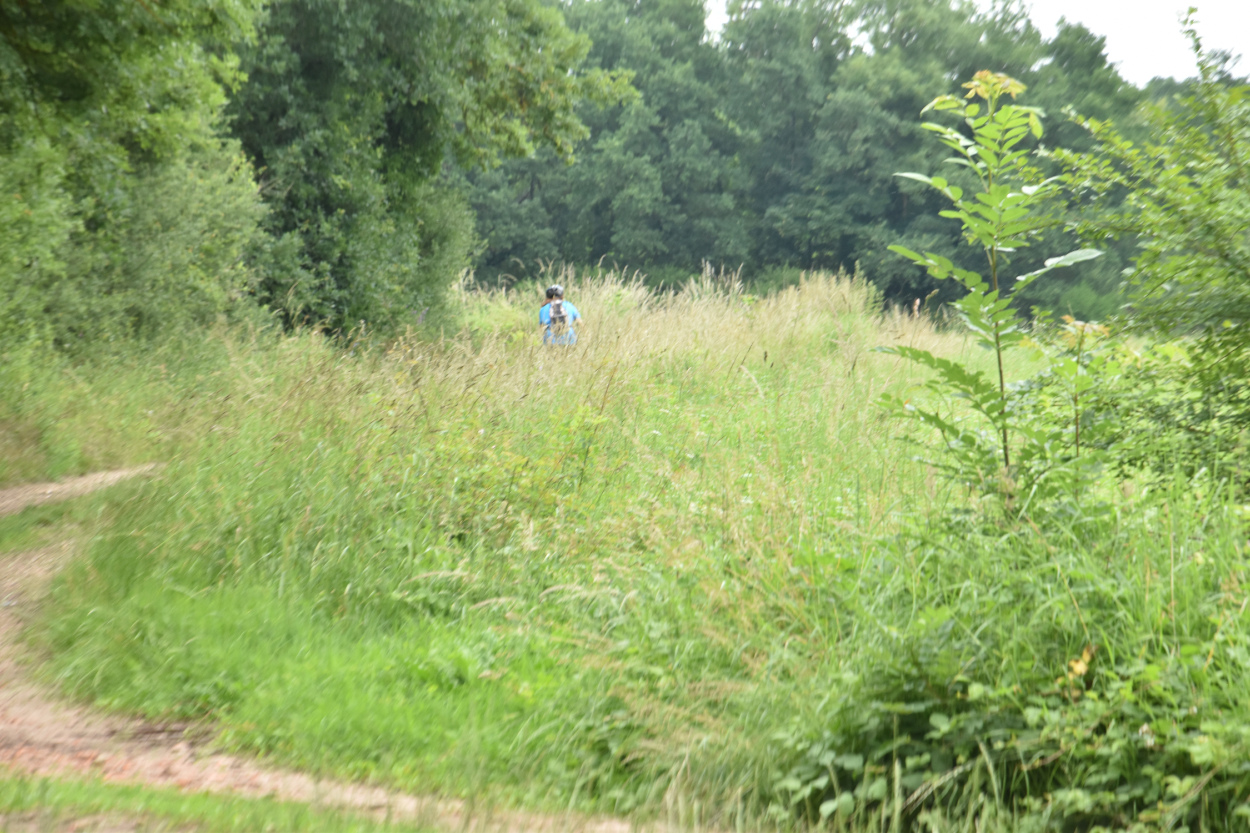

Supplement: S1 Data — (ZIP) [file pone.0234210.s002.zip › Pictures_DataBase_Environment/Clean/Clean - Rural/Clean - Rural - With Individuals/Rur.Pers.PROPRE08.1.JPG]

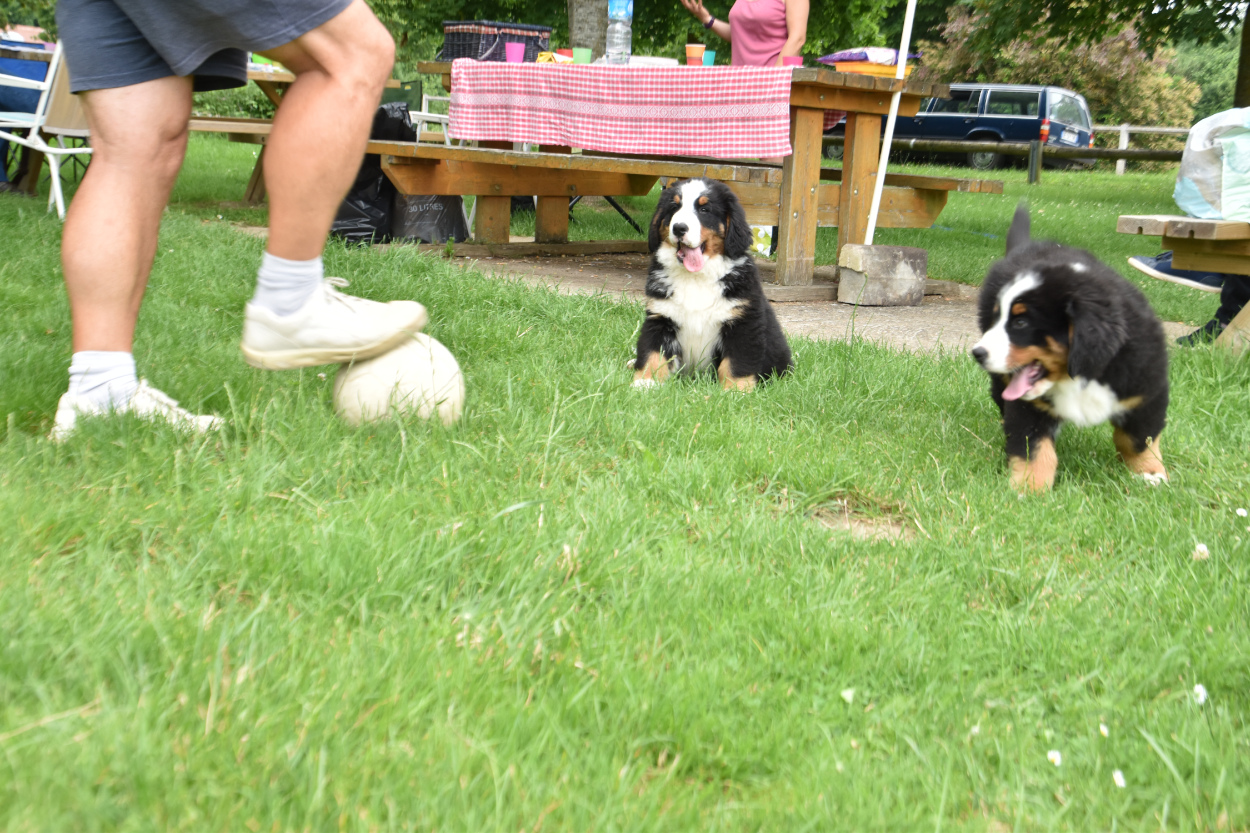

Supplement: S1 Data — (ZIP) [file pone.0234210.s002.zip › Pictures_DataBase_Environment/Clean/Clean - Rural/Clean - Rural - With Individuals/Rur.Pers.PROPRE11.1.JPG]

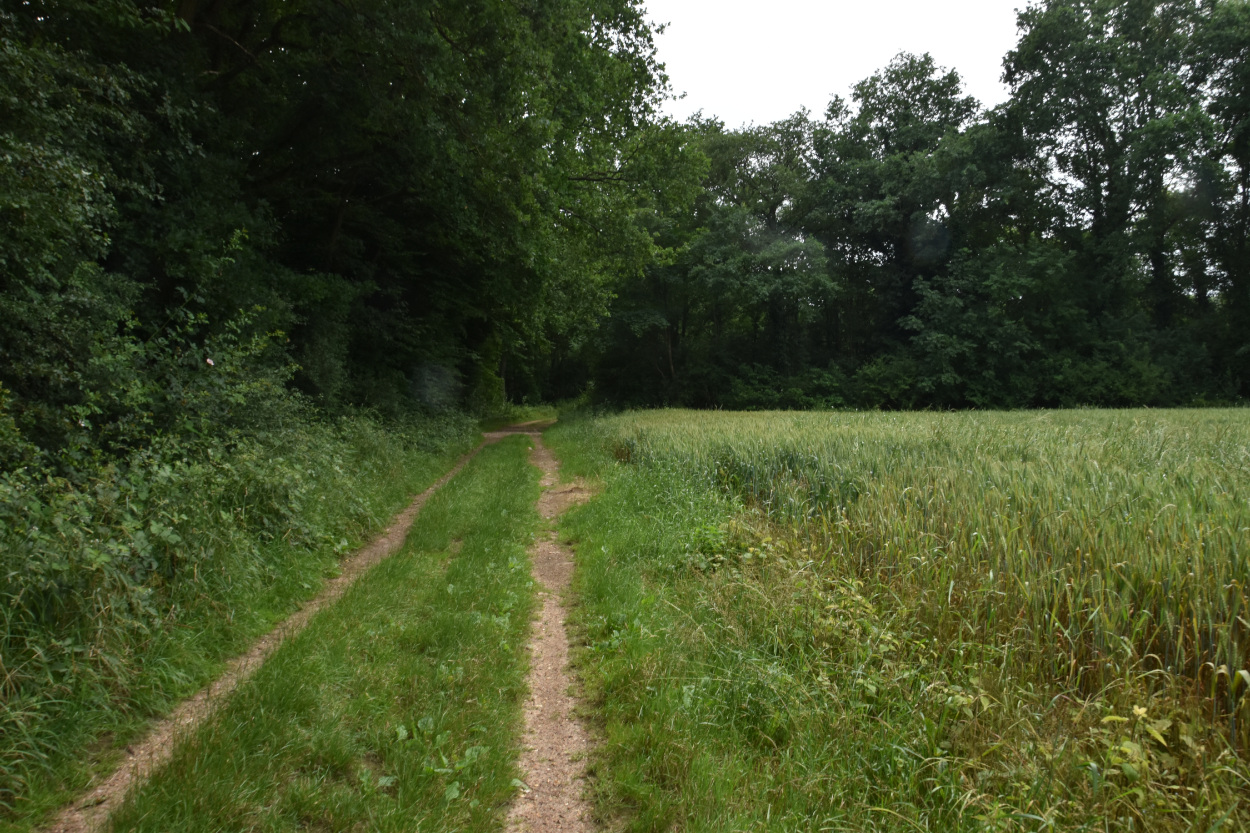

Supplement: S1 Data — (ZIP) [file pone.0234210.s002.zip › Pictures_DataBase_Environment/Clean/Clean - Rural/Clean - Rural - Without Individuals/Rur.PROPRE02.1.JPG]
